# Raising the Diversity of Ugi Reactions Through Selective Alkylations and Allylations of Ugi Adducts

**DOI:** 10.3389/fchem.2019.00020

**Published:** 2019-01-29

**Authors:** Alaa Zidan, Abeer M. El-Naggar, Nour E. A. Abd El-Sattar, Ali Khalil Ali, Laurent El Kaïm

**Affiliations:** ^1^Laboratoire de Synthèse Organique, CNRS, Ecole Polytechnique, ENSTA ParisTech, UMR 7652, Université Paris-Saclay, Palaiseau, France; ^2^Chemistry Department, Faculty of Science, Ain Shams University, Cairo, Egypt

**Keywords:** Ugi reaction, allylation, palladium, Tsuji-Trost reaction, metathesis, piperidines

## Abstract

We report here selective Tsuji-Trost type allylation of Ugi adducts using a strategy based on the enhanced nucleophilicity of amide dianions. Ugi adducts derived from aromatic aldehydes were easily allylated at their peptidyl position with allyl acetate in the presence of palladium catalysts. These substitutions were compared to more classical transition metal free allylations using allyl bromides.

## Introduction

**Scheme 1:**
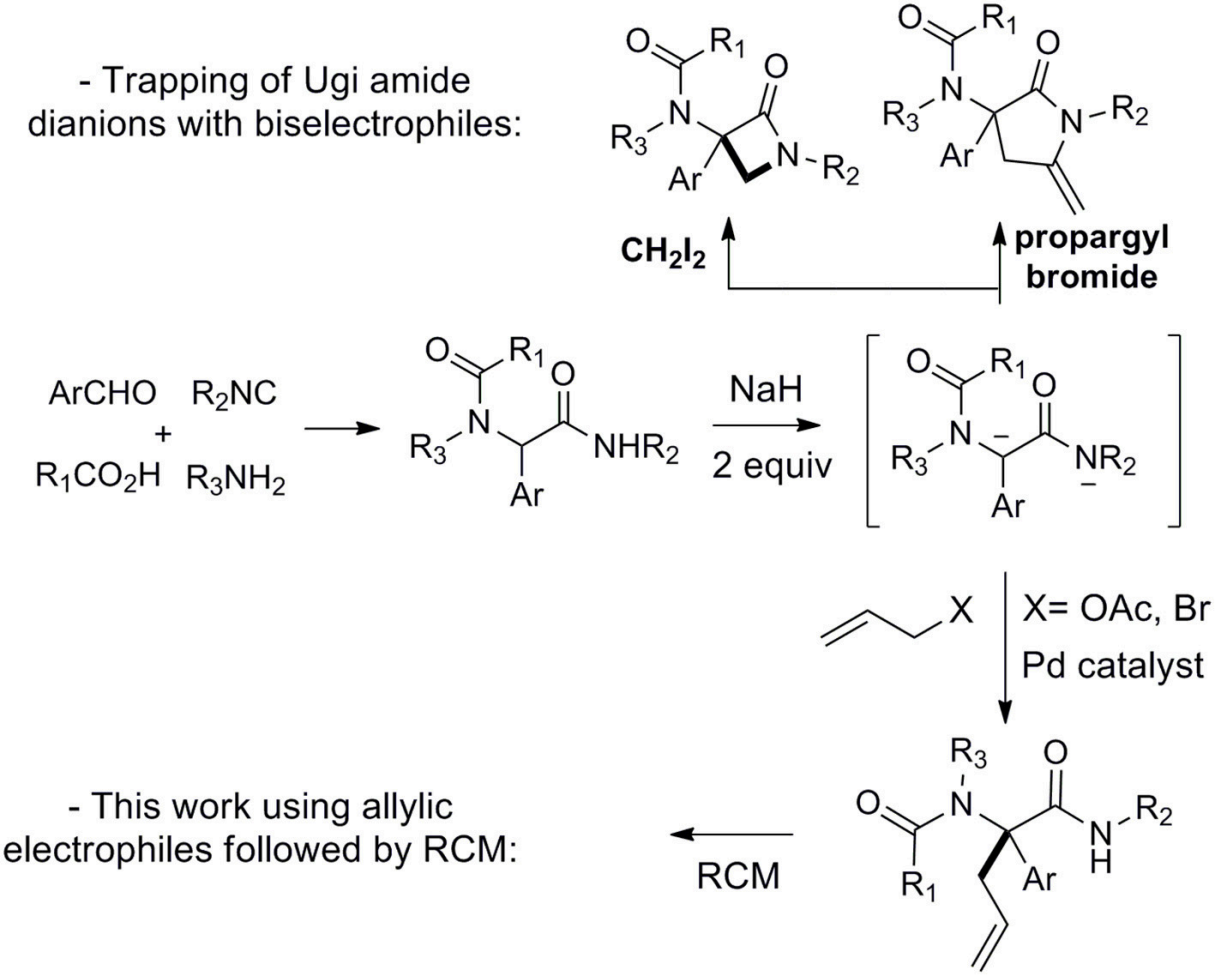
Reaction of Ugi amide dianion with mono and biselectrophiles.

Since its discovery and even more after the 1980's, the Ugi reaction has fascinated chemists with the high diversity brought by its four components nature together with its impressive functional tolerance (Dömling and Ugi, 2000; Hulme and Gore, 2003; Orru and De Greef, 2003; Zhu and Bienaymé, 2005; Dömling, 2006; Dömling et al., 2012; Zhu et al., 2014; Boyarskiy et al., 2015; Váradi et al., 2016; Lei et al., 2018). Besides important efforts devoted to the preparation of libraries of heterocycles through cyclization of properly functionalized Ugi adducts (Tempest, 2005; Sunderhaus and Martin, 2009; Ivachtchenko et al., 2010; Orru and Ruijter, 2010a,b; Sadjadi and Heravi, 2011; Eckert, 2012; Sharma et al., 2015), a number of studies have focused on raising the diversity by letting Ugi adducts react in further intermolecular couplings (Elders et al., 2009; Brauch et al., 2010; Zarganes-Tzitzikas et al., 2015a,b; Kaur et al., 2016). In contrast with the previous intramolecular couplings, these strategies (such as the combination of MCRs) are much more sensitive to steric hindrance and require more attention in selecting the functionalities required for further couplings. For this reason, most transformations involving the peptidyl position of Ugi adducts are limited to intramolecular reactions (Bossio et al., 1997; Trifilenkov et al., 2007; Salcedo et al., 2008; El Kaïm et al., 2011; Tyagi et al., 2013; Zhang et al., 2013; Ben Abdessalem et al., 2015; Ghandi et al., 2015; Vachhani et al., 2015; Li et al., 2016). We recently proposed a dianionic amide strategy to raise the nucleophilic behavior of Ugi adducts derived from aromatic aldehydes and demonstrated the interest of this approach using bis-electrophilic derivatives prone to trap the dianions and form heterocycles (Scheme 1) (Zidan et al., 2017, 2018). Following the high yielding cyclizations observed in these studies, we decided to explore more thoroughly the synthetic potential of these dianions (Thompson, 1994; Langer and Freiberg, 2004) toward more simple electrophiles. We now wish to report further applications of this chemistry in Tsuji-Trost reactions as well as metal-free alkylations with bromide derivatives (Scheme 1).

## Results and Discussion

Following our interest in Tsuji-Trost reactions involving isocyanide based MCRs (Dos Santos and El Kaïm, 2014; Cordier et al., 2015; El Mamouni et al., 2016), we decided to explore the behavior of Ugi adducts dianions toward allyl acetate. Besides the efficiency and regioselectivity issues of the reaction of these dianions with π-allyl palladium complexes, such study might bring interesting pathways for further enantioselective approaches. For this study, Ugi adduct **1a** was selected due to its good behavior in our previous studies with propargyl bromide and diiodomethane. It was prepared in 89% yield from 4-chloro-benzaldehyde, propylamine, acetic acid and *tert*-butylisocyanide (Table 1). When **1a** was heated with allyl acetate in THF using 2.5 equivalents of potassium *tert*-butoxide together with a Pd(dba)_2_/PPh_3_ catalytical couple, we were delighted to observe a selective C-allylation of **1a** giving **3a** in 65% isolated yield after 2 h refluxing (entry 1, Table 1).

**Table 1:** Screening of various conditions^a^.

**Entry**	**Base (equiv)**	**Solvent**	**Temp (^**°**^C)**	**Time (h)**	**Yield (%)**
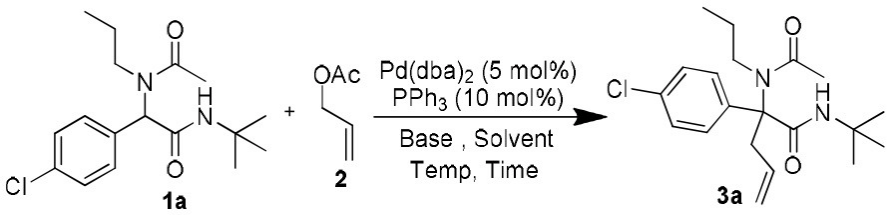					
1	*t*-BuOK(2.5)	THF	reflux	2	65
2	*t*-BuOK(2.5)	DMF	70	12	70
3	KHMDS (2.5)	THF	reflux	1	95
4	KHMDS (2.5)	THF	50	1	93
5	KHMDS (1.3)	THF	50	1	37
6	KHMDS (2.5)	THF	rt	1	88
7	NaH (2.5)	THF	reflux	12	45
8	NaH (2.5)	DMF	70	12	50
**9**	**NaH (2.5)**	**DMSO**	**rt**	**1**	**96**
10^b^	NaH (2.5)	DMSO	rt	12	Traces
11^c^	NaH (2.5)	DMSO	rt	12	80
12^d^	NaH (2.5)	DMSO	rt	1	94
13^e^	KHMDS (2.5)	THF	rt	12	-

a *Reaction conditions: **1a** (0.5 mmol), **2** (0.75 mmol), Pd(dba)_2_ (0.025 mmol), and PPh_3_ (0.05 mmol) in solvent (0.5 M)*.

b *Using Xantphos (5 mol%) instead of PPh_3_*.

c *Using Johnphos (10 mol%) instead of PPh_3_*.

d *Using Pd(PPh_3_)_4_ (5 mol%) instead of Pd(dba)_2_/PPh_3_*.

e *Reaction without Pd(dba)_2_ and PPh_3_. Bold font indicates the selected value for the optimization*.

Different bases in various solvents were then evaluated using the same palladium/phosphine couple. The enhanced nucleophilicty of the 1,3-amide dianion toward the π-allyl palladium cationic complex was confirmed experimentally by comparing the use of 2.5 and 1.3 equiv of KHMDS (affording respectively 93 and 37% isolated yields: entries 4 and 5, Table 1). NaH in DMSO, gave the best conditions affording to our delight a nearly quantitative yield of **3a** in only 1 h at room temperature (entry 9, Table 1). Further modifications of the catalyst using more complex phosphines led to longer reaction time and lower yields (entries 10–12, Table 1), while in absence of palladium source, no product was observed (entry 13, Table 1).

**Scheme 2:**
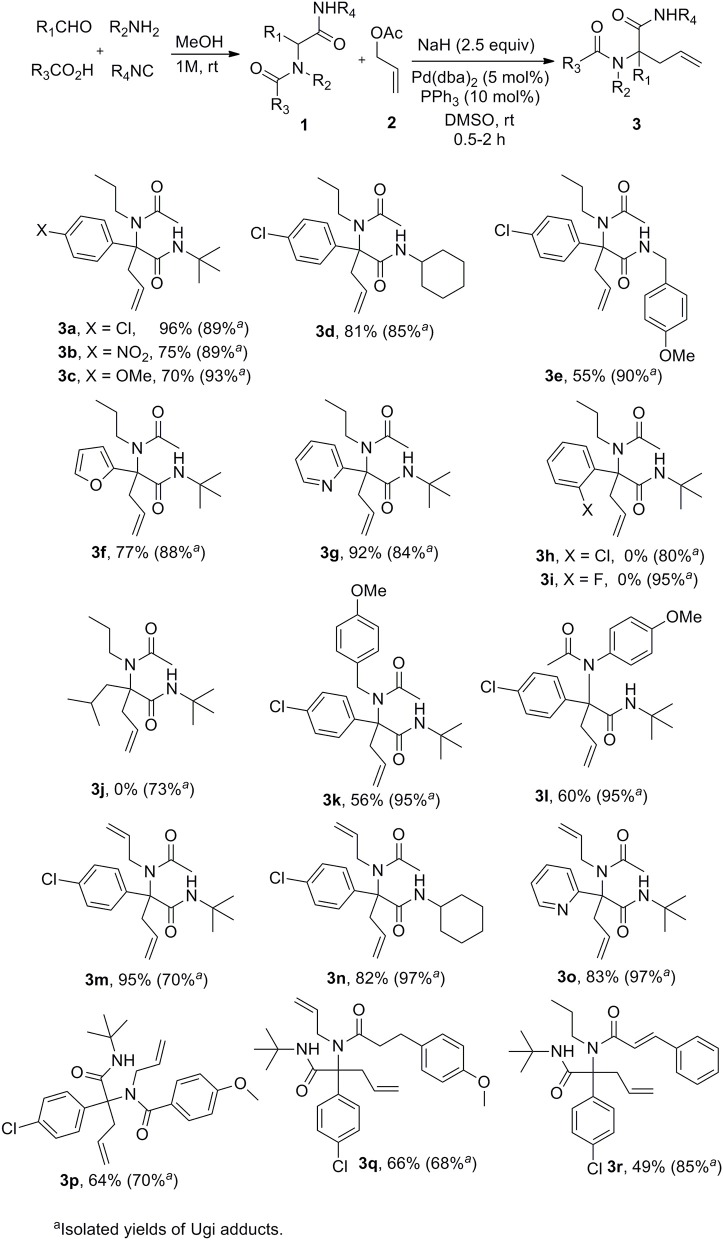
Scope of the Ugi/Tsuji-Trost cascade.

A set of Ugi adducts were then prepared in methanol and submitted to these optimized allylation conditions. The results are gathered in Scheme 2. As observed in our previous studies (Zidan et al., 2017, 2018), an aryl group tethering the peptidyl position is required for efficient allylation. Indeed, Ugi adduct **1j** prepared from isovaleraldehyde failed to form the expected **3j** probably due to the inability to form the dianionic intermediate with this less acidic substrate. Surprisingly, we didn't observe much correlation between yields and the electronic nature of the aldehyde as shown by the similar yields obtained with both 4-nitro or 4-methoxy substituted derivatives **3b** and **3c**. A much stronger effect of the substitution partner of the aromatic moiety was observed with 2-chloro substituted Ugi adduct **1h** which failed to give any adduct under these conditions as observed with **1j**. The same behavior was observed for the attempted synthesis of the fluoro analog **3i**. This lack of reactivity can probably be explained by the steric hindrance brought by substituents at the ortho position preventing to reach the planar geometry required for benzylic anion stabilization. Initial trials on enantioselective allylations were rather deceiving. When triphenylphosphine was replaced by BINAP (5mol %) for the reaction of **1a** with **2a**, **3a** could be formed rapidly in good yield (89%) but poor enantioselectivity (5%) whereas the use of Trost ligand (DACH-phenyl) failed to give any allylation reaction. A wider scope of chiral ligands as well as alternative solvents allowing lower temperatures will have to be evaluated to reach an enantioselective version. The reaction could not be extended to more substituted allylic acetate derivatives such as cinnamyl acetate. The latter failed to react with Ugi adduct **1a** even under prolonged heating, leading only to saponification of the ester and isolation of cinnamyl alcohol.

**Scheme 3:**
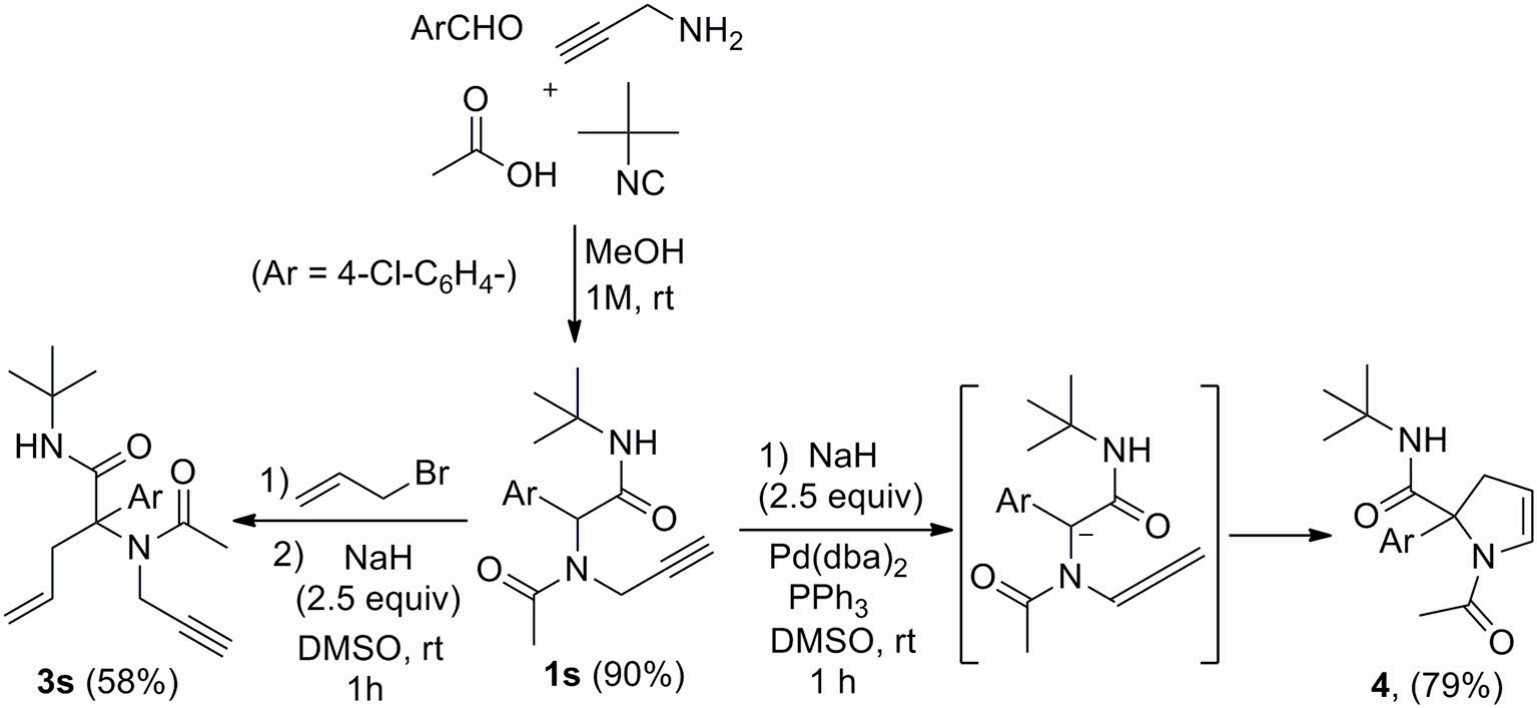
Allylation of Ugi propargyl adduct.

Submitting propargylamine-derived Ugi adduct **1s** to the same reaction conditions didn't afford any allylated product but resulted in the clean formation of 2,3-dihydropyrrole **4** (Scheme 3), a reaction already reported by Miranda et al. (Polindara-García and Miranda, 2012). They could isolate the same dihydropyrrole **4** in 36% yield treating **1s** with *t*-BuOK (2.5 equiv) in THF, while we could obtain 79% yield with our conditions. This selectivity was not very surprising when considering the respective intra and intermolecular properties of the two pathways. However, the ability to form enyne derivatives from Ugi adducts together with the interest of reversing a preferred selectivity were challenging enough to explore different set of conditions. Indeed, the dihydropyrrole formation probably involves a first isomerization into allene followed by further 5-endo-trig cyclization. This could leave some space for a previous intermolecular allylation if the lifetime of the dianion could be reduced by increasing the kinetic of the allylation step. Introducing the allyl acetate together with the palladium catalyst and the Ugi adduct in DMSO followed by the addition of the base resulted in a lower 54% yield of **4** together with a complex mixture of allylated products. We next explored the use of allyl bromide as a potential electrophilic species in the absence of palladium. When adding the latter to Ugi adduct **1s** followed by NaH, we were delighted to observe the expected enyne **3s** obtained in 58% isolated yield without any trace of dihydropyrrole (Scheme 3). This interesting control of the selectivity offers an attractive access to enyne derivatives for further cyclization studies.

**Scheme 4:**
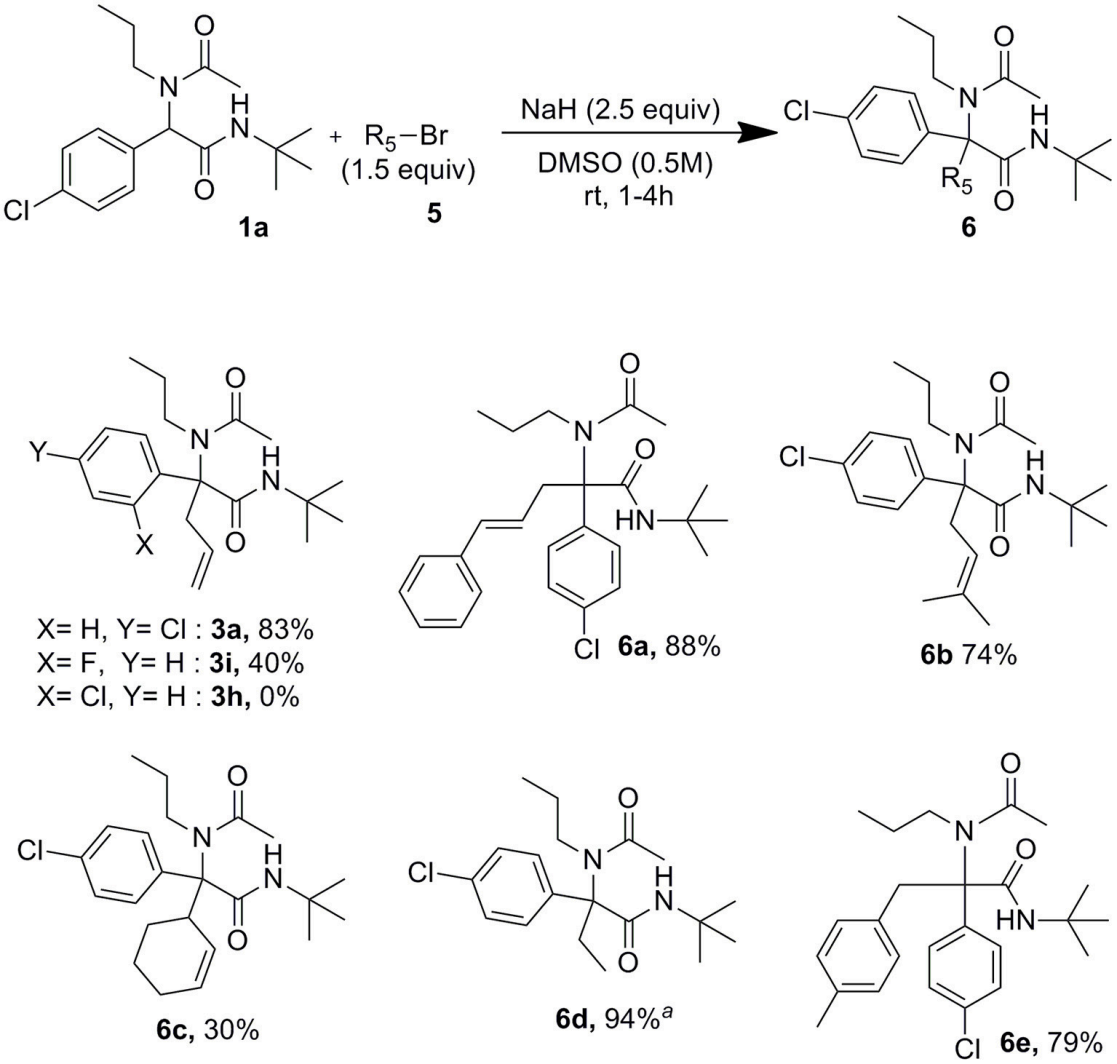
Scope of alkylating agents.

These conditions settled with allyl bromide proved useful as well for the reaction of other alkyl bromides and iodides as shown in Scheme 4. The lowest 30% yield of **6c** obtained using 3-bromocyclohex-1-ene may be explained by the use of secondary halides together with the high steric hindrance around the peptidyl position of the Ugi adduct. In order to confirm further the importance of forming dianionic intermediates, the formation of **3a** was attempted using allyl bromide in excess (1.5 equiv) but reducing the amount of sodium hydride to 0.9 equiv. In this case, only traces of allylated compound could be identified after 2 h at rt whereas the reaction was completed after the same time when using 2.5 equiv of sodium hydride. Interestingly, whereas under Tsuji-Trost type conditions we couldn't observe any allylation of both 2-chloro and 2-fluoro substituted derivatives **1h** and **1i**, the latter gave us a moderate 40% isolated yields of **3i** when using just sodium hydride with allyl bromide.

**Scheme 5:**
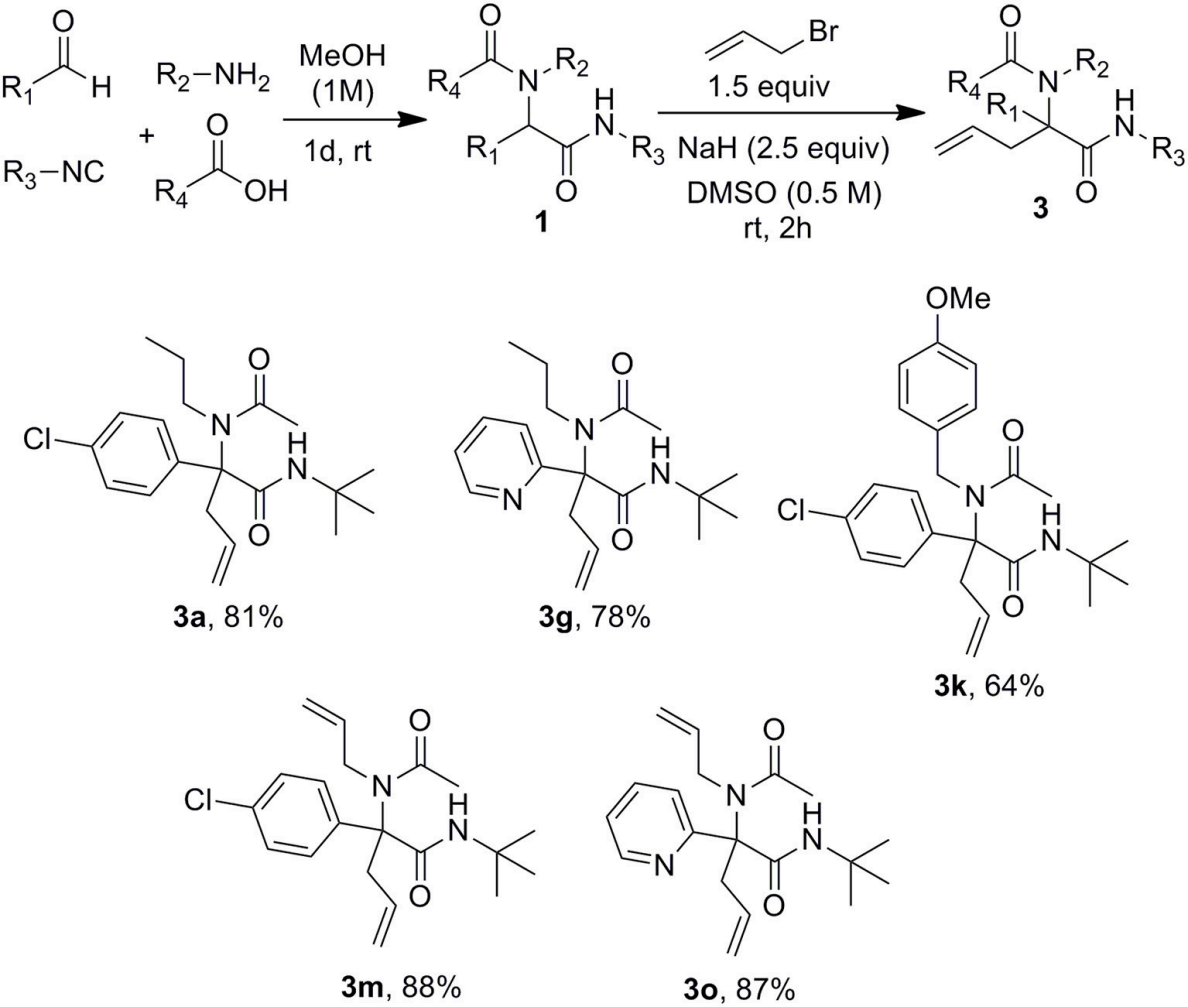
Cascade one pot reaction.

A further interest of the use of palladium free conditions may be found in the ability to carry the reaction starting directly from the four Ugi components. Indeed remaining isocyanides after the Ugi reaction are potentially inhibitor for most classical palladium catalyzed processes making thus one-pot processes difficult to achieve (El Kaim et al., 2008)^1^. After completion of the Ugi adduct, the methanol was evaporated under reduced pressure. The solvent was then replaced by DMSO. Addition of sodium hydride followed by allyl bromide afforded the final allylated Ugi adducts in good overall yields (Scheme 5).

**Scheme 6:**
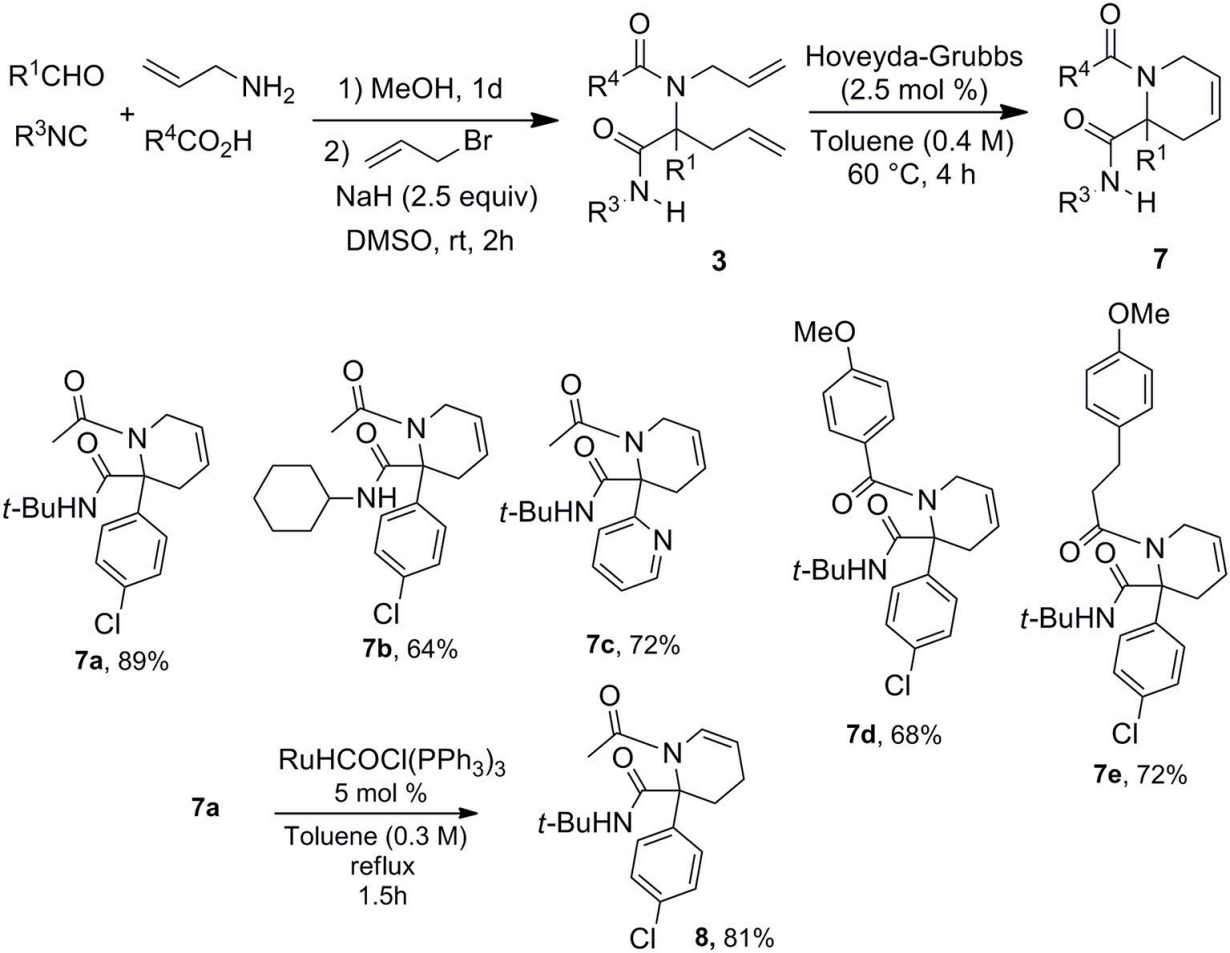
Ugi/allylation/RCM strategy.

With this easy allylation of Ugi adducts in hand, we decided to take advantage of the diversity offered by the latter coupling to explore Ugi/allylation/Ring Closure Methathesis (RCM) strategies toward various nitrogen based heterocycles. The use of RCM as Ugi post-condensation has already demonstrated its power for the formation of 5, 6-membered heterocycles as well as macrocyclic derivatives (Banfi et al., 2003; Beck et al., 2003; Hebach and Kazmaier, 2003; Ribelin et al., 2007; Ku et al., 2011). However, the synthetic potential of these strategies is in a way limited by the need of introducing the two allylated moieties at an early stage which reduces the diversity offered by the two-step process. The late stage allylation we propose is highly versatile allowing to settle the strategy with a single alkenyl moiety in the starting components. Thus using allyl amine, we could easily prepare a library of piperidines with five points of diversity (Scheme 6).

**Scheme 7:**
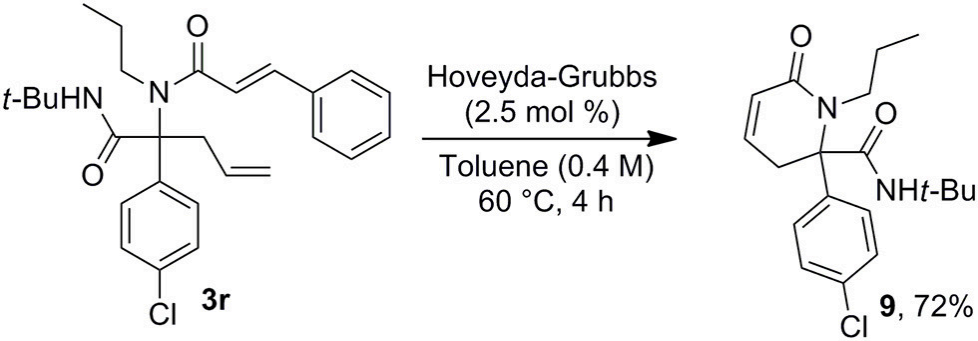
Ugi/allylation/MCR toward piperidones.

This access to 3,4-dehydropiperidine derivatives **7** could be further enriched by a potential isomerization into cyclic enamines as demonstrated by the ruthenium hydride catalyzed conversion of **7a** into **8** (Scheme 6). The versatility of the method can be pictured by the alternative choice of the alkenyl moiety on the acidic component such as in cinnamic acid offering now a very simple access to 3,4-dehydropiperidine-2-one **9** (Scheme 7).

## Conclusion

In summary, we have extended the potential of the Ugi reaction using 1,3-amide dianionic species as intermediates for efficient allylations at the peptidyl moiety of Ugi adducts. The procedure raises the diversity of Ugi adducts and offers unique opportunities for the preparation of nitrogen based heterocycles through association with ring closure metathesis (Supplementary Data Sheet 1). The power of the latter strategy has been demonstrated by the preparation of various piperidines which are important scaffolds for medicinal applications.

## Author Contributions

AZ was responsible for designing and performing the experiments. AZ, AE-N, NA, AA, and LE discussed the evolution of the project and revised the manuscript together. LE and AA directed the project and wrote the publication.

### Conflict of Interest Statement

The authors declare that the research was conducted in the absence of any commercial or financial relationships that could be construed as a potential conflict of interest.

## Footnotes

^1^However, one-pot processes may be envisioned if the remaining isocyanide is destroyed by an acidic hydrolysis prior palladium addition.
