# Supplementary material for: Raising the Diversity of Ugi Reactions Through Selective Alkylations and Allylations of Ugi Adducts
Source: Front Chem. 2019 Jan 29;7:20. doi: 10.3389/fchem.2019.00020 (PMC6361783; doi:10.3389/fchem.2019.00020)

Experimental Data

Raising the diversity of Ugi reactions through selective alkylations and allylations of Ugi adducts

Alaa Zidan,[a],[b] Abeer M. El-Naggar,[b] Nour E. A. Abd El-Sattar,[b] Ali Khalil Ali*[b] and Laurent El Kaim*[a]

[a] Laboratoire de Synthèse Organique, CNRS, Ecole Polytechnique, ENSTA ParisTech-UMR 7652, Université Paris-Saclay, 828 Bd des Maréchaux, 91128 Palaiseau, France. [b] Chemistry Department, Faculty of Science, Ain Shams University, Abbasia, Cairo 11566, Egypt.

Table of Contents

General Information E3

Ugi reaction E4

- **General procedure I** E4
- **Synthesis of Ugi adducts (1a-1s)** E4

Tsuji-Trost reaction of Ugi adducts E16

- **General procedure II** E16
- **Synthesis of allylated Ugi adducts (3a-3r)** E16

Synthesis of 2,3-dihydropyrrole derivative (4) E26

General Alkylation reaction E28

- **General procedure III** E28
- **Synthesis of Ugi 5CP (3a, 3i, 3s, 6a-6e)** E28

Cascade reaction E34

- **General procedure IV** E34
- **Synthesis of some allylated Ugi adducts** E34

Ring Closure Metathesis **E37**

- **General procedure V** **E37**
- **Synthesis of 3,4-dehydropiperidine derivatives (7a-7e), (9)** **E37**

Isomerization of double bond **E42**

- **Synthesis of isomer 8** **E42**

NMR spectra **S43**

Mass Spectra **S92**

General Information

All reactions requiring anhydrous conditions were conducted in dried apparatus under an inert atmosphere of argon. All commercial materials were used without further purification. Sodium hydride used is 60% oil dispersion. Reactions were followed by thin-layer chromatography (TLC) performed using precoated plates of silica 60 F_{254}, U.V light as a visualizing agent and $KMnO_4$ stain with heat as developing agent. Colomn chromatography was carried out on silica gel (40-63 µm). ^{1}H-NMR spectra were recorded on a Brucker Avance 400 MHz spectrometer, using $CDCl_3$ as solvent. ^{13}C-NMR spectra were recorded on a 100.6 MHz spectrometer using $CDCl_3$ as solvent. Chemical shifts are expressed in ppm relative to internal TMS. Coupling constants (*J)* are quoted in hertz (Hz), data are reported as follows: chemical shift, multiplicity (s = singlet, d = doublet, t = triplet, q = quartet, br = broad, m = multiplet), coupling constant (Hz), integration. High-Resolution Mass spectra (HRMS) were carried out with JEOL JMS-GCmate II spectrometer. IR spectra were performed on a Perkin-Elmer FT 1600 spectrometer with wavelengths in cm^{-1} and only peaks of interest are reported. Melting points (mp) were determined on a Stuart SMP3 apparatus and were left uncorrected.

General procedure I for Ugi products

To a 1 M solution of aldehyde in methanol were added successively 1.0 equiv of amine, 1.0 equiv of acid and 1.0 equiv of isocyanide. The resulting mixture was stirred at room temperature for 1 day. The solvent was removed under reduced pressure and the crude was purified by flash colomn chromatography on silica gel to afford Ugi product.

N-(tert-butyl)-2-(4-chlorophenyl)-2-(N-propylacetamido)acetamide (1a)

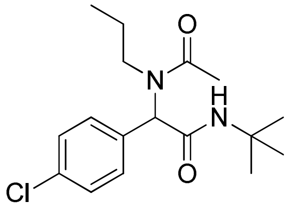

1a

This compound was synthesized according to the general procedure **I**, using 4-chlorobenzaldehyde (141 mg, 1.0 mmol), propylamine (0.08 ml, 1.0 mmol), acetic acid (0.06 ml, 1.0mmol) and tert-butyl isocyanide (0.11 ml, 1.0 mmol). Purification by flash chromatography using (Et_2O : PE = 70 : 30) gave the desired product in 89% isolated yield (288 mg, 0.89 mmol) as white solid. **m.p. =**173-175 °C. $\mathbf{R_f}$ (Et_2O : PE = 7 : 3) = 0.3. ^{1}**H-NMR** ($CDCl_3$, 400 MHz): δ (ppm) 7.27 (br s, 4H), 5.83 (br s, 1H), 5.70 (s, 1H), 3.18 (t, *J* = 8.2 Hz, 2H), 2.11 (s, 3H), 1.43-1.36 (m, 1H), 1.26 (s, 9H), 1.02-0.92 (m, 1H), 0.64 (t, *J* = 7.4 Hz, 3H). ^{13}**C-NMR** ($CDCl_3$, 100.6 MHz): δ (ppm) 171.6, 168.9, 134.5, 134.2, 130.6, 128.9, 61.8, 51.6, 49.5, 28.6, 23.0, 21.9, 11.2. **HRMS:** Calculated for $C_{17}H_{25}ClN_2O_2$: 324.1605, Found: 324.1603. **I.R.** (thin film): ν 3426, 2970, 2935, 2877, 1679, 1633, 1514, 1252, 1140, 1094 and 1016 cm^{-1}.

N-(tert-butyl)-2-(4-nitrophenyl)-2-(N-propylacetamido)acetamide (1b)

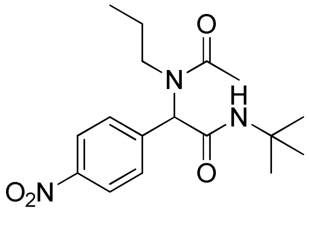

1b

This compound was synthesized according to the general procedure **I**, using 4-nitrobenzaldehyde (151 mg, 1.0mmol), propylamine (0.08 ml, 1.0mmol), acetic acid (0.06 ml, 1.0mmol) and tert-butyl isocyanide (0.11 ml, 1.0mmol). Purification by flash chromatography using (EtOAc : PE = 50 : 50) gave the desired product in 89% isolated yield (297 mg, 0.89 mmol) as white solid. **m.p.** =96-98°C. **R_f** (Et_2O : PE = 9:1) = 0.37. **^{1}H-NMR** ($CDCl_3$, 400 MHz): δ (ppm) 8.14 (d, *J* = 8.7 Hz, 2H), 7.48 (d, *J* = 8.6 Hz, 2H), 6.27 (br s, 1H), 5.69 (s, 1H), 3.26 (t, *J* = 8.1 Hz, 2H), 2.15 (s, 3H), 1.52-1.44 (m, 1H), 1.29 (s, 9H), 1.19-1.13 (m, 1H), 0.71 (t, *J* = 7.4 Hz, 3H). **^{13}C-NMR** ($CDCl_3$, 100.6 MHz): δ (ppm) 171.8, 168.2, 147.5, 143.5, 129.4, 123.7, 62.9, 51.8, 50.4, 28.6, 22.9, 21.9, 11.2. **HRMS:** Calculated for $C_{17}H_{25}N_3O_4$: 335.1845, Found: 335.1837. **I.R.** (thin film): ν 3426, 2970, 2935, 2877, 1674, 1633, 1607, 1524, 1350, 1252 and 1222 cm^{-1}.

N-(tert-butyl)-2-(4-methoxyphenyl)-2-(N-propylacetamido)acetamide (1c)

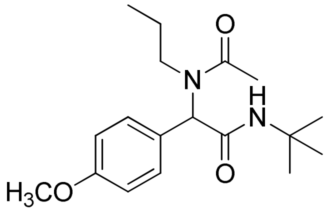

1c

This compound was synthesized according to the general procedure **I**, using 4-methoxybenzaldehyde (0.12 ml, 1.0 mmol), propylamine (0.08 ml, 1.0mmol), acetic acid (0.06 ml, 1.0mmol) and tert-butyl isocyanide (0.11 ml, 1.0mmol). Purification by flash chromatography using (EtOAc : PE = 60 : 40) gave the desired product in 93% isolated yield (298 mg, 0.93 mmol) as white solid. **m.p.** =170-172 °C. **R_f:** (Et_2O : PE = 9:1) = 0.34. **^{1}H-NMR** ($CDCl_3$, 400 MHz): δ (ppm) 7.25 (d, *J* = 8.6 Hz, 2H), 6.81 (d, *J* = 8.7 Hz, 2H), 5.73 (s, 1H), 5.61 (br s, 1H), 3.75 (s, 3H), 3.16 (t, *J* = 8.2 Hz, 2H), 2.10 (s, 3H), 1.34-1.28 (m, 1H), 1.26 (s, 9H), 0.91-0.81 (m, 1H), 0.59 (t, *J* = 7.4 Hz, 3H). **^{13}C-NMR** ($CDCl_3$, 100.6 MHz): δ (ppm) 171.4, 169.5, 159.6, 130.8, 127.9, 114.1, 61.6, 55.3, 51.5, 49.1, 28.7, 23.1, 21.9, 11.3. **HRMS:** Calculated for $C_{18}H_{28}N_2O_3$: 320.2100, Found: 320.2099. **I.R.** (thin film): ν 3425, 2969, 2935, 2876, 1683, 1625, 1512, 1251, 1180 and 1035 cm^{-1}.

2-(4-chlorophenyl)-N-cyclohexyl-2-(N-propylacetamido)acetamide (1d)

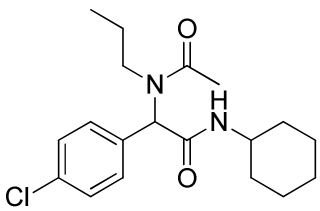

1d

This compound was synthesized according to the general procedure **I**, using 4-chlorobenzaldehyde (141 mg, 1.0 mmol), propylamine (0.08 ml, 1.0 mmol), acetic acid (0.06 ml, 1.0 mmol) and cyclohexylisocyanide (109 mg, 1.0 mmol). Purification by flash chromatography using (Et_2O : PE = 90 : 10) gave the desired product in 85% isolated yield (320 mg, 0.85 mmol) as white solid. **m.p. =** 189-191°C. $\mathbf{R_f}$ (Et_2O : PE = 9 : 1) = 0.39. ^{1}**H-NMR** ($CDCl_3$, 400 MHz): δ (ppm) 7.26 (br s, 4H), 6.06 (br s, 1H), 5.73 (s, 1H), 3.74-3.67 (m, 1H), 3.19 (t , *J* = 8.0 Hz, 2H), 2.10 (s, 3H), 1.82-1.80 (m, 2H), 1.61-1.49 (m, 3H), 1.44-1.36 (m, 1H), 1.31-1.22 (m, 2H), 1.13-1.01 (m, 4H), 0.66 (t, *J* = 7.3 Hz, 3H). ^{13}**C-NMR** ($CDCl_3$, 100.6 MHz): δ (ppm) 171.6, 168.6, 134.4, 134.2, 130.5, 128.9, 61.6, 49.7, 48.5, 32.8, 32.7, 25.5, 24.8, 24.7, 22.9, 21.9, 11.2. **HRMS:** Calculated for $C_{19}H_{27}ClN_2O_2$: 350.1761, Found: 350.1678. **I.R.** (thin film): ν 3425, 2936, 2857, 1677, 1630, 1514, 1491, 1416, 1251 and 1094 cm^{-1}.

2-(4-chlorophenyl)-N-(4-methoxybenzyl)-2-(N-propylacetamido)acetamide (1e)

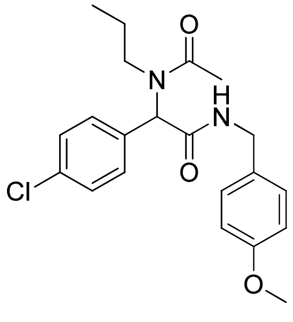

1e

This compound was synthesized according to the general procedure **I**, using 4-chlorobenzaldehyde (141 mg, 1.0 mmol), propylamine (0.08 ml, 1.0 mmol), acetic acid (0.06 ml, 1.0 mmol) and 1-(isocyanomethyl)-4-methoxybenzene (147 mg, 1.0 mmol). Purification by flash

chromatography using (EtOAc : DCM = 20 : 80) gave the desired product in 90% isolated yield (350 mg, 0.9 mmol) as white solid. **m.p. =** 95-97 °C. **R_f** (Et_2O : PE = 9:1) = 0.29. **^{1}H-NMR** ($CDCl_3$, 400 MHz): δ (ppm) 7.23 (br s, 4H), 7.08 (d, J = 8.5 Hz, 2H), 6.74 (d, J = 8.6 Hz, 2H), 6.59 (br s, 1H), 5.79 (s, 1H), 4.29 (dd, ABX system, J_{ab} = 14.6, J_{ax} = 5.5, J_{bx} = 5.8 Hz, 2H), 3.69 (s, 3H), 3.20-3.15 (m, 2H), 2.04 (s, 3H), 1.4-1.3 (m, 1H), 1.01-0.90 (m, 1H), 0.62 (t, J = 7.4 Hz, 3H). **^{13}C-NMR** ($CDCl_3$, 100.6 MHz): δ (ppm) 171.7, 169.5, 158.9, 134.4, 134.2, 130.7, 130.1, 129.1, 128.9, 114.0, 61.5, 55.3, 49.6, 43.1, 23.0, 21.8, 11.2. **HRMS:** Calculated for $C_{21}H_{25}ClN_2O_3$: 388.1554, Found: 388.1569. **I.R.** (thin film): ν 3433, 3067, 3035, 3004, 2967, 2935, 2878, 2839, 1676, 1631, 1513, 1301, 1250, 1176, 1094 and 1035 cm^{-1}.

N-(tert-butyl)-2-(furan-2-yl)-2-(N-propylacetamido)acetamide (1f)

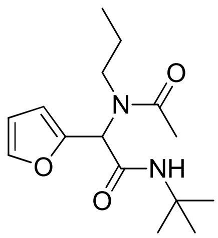

1f

This compound was synthesized according to the general procedure **I**, using 2-furanaldehyde (0.08 ml, 1.0mmol), propylamine (0.08 ml, 1.0mmol), acetic acid (0.06 ml, 1.0mmol) and tert-butyl isocyanide (0.11 ml, 1.0mmol). Purification by flash chromatography using (Et_2O : DCM = 10 : 90) gave the desired product in 88% isolated yield (248 mg, 0.88mmol) as white solid. **m.p.** =113-115 °C. **R_f:** (Et_2O : PE = 9:1) = 0.42. **^{1}H-NMR** ($CDCl_3$, 400 MHz): δ (ppm) 7.45 (d, J = 1.2 Hz, 1H), 6.68 (d, J = 3.2 Hz, 1H), 6.43 (dd, J = 1.9, 3.2 Hz, 1H), 6.06 (br s, 1H), 6.02 (s, 1H), 3.34 (ddd, ABX_2 system, J= 15.6, 10.8, 5.6 Hz, 1H), 3.21 (ddd, ABX_2 system, J= 15.6, 10.8, 5.2 Hz, 1H), 2.21 (s, 3H), 1.54-1.45 (m, 1H), 1.37 (s, 9H), 1.10-0.99 (m, 1H), 0.78 (t, J = 7.4 Hz, 3H). **^{13}C-NMR** ($CDCl_3$, 100.6 MHz): δ (ppm) 171.4, 167.2, 148.9, 142.7, 111.6, 110.8, 55.8, 51.5, 48.9, 28.6, 22.5, 21.7, 11.2. **HRMS:** Calculated for [M-CONHt-Bu] $C_{10}H_{14}NO_2^+$: 180.1019, Found: 180.1022 (86%). **I.R.** (thin film): ν 3425, 2970, 2934, 2877, 1687, 1627, 1517, 1419, 1223 and 1015 cm^{-1}.

N-(tert-butyl)-2-(N-propylacetamido)-2-(pyridin-2-yl)acetamide (1g)

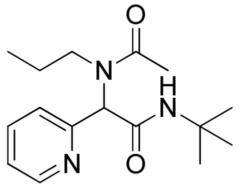

1g

This compound was synthesized according to the general procedure **I**, using pyridine-2-carboxaldehyde (0.1 ml, 1.0 mmol), propylamine (0.08 ml, 1.0 mmol), acetic acid (0.06 ml, 1.0 mmol) and tert-butyl isocyanide (0.11 ml, 1.0 mmol). Purification by flash chromatography using pure EtOAc gave the desired product in 84% isolated yield (244 mg, 0.84 mmol) as yellow solid. **m.p. =** 129-131°C. **R$_f$** (pure EtOAc) = 0.33. **^{1}H-NMR** ($CDCl_3$, 400 MHz): δ (ppm) 8.60 (d, *J* = 4.4 Hz, 1H), 7.72 (td, *J* = 8.0, 1.6 Hz, 1H), 7.47 (d, *J* = 8.0 Hz, 1H), 7.37 (br s, 1H), 7.26 (dd, *J* = 7.2, 5.2 Hz, 1H), 5.65 (s, 1H), 3.43 (t, *J* = 8.2 Hz, 2H), 2.24 (s, 3H), 1.65-1.56 (m, 1H), 1.40-1.33 (m, 10H), 0.84 (t, *J* = 7.4 Hz, 3H). **^{13}C-NMR** ($CDCl_3$, 100.6 MHz): δ (ppm) 171.4, 167.7, 156.6, 148.7, 137.0, 123.6, 122.6, 66.7, 51.3, 51.2, 28.7, 22.8, 21.9, 11.3. **HRMS:** Calculated for $C_{16}H_{25}N_3O_2$: 291.1947, Found: 291.1954. **I.R.** (thin film): ν 3427, 3061, 2969, 2934, 2877, 1655, 1592, 1573, 1550, 1516, 1253, 1224, 1140, 1036 cm^{-1}.

N-(tert-butyl)-2-(2-chlorophenyl)-2-(N-propylacetamido)acetamide (1h)

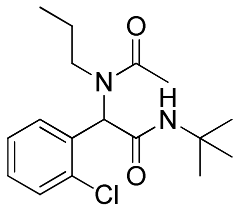

1h

This compound was synthesized according to the general procedure **I**, using 2-chlorobenzaldehyde (141 mg, 1.0 mmol), propylamine (0.08 ml, 1.0 mmol), acetic acid (0.06 ml, 1.0 mmol) and tert-butyl isocyanide (0.11 ml, 1.0 mmol). Purification by flash chromatography using (Et_2O : DCM = 15 : 85) gave the desired product in 80 % isolated yield (261 mg, 0.8 mmol) as white solid. **m.p. =** 187-189 °C. **R$_f$** (Et_2O : PE = 7 : 3) = 0.3. **^{1}H-NMR** ($CDCl_3$, 400 MHz): δ (ppm) 7.68-7.66 (m, 1H), 7.47-7.45 (m, 1H), 7.37-7.34 (m, 2H), 6.08 (s, 1H), 5.69 (br s, 1H), 3.26 (dt, ABX_2 system, J_{ab} = 10.4, J_{ax} = J_{bx} = 5.6 Hz, 2H), 2.23 (s, 3H), 1.52-1.47 (m, 1H),

1.39 (s, 9H), 1.00-0.91 (m, 1H), 0.68 (t, *J* = 7.4 Hz, 3H). **^{13}C-NMR** ($CDCl_3$, 100.6 MHz): δ (ppm) 171.3, 168.8, 135.8, 133.7, 130.8, 129.9, 127.1, 59.9, 51.7, 49.3, 28.6, 22.7, 21.8, 11.2. **HRMS:** Calculated for $C_{17}H_{25}ClN_2O_2$: 324.1605, Found: 324.1614. **I.R.** (thin film): ν 3426, 2970, 2935, 2877, 1679, 1633, 1514, 1252, 1140, 1094 and 1016 cm^{-1}.

N-(tert-butyl)-2-(2-fluorophenyl)-2-(N-propylacetamido)acetamide (1i)

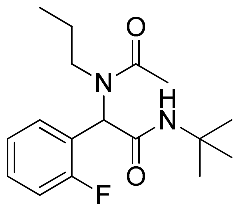

1i

This compound was synthesized according to the general procedure **I**, using 2-fluorobenzaldehyde (0.11 ml, 1.0 mmol), propylamine (0.08 ml, 1.0 mmol), acetic acid (0.06 ml, 1.0 mmol) and tert-butyl isocyanide (0.11 ml, 1.0 mmol). Purification by flash chromatography using (Et_2O : DCM = 15 : 85) gave the desired product in 95 % isolated yield (294 mg, 0.95 mmol) as white solid. **m.p. =** 155-157 °C. **R_f** (Et_2O : DCM = 1.5 : 8.5) = 0.26. **^{1}H-NMR** ($CDCl_3$, 400 MHz): δ (ppm) 7.59 (td, *J* = 7.6, 1.3 Hz, 1H), 7.37 (td, *J* = 7.4, 1.6 Hz, 1H), 7.18 (t, *J* = 7.3 Hz, 1H), 7.12-7.06 (m, 1H), 6.05 (s, 1H), 5.95 (br s, 1H), 3.29 (dd, *J* =16.7, 8.6 Hz, 2H), 2.19 (s, 3H), 1.53 – 1.44 (m, 1H), 1.36 (s, 9H), 1.05-1.02 (m, 1H), 0.70 (t, *J* = 7.4 Hz, 3H). **^{13}C-NMR** ($CDCl_3$, 100.6 MHz): δ (ppm) 171.3, 168.6, 161.3 (d, *J* = 248.9 Hz), 130.8 (d, *J* = 2.8 Hz), 130.4(d, *J* = 8.3 Hz), 124.3 (d, *J* = 3.5 Hz), 123.4 (d, *J* = 13.7 Hz), 115.6 (d, *J* = 21.9 Hz), 56.4, 51.6, 49.5, 28.6, 22.9, 21.8, 11.2. **HRMS:** Calculated for [M-CONH*t*-Bu] $C_{12}H_{16}FNO$: 209.1216, Found: 209.1213. **I.R.** (thin film): ν 3308, 3070, 2965, 2933, 2874, 1680, 1626, 1549, 1453, 1363, 1224, 1097, 1033, 921 and 728 cm^{-1}.

N-(tert-butyl)-4-methyl-2-(N-propylacetamido)pentanamide (1j)

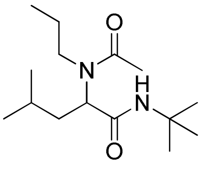

1j

This compound was synthesized according to the general procedure **I**, using 3-methylbutanal (0.11 ml, 1.0 mmol), propylamine (0.08 ml, 1.0 mmol), acetic acid (0.06 ml, 1.0 mmol) and tert-butyl isocyanide (0.11 ml, 1.0 mmol). Purification by flash chromatography using (Et_2O : DCM = 10 : 90) gave the desired product in 73% isolated yield (197 mg, 0.73mmol) as colorless oil. **R_f:** (Et_2O : DCM = 1 : 9) = 0.28. **^{1}H-NMR** ($CDCl_3$, 400 MHz): δ (ppm) 6.37 (br s, 1H), 4.78 (t, J = 7.6 Hz, 1H), 3.16-3.08 (m, 2H), 2.08 (s, 3H), 1.72-1.65 (m, 1H), 1.61-1.55 (m, 1H), 1.51-1.38 (m, 3H), 1.23 (s, 9H), 0.83 (2d, J = 6.4, 6.8 Hz, 1t, J = 7.6 Hz, 9H). **^{13}C-NMR** ($CDCl_3$, 100.6 MHz): δ (ppm) 172.1, 170.8, 55.6, 50.9, 47.4, 36.6, 28.6, 24.9, 23.1, 22.9, 22.4, 21.9, 11.5. **HRMS:** Calculated for $C_{15}H_{30}N_2O_2$: 270.2307, Found: 270.1915. **I.R.** (thin film): ν 3425, 2965, 2935, 2874, 1679, 1620, 1530, 1455, 1367, 1253, 1129 and 1024 cm^{-1}.

N-(tert-butyl)-2-(4-chlorophenyl)-2-(N-(4-methoxybenzyl)acetamido)acetamide (1k)

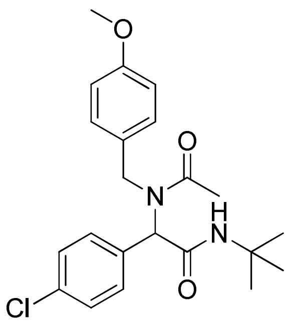

1k

This compound was synthesized according to the general procedure **I**, using 4-chlorobenzaldehyde (141 mg, 1.0 mmol), 4-methoxybenzylamine (0.13 ml, 1.0 mmol), acetic acid (0.06 ml, 1.0 mmol) and tert-butyl isocyanide (0.11 ml, 1.0 mmol). Purification by flash chromatography using (Et_2O : PE = 70 : 30) gave the desired product in 95% isolated yield (384 mg, 0.95mmol) as white solid. **m.p. =**124-126 °C. **R_f:** (EtOAc : PE = 4 : 6) = 0.26. **^{1}H-NMR** ($CDCl_3$, 400 MHz): δ (ppm) 7.30 (d, J = 9.2 Hz, 2H), 7.24 (d, J = 8.4 Hz, 2H), 6.89 (d, J = 8.4 Hz, 2H), 6.75 (d, J = 8.4 Hz, 2H), 5.90 (m, 2H), 4.61 (d, AB system, J_{ab} = 17.6 Hz, 1H), 4.52 (d, AB system, J_{ab} = 17.2 Hz, 1H), 3.78 (s, 3H), 2.12 (s, 3H), 1.35 (s, 9H). **^{13}C-NMR** ($CDCl_3$, 100.6 MHz): δ (ppm) 172.6, 168.7, 158.6, 134.3, 134.1, 130.9, 129.4, 128.8, 127.3, 113.9, 61.9, 55.3, 51.7, 50.2, 28.6, 22.6. **HRMS:** Calculated for [M-CON*t*-Bu] $C_{17}H_{18}ClNO_2$: 303.1026, Found: 303.1024. **I.R.** (thin film): ν 3426, 2968, 2936, 2873, 2839, 1684, 1645, 1513, 1409, 1248, 1176, 1094, 1035 cm^{-1}.

N-(tert-butyl)-2-(4-chlorophenyl)-2-(N-(4-methoxyphenyl)acetamido)acetamide (1l)

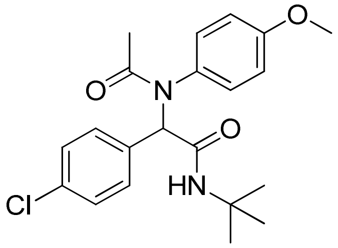

1l

This compound was synthesized according to the general procedure **I**, using 4-chlorobenzaldehyde (141 mg, 1.0 mmol), 4-methoxyaniline (0.12 ml, 1.0 mmol), acetic acid (0.06 ml, 1.0 mmol) and tert-butyl isocyanide (0.11 ml, 1.0 mmol). Purification by flash chromatography using (Et_2O : PE = 80 : 20) gave the desired product in 95 % isolated yield (371 mg, 0.95 mmol) as white solid. **m.p. =** 214-216 °C. **R$_f$** (EtOAc : PE = 4 : 6) = 0.29. **^{1}H-NMR** ($CDCl_3$, 400 MHz): δ (ppm) 7.19 (d, *J* = 8.4 Hz, 2H), 7.10 (d, *J* = 8.5 Hz, 2H), 6.75 (br s, 2H), 6.00 (s, 1H), 5.75 (br s, 1H), 3.80 (s, 3H), 1.89 (s, 3H), 1.38 (s, 9H). **^{13}C-NMR** ($CDCl_3$, 100.6 MHz): δ (ppm) 171.8, 168.7, 159.1, 134.3, 133.4, 132.9, 131.8, 131.3, 128.5, 114.1, 64.0, 55.4, 51.6, 28.7, 23.2. **HRMS:** Calculated for $C_{21}H_{25}ClN_2O_3$: 388.1554, Found: 388.1543. **I.R.** (thin film): ν 3427, 3043, 2968, 2936, 2872, 2840, 1661, 1597, 1511, 1248, 1169, 1093, 1017 cm^{-1}.

2-(N-allylacetamido)-N-(tert-butyl)-2-(4-chlorophenyl)acetamide (1m)

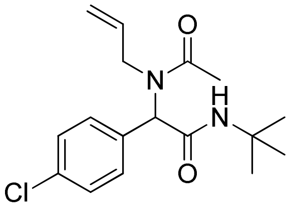

1m

This compound was synthesized according to the general procedure **I**, using 4-chlorobenzaldehyde (141 mg, 1.0 mmol), allylamine (0.075 ml, 1.0mmol), acetic acid (0.06 ml, 1.0 mmol) and tert-butyl isocyanide (0.11 ml, 1.0mmol). Purification by flash chromatography using (Et_2O : PE = 70 : 30) gave the desired product in 70% isolated yield (226 mg, 0.7 mmol) as white solid. **m.p. =** 140-142 °C. **R$_f$:** (Et_2O : PE = 7 : 3) = 0.43. **^{1}H-NMR** ($CDCl_3$, 400 MHz): δ (ppm) 7.36 (br s, 4H), 6.05 (s, 1H), 5.81 (br s, 1H), 5.50-5.41 (m, 1H), 5.02-4.96 (m, 2H), 4.02

(2dd, ABX system, J_{ab} = 17.8 , J_{ax} = 5.2, J_{bx} = 4.8 Hz, 2H), 2.18 (s, 3H), 1.38 (s, 9H). **^{13}C-NMR** ($CDCl_3$, 100.6 MHz): δ (ppm) 172.2, 168.7, 134.4, 134.3, 134.1, 130.9, 128.9, 116.6, 60.6, 51.7, 49.2, 28.6, 22.2. **HRMS:** Calculated for $C_{17}H_{23}ClN_2O_3$: 322.1448, Found: 322.1456. **I.R. (thin film):** ν 3426, 3086, 2970, 2935, 2873, 1655, 1598, 1515, 1409, 1254, 1193, 1092 cm^{-1}.

2-(N-allylacetamido)-2-(4-chlorophenyl)-N-cyclohexylacetamide (1n)

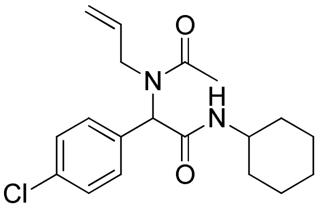

1n

This compound was synthesized according to the general procedure **I**, using 4-chlorobenzaldehyde (141 mg, 1.0 mmol), allylamine (0.075 ml, 1.0 mmol), acetic acid (0.06 ml, 1.0 mmol) and cyclohexylisocyanide (109 mg, 1.0 mmol). Purification by flash chromatography using gradient (Et_2O : DCM = 7 : 93 – 30 : 70) gave the desired product in 97% isolated yield (340 mg, 0.97 mmol) as white solid. **m.p. =**174-176°C. **R_f** (EtOAc : PE = 4 : 6) = 0.23. **^{1}H-NMR** ($CDCl_3$, 400 MHz): δ (ppm) 7.25 (br s, 4H), 5.96 (s, 1H), 5.82 (br s, 1H), 5.39 (ddt, J = 17.6, 10.3, 5.3 Hz, 1H), 4.94-4.90 (m, 2H), 3.96-3.85 (m, 2H), 3.76-3.66 (m, 1H), 2.08 (s, 3H), 1.84-1.81 (m, 2H), 1.63-1.50 (m, 3H), 1.32-1.22 (m, 2H), 1.11- 0.97 (m, 3H). **^{13}C-NMR** ($CDCl_3$, 100.6 MHz): δ (ppm) 172.3, 168.3, 134.4, 134.1, 133.9, 130.9, 128.9, 116.7, 60.4, 49.4, 48.6, 32.8, 25.5, 24.8, 24.8, 22.2. **HRMS:** Calculated for $C_{19}H_{25}ClN_2O_2$: 348.1605, Found: 348.1614. **I.R.** (thin film): ν 3424, 3086, 2936, 2857, 1711, 1676, 1640, 1513, 1409, 1258 and 1094 cm^{-1}.

2-(N-allylacetamido)-N-(tert-butyl)-2-(pyridin-2-yl)acetamide (1o)

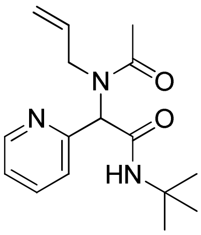

1o

This compound was synthesized according to the general procedure **I**, using pyridine-2-carboxaldehyde (0.1 ml, 1.0 mmol), allylamine (0.075 ml, 1.0 mmol), acetic acid (0.06 ml, 1.0 mmol) and tert-butyl isocyanide (0.11 ml, 1.0 mmol). Purification by flash chromatography using pure EtOAc gave the desired product in 97% isolated yield (281 mg, 0.97 mmol) as white solid. **m.p.** =79-81°C. **R$_f$:** (pure EtOAc) = 0.3. **^{1}H-NMR** ($CDCl_3$, 400 MHz): δ (ppm) 8.48 (d, *J* = 4.2 Hz, 1H), 7.61 (t, *J* = 7.7 Hz, 1H), 7.35 (d, *J* = 7.9 Hz, 1H), 7.16 (dd, *J* = 7.2, 5.1 Hz, 1H), 6.94 (br s, 1H), 5.95 (s, 1H), 5.50 (ddd, *J* = 22.4, 10.5, 5.4 Hz, 1H), 4.92 (2d, $J_{trans-1,3}$ = 12.4, $J_{cis-1,3}$ = 5.2 Hz, 2H), 4.04 (d, *J* = 5.3 Hz, 2H), 2.10 (s, 3H), 1.29 (s, 9H). **^{13}C-NMR** ($CDCl_3$, 100.6 MHz): δ (ppm) 172.1, 167.6, 156.3, 148.9, 137.0, 134.1, 124.3, 122.8, 116.6, 63.1, 51.5, 50.5, 28.7, 22.1. **HRMS:** Calculated for $C_{16}H_{23}N_3O_2$: 289.1790, Found: 289.1783. **I.R**. (thin film): ν 3427, 3062, 2970, 2933, 2874, 1649, 1592, 1469, 1259, 1224 and 1053 cm^{-1}.

N-allyl-N-(2-(tert-butylamino)-1-(4-chlorophenyl)-2-oxoethyl)-4-methoxybenzamide (1p)

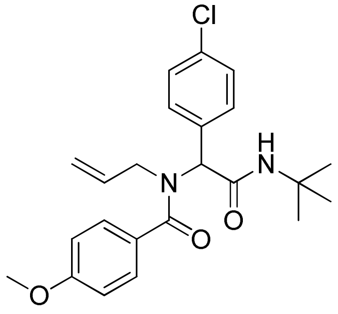

1p

This compound was synthesized according to the general procedure **I**, using 4-chlorobenzaldehyde (141 mg, 1.0 mmol), allylamine (0.075 ml, 1.0 mmol), 4-methoxybenzoic acid (152 mg, 1.0 mmol) and tert-butyl isocyanide (0.11 ml, 1.0 mmol). Purification by flash chromatography using (Et_2O : DCM = 5 : 95) gave the desired product in 70% isolated yield (292 mg, 0.7 mmol) as white solid. **m.p.** =138-140°C. **R$_f$:** (EtOAc : PE = 4 : 6) = 0.43. **^{1}H-NMR** ($CDCl_3$, 400 MHz): δ (ppm) 7.39 (d, *J* = 8.4 Hz, 2H), 7.34-7.25 (m, 4H), 6.82 (d, *J* = 8.5 Hz, 2H), 5.93 (br s, 1H), 5.50 (br s, 1H), 5.43 (s, 1H), 4.93-4.87 (m, 2H), 3.89 (2dd, ABX system, J_{ab} = 16.8, J_{ax} = J_{bx} = 5.6 Hz, 2H), 3.75 (s, 3H), 1.29 (s, 9H). **^{13}C-NMR** ($CDCl_3$, 100.6 MHz): δ (ppm) 172.9, 168.4, 161.1, 134.3, 134.3, 133.8, 130.7, 128.9, 128.0, 117.6, 113.8, 55.4, 51.7, 28.7. **HRMS:** Calculated for $C_{23}H_{27}ClN_2O_3$:414.1710, Found: 414.1701. **I.R.** (thin film): ν 3426, 3084, 2968, 2936, 2841, 1681, 1621, 1576, 1513, 1252, 1175 and 1033 cm^{-1}.

N-allyl-N-(2-(tert-butylamino)-1-(4-chlorophenyl)-2-oxoethyl)-3-(4-methoxyphenyl)propanamide (1q)

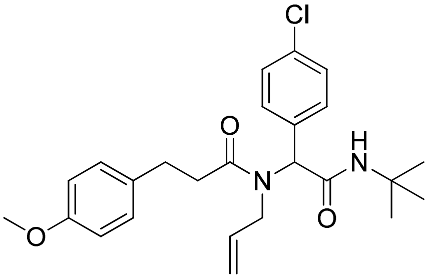

1q

This compound was synthesized according to the general procedure **I**, using 4-chlorobenzaldehyde (141 mg, 1.0 mmol), allylamine (0.075 ml, 1.0 mmol), (4-methoxybenzyl)acetic acid (180 mg, 1.0 mmol) and tert-butyl isocyanide(0.11 ml, 1.0 mmol). Purification by flash chromatography using (Et_2O : DCM = 7 : 93) gave the desired product in 68% isolated yield (302 mg, 0.68mmol) as white solid. **m.p. =**121-123°C. **R_f:** (Et_2O : PE = 7 : 3) = 0.47. **^{1}H-NMR** ($CDCl_3$, 400 MHz): δ (ppm) 7.23 (d, J = 8.5 Hz, 2H), 7.18 (d, J = 8.6 Hz, 2H), 7.03 (d, J = 8.5 Hz, 2H), 6.74 (d, J = 8.6 Hz, 2H), 5.89 (s, 1H), 5.68 (br s, 1H), 5.33 (ddd, J = 22.1, 10.3, 5.1 Hz, 1H), 4.88-4.80 (m, 2H), 3.84 (dd, ABX system, J_{ab} = 17.6, J_{ax} = 4.8, J_{bx} = 5.2 Hz, 2H), 3.71 (s, 3H), 2.85 (td, ABX_2 system, J = 6.8, 3.2 Hz, 2H), 2.58 (td, ABX_2 system, J = 7.2, 2.4 Hz, 2H), 1.26 (s, 9H). **^{13}C-NMR** ($CDCl_3$, 100.6 MHz): δ (ppm) 174.0, 168.7, 158.0, 134.3, 134.2, 133.1, 130.9, 129.4, 128.9, 116.6, 113.9, 61.0, 55.3, 51.7, 48.6, 35.7, 30.5, 28.6. **HRMS:** Calculated for $C_{25}H_{31}ClN_2O_3$: 422.2023, Found: 442.2023. **I.R.** (thin film): ν 3426, 3086, 3069, 2968, 2936, 2912, 2838, 1683, 1641, 1513, 1411, 1367, 1301, 1247, 1179 and 1094 cm^{-1}.

N-(2-(tert-butylamino)-1-(4-chlorophenyl)-2-oxoethyl)-N-propylcinnamamide (1r)

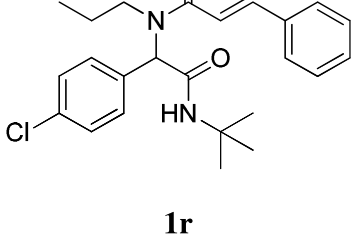

1r

This compound was synthesized according to the general procedure **I**, using 4-chlorobenzaldehyde (141 mg, 1.0 mmol), propylamine (0.08 ml, 1.0mmol), cinnamic acid (148 mg, 1.0 mmol) and tert-butyl isocyanide (0.11 ml, 1.0 mmol) in CF_3CH_2OH at 60 °C. Purification by flash chromatography using (Et_2O : DCM = 4 : 96) gave the desired product in 85% isolated yield (349 mg, 0.85 mmol) as white solid. **m.p. =** 194-196 °C. **R$_f$:** (Et_2O : DCM = 0.4 : 9.6) = 0.28. **^{1}H-NMR** ($CDCl_3$, 400 MHz): δ (ppm) 7.70 (d, *J* = 15.4 Hz, 1H), 7.45 (d, *J* = 5.4 Hz, 2H), 7.32-7.26 (m, 7H), 6.78 (d, *J* = 15.2 Hz, 1H), 5.97 (br s, 1H), 5.90 (s, 1H), 3.43-3.30 (m, 2H), 1.54-1.45 (m, 1H), 1.29 (s, 9H), 1.14-1.02 (m, 1H), 0.70 (t, *J* = 7.4 Hz, 3H). **^{13}C-NMR** ($CDCl_3$, 100.6 MHz): δ (ppm) 168.8, 167.3, 143.8, 135.1, 134.5, 134.2, 130.5, 129.9, 128.9, 128.9, 128.0, 117.4, 62.0, 51.7, 48.5, 28.7, 24.0, 11.4. **HRMS:** Calculated for [M-CONHCMe$_3$] $C_{19}H_{19}ClNO$: 312.1161, Found: 312.1156. **I.R.** (thin film): ν 3426, 2969, 2933, 2877, 1684, 1646, 1598, 1513, 1493, 1453, 1418, 1367, 1220, 1128, 1094, 1016 and 977 cm^{-1}.

N-(tert-butyl)-2-(4-chlorophenyl)-2-(N-(prop-2-yn-1-yl)acetamido)acetamide (1s)

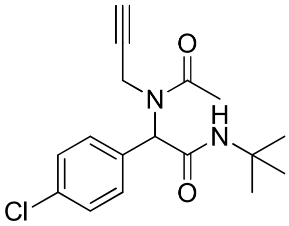

1s

This compound was synthesized according to the general procedure **I**, using 4-chlorobenzaldehyde (141 mg, 1.0 mmol), propargylamine (0.07 ml, 1.0 mmol), acetic acid (0.06 ml, 1.0 mmol) and tert-butyl isocyanide (0.11 ml, 1.0 mmol). Purification by flash chromatography using (Et_2O : PE = 70 : 30) gave the desired product in 90 % isolated yield (289 mg, 0.9 mmol) as white solid. **m.p. =** 157-159 °C. **R$_f$:** (Et_2O : PE = 7 : 3) = 0.31. **^{1}H-NMR** ($CDCl_3$, 400 MHz): δ (ppm) 7.26 (s, 4H), 6.06 (s, 1H), 5.72 (br s, 1H), 4.10 – 3.96 (m, 2H), 2.23 (s, 3H), 1.96 (s, 1H), 1.29 (s, 9H). **^{13}C-NMR** ($CDCl_3$, 100.6 MHz): δ (ppm) 171.9, 168.5, 134.5, 133.6, 130.8, 129.0, 79.3, 71.9, 59.7, 51.9, 35.9, 28.6, 22.1. **HRMS:** Calculated for $C_{17}H_{21}ClN_2O_2$: 320.1292, Found: 320.1279. **I.R.** (thin film): ν 3424, 3004, 2969, 1681, 1651, 1517, 1455, 1367, 1252, 1193 and 1016 cm^{-1}.

General procedure II for Tsuji-Trost reaction

A solution of Ugi adduct (1.0 equiv) and sodium hydride (2.5 equiv) in DMSO (0.5 M) was stirred at room temp under Argon for 5 min, followed by the addition of allyl acetate derivative (1.5 equiv), $Pd(dba)_2$ (0.05 equiv) and PPh_3 (0.1 equiv). The resulting solution was stirred at room temp for 0.5-2 hrs depending on the reaction progress checked by TLC. After the completion of the reaction, it was diluted with CH_2Cl_2 and extracted with water. The organic phase was dried over $MgSO_4$ and the solvent was removed under reduced pressure. The crude residue was purified by flash chromatography on silica gel to give a pure product.

N-(tert-butyl)-2-(4-chlorophenyl)-2-(N-propylacetamido)pent-4-enamide (3a)

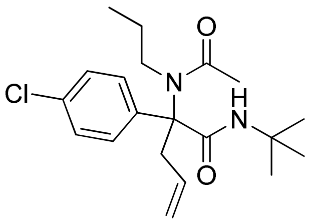

3a

This compound was synthesized according to the general procedure **II**, using Ugi product **1a** (163 mg, 0.5 mmol), sodium hydride (50 mg, 1.25 mmol), allylacetate (0.08 ml, 0.75 mmol), $Pd(dba)_2$ (14 mg, 0.025 mmol) and PPh_3 (13 mg, 0.05 mmol). Purification by flash chromatography using (Et_2O : DCM = 10 : 90) gave the desired product in 96% isolated yield (175 mg, 0.48 mmol) as yellow oil. **R_f:** (Et_2O : PE = 6 : 4) = 0.43. **^{1}H-NMR** ($CDCl_3$, 400 MHz): δ (ppm) 7.25 (br s, 1H), 7.17 (d, J = 8.8 Hz, 2H), 7.04 (d, J = 8.0 Hz, 2H), 5.48 (ddt, J = 10.3, 7.2, 6.5 Hz, 1H), 4.85 (2dd, $J_{1,2}$ = 1.2, $J_{cis-1,3}$ = 9.2, $J_{trans-1,3}$ = 16.8 Hz, 2H), 3.38-3.30 (m, 2H), 2.95 (dd, ABX system, J_{ab} = 14.6, J_{ax} = 6.2 Hz, 1H), 2.78 (dd, ABX system, J_{ba} = 14.6, J_{bx} = 7.4 Hz, 1H), 2.12 (s, 3H), 1.76-1.67 (m, 1H), 1.52-1.43 (m, 1H), 1.24 (s, 9H), 0.79 (t, J = 7.4 Hz, 3H). **^{13}C-NMR** ($CDCl_3$, 100.6 MHz): δ (ppm) 173.0, 171.2, 139.2, 133.5, 132.5, 127.9, 127.8, 118.9, 71.2, 51.2, 48.8, 42.7, 28.4, 24.3, 23.5, 11.4. **HRMS:** Calculated for $C_{20}H_{29}ClN_2O_2$: 364.1918, Found: 364.1911. **I.R.** (thin film): ν 3428, 3080, 2969, 2934, 2877, 1670, 1631, 1547, 1404, 1367, 1097 and 1014 cm^{-1}.

N-(tert-butyl)-2-(4-nitrophenyl)-2-(N-propylacetamido)pent-4-enamide (3b)

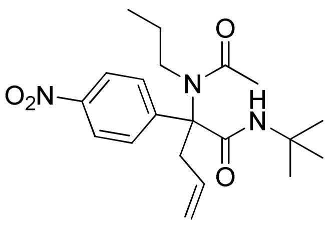

3b

This compound was synthesized according to the general procedure **II**, using Ugi product **1b** (168 mg, 0.5 mmol), sodium hydride (50 mg, 1.25 mmol), allylacetate (0.08 ml, 0.75 mmol), $Pd(dba)_2$ (14 mg, 0.025 mmol) and PPh_3 (13 mg, 0.05 mmol). Purification by flash chromatography using (EtOAc : PE = 20 : 80) gave the desired product in 75% isolated yield (140 mg, 0.37 mmol) as yellow oil. **R_f:** (Et_2O : PE = 7 : 3) = 0.43. **^{1}H-NMR** ($CDCl_3$, 400 MHz): δ (ppm) 8.05 (d, J = 8.8 Hz, 2H), 7.63 (br s, 1H), 7.15 (d, J = 8.4 Hz, 2H), 5.45 (ddt, J = 16.8, 10.2, 6.9 Hz, 1H), 4.82 (d, J = 10.0 Hz, 1H), 4.68 (d, J = 16.8 Hz, 1H), 3.49-3.43 (m, 2H), 2.93 (dd, ABX system, J_{ab} = 14.4, J_{ax} = 6.2 Hz, 1H), 2.68 (dd, ABX system, J_{ba} = 14.4, J_{bx} = 7.7 Hz, 1H), 2.16 (s, 3H), 1.85-1.76 (m, 1H), 1.55-1.46 (m, 1H), 1.26 (s, 9H), 0.87 (t, J = 7.4 Hz, 3H). **^{13}C-NMR** ($CDCl_3$, 100.6 MHz): δ (ppm) 173.6, 170.7, 148.8, 146.2, 132.4, 126.6, 122.8, 119.7, 71.3, 51.3, 48.8, 42.9, 28.4, 24.4, 23.4, 11.5. **HRMS:** Calculated for $C_{20}H_{29}N_3O_4$: 375.2158, Found: 375.2173. **I.R.** (thin film): ν 3288, 3082, 2968, 2934, 2877, 1673, 1630, 1605, 1520, 1399, 1350, 1244 cm^{-1}.

N-(tert-butyl)-2-(4-methoxyphenyl)-2-(N-propylacetamido)pent-4-enamide (3c)

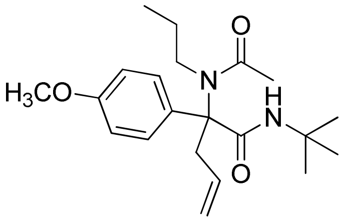

3c

This compound was synthesized according to the general procedure **II**, using Ugi product **1c** (160 mg, 0.5 mmol), sodium hydride (50 mg, 1.25 mmol), allylacetate (0.08 ml, 0.75 mmol), $Pd(dba)_2$ (14 mg, 0.025 mmol) and PPh_3 (13 mg, 0.05 mmol). Purification by flash chromatography using

(EtOAc : PE = 40 : 60) gave the desired product in 70% isolated yield (127 mg, 0.35 mmol) as yellow oil. **R_f:** (Et_2O : PE = 7 : 3) = 0.3. **^{1}H-NMR** ($CDCl_3$, 400 MHz): δ (ppm) 7.16 (d, J = 8.4 Hz, 2H), 6.76 (d, J = 8.8 Hz, 3H), 5.57 (ddt, J = 17.0, 10.2, 6.8 Hz, 1H), 4.95 (2d, $J_{cis\text{-}1,3}$ = 10.4, $J_{trans\text{-}1,3}$ = 17.2 Hz, 2H), 3.73 (s, 3H), 3.19 (dd, J = 10.1, 5.6 Hz, 2H), 3.10 (dd, ABX system, J_{ab} = 14.8, J_{ax} = 6.3 Hz, 1H), 2.92 (dd, ABX system, J_{ba} = 14.8, J_{bx} = 7.0 Hz, 1H), 2.10 (s, 3H), 1.64-1.57 (m, 1H), 1.51-1.40 (m, 1H), 1.24 (s, 9H), 0.71 (t, J = 7.3 Hz, 3H). **^{13}C-NMR** ($CDCl_3$, 100.6 MHz): δ (ppm) 172.5, 171.6, 158.5, 134.6, 131.9, 128.5, 118.5, 113.3, 71.2, 55.2, 51.2, 48.9, 42.0, 28.5, 24.1, 23.7, 11.4. **HRMS:** Calculated for $C_{21}H_{32}N_2O_3$: 360.2413, Found: 360.2420. **I.R.** (thin film): ν 3410, 3079, 2968, 2935, 2876, 2839, 1669, 1637, 1611, 1512, 1402, 1366, 1250, 1183, 1036 cm^{-1}.

2-(4-chlorophenyl)-N-cyclohexyl-2-(N-propylacetamido)pent-4-enamide (3d)

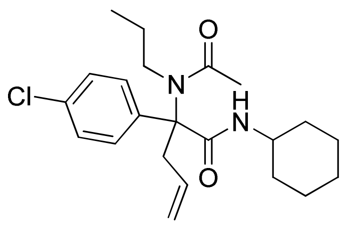

3d

This compound was synthesized according to the general procedure **II**, using Ugi product **1d** (175 mg, 0.5 mmol), sodium hydride (50 mg, 1.25 mmol), allylacetate (0.08 ml, 0.75 mmol), $Pd(dba)_2$ (14 mg, 0.025 mmol) and PPh_3 (13 mg, 0.05 mmol). Purification by flash chromatography using (Et_2O : DCM = 15 : 85) gave the desired product in 81% isolated yield (159 mg, 0.41 mmol) as yellow oil. **R_f:** (Et_2O : PE = 7 : 3) = 0.33. **^{1}H-NMR** ($CDCl_3$, 400 MHz): δ (ppm) 7.17 (d, J = 8.7 Hz, 2H), 7.06 (d, J = 8.2 Hz, 2H), 5.48 (ddt, J = 10.5, 7.2, 6.5 Hz, 1H), 4.91 – 4.82 (m, 2H), 3.77-3.68 (m, 1H), 3.39-3.29 (m, 2H), 3.02 (dd, ABX system, J_{ab} = 14.5, J_{ax} = 6.3 Hz, 1H), 2.79 (dd, ABX system, J_{ba} = 14.5, J_{bx} = 7.4 Hz, 1H), 2.11 (s, 3H), 1.85 – 1.65 (m, 3H), 1.65-1.45 (m, 4H), 1.32-1.22 (m, 2H), 1.15 – 0.97 (m, 3H), 0.78 (t, J = 7.4 Hz, 3H). **^{13}C-NMR** ($CDCl_3$, 100.6 MHz): δ (ppm) 173.1, 171.3, 139.0, 133.4, 132.6, 128.0, 127.9, 119.0, 70.8, 48.8, 48.3, 42.6, 32.5, 32.5, 25.8, 24.6, 24.3, 23.5, 11.4. **HRMS:** Calculated for $C_{22}H_{31}ClN_2O_2$: 390.2074, Found: 390.2088. **I.R.** (thin film): ν 3428, 3081, 2934, 2877, 2857, 1650, 1538, 1352, 1245, 1153, 1098, 1014 cm^{-1}.

2-(4-chlorophenyl)-N-(4-methoxybenzyl)-2-(N-propylacetamido)pent-4-enamide (3e)

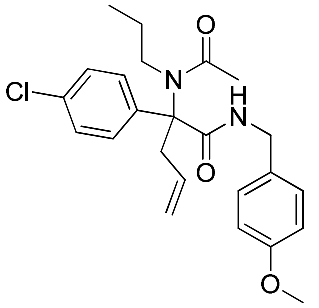

3e

This compound was synthesized according to the general procedure **II**, using Ugi product **1e** (194 mg, 0.5 mmol), sodium hydride (50 mg, 1.25 mmol), allylacetate (0.08 ml, 0.75 mmol), $Pd(dba)_2$ (14 mg, 0.025 mmol) and PPh_3 (13 mg, 0.05 mmol). Purification by flash chromatography using (Et_2O : PE = 70 : 30) gave the desired product in 55% isolated yield (108 mg, 0.276 mmol) as yellow oil. **R_f:** (Et_2O : PE = 8 : 2) = 0.35. **^{1}H-NMR** ($CDCl_3$, 400 MHz): δ (ppm) 7.39 (br s, 1H), 7.15 (d, J = 8.8 Hz, 2H), 7.09 (d, J = 8.7 Hz, 2H), 7.05 (d, J = 8.6 Hz, 2H), 6.76 (d, J = 8.7 Hz, 2H), 5.48 (dddd, J = 16.9, 10.9, 7.4, 6.3 Hz, 1H), 4.92 – 4.83 (m, 2H), 4.29 (dd, ABX system, J_{ab} = 5.7, J_{ax} = J_{bx} = 1.2 Hz, 2H), 3.72 (s, 3H), 3.33-3.19 (m, 2H), 3.04 (dd, ABX system, J_{ab} = 14.6, J_{ax} = 6.2 Hz, 1H), 2.84 (dd, ABX system, J_{ba} = 14.5, J_{bx} = 7.4 Hz, 1H), 2.09 (s, 3H), 1.64-1.55 (m, 1H), 1.41-1.31 (m, 1H), 0.72 (t, J = 7.4 Hz, 3H). **^{13}C-NMR** ($CDCl_3$, 100.6 MHz): δ (ppm) 173.1, 172.1, 158.8, 138.6, 133.3, 132.8, 130.4, 129.2, 128.2, 128.0, 119.1, 113.9, 70.8, 55.3, 48.9, 43.4, 42.4, 24.1, 23.6, 11.3. **HRMS:** Calculated for $C_{24}H_{29}ClN_2O_3$: 428.1867, Found: 428.1859. **I.R.** (thin film): ν 3438, 3081, 2963, 2935, 2877, 2839, 1667, 1636, 1514, 1404, 1248, 1176, 1097, 1035, 1015 cm^{-1}.

N-(tert-butyl)-2-(furan-2-yl)-2-(N-propylacetamido)pent-4-enamide (3f)

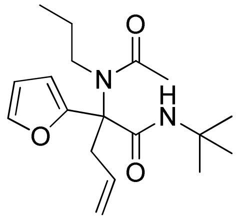

3f

This compound was synthesized according to the general procedure **II**, using Ugi product **1f** (140 mg, 0.5 mmol), sodium hydride (50 mg, 1.25 mmol), allylacetate (0.08 ml, 0.75 mmol), $Pd(dba)_2$ (14 mg, 0.025 mmol) and PPh_3 (13 mg, 0.05 mmol). Purification by flash chromatography using (Et_2O : DCM = 10 : 90) gave the desired product in 77% isolated yield (123 mg, 0.38 mmol) as yellow oil. **R_f:** (Et_2O : PE = 7 : 3) = 0.33. **^{1}H-NMR** ($CDCl_3$, 400 MHz): δ (ppm) 7.37 (dd, J = 1.8, 0.7 Hz, 1H), 6.43 (dd, J = 3.3, 0.6 Hz, 1H), 6.31 (dd, J = 3.3, 1.8 Hz, 1H), 5.97 (br s, 1H), 5.74-5.64 (ddt, J = 17.2, 10.1, 7.1 Hz, 1H), 5.12 (dd, $J_{trans-1,3}$ = 17.1, $J_{1,2}$ = 1.8 Hz, 1H), 5.01 (dd, $J_{cis-1,3}$ = 10.0, $J_{1,2}$ = 1.6 Hz, 1H), 3.14 (dd, ABX system, J_{ab} = 14.0, J_{ax} = 6.8, J_{bx} = 7.6 Hz, 2H), 3.02-2.94 (m, 1H), 2.81 (ddd, ABXY system, J = 16.4, 11.2, 5.6 Hz, 1H), 2.05 (s, 3H), 1.55-1.44 (m, 2H), 1.26 (s, 9H), 0.64 (t, J = 7.4 Hz, 3H). **^{13}C-NMR** ($CDCl_3$, 100.6 MHz): δ (ppm) 171.0, 168.8, 152.3, 142.4, 134.8, 118.9, 111.5, 110.8, 68.2, 51.4, 49.6, 38.6, 28.5, 23.8, 23.1, 11.4. **HRMS:** Calculated for $C_{18}H_{28}N_2O_3$: 320.2100, Found: 320.2090. **I.R.** (thin film): ν 3415, 3081, 2971, 2933, 2876, 1679, 1643, 1515, 1407, 1366, 1250, 1222, 1144, 1022 cm^{-1}.

N-(tert-butyl)-2-(N-propylacetamido)-2-(pyridin-2-yl)pent-4-enamide (3g)

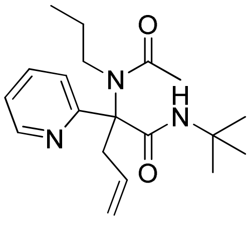

3g

This compound was synthesized according to the general procedure **II**, using Ugi product **1g** (146 mg, 0.5 mmol), sodium hydride (50 mg, 1.25 mmol), allylacetate (0.08 ml, 0.75 mmol), $Pd(dba)_2$ (14 mg, 0.025 mmol) and PPh_3 (13 mg, 0.05 mmol). Purification by flash chromatography using (Et_2O : DCM = 20 : 80) gave the desired product in 92% isolated yield (152mg, 0.46 mmol) as yellow oil. **R_f:** (Et_2O : PE = 9 : 1) = 0.26. **^{1}H-NMR** ($CDCl_3$, 400 MHz): δ (ppm) 9.65 (br s, 1H), 8.39 (d, J = 4.4 Hz, 1H), 7.55 (t, J = 7.5 Hz, 1H), 7.17 (d, J = 8.0 Hz, 1H), 7.05 (t, J = 5.6 Hz, 1H), 5.47-5.37 (ddt, J = 16.8, 10.8, 7.2 Hz, 1H), 4.89 – 4.79 (m, 2H), 3.55 – 3.40 (m, 2H), 2.96 (dd, ABX system, J_{ab} = 14.0, J_{ax} = 6.5 Hz, 1H), 2.70 (dd, ABX system, J_{ba} = 13.9, J_{bx} = 7.2 Hz, 1H), 2.09 (s, 3H), 1.94-1.85 (m, 1H), 1.77-1.68 (m, 1H), 1.30 (s, 9H), 0.86 (t, J = 7.4 Hz, 3H). **^{13}C-NMR** ($CDCl_3$, 100.6 MHz): δ (ppm) 171.9, 170.0, 162.6, 146.5, 136.9, 133.0, 121.3, 120.7, 118.2, 71.0, 50.9, 48.7, 43.6, 28.7, 24.7, 23.1, 11.2. **HRMS:** Calculated for [M-$CONHCMe_3$]

$C_{14}H_{19}N_2O$: 231.1497, Found: 231.1494. **I.R.** (thin film): ν 3401, 3061, 2970, 2933, 2876, 1667, 1651, 1574, 1431, 1405, 1365, 1247, 1224 and 1008 cm^{-1}.

N-(tert-butyl)-2-(4-chlorophenyl)-2-(N-(4-methoxybenzyl)acetamido)pent-4-enamide (3k)

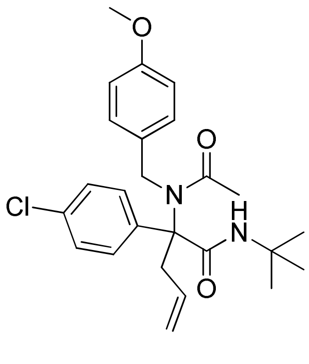

3k

This compound was synthesized according to the general procedure **II**, using Ugi product **1k** (202 mg, 0.5 mmol), sodium hydride (50 mg, 1.25 mmol), allylacetate (0.08 ml, 0.75 mmol), $Pd(dba)_2$ (14 mg, 0.025 mmol) and PPh_3 (13 mg, 0.05 mmol). Purification by flash chromatography using (Et_2O : DCM = 10 : 90) gave the desired product in 56% isolated yield (123 mg, 0.28 mmol) as yellow oil. **R_f:** (Et_2O : DCM = 1 : 9) = 0.32. **^{1}H-NMR** (CD_3OD, 400 MHz): δ (ppm) 7.44 (d, J = 8.8 Hz, 2H), 7.25 (d, J = 8.8 Hz, 4H), 6.91 (d, J = 8.7 Hz, 2H), 6.53 (br s, 1H), 5.82-5.69 (m, 1H), 5.06 (dd, J = 11.3, 6.4 Hz, 2H), 2H), 4.61-4.41 (m, 2H), 3.80 (s, 3H), 2.22 (2 dd, ABX system, J_{ab} = 14.4, J_{ax} = 6.4 Hz, 1H), 2.15 (s, 3H), 1.31 (s, 9H). **^{13}C-NMR** (CD_3OD, 100.6 MHz): δ (ppm) 175.7, 172.2, 160.4, 138.5, 135.1, 134.9, 131.2, 131.0, 129.1, 128.5, 119.9, 115.2, 73.3, 55.8, 52.7, 51.4, 41.4, 28.8, 24.0. **HRMS:** Calculated for [M-CONHCMe$_3$] $C_{20}H_{21}ClNO_2$: 342.1261, Found: 342.1254. **I.R.** (thin film): ν 3429, 3080, 3000, 2967, 2935, 2911, 2839, 1672, 1614, 1513, 1393, 1248, 1176, 1098, 1035, 1014 cm^{-1}.

N-(tert-butyl)-2-(4-chlorophenyl)-2-(N-(4-methoxyphenyl)acetamido)pent-4-enamide (3l)

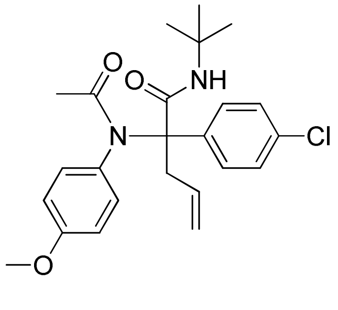

3l

This compound was synthesized according to the general procedure **II**, using Ugi product **1l** (151 mg, 0.5 mmol), sodium hydride (50 mg, 1.25 mmol), allylacetate (0.08 ml, 0.75 mmol), Pd(dba)$_2$ (14 mg, 0.025 mmol) and PPh$_3$ (13 mg, 0.05 mmol). Purification by flash chromatography using (Et$_2$O : DCM = 5 : 95) gave the desired product in 60% isolated yield (128 mg, 0.30 mmol) as yellow oil. **R$_f$:** (Et$_2$O : DCM = 5 : 95) = 0.43. **^{1}H-NMR** (CDCl$_3$, 400 MHz): δ (ppm) 7.37-7.31 (m, 4H), 7.27 (dd, *J* = 8.6, 2.6 Hz, 1H), 7.11 (dd, *J* = 8.7, 2.6 Hz, 1H), 7.02 (br s, 1H), 6.96 (dd, *J* = 8.7, 2.9 Hz, 1H), 6.89 (dd, *J* = 8.7, 2.9 Hz, 1H), 5.44 (ddt, *J* = 17.2, 10.4, 6.4 Hz, 1H), 4.80 (dd, $J_{cis\text{-}1,3}$ = 10.3, $J_{1,2}$ = 1.4 Hz, 1H), 4.69 (dd, $J_{trans\text{-}1,3}$ = 17.1, $J_{1,2}$ = 1.4, 1H), 3.87 (s, 3H), 2.80 (dd, ABX system, J_{ab} = 13.9, J_{ax} = 6.2 Hz, 1H), 2.25 (dd, ABX system, , J_{ba} = 13.9, J_{bx} = 7.6 Hz, 1H), 1.82 (s, 3H), 1.43 (s, 9H). **^{13}C-NMR** (CDCl$_3$, 100.6 MHz): δ (ppm) 172.3, 170.0, 159.5, 138.6, 133.5, 133.4, 132.8, 131.4, 131.2, 128.7, 128.1, 118.1, 114.6, 114.4, 72.2, 55.5, 51.5, 44.6, 28.6, 25.5. **HRMS:** Calculated for $C_{24}H_{29}ClN_2O_3$:428.1867, Found: 428.1885. **I.R.** (thin film): ν 3428, 3080, 3041, 2967, 2935, 2873, 2841, 1672, 1606, 1543, 1457, 1375, 1249, 1170, 1098, 1035, 1014 cm^{-1}.

2-(N-allylacetamido)-N-(tert-butyl)-2-(4-chlorophenyl)pent-4-enamide (3m)

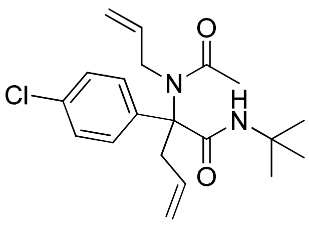

3m

This compound was synthesized according to the general procedure **II**, using Ugi product **1m** (161 mg, 0.5 mmol), sodium hydride (50 mg, 1.25 mmol), allylacetate (0.08 ml, 0.75 mmol), Pd(dba)$_2$ (14 mg, 0.025 mmol) and PPh$_3$ (13 mg, 0.05 mmol). Purification by flash chromatography using (Et$_2$O : DCM = 7 : 93) gave the desired product in 91% isolated yield (166 mg, 0.46mmol) as yellow oil. **R$_f$:** (Et$_2$O : DCM = 0.7 : 9.3) = 0.38. **^{1}H-NMR** (CDCl$_3$, 400 MHz): δ (ppm) 7.20 (br s, 4H), 6.48 (br s, 1H), 5.82-5.73 (m, 1H), 5.56-5.46 (ddt, *J* = 17.1, 10.2, 6.9 Hz, 1H), 5.29 (dd, $J_{trans\text{-}1,3}$ = 17.2, $J_{1,2}$ = 1.0 Hz, 1H), 5.18 (dd, $J_{cis\text{-}1,3}$ = 10.5, $J_{1,2}$ = 1.0 Hz, 1H), 5.01 – 4.91 (m, 2H), 3.94-3.83 (m, 2H), 3.15 (dd, ABX system, J_{ab} = 14.6, J_{ax} = 6.3 Hz, 1H), 2.90 (dd,

ABX system, J_{ab} = 14.6, J_{ax} = 7.3 Hz, 1H), 2.08 (s, 3H), 1.24 (s, 9H). **^{13}C-NMR** ($CDCl_3$, 100.6 MHz): δ (ppm) 173.3, 170.4, 138.2, 134.5, 133.8, 133.2, 128.8, 128.2, 119.2, 117.3, 71.8, 51.4, 49.4, 41.8, 28.5, 24.0. **HRMS:** Calculated for $C_{20}H_{27}ClN_2O_2$: 362.1761, Found: 362.1749. **I.R.** (thin film): ν 3428, 3083, 2969, 2932, 2874, 1671, 1595, 1543, 1493, 1392, 1243, 1223, 1098, 1014 cm^{-1}.

2-(N-allylacetamido)-2-(4-chlorophenyl)-N-cyclohexylpent-4-enamide (3n)

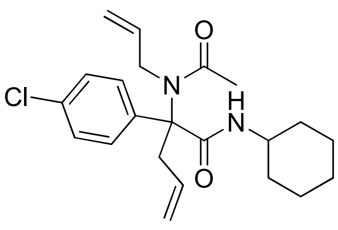

3n

This compound was synthesized according to the general procedure **II**, using Ugi product **1n** (175 mg, 0.5 mmol), sodium hydride (50 mg, 1.25 mmol), allylacetate (0.08 ml, 0.75 mmol), $Pd(dba)_2$ (14 mg, 0.025 mmol) and PPh_3 (13 mg, 0.05 mmol). Purification by flash chromatography using (Et_2O : DCM = 15 : 85) gave the desired product in 82% isolated yield (159 mg, 0.41 mmol) as yellow oil. **R_f:** (Et_2O : DCM = 1.5 : 8.5) = 0.45. **^{1}H-NMR** ($CDCl_3$, 400 MHz): δ (ppm) 7.22 (br s, 4H), 6.41 (d, J = 7.0 Hz, 1H), 5.81-5.72 (m, 1H), 5.56-5.46 (ddt, J = 16.8, 10.0, 6.8 Hz, 1H), 5.33 (dd, $J_{trans-1,3}$ = 17.3, $J_{1,2}$ = 1.0 Hz, 1H), 5.19 (dd, $J_{cis-1,3}$ = 10.5, $J_{1,2}$ = 0.8 Hz, 1H), 4.97 (2 dd, $J_{trans-1,3}$ = 16.8, $J_{cis-1,3}$ = 10.0, $J_{1,2}$ = 1.2 Hz, 2H), 3.87 (br s, 2H), 3.75-3.67 (m, 1H), 3.17 (dd, ABX system, J_{ab} = 14.5, J_{ax} = 6.2 Hz, 1H), 2.94 (dd, ABX system, J_{ba} = 14.6, J_{bx} = 7.4 Hz, 1H), 2.08 (s, 3H), 1.84-1.75 (m, 2H), 1.59-1.46 (m, 3H), 1.32-1.22 (m, 2H), 1.12-0.98 (m, 3H). **^{13}C-NMR** ($CDCl_3$, 100.6 MHz): δ (ppm) 173.3, 170.5, 137.9, 134.4, 133.7, 133.4, 128.9, 128.3, 119.2, 117.3, 71.3, 49.4, 48.5, 41.5, 32.7, 32.6, 25.6, 24.7, 24.6, 23.9. **HRMS:** Calculated for $C_{22}H_{29}ClN_2O_2$: 388.1918, Found: 388.1913. **I.R.** (thin film): ν 3427, 3083, 3015, 2983, 2934, 2857, 1655, 1603, 1533, 1451, 1395, 1253, 1098, 1015 cm^{-1}.

2-(N-allylacetamido)-N-(tert-butyl)-2-(pyridin-2-yl)pent-4-enamide (3o)

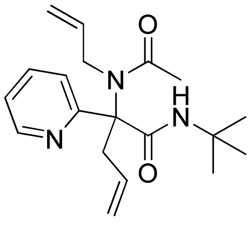

3o

This compound was synthesized according to the general procedure **II**, using Ugi product **1o** (145 mg, 0.5 mmol), sodium hydride (50 mg, 1.25 mmol), allylacetate (0.08 ml, 0.75 mmol), $Pd(dba)_2$ (14 mg, 0.025 mmol) and PPh_3 (13 mg, 0.05 mmol). Purification by flash chromatography using (Et_2O : DCM = 30 : 70) gave the desired product in 83% isolated yield (136 mg, 0.41 mmol) as yellow oil. **R_f:** (Et_2O : DCM = 3 : 7) = 0.39. **^{1}H-NMR** ($CDCl_3$, 400 MHz): δ (ppm) 9.41 (br s, 1H), 8.41 (d, J = 4.3 Hz, 1H), 7.57 (t, J = 7.5 Hz, 1H), 7.22 (d, J = 8.0 Hz, 1H), 7.08-7.06 (m, 1H), 6.12-6.03 (m, 1H), 5.51-5.35 (m, 2H), 5.21 (d, $J_{cis\text{-}1,3}$ = 10.4 Hz, 1H), 4.88-4.83 (m, 2H), 4.18 (br s, 2H), 2.99 (dd, ABX system, J_{ab} = 13.9, J_{ax} = 6.8 Hz, 1H), 2.79 (dd, ABX system, J_{ba} = 14.0, J_{bx} = 7.1 Hz, 1H), 2.07 (s, 3H), 1.28 (s, 9H). **^{13}C-**NMR ($CDCl_3$, 100.6 MHz): δ (ppm) 172.4, 169.7, 162.2, 146.8, 137.0, 136.0, 133.1, 121.5, 120.9, 118.5, 116.9, 71.6, 50.9, 49.5, 43.0, 28.6, 23.5. **HRMS:** Calculated for $C_{19}H_{27}N_3O_2$: 329.2103, Found: 329.2098. **I.R.** (thin film): ν 3221, 3061, 2975, 2930, 1659, 1592, 1533, 1432, 1395, 1365, 1246, 1224 and 992 cm^{-1}.

N-allyl-N-(1-(tert-butylamino)-2-(4-chlorophenyl)-1-oxopent-4-en-2-yl)-4-methoxybenzamide (3p)

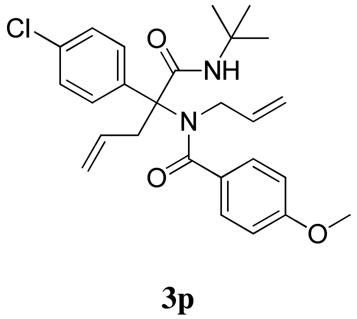

3p

This compound was synthesized according to the general procedure **II**, using Ugi product **1p** (208 mg, 0.5 mmol), sodium hydride (50 mg, 1.25 mmol), allylacetate (0.08 ml, 0.75 mmol), $Pd(dba)_2$ (14 mg, 0.025 mmol) and PPh_3 (13 mg, 0.05 mmol). Purification by flash

chromatography using (Et_2O : DCM = 2 : 98) gave the desired product in 64% isolated yield (146 mg, 0.32mmol) as yellow oil. **R_f:** (Et_2O : DCM = 0.2 : 9.8) = 0.46. **^{1}H-NMR** ($CDCl_3$, 400 MHz): δ (ppm) 8.10 (br s, 1H), 7.44 (d, J = 8.7 Hz, 2H), 7.14 (d, J = 8.7 Hz, 2H), 7.07 (d, J = 8.7 Hz, 2H), 6.86 (d, J = 8.7 Hz, 2H), 5.77-5.58 (m, 2H), 4.96 (dd, $J_{cis-1,3}$ = 10.2, $J_{1,2}$ = 4.5 Hz, 2H), 4.90 (d, $J_{trans-1,3}$ = 17.1 Hz, 2H), 4.27 (dd, ABX system, J_{ab} = 16.4, J_{ax} = 6.4 Hz, 1H), 4.05 (dd, ABX system, J_{ba} = 16.4, J_{bx} = 6.8 Hz, 1H), 3.78 (s, 3H), 3.00 (dd, ABX system, J_{ab} = 15.0, J_{ax} = 7.6 Hz, 1H), 2.86 (dd, ABX system, J_{ba}= 15.0, J_{bx} = 6.4 Hz, 1H), 1.29 (s, 9H). **^{13}C-NMR** ($CDCl_3$, 100.6 MHz): δ (ppm) 175.5, 171.5, 161.8, 139.1, 134.1, 133.0, 132.5, 130.1, 128.9, 127.8, 127.6, 119.2, 118.4, 113.9, 71.6, 55.5, 52.2, 51.1, 42.8, 28.4. **HRMS:** Calculated for [M-$CONHCMe_3$] $C_{21}H_{21}ClNO_2$: 354.1261, Found: 354.1262. **I.R.** (thin film): ν 3429, 3079, 2968, 2936, 2841, 1672, 1623, 1551, 1511, 1379, 1252, 1172, 1097, 1032, 1014 cm^{-1}.

2-(N-allyl-3-(4-methoxyphenyl)propanamido)-N-(tert-butyl)-2-(4-chlorophenyl)pent-4-enamide (3q)

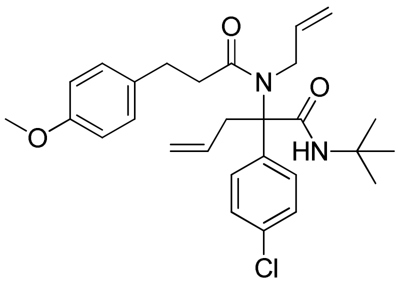

3q

This compound was synthesized according to the general procedure **II**, using Ugi product **1q** (222 mg, 0.5 mmol), sodium hydride (50 mg, 1.25 mmol), allylacetate (0.08 ml, 0.75 mmol), $Pd(dba)_2$ (14 mg, 0.025 mmol) and PPh_3 (13 mg, 0.05 mmol). Purification by flash chromatography using (EtOAc : DCM = 4 : 96) gave the desired product in 66% isolated yield (160 mg, 0.33 mmol) as yellow oil. **R_f:** (EtOAc : DCM = 0.4 : 9.6) = 0.35. **^{1}H-NMR** ($CDCl_3$, 400 MHz): δ (ppm) 7.17 (m, 2H), 7.09 (d, J = 8.0 Hz, 2H), 7.02 (d, J = 7.7 Hz, 2H), 6.75 (d, J = 8.4 Hz, 2H), 6.55 (br s, 1H), 5.77-5.70 (m, 1H), 5.51-5.41 (m, 1H), 5.18 (2d, J = 16.8, 10.8 Hz, 2H), 4.93-4.88 (m, 2H), 3.92-3.77 (m, 2H), 3.72 (s, 3H), 3.13 (dd, ABX system, J_{ab} = 14.5, J_{ax} = 5.9 Hz, 1H), 2.86-2.79 (m, 3H), 2.65-2.55 (m, 2H), 1.22 (s, 9H). **^{13}C-NMR** ($CDCl_3$, 100.6 MHz): δ (ppm) 175.0, 170.4, 158.0, 138.3, 134.8, 133.8, 133.1, 133.05, 129.5, 128.6, 128.2, 119.1, 117.3, 113.9, 72.0, 55.3, 51.4, 48.5, 41.9, 37.2, 30.4, 28.5. **HRMS:** Calculated for [M-$CONHCMe_3$]

$C_{23}H_{25}ClNO_2$: 382.1579, Found: 382.1579. **I.R.** (thin film): ν 3407, 3082, 2967, 2935, 2838, 1671, 1612, 1513, 1456, 1393, 1366, 1247, 1098, 1036 and 1014 cm^{-1}.

N-(tert-butyl)-2-(4-chlorophenyl)-2-(3-(4-methoxyphenyl)-N-propylacrylamido)pent-4-enamide (3r)

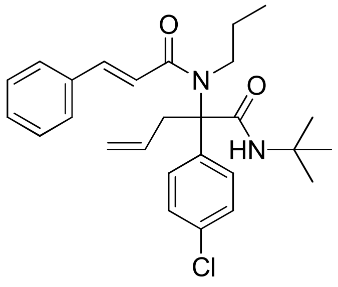

3r

This compound was synthesized according to the general procedure **II**, using Ugi product **1r** (206 mg, 0.5 mmol), sodium hydride (50 mg, 1.25 mmol), allylacetate (0.08 ml, 0.75 mmol), $Pd(dba)_2$ (14 mg, 0.025 mmol) and PPh_3 (13 mg, 0.05 mmol). Purification by flash chromatography using (pure DCM) gave the desired product in 49% isolated yield (110 mg, 0.24 mmol) as yellow oil. **R_f:** (DCM) = 0.38. **^{1}H-NMR** ($CDCl_3$, 400 MHz): δ (ppm) 7.64 (br s, 1H), 7.55 (d, J = 15.3 Hz, 1H), 7.42 (d, J = 3.7 Hz, 2H), 7.30 (dd, J = 5.0, 1.8 Hz, 3H), 7.17 (d, J = 8.7 Hz, 2H), 7.06 (d, J = 7.3 Hz, 2H), 6.82 (d, J = 14.7 Hz, 1H), 5.57 – 5.46 (m, 1H), 4.86 (2d, $J_{cis-1,3}$ = 10.4, $J_{trans-1,3}$ = 17.2 Hz, 2H), 3.64 – 3.52 (m, 1H), 3.47 (ddd, ABXY system, J_{ab} = 15.6, J_{ax} =10.6, J_{ay} =4.9 Hz, 1H), 2.97 (dd, ABX system, J_{ab} = 14.6, J_{ax} = 6.2 Hz, 1H), 2.84 (dd, ABX system, J_{ba} =14.6, J_{bx} =7.5 Hz, 1H), 1.82 – 1.70 (m, 1H), 1.67 – 1.55 (m, 1H), 1.26 (s, 9H), 0.84 (t, J = 7.4 Hz, 3H). **^{13}C-NMR** ($CDCl_3$, 100.6 MHz): δ (ppm) 171.4, 169.7, 143.9, 139.4, 134.9, 133.2, 132.5, 130.0, 128.9, 128.0, 127.9, 119.4, 119.0, 71.7, 51.2, 48.2, 43.0, 28.5, 24.3, 11.6. **HRMS:** Calculated for $C_{27}H_{33}ClN_2O_2$: 452.2231, Found: 452.2228. **I.R.** (thin film): ν 3410, 3076, 2969, 2922, 2843, 1668, 1615, 1512, 1455, 1396, 1367, 1253, 1089, 1032 and 1013 cm^{-1}.

1-Acetyl-N-(tert-butyl)-2-(4-chlorophenyl)-2,3-dihydro-1H-pyrrole-2-carboxamide (4)

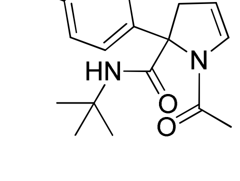

4

This compound was synthesized using Ugi product **1s** (161 mg, 0.5 mmol), sodium hydride (50 mg, 1.25 mmol) in DMSO (0.5 M) for 1 hour. Purification by flash chromatography using gradient eluent (Et_2O : DCM = 6 : 94 – 10 : 90) gave the desired product in 79 % isolated yield (127 mg, 0.4 mmol) as white solid. **m.p. =** 153-155 °C. **R_f:** (Et_2O : DCM) = 0.25. **^{1}H-NMR** ($CDCl_3$, 400 MHz): δ (ppm) 8.12 (br s, 1H), 7.21 (d, *J* = 7.9 Hz, 2H), 7.06 (d, *J* = 8.3 Hz, 2H), 5.90 (d, *J* = 6.3 Hz, 1H), 5.75 (d, *J* = 5.6 Hz, 1H), 4.43 (br s, 2H), 2.13 (s, 3H), 1.28 (s, 9H). **^{13}C-NMR** ($CDCl_3$, 100.6 MHz): δ (ppm) 170.5, 170.2, 138.1, 135.1, 133.3, 128.7, 127.1, 121.3, 81.0, 57.1, 51.3, 28.6, 24.0. **HRMS:** Calculated for $C_{17}H_{21}ClN_2O_2$: 320.1292, Found: 320.1926. **I.R.** (thin film): ν 3275, 3007, 2931, 1673, 1626, 1554, 1493, 1403, 1363, 1290, 1237, 1096, 1015 and 977 cm^{-1}.

General procedure III for alkylation reaction

A solution of Ugi adducts (1.0 equiv) and sodium hydride (2.5 equiv) in DMSO (0.5 M) was stirred at room temp under Argon for 5 min, followed by the addition of alkyl halide derivative (1.5 equiv). The resulting solution was stirred at room temp for 1-4 hrs depending on the reaction progress checked by TLC. After the completion of the reaction, it was diluted with CH_2Cl_2 and extracted with water. The organic phase was dried over $MgSO_4$ and the solvent was removed under reduced pressure. The crude residue was purified by flash chromatography on silica gel to give a pure product.

N-(tert-butyl)-2-(4-chlorophenyl)-2-(N-(prop-2-yn-1-yl)acetamido)pent-4-enamide (3s)

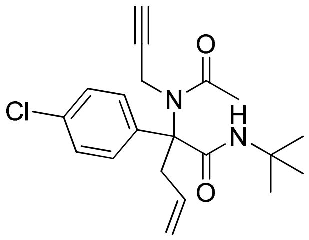

3s

This compound was synthesized according to the general procedure **III**, using Ugi product **1s** (160 mg, 0.5 mmol), sodium hydride (50 mg, 1.25 mmol), allyl bromide (0.06 ml, 0.75 mmol). Purification by flash chromatography using (Et_2O : DCM = 5 : 95) gave the desired product in 58 % isolated yield (105 mg, 0.29 mmol) as light yellow oil. **R_f:** (Et_2O : DCM = 0.5 : 9.5) = 0.19. **^{1}H-NMR** (DMSO-d_6, 400 MHz): δ (ppm) 7.50 (d, *J* = 8.7 Hz, 2H), 7.33 (d, *J* = 8.8 Hz, 2H), 6.44 (br s, 1H), 5.63 (ddt, *J* = 17.0, 10.3, 6.8 Hz, 1H), 5.05 – 4.96 (m, 2H), 4.26 (dd, *J* = 8.4, 2.2 Hz, 2H), 3.37 (t, *J* = 2.3 Hz, 1H), 3.1-3.05 (m, 2H), 2.15 (s, 3H), 1.26 (s, 9H). **^{13}C-NMR** (DMSO-d_6, 100.6 MHz): δ (ppm) δ 171.9, 169.7, 139.8, 134.1, 132.0, 123.0, 127.7, 118.8, 81.4, 76.0, 71.3, 51.1, 41.9, 36.6, 28.7, 23.9. **HRMS:** Calculated for [M-CONH*t*-Bu] $C_{15}H_{16}ClNO$: 260.0848, Found: 260.0844. **I.R.** (thin film): ν 3299, 3077, 2971, 2901, 1666, 1511, 1492, 1391, 1364, 1241, 1076, 1013, 920 and 733 cm^{-1}.

N-(tert-butyl)-2-(4-chlorophenyl)-2-(N-propylacetamido)pent-4-enamide (3a)

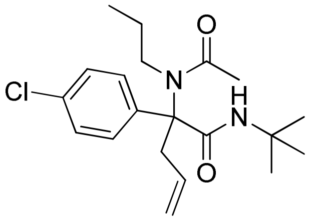

3a

This compound was synthesized according to the general procedure **III**, using Ugi product **1a** (163 mg, 0.5 mmol), sodium hydride (50 mg, 1.25 mmol), allyl bromide (0.06 ml, 0.75 mmol). Purification by flash chromatography using (Et_2O : DCM = 10 : 90) gave the desired product in 83% isolated yield (121 mg, 0.33 mmol) as yellow oil.

N-(tert-butyl)-2-(2-fluorophenyl)-2-(N-propylacetamido)pent-4-enamide (3i)

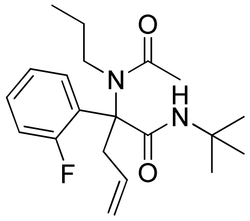

3i

This compound was synthesized according to the general procedure **III**, using Ugi product **1i** (154 mg, 0.5 mmol), sodium hydride (50 mg, 1.25 mmol), allyl bromide (0.06 ml, 0.75 mmol). Purification by flash chromatography using (Et_2O : DCM = 5 : 95) gave the desired product in 40 % isolated yield (70 mg, 0.2 mmol) as light yellow oil. **R_f:** (Et_2O : DCM = 0.5 : 9.5) = 0.38. **^{1}H-NMR** ($CDCl_3$, 400 MHz): δ (ppm) 7.93 (br s, 1H), 7.26 – 7.18 (m, 1H), 7.16 – 7.05 (m, 2H), 6.97 (dd, *J* = 12.7, 8.0 Hz, 1H), 5.70-5.59 (m, 1H), 4.92 – 4.81 (m, 2H), 3.50 (2 ddd, ABXY system, *J* = 15.6, 10.6, 4.9 Hz, 2H), 3.13 (dd, ABX system, J_{ab} = 14.4, J_{ax} = 6.2 Hz, 1H), 2.92 (dd, ABX system, J_{ba} = 14.4, J_{bx} = 7.6 Hz, 1H), 2.24 (s, 3H), 1.91-1.82 (m, 1H), 1.67 – 1.51 (m, 1H), 1.37 (s, 9H), 0.93 (t, *J* = 7.4 Hz, 3H). **^{13}C-NMR** ($CDCl_3$, 100.6 MHz): δ (ppm) 173.0, 171.1, 159.4 (d, *J* = 246.6 Hz), 133.9, 128.4 (d, *J* = 3.4 Hz), 128.3 (d, *J* = 5.1 Hz), 123.4 (d, *J* = 3.3 Hz), 118.23, 115.7 (d, *J* = 23.7 Hz), 70.09 (d, *J* = 2.6 Hz), 51.0, 48.5, 41.1, 28.4, 24.1, 23.3, 11.5. **HRMS:**

Calculated for [M-CONH*t*-Bu] $C_{15}H_{19}FNO$: 248.1496, Found: 248.1507. **I.R.** (thin film): ν 3269, 3075, 2963, 2931, 2875, 1674, 1627, 1544, 1489, 1402, 1363, 1219, 914 and 754 cm^{-1}.

N-tert-butyl-2-(4-chlorophenyl)-5-phenyl-2-(N-propylacetamido)pent-4-enamide (6a)

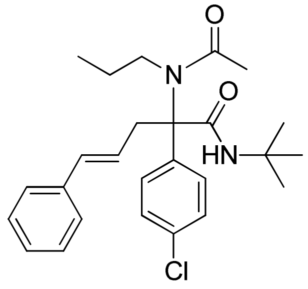

6a

This compound was synthesized according to the general procedure **III**, using Ugi product **1a** (163 mg, 0.5 mmol), sodium hydride (50 mg, 1.25 mmol), cinnamyl bromide (148 mg, 0.75 mmol). Purification by flash chromatography using (Et_2O : DCM = 7 : 93) gave the desired product in 88% isolated yield (193 mg, 0.44 mmol) as colourless oil. $\mathbf{R_f}$**:** (Et_2O : DCM = 0.7 : 9.3) = 0.41. **^{1}H-NMR** ($CDCl_3$, 400 MHz): δ (ppm) 7.43 – 6.46 (m, 9H), 6.13 (d, J = 15.6 Hz, 1H), 5.84 (dt, J = 16.0, 7.2 Hz, 1H), 3.44 – 3.28 (m, 2H), 3.10 (dd, ABX system, J_{ab} = 14.4, J_{ax} = 6.4 Hz, 1H), 2.92 (dd, ABX system, J_{ba} = 14.4, J_{bx} = 7.4 Hz, 1H), 2.14 (s, 3H), 1.78 – 1.67 (m, 1H), 1.58-1.43 (m, 1H), 1.24 (s, 9H), 0.80 (t, J = 7.2 Hz, 3H). **^{13}C-NMR** ($CDCl_3$, 100.6 MHz): δ (ppm) 173.0, 171.2, 139.3, 137.2, 134.0, 132.6, 128.5, 127.9, 127.4, 126.4, 126.1, 125.0, 71.6, 51.2, 48.9, 41.8, 31.01, 28.4, 24.3, 23.6, 11.5. **HRMS:** Calculated for $C_{26}H_{33}ClN_2O_2$: 440.2231, Found: 440.2229. **I.R.** (thin film): ν 3428, 3083, 3062, 3028, 2969, 2934, 2877, 1670, 1633, 1547, 1493, 1404, 1366, 1243, 1224, 1098 and 1014 cm^{-1}.

N-tert-butyl-2-(4-chlorophenyl)-5-methyl-2-(N-propylacetamido)hex-4-enamide (6b)

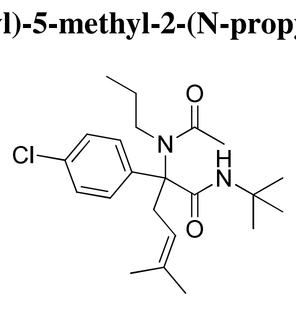

6b

This compound was synthesized according to the general procedure **III**, using Ugi product **1a** (163 mg, 0.5 mmol), sodium hydride (50 mg, 1.25 mmol), 2-methyl-4-bromo-2-butene (0.09 ml, 0.75 mmol). Purification by flash chromatography using (Et_2O : DCM = 10 : 90) gave the desired product in 74% isolated yield (146 mg, 0.37 mmol) as light yellow oil. **R_f:** (Et_2O : DCM = 1 : 9) = 0.35. **^{1}H-NMR** ($CDCl_3$, 400 MHz): δ (ppm) 7.28 (br s, 1H), 7.15 (d, *J* = 8.6 Hz, 2H), 7.03 (d, *J* = 6.7 Hz, 2H), 4.84 (t, *J* = 5.6 Hz, 1H), 3.26 (t, *J* = 8.4 Hz, 2H), 2.81 (d, *J* = 6.4 Hz, 2H), 2.14 (s, 3H), 1.73-1.65 (m, 1H), 1.52 (s, 3H), 1.45-1.36 (m, 1H), 1.28 (s, 3H), 1.23 (s, 9H), 0.76 (t, *J* = 7.3 Hz, 3H). **^{13}C-NMR** ($CDCl_3$, 100.6 MHz): δ (ppm) 173.0, 171.8, 138.8, 136.0, 132.4, 128.3, 127.6, 118.5, 71.5, 51.0, 49.0, 36.3, 28.4, 26.0, 24.4, 23.5, 17.9, 11.5. **HRMS:** Calculated for [M-$CONHCMe_3$] $C_{17}H_{23}ClNO$: 292.1474, Found: 292.1475. **I.R.** (thin film): ν 3430, 3059, 2969, 2933, 2877, 1668, 1631, 1547, 1492, 1405, 1366, 1244, 1224, 1097 and 1015 cm^{-1}.

N-(tert-butyl)-2-(4-chlorophenyl)-2-(cyclohex-2-en-1-yl)-2-(N-propylacetamido)acetamide (6c)

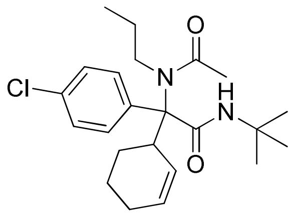

6c

This compound was synthesized according to the general procedure **III**, using Ugi product **1a** (163 mg, 0.5 mmol), sodium hydride (50 mg, 1.25 mmol), 3-bromocyclohexene (0.09 ml, 0.75 mmol). Purification by flash chromatography using (Et_2O : DCM = 5 : 95) gave the desired product in 30% isolated yield (60 mg, 0.15 mmol) as light yellow oil. **R_f:** (Et_2O : DCM = 0.5 : 9.5) = 0.45. **^{1}H-NMR** (CD_3OD, 400 MHz): δ (ppm) 7.21-7.13 (m, 4H), 6.82 (br s, 1H), 5.60 – 5.45 (m, 2H), 3.50 – 3.27 (m, 3H), 2.11 (s, 3H), 1.90 – 1.77 (m, 1H), 1.74 – 1.60 (m, 3H), 1.49 – 1.30 (m, 3H), 1.21 (br s, 10H), 0.75 (t, *J* = 7.3 Hz, 3H). **^{13}C-NMR** (CD_3OD, 100.6 MHz): δ (ppm) 176.0, 172.0, 138.1, 133.8, 131.3, 129.8, 129.4, 128.0, 75.4, 52.5, 41.5, 28.8, 26.4, 26.1, 24.5, 24.2, 23.0, 11.7. **HRMS:** Calculated for [M-$CONHCMe_3$] $C_{18}H_{23}ClNO$: 304.1474, Found: 304.1472. **I.R.** (thin film): ν 3426, 3037, 2967, 2935, 2877, 2839, 1669, 1635, 1544, 1492, 1456, 1394, 1367, 1243, 1224, 1097 and 1014 cm^{-1}.

N-(tert-butyl)-2-(4-chlorophenyl)-2-(N-propylacetamido)butanamide (6d)

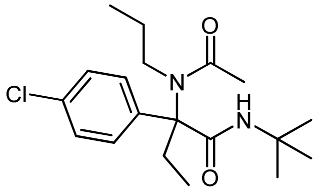

6d

This compound was synthesized according to the general procedure **III**, using Ugi product **1a** (163 mg, 0.5 mmol), sodium hydride (50 mg, 1.25 mmol), ethyl iodide (0.06 ml, 0.75 mmol). Purification by flash chromatography using (Et_2O : DCM = 7 : 93) gave the desired product in 94% isolated yield (165 mg, 0.47 mmol) as colourless oil. **R_f:** (Et_2O : DCM = 0.6 : 9.4) = 0.25. **^{1}H-NMR** ($CDCl_3$, 400 MHz): δ (ppm) 7.59 (br s, 1H), 7.16 (d, *J* = 8.3 Hz, 2H), 7.01 (br s, 2H), 3.44 – 3.27 (m, 2H), 2.18-2.12 (m, 4H), 1.89 (dq, *J* = 13.8, 7.0 Hz, 1H), 1.79-1.70 (m, 1H), 1.54-1.40 (m, 1H), 1.24 (s, 9H), 0.84 (t, *J* = 7.3 Hz, 3H), 0.56 (t, *J* = 6.4 Hz, 3H). **^{13}C-NMR** ($CDCl_3$, 100.6 MHz): δ (ppm) 173.2, 171.5, 139.7, 132.0, 127.7, 127.4, 71.5, 50.9, 48.4, 30.9, 28.4, 24.5, 23.5, 11.6, 9.4. **HRMS:** Calculated for [M-CONHCMe$_3$] $C_{14}H_{19}ClNO$: 252.1161, Found: 252.1153. **I.R.** (thin film): ν 3436, 3061, 2971, 2935, 2878, 1668, 1630, 1547, 1490, 1404, 1366, 1225, 1098 and 1014 cm^{-1}.

N-(tert-butyl)-2-(4-chlorophenyl)-2-(N-propylacetamido)-3-(p-tolyl)propanamide (6e)

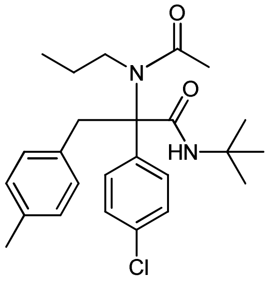

6e

This compound was synthesized according to the general procedure **III**, using Ugi product **1a** (163 mg, 0.5 mmol), sodium hydride (50 mg, 1.25 mmol), 4-methylbenzyl bromide (139 mg, 0.75 mmol). Purification by flash chromatography using (Et_2O : DCM = 5 : 95) gave the desired product in 79% isolated yield (170 mg, 0.40 mmol) as colourless oil. **R_f:** (Et_2O : DCM = 0.5 :

9.5) = 0.25. **^{1}H-NMR** (CD_3OD, 400 MHz): δ (ppm) 7.04 (d, J = 8.7 Hz, 2H), 6.92 (d, J = 8.3 Hz, 2H), 6.79 (d, J = 7.9 Hz, 2H), 6.64 (d, J = 8.0 Hz, 2H), 3.80 (d, AB system, J_{ab} = 12.6 Hz, 1H), 3.38 (dt, J = 10.6, 5.7 Hz, 2H), 3.02 (d, AB system, J_{ba} = 12.5 Hz, 1H), 2.13 (s, 3H), 2.09 (s, 3H), 1.87-1.75 (m, 1H), 1.63-1.54 (m, 1H), 1.18 (s, 9H), 0.80 (t, J = 7.4 Hz, 3H). **^{13}C-NMR** (CD_3OD, 100.6 MHz): δ (ppm) 175.2, 171.7, 140.4, 137.4, 134.0, 133.5, 132.4, 130.0, 129.4, 128.4, 74.5, 52.5, 43.5, 29.9, 28.7, 24.9, 24.1, 21.1, 11.5. **HRMS:** Calculated for [M-$CONHCMe_3$] $C_{20}H_{23}ClNO$: 328.1474, Found: 328.1480. **I.R.** (thin film): ν 3424, 2970, 2933, 2877, 1669, 1634, 1543, 1491, 1394, 1366, 1224, 1096 and 1015 cm^{-1}.

General procedure IV for Cascade reaction

To a 1 M solution of aldehyde in methanol were added successively 1.0 equiv of amine, 1.0 equiv of acid and 1.0 equiv of isocyanide. The resulting mixture was stirred at room temperature for 1 day. Then methanol was removed under reduced pressure and replaced by 0.5 M DMSO, sodium hydride (2.5 equiv) was added and the resulting solution was stirred at room temp under Argon for 5 min, followed by the addition of allyl bromide (1.5 equiv), stirring was then continued at room temperature for extra 2-4 hrs depending on the reaction progress checked by TLC. After the completion of the reaction, it was diluted with CH_2Cl_2 and extracted with water. The organic phase was dried over $MgSO_4$ and the solvent was removed under reduced pressure. The crude residue was purified by flash chromatography on silica gel to give a pure product.

N-(tert-butyl)-2-(4-chlorophenyl)-2-(N-propylacetamido)pent-4-enamide (3a)

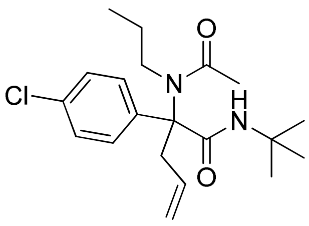

3a

This compound was synthesized according to the general procedure **IV**, using 4-chlorobenzaldehyde (141 mg, 1.0 mmol), propylamine (0.08 ml, 1.0 mmol), acetic acid (0.06 ml, 1.0 mmol) and tert-butyl isocyanide (0.11 ml, 1.0 mmol), sodium hydride (100 mg, 2.5 mmol) and allylbromide (0.13 ml, 1.5 mmol). Purification by flash chromatography using (Et_2O : DCM = 10 : 90) gave the desired product in 81% isolated yield (297 mg, 0.81 mmol) as yellow oil.

N-(tert-butyl)-2-(N-propylacetamido)-2-(pyridin-2-yl)pent-4-enamide (3g)

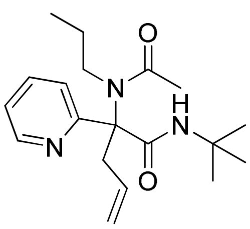

3g

This compound was synthesized according to the general procedure **IV**, using pyridine-2-carboxaldehyde (0.1 ml, 1.0 mmol), propylamine (0.08 ml, 1.0 mmol), acetic acid (0.06 ml, 1.0 mmol) and tert-butyl isocyanide (0.11 ml, 1.0 mmol), sodium hydride (100 mg, 2.5 mmol) and allylbromide (0.13 ml, 1.5 mmol). Purification by flash chromatography using (Et_2O : DCM = 20 : 80) gave the desired product in 78% isolated yield (257 mg, 0.78 mmol) as yellow oil.

N-(tert-butyl)-2-(4-chlorophenyl)-2-(N-(4-methoxybenzyl)acetamido)pent-4-enamide (3k)

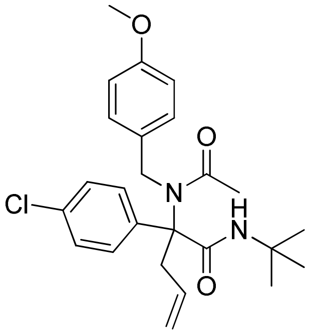

3k

This compound was synthesized according to the general procedure **IV**, using 4-chlorobenzaldehyde (141 mg, 1.0 mmol), 4-methoxybenzylamine (0.13 ml, 1.0 mmol), acetic acid (0.06 ml, 1.0 mmol) and tert-butyl isocyanide (0.11 ml, 1.0 mmol), sodium hydride (100 mg, 2.5 mmol) and allylbromide (0.13 ml, 1.5 mmol). Purification by flash chromatography using (Et_2O : DCM = 10 : 90) gave the desired product in 64% isolated yield (282 mg, 0.64 mmol) as yellow oil.

2-(N-allylacetamido)-N-(tert-butyl)-2-(4-chlorophenyl)pent-4-enamide (3m)

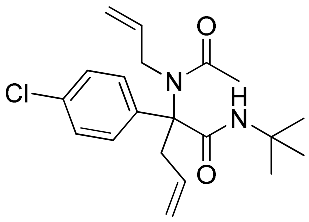

3m

This compound was synthesized according to the general procedure **IV**, using 4-chlorobenzaldehyde (141 mg, 1.0mmol), allylamine (0.075 ml, 1.0 mmol), acetic acid (0.06 ml, 1.0 mmol) and tert-butyl isocyanide (0.11 ml, 1.0 mmol), sodium hydride (100 mg, 2.5 mmol)

and allylbromide (0.13 ml, 1.5 mmol). Purification by flash chromatography using (Et_2O : DCM = 7 : 93) gave the desired product in 88% isolated yield (320 mg, 0.88 mmol) as yellow oil.

2-(N-allylacetamido)-N-(tert-butyl)-2-(pyridin-2-yl)pent-4-enamide (3o)

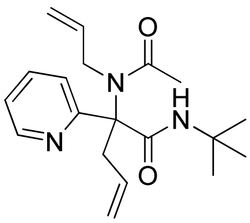

3o

This compound was synthesized according to the general procedure **IV**, using pyridine-2-carboxaldehyde (0.1 ml, 1.0 mmol), allylamine (0.075 ml, 1.0 mmol), acetic acid (0.06 ml, 1.0 mmol) and tert-butyl isocyanide (0.11 ml, 1.0 mmol), sodium hydride (100 mg, 2.5 mmol) and allylbromide (0.13 ml, 1.5 mmol). Purification by flash chromatography using (Et_2O : DCM = 30 : 70) gave the desired product in 87% isolated yield (287 mg, 0.87 mmol) as yellow oil.

General procedure V for Ring Closure Metathesis step

A solution of allylated Ugi product (**3**) (1.0 equiv) and Hoveyda-Grubbs catalyst 2nd Generation (2.5 mol%) in Toluene (0.4 M) were stirred at 60 °C for 4 hours. The resulting mixture was filtered then the solvent was evaporated under reduced pressure. The crude residue was purified by flash chromatography on silica gel to afford the corresponding cyclic product.

1-acetyl-N-tert-butyl-2-(4-chlorophenyl)-1,2,3,6-tetrahydropyridine-2-carboxamide (7a)

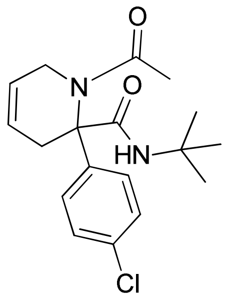

7a

This compound was synthesized according to the general procedure **V**, using allylated Ugi product **3m** (145 mg, 0.4 mmol) and Hoveyda-Grubbs catalyst 2nd Generation (6 mg, 0.01 mmol). Purification by flash chromatography using (Et_2O : DCM = 20 : 80) gave the desired product in 89% isolated yield (119 mg, 0.36 mmol) as light brown oil. **R_f:** (Et_2O : DCM = 1.5 : 8.5) = 0.23. **^{1}H-NMR** ($CDCl_3$, 400 MHz): δ (ppm) 7.21 (d, J = 8.7 Hz, 2H), 7.11 (d, J = 8.2 Hz, 2H), 6.42 (br s, 1H), 5.97-5.93 (m, 1H), 5.77-5.65 (m, 1H), 4.07-4.04 (m, 1H), 3.91 (dd, ABX system, J_{ab} = 16.3, J_{ax} = 1.5 Hz, 1H), 2.99 (dd, ABX system, J_{ab} = 16.3, J_{ax} =3.2 Hz, 1H), 2.38 (dd, ABX system, J_{ba} = 16.3, J_{bx} =3.6 Hz, 1H), 2.14 (s, 3H), 1.22 (s, 9H). **^{13}C-NMR** ($CDCl_3$, 100.6 MHz): δ (ppm) 172.7, 170.6, 139.9, 133.0, 128.6, 127.6, 127.2, 122.6, 68.8, 51.2, 45.6, 36.7, 28.5, 24.1. **HRMS:** Calculated for [M-CONHCMe3] $C_{13}H_{13}ClNO$: 234.0691, Found: 234.0887. **I.R.** (thin film): ν 3428, 3061, 2970, 2931, 1677, 1601, 1513, 1493, 1369, 1269, 1221, 1098 and 1015 cm^{-1}.

1-acetyl-2-(4-chlorophenyl)-N-cyclohexyl-1,2,3,6-tetrahydropyridine-2-carboxamide (7b)

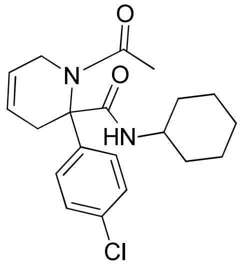

7b

This compound was synthesized according to the general procedure **V**, using allylated Ugi product **3n** (156 mg, 0.4 mmol) and Hoveyda-Grubbs catalyst 2nd Generation (6 mg, 0.01 mmol). Purification by flash chromatography using (pure Et_2O) gave the desired product in 64% isolated yield (89 mg, 0.26 mmol) as light brown oil. $\mathbf{R_f}$**:** (Et_2O) = 0.32. ^{1}**H-NMR** ($CDCl_3$, 400 MHz): δ (ppm) 7.22 (d, J = 8.7 Hz, 2H), 7.13 (d, J = 8.2 Hz, 2H), 6.25 (br s, 1H), 5.96-5.91 (m, 1H), 5.75-5.62 (m, 1H), 4.10-4.06 (m, 1H), 3.87 (dd, ABX system, J_{ab} = 16.4, J_{ax} = 1.3 Hz, 1H), 3.73-3.64 (m, 1H), 3.04 (dd, ABX system, J_{ab} = 16.3, J_{ax} = 2.1 Hz, 1H), 2.45 (dd, ABX system, J_{ba} = 16.3, J_{bx} = 4.5 Hz, 1H), 2.14 (s, 3H), 1.79-1.75 (m, 2H), 1.55-1.44 (m, 3H), 1.31-1.18 (m, 2H), 1.09-0.94 (m, 3H). ^{13}**C-NMR** ($CDCl_3$, 100.6 MHz): δ (ppm) 172.7, 170.8, 139.2, 133.2, 128.7, 127.9, 126.9, 122.9, 68.1, 48.5, 45.6, 36.4, 32.6, 25.7, 24.7, 24.0. **HRMS:** Calculated for $C_{20}H_{25}ClN_2O_2$: 360.1605, Found: 360.1613. **I.R.** (thin film): ν 3427, 3055, 2935, 2857, 1659, 1511, 1493, 1406, 1345, 1253, 1221, 1098 and 1015 cm^{-1}.

1-acetyl-N-tert-butyl-2-(pyridin-2-yl)-1,2,3,6-tetrahydropyridine-2-carboxamide (7c)

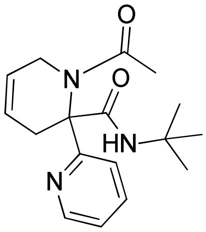

7c

This compound was synthesized according to the general procedure **V**, using allylated Ugi product **3o** (132 mg, 0.4 mmol) and Hoveyda-Grubbs catalyst 2nd Generation (6 mg, 0.01 mmol). Purification by flash chromatography using (EtOAc : DCM = 20 : 80) gave the desired product in

72% isolated yield (87 mg, 0.29 mmol) as light brown oil. **R$_f$:** (EtOAc : DCM = 1 : 9) = 0.22. **^{1}H-NMR** ($CDCl_3$, 400 MHz): δ (ppm) 8.64 (br s, 1H), 8.45 (br s, 1H), 7.65-7.49 (m, 1H), 7.15– 7.00 (m, 2H), 6.04 – 5.82 (m, 1H), 5.71-5.55 (m, 1H), 4.13 (br s, 2H), 2.85-2.73 (m, 2H), 2.18 (s, 3H), 1.29 (s, 9H). **^{13}C-NMR** ($CDCl_3$, 100.6 MHz): δ (ppm) 171.5, 170.2, 161.4, 153.1, 147.7, 136.8, 126.2, 124.8, 121.7, 68.5, 50.9, 45.2, 36.8, 28.6, 23.5. **HRMS:** Calculated for $C_{17}H_{23}N_3O_2$: 301.179, Found: 301.1801. **I.R.** (thin film): ν 3235, 3060, 2971, 2931, 2876, 1677, 1604, 1591, 1434, 1367, 1279, 1224 and 1021cm^{-1}.

N-tert-butyl-2-(4-chlorophenyl)-1-(4-methoxybenzoyl)-1,2,3,6-tetrahydropyridine-2-carboxamide (7d)

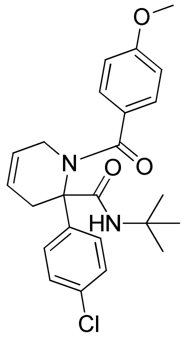

7d

This compound was synthesized according to the general procedure **V**, using allylated Ugi product **3p** (182 mg, 0.4 mmol) and Hoveyda-Grubbs catalyst 2nd Generation (6 mg, 0.01 mmol). Purification by flash chromatography using (Et_2O : DCM = 4 : 96) gave the desired product in 68% isolated yield (116 mg, 0.27 mmol) as light brown oil. **R$_f$:** (Et_2O : DCM = 0.4 : 9.6) = 0.32. **^{1}H-NMR** ($CDCl_3$, 400 MHz): δ (ppm) 7.55 (d, J = 8.8 Hz, 2H), 7.28 (d, J = 8.7 Hz, 2H), 7.21 (d, J = 9.0 Hz, 2H), 6.88 (d, J = 8.8 Hz, 2H), 6.78 (br s, 1H), 5.90-5.85 (m, 1H), 5.50-5.46 (m, 1H), 3.98-3.84 (m, 2H), 3.79 (s, 3H), 3.15 (ddd, ABXY system, J_{ab} = 17.1, J_{ax} = 4.1, J_{ay} =1.9 Hz, 1H), 2.55 (ddd, ABXY system, J_{ba} = 17.1, J_{bx} = 3.8, J_{by} = 2.0 Hz, 1H), 1.23 (s, 9H). **^{13}C-NMR** ($CDCl_3$, 100.6 MHz): δ (ppm) 175.2, 170.2, 162.2, 140.0, 132.9, 130.5, 128.5, 128.0, 127.3, 126.1, 123.0, 114.0, 68.1, 55.5, 51.2, 48.3, 34.4, 28.6. **HRMS:** Calculated for $C_{24}H_{27}ClN_2O_3$: 426.171, Found: 426.1729. **I.R.** (thin film): ν 3432, 3046, 2967, 2935, 2870, 2841, 1676, 1636, 1607, 1548, 1510, 1394, 1255, 1171, 1096 and 1032 cm^{-1}.

N-tert-butyl-2-(4-chlorophenyl)-1-(3-(4-methoxyphenyl)propanoyl)-1,2,3,6-tetrahydropyridine-2-carboxamide (7e)

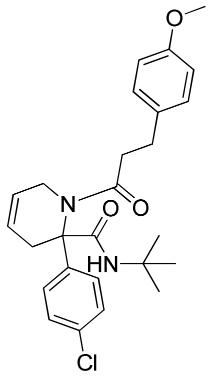

7e

This compound was synthesized according to the general procedure **V**, using allylated Ugi product **3q** (193 mg, 0.4 mmol) and Hoveyda-Grubbs catalyst 2nd Generation (6 mg, 0.01 mmol). Purification by flash chromatography using (Et_2O : PE = 50 : 50) gave the desired product in 72% isolated yield (131 mg, 0.288 mmol) as light brown oil. **R_f:** (Et_2O : PE = 5 : 5) = 0.22. **^{1}H-NMR** ($CDCl_3$, 400 MHz): δ (ppm) 7.15 (d, J = 8.7 Hz, 2H), 7.05 (d, J = 8.4 Hz, 2H), 7.01 (d, J = 8.5 Hz, 2H), 6.76 (d, J = 8.7 Hz, 2H), 6.63 (br s, 1H), 5.96-5.92 (m, 1H), 5.70 – 5.61 (m, 1H), 4.01-3.88 (m, 2H), 3.72 (s, 3H), 2.96 (dd, ABX system, J_{ab} = 16.2, J_{ax} = 4.9 Hz, 1H), 2.83 (t, J = 7.4 Hz, 2H), 2.67-2.63 (m, 2H), 2.29 (dd, ABX system, J_{ba}= 16.2, J_{bx} = 2.7 Hz, 1H), 1.21 (s, 9H). **^{13}C-NMR** ($CDCl_3$, 100.6 MHz): δ (ppm) 174.5, 170.7, 158.1, 140.2, 133.1, 132.7, 129.5, 128.5, 127.6, 127.3, 122.7, 113.9, 69.1, 55.3, 51.2, 44.8, 37.5, 37.0, 30.0, 28.5. **HRMS:** Calculated for $C_{26}H_{31}ClN_2O_3$: 454.2033, Found: 454.2012. **I.R.** (thin film): ν 3429, 2967, 2935, 2873, 2838, 1673, 1611, 1513, 1492, 1405, 1367, 1247, 1097, 1036 and 1015 cm^{-1}.

N-(tert-butyl)-2-(4-chlorophenyl)-6-oxo-1-propyl-1,2,3,6-tetrahydropyridine-2-carboxamide (9)

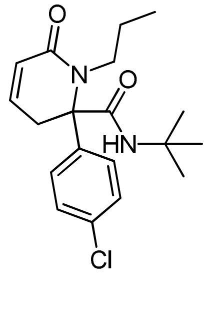

9

This compound was synthesized according to the general procedure **V**, using allylated Ugi product **3r** (181 mg, 0.4 mmol) and Hoveyda-Grubbs catalyst 2nd Generation (6 mg, 0.01mmol). Purification by flash chromatography using (Et_2O : DCM = 10 : 90) gave the desired product in 72% isolated yield (100 mg, 0.29 mmol) as light brown oil. **R_f:** (Et_2O : DCM = 1 : 9) = 0.3. **^{1}H-NMR** ($CDCl_3$, 400 MHz): δ (ppm) 7.30 (d, J = 8.8 Hz, 2H), 7.23 (d, J = 8.8 Hz, 2H), 6.43 (ddd, J = 9.8, 5.2, 3.3 Hz, 1H), 5.90 (dd, J = 9.8, 1.0 Hz, 1H), 5.64 (br s, 1H), 3.30 (ddd, ABXY system, J_{ab} = 17.7, J_{ax} = 5.2, J_{ay} = 1.0 Hz, 1H), 3.15 (ddd, ABXY system, J_{ab} = 12.9, J_{ax} = 11.1, J_{ay} = 4.9 Hz, 1H), 2.86 (ddd, ABXY system, J_{ba} = 12.9, J_{bx} = 11.3, J_{by} = 5.0 Hz, 1H), 2.79 (ddd, ABXY system, J_{ba} = 17.7, J_{bx} = 3.0, J_{by} = 2.6 Hz, 1H), 1.60-1.50 (m, 1H), 1.29 (br s, 10H), 0.62 (t, J = 7.4 Hz, 3H). **^{13}C-NMR** ($CDCl_3$, 100.6 MHz): δ (ppm) 170.0, 165.5, 138.6, 137.3, 134.6, 129.6, 128.9, 125.1, 72.7, 52.3, 47.4, 36.6, 28.5, 22.4, 11.5. **HRMS:** Calculated for [M-CONHCMe$_3$] $C_{14}H_{15}ClNO$: 248.0843, Found: 248.0831. **I.R.** (thin layer): ν 3449, 3058, 2971, 2934, 2876, 1667, 1612, 1510, 1455, 1403, 1368, 1221, 1098 and 1016 cm^{-1}.

Isomerization of double bond

1-acetyl-N-tert-butyl-2-(4-chlorophenyl)-1,2,3,4-tetrahydropyridine-2-carboxamide (8)

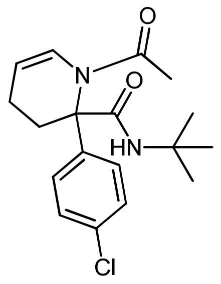

8

A solution of 1-acetyl-N-tert-butyl-2-(4-chlorophenyl)-1,2,3,6-tetrahydropyridine-2-carboxamide **7a** (101 mg, 0.3 mmol) and carbonylchlorohydridotris(triphenylphosphine)ruthenium (II) (14 mg, 0.015 mmol) in toluene (0.3 M) were refluxed for 1.5 hrs. The resulting mixture was filtered then the solvent was evaporated under reduced pressure. The crude residue obtained was purified by flash chromatography using (Et_2O : DCM = 10 : 90) affording the desired product in 81% isolated yield (82 mg, 0.25 mmol) as yellow oil. **R_f:** (Et_2O : PE = 1 : 9) = 0.31. **^{1}H-NMR** ($CDCl_3$, 400 MHz): δ (ppm) 7.25 (d, J = 8.8 Hz, 2H), 7.06 (d, J = 8.2 Hz, 2H), 6.61 (br s, 1H), 5.68 (br s, 1H), 4.96-4.87 (m, 1H), 2.47 – 2.40 (m, 1H), 2.23 (s, 3H), 2.14 (dt, ABX_2 system, J_{ab} = 13.7, J_{ax} = 4.5 Hz, 1H), 2.02-1.97 (m, 1H), 1.55-1.46 (m, 1H), 1.22 (s, 9H). **^{13}C-NMR** ($CDCl_3$, 100.6 MHz): δ (ppm) 170.3, 169.5, 137.1, 133.4, 128.9, 128.4, 125.6, 108.1, 68.2, 51.3, 34.7, 28.5, 23.1, 19.2. **HRMS:** Calculated for $C_{18}H_{23}ClN_2O_2$: 334.1448, Found: 334.1444. **I.R**. (thin film): ν 3431, 3073, 2971, 2931, 2848, 1676, 1650, 1513, 1493, 1455, 1374, 1341, 1275, 1221, 1099 and 1013 cm^{-1}.

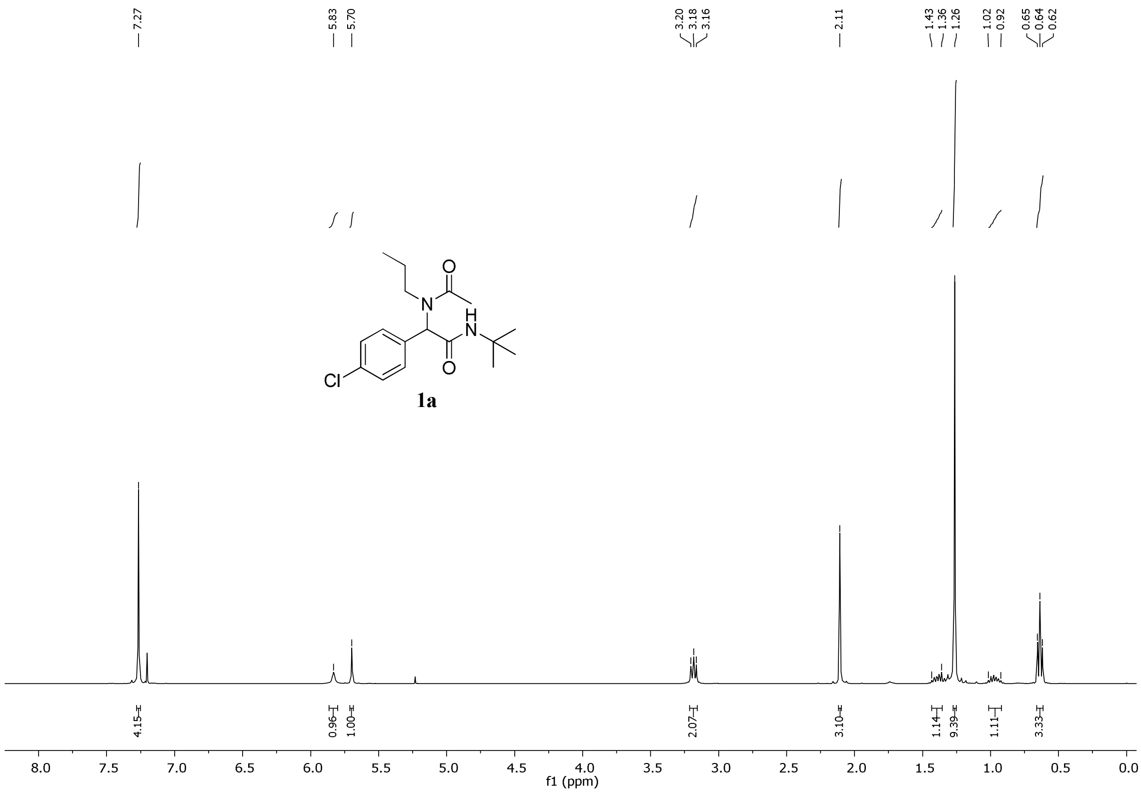
7.27
5.83
5.70
3.20
3.18
3.16
2.11
1.43
1.36
1.26
1.02
0.92
0.65
0.64
0.62
Cl
N
O
H
N
O
1a
4.15
0.96
1.00
2.07
3.10
1.14
9.39
1.11
3.33
8.0 7.5 7.0 6.5 6.0 5.5 5.0 4.5 4.0 3.5 3.0 2.5 2.0 1.5 1.0 0.5 0.0
f1 (ppm)

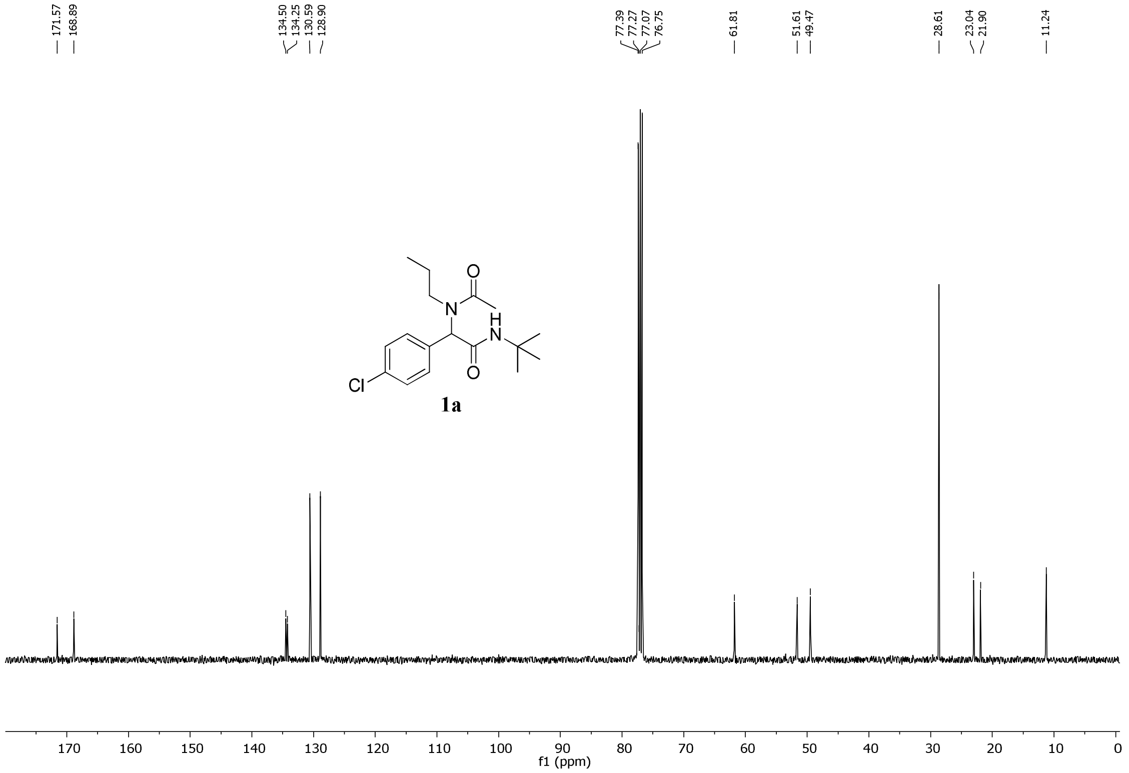
171.57
168.89
134.50
134.25
130.59
128.90
77.39
77.27
77.07
76.75
61.81
51.61
49.47
28.61
23.04
21.90
11.24
Cl
N
O
H
N
O
1a
170 160 150 140 130 120 110 100 90 80 70 60 50 40 30 20 10 0
f1 (ppm)

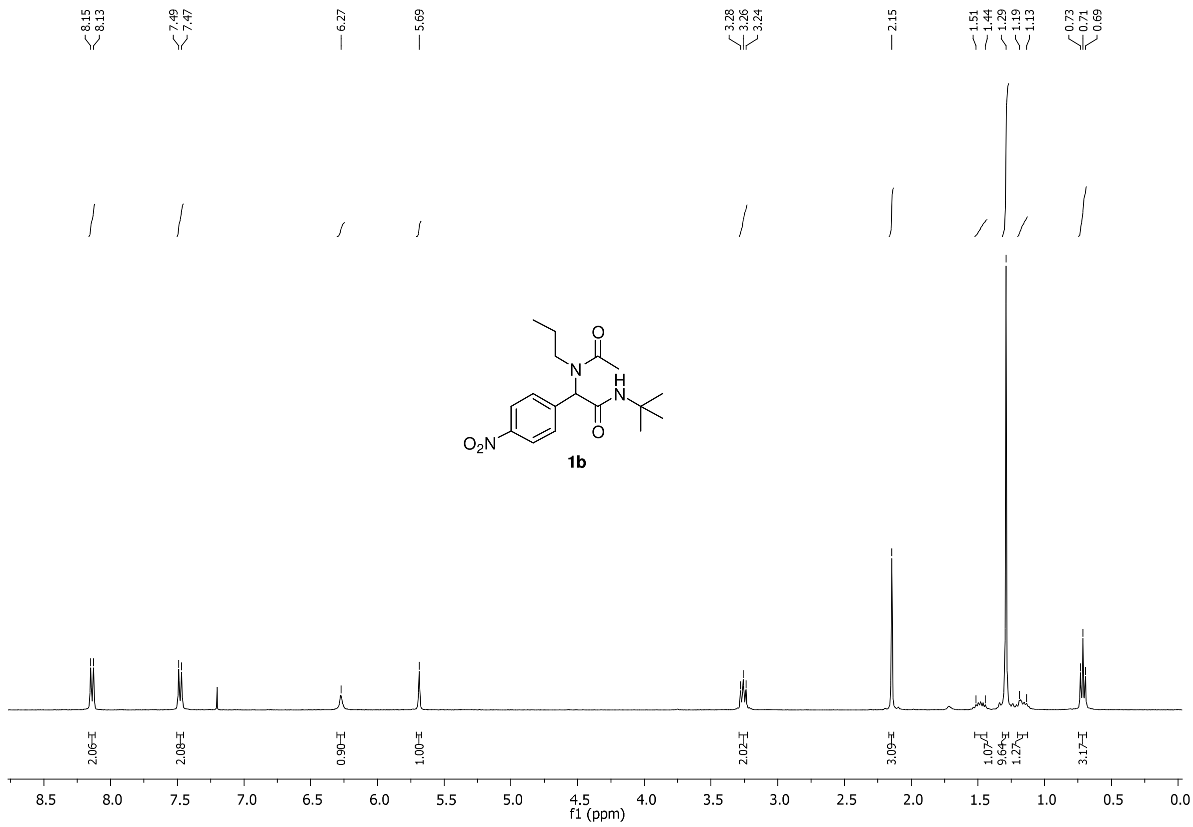
8.15
8.13
7.49
7.47
6.27
5.69
3.28
3.26
3.24
2.15
1.51
1.44
1.29
1.19
1.13
0.73
0.71
0.69
O
N
H
N
O
O_2N
1b
2.06
2.08
0.90
1.00
2.02
3.09
1.07
9.64
1.27
3.17
f1 (ppm)

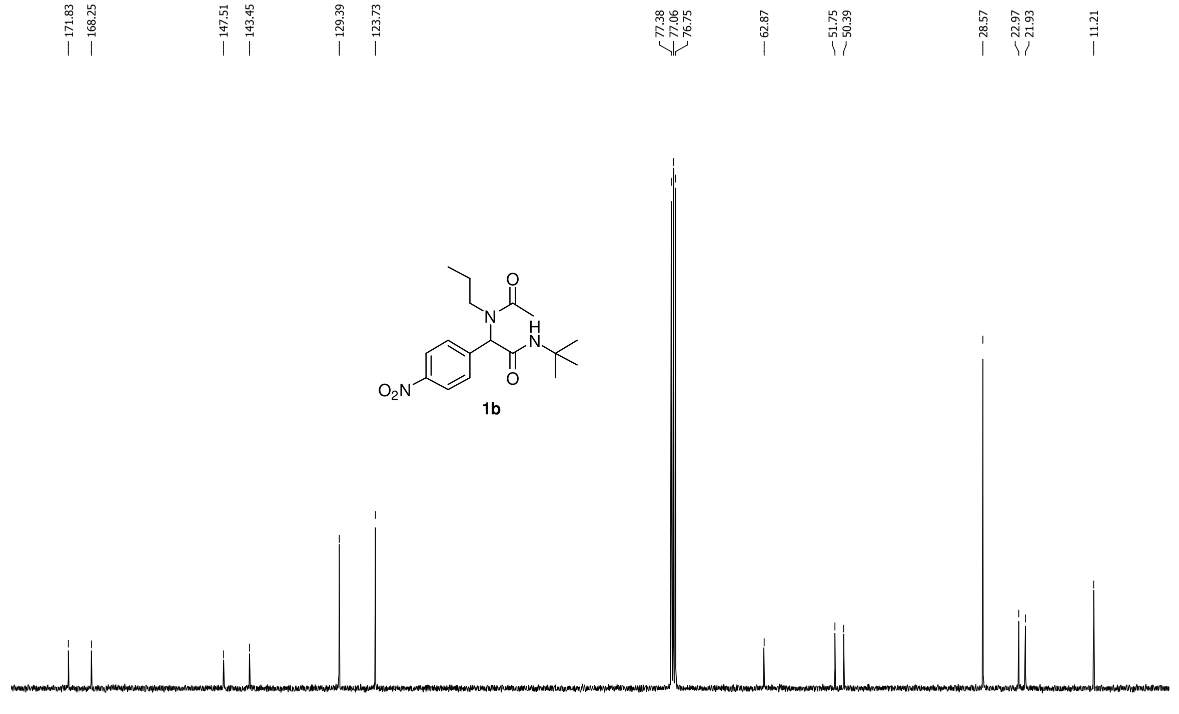
171.83
168.25
147.51
143.45
129.39
123.73
77.38
77.06
76.75
62.87
51.75
50.39
28.57
22.97
21.93
11.21
O
N
H
N
O
O_2N
1b
f1 (ppm)

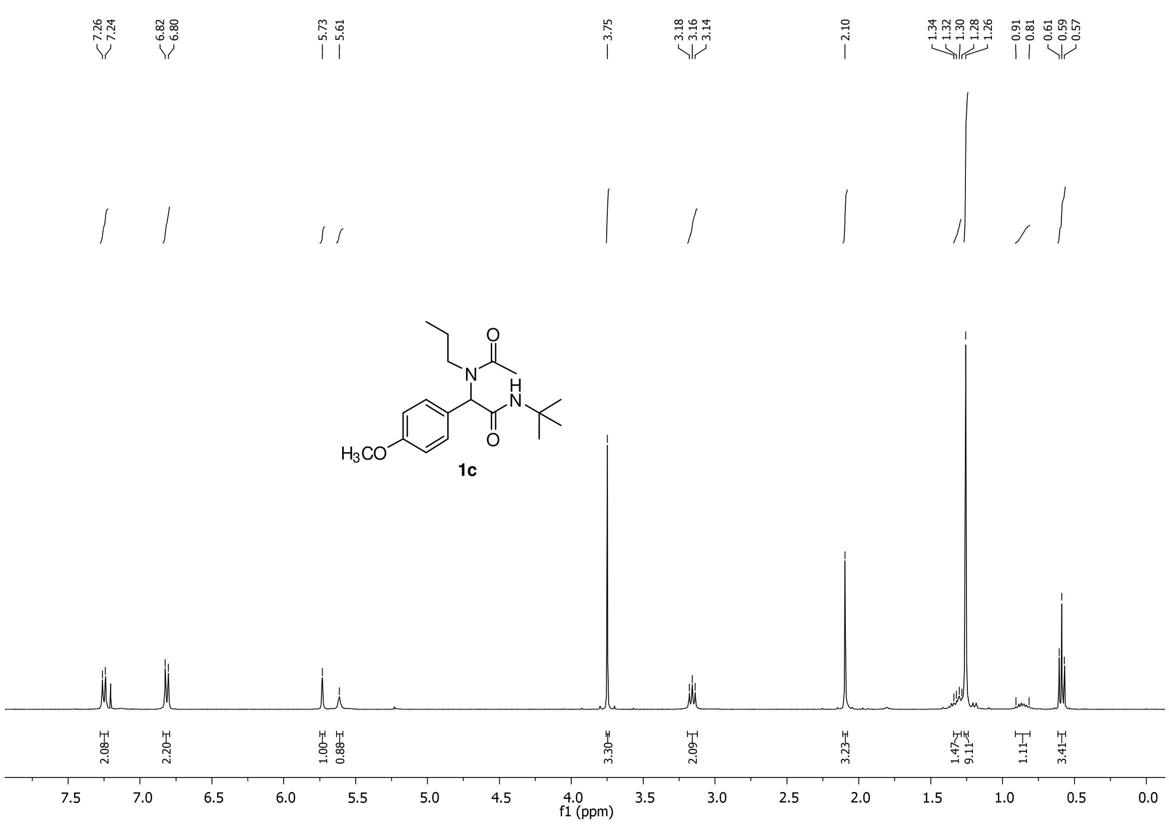
7.26
7.24
6.82
6.80
5.73
5.61
3.75
3.18
3.16
3.14
2.10
1.34
1.32
1.30
1.28
1.26
0.91
0.81
0.61
0.59
0.57
H3CO
N
H
O
1c
2.08
2.20
1.00
0.88
3.30
2.09
3.23
1.47
9.11
1.11
3.41
f1 (ppm)

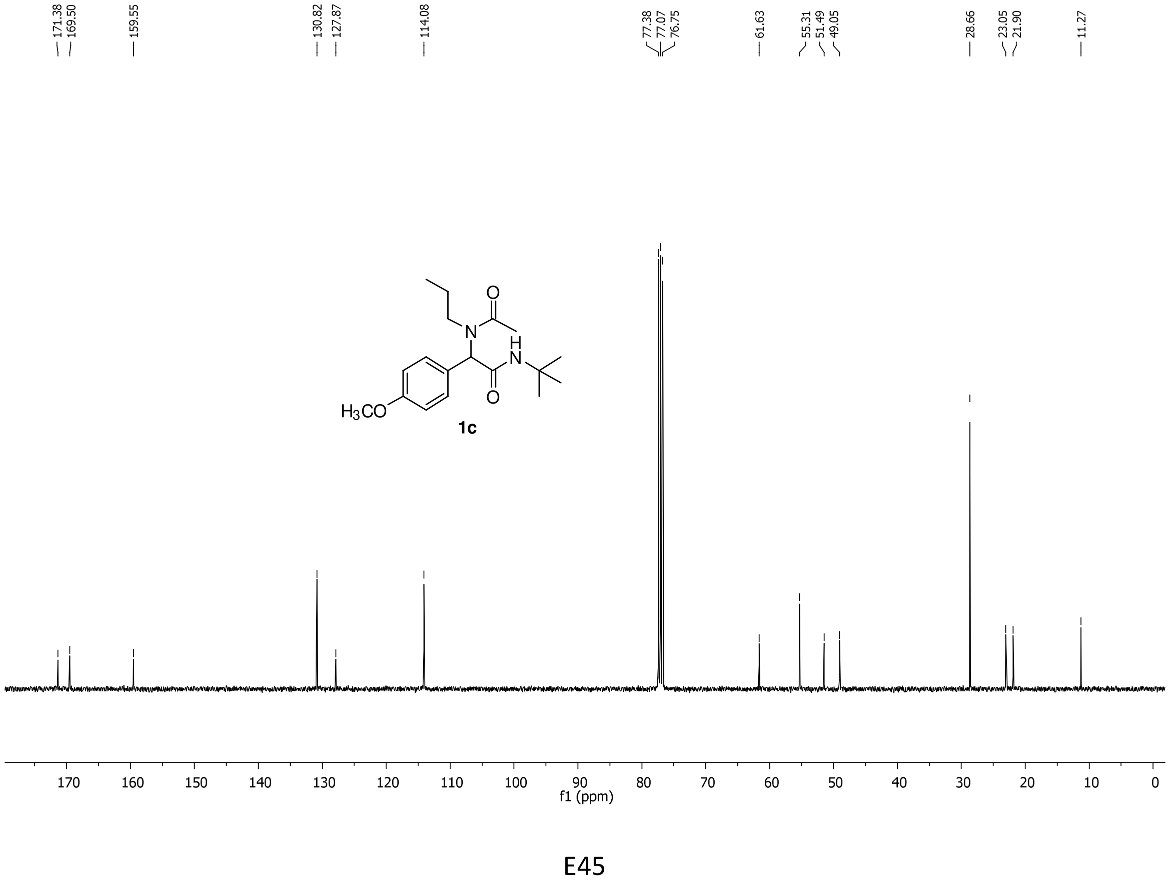
171.38
169.50
159.55
130.82
127.87
114.08
77.38
77.07
76.75
61.63
55.31
51.49
49.05
28.66
23.05
21.90
11.27
H3CO
N
H
O
1c
f1 (ppm)

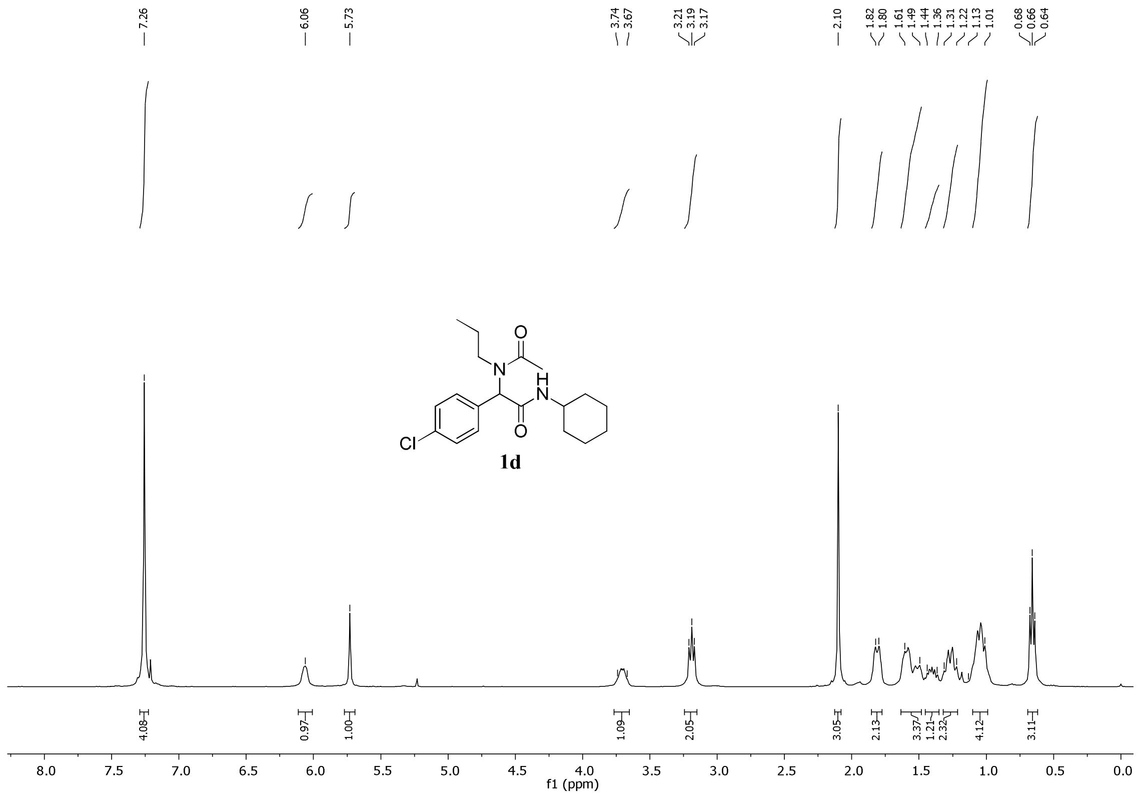
7.26
6.06
5.73
3.74
3.67
3.21
3.19
3.17
2.10
1.82
1.80
1.61
1.49
1.44
1.36
1.31
1.22
1.13
1.01
0.68
0.66
0.64
O
N
H
N
O
Cl
1d
4.08
0.97
1.00
1.09
2.05
3.05
2.13
3.37
1.21
2.32
4.12
3.11
8.0
7.5
7.0
6.5
6.0
5.5
5.0
4.5
4.0
3.5
3.0
2.5
2.0
1.5
1.0
0.5
0.0
f1 (ppm)

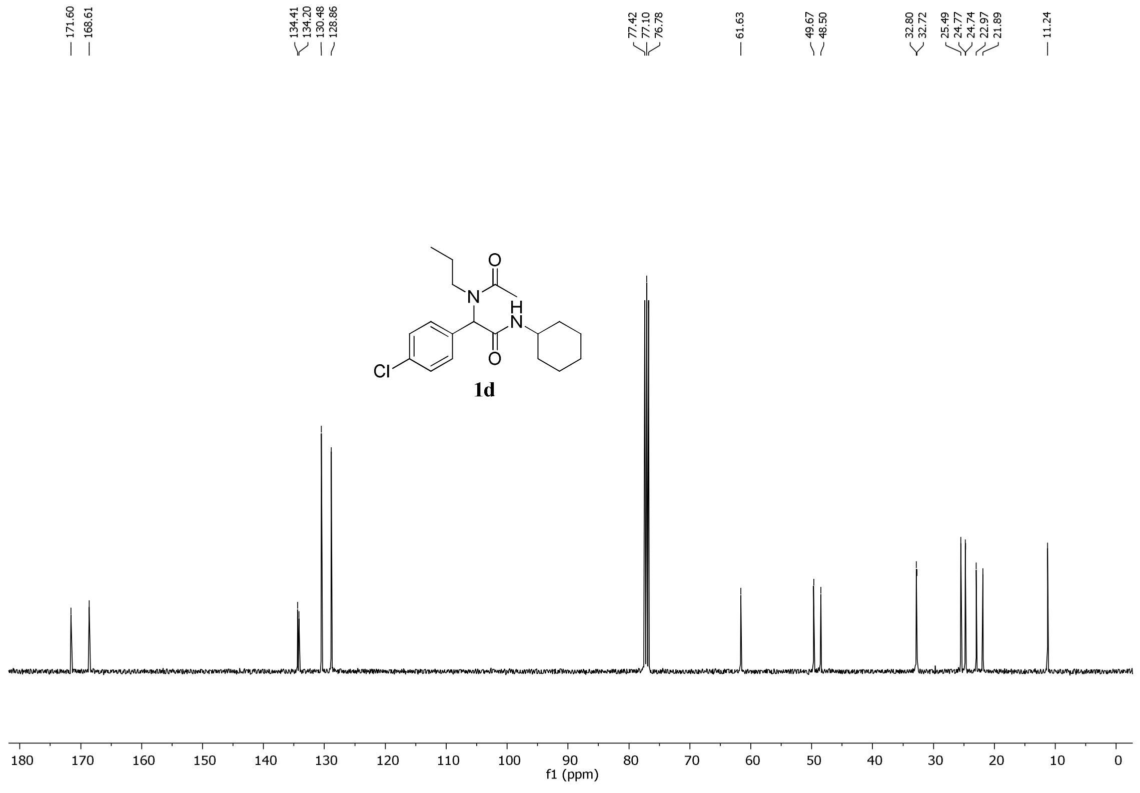
171.60
168.61
134.41
134.20
130.48
128.86
77.42
77.10
76.78
61.63
49.67
48.50
32.80
32.72
25.49
24.77
24.74
22.97
21.89
11.24
O
N
H
N
O
Cl
1d
180
170
160
150
140
130
120
110
100
90
80
70
60
50
40
30
20
10
0
f1 (ppm)

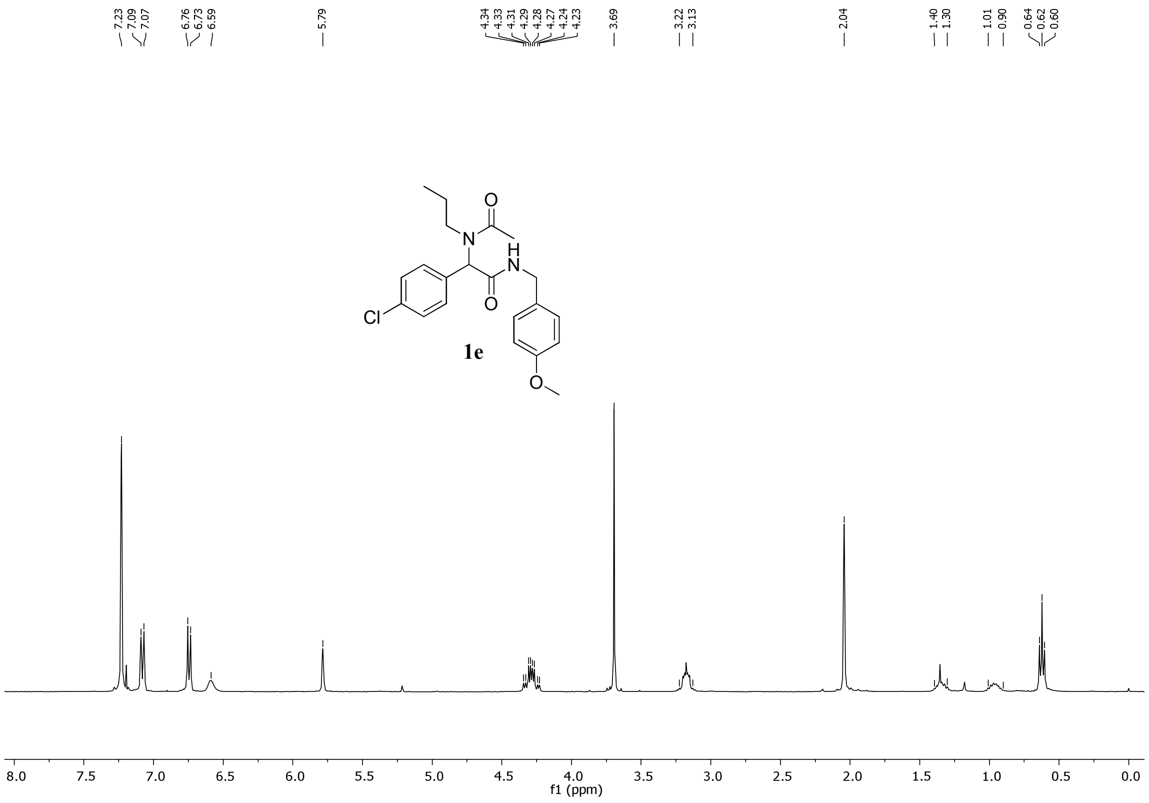
7.23
7.09
7.07
6.76
6.73
6.59
5.79
4.34
4.33
4.31
4.29
4.28
4.27
4.24
4.23
3.69
3.22
3.13
2.04
1.40
1.30
1.01
0.90
0.64
0.62
0.60
O
N
H
N
Cl
O
1e
O
8.0 7.5 7.0 6.5 6.0 5.5 5.0 4.5 4.0 3.5 3.0 2.5 2.0 1.5 1.0 0.5 0.0
f1 (ppm)

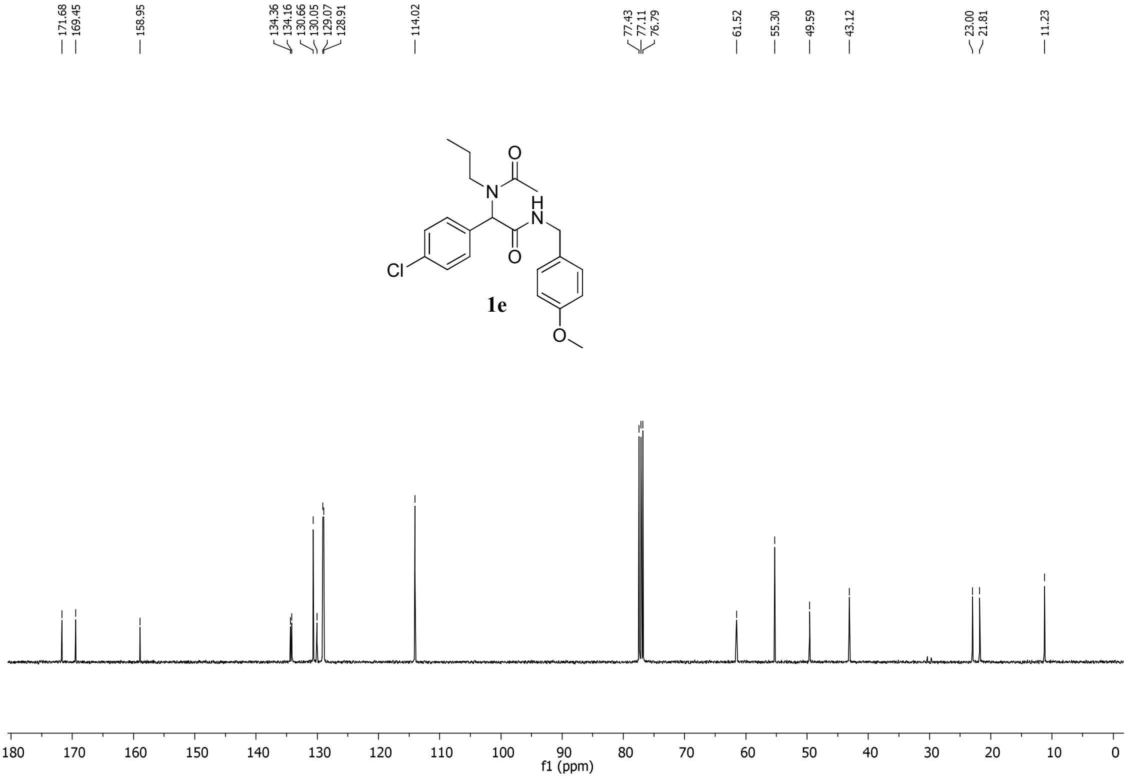
171.68
169.45
158.95
134.36
134.16
130.66
130.05
129.07
128.91
114.02
77.43
77.11
76.79
61.52
55.30
49.59
43.12
23.00
21.81
11.23
O
N
H
N
Cl
O
1e
O
180 170 160 150 140 130 120 110 100 90 80 70 60 50 40 30 20 10 0
f1 (ppm)

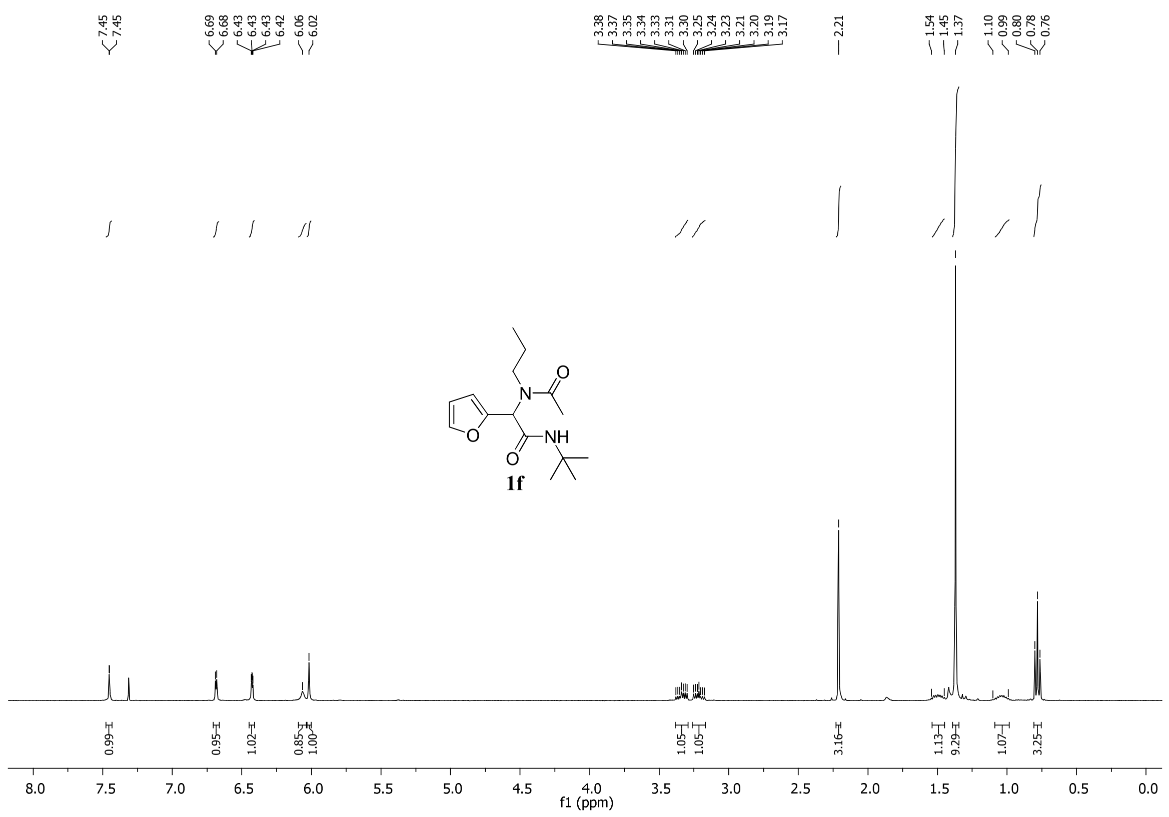
7.45
7.45
6.69
6.68
6.43
6.43
6.43
6.42
6.06
6.02
3.38
3.37
3.35
3.34
3.33
3.31
3.30
3.25
3.24
3.23
3.21
3.20
3.19
3.17
2.21
1.54
1.45
1.37
1.10
0.99
0.80
0.78
0.76
1f
0.99
0.95
1.02
0.85
1.00
1.05
1.05
3.16
1.13
9.29
1.07
3.25
f1 (ppm)

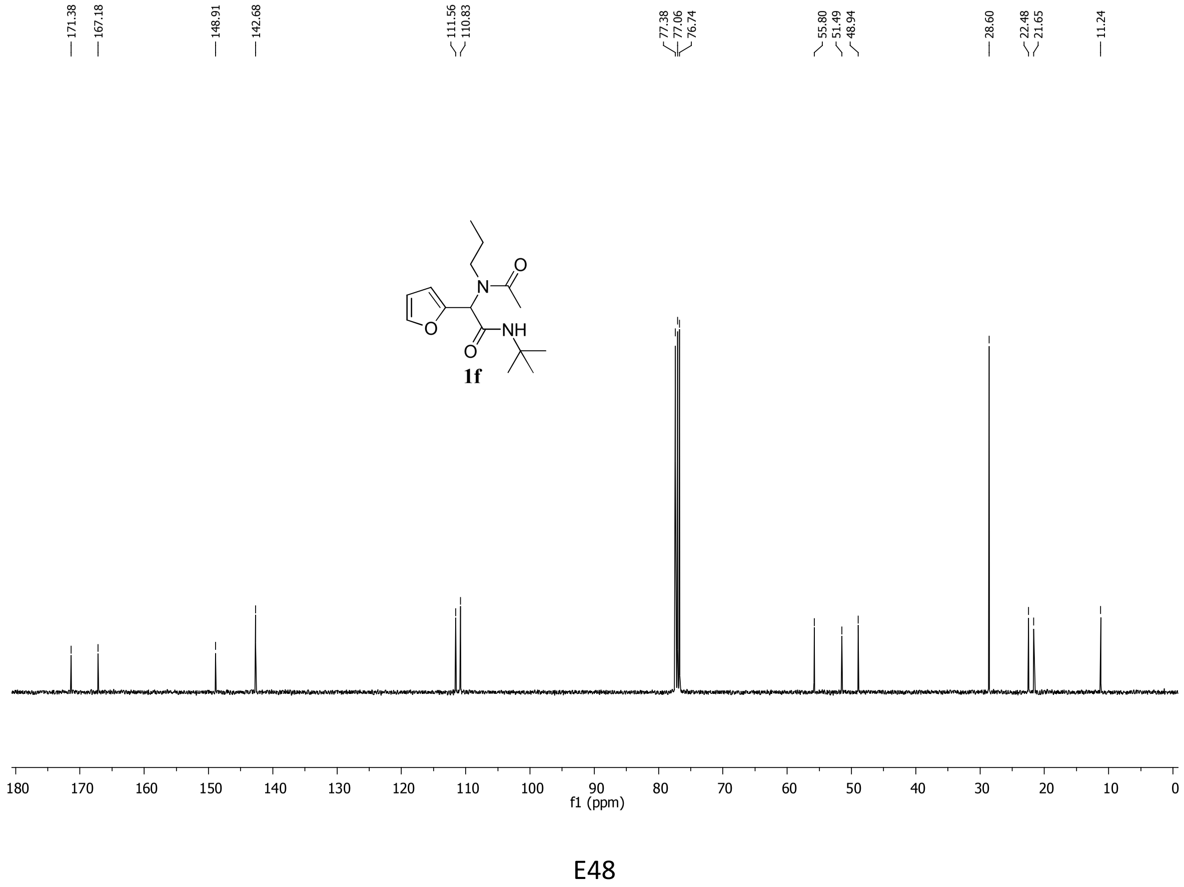
171.38
167.18
148.91
142.68
111.56
110.83
77.38
77.06
76.74
55.80
51.49
48.94
28.60
22.48
21.65
11.24
1f
f1 (ppm)

E48

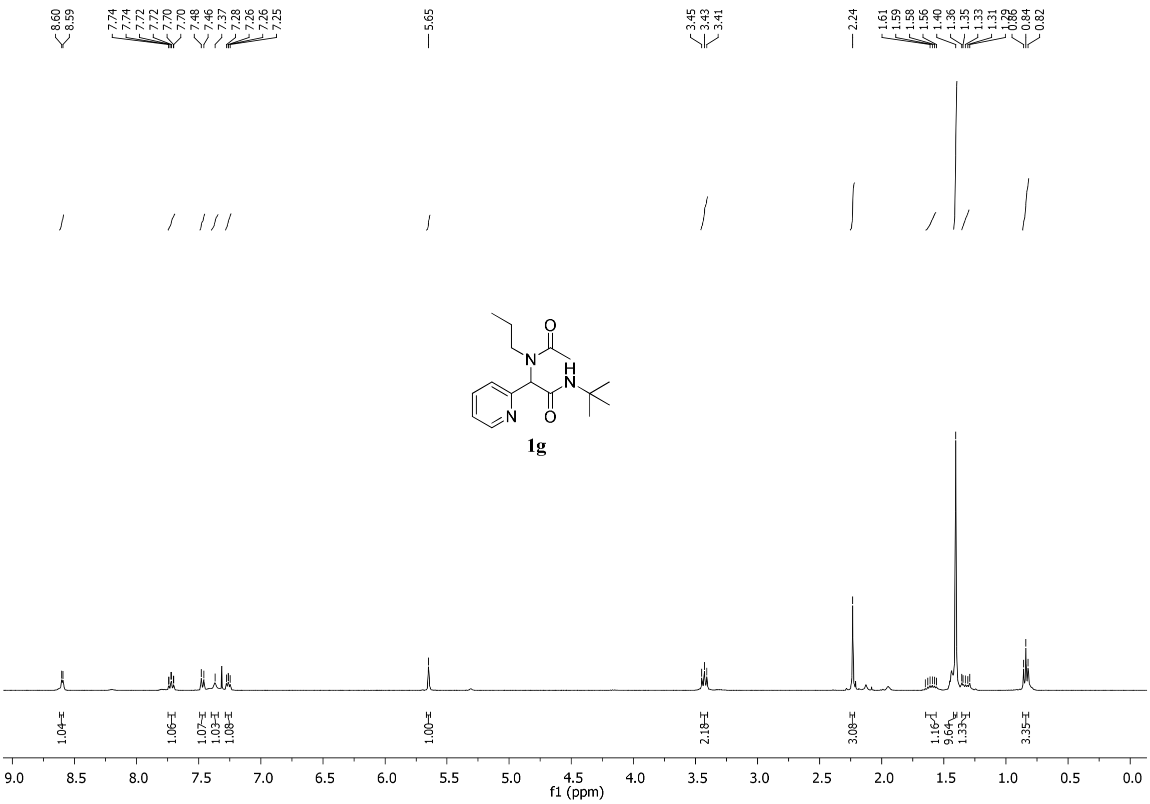
8.60
8.59
7.74
7.74
7.72
7.72
7.70
7.70
7.48
7.46
7.37
7.28
7.26
7.26
7.25
5.65
3.45
3.43
3.41
2.24
1.61
1.59
1.58
1.56
1.40
1.36
1.35
1.33
1.31
1.29
0.86
0.84
0.82
1g
1.04
1.06
1.07
1.03
1.08
1.00
2.18
3.08
1.16
9.64
1.33
3.35
9.0 8.5 8.0 7.5 7.0 6.5 6.0 5.5 5.0 4.5 4.0 3.5 3.0 2.5 2.0 1.5 1.0 0.5 0.0
f1 (ppm)

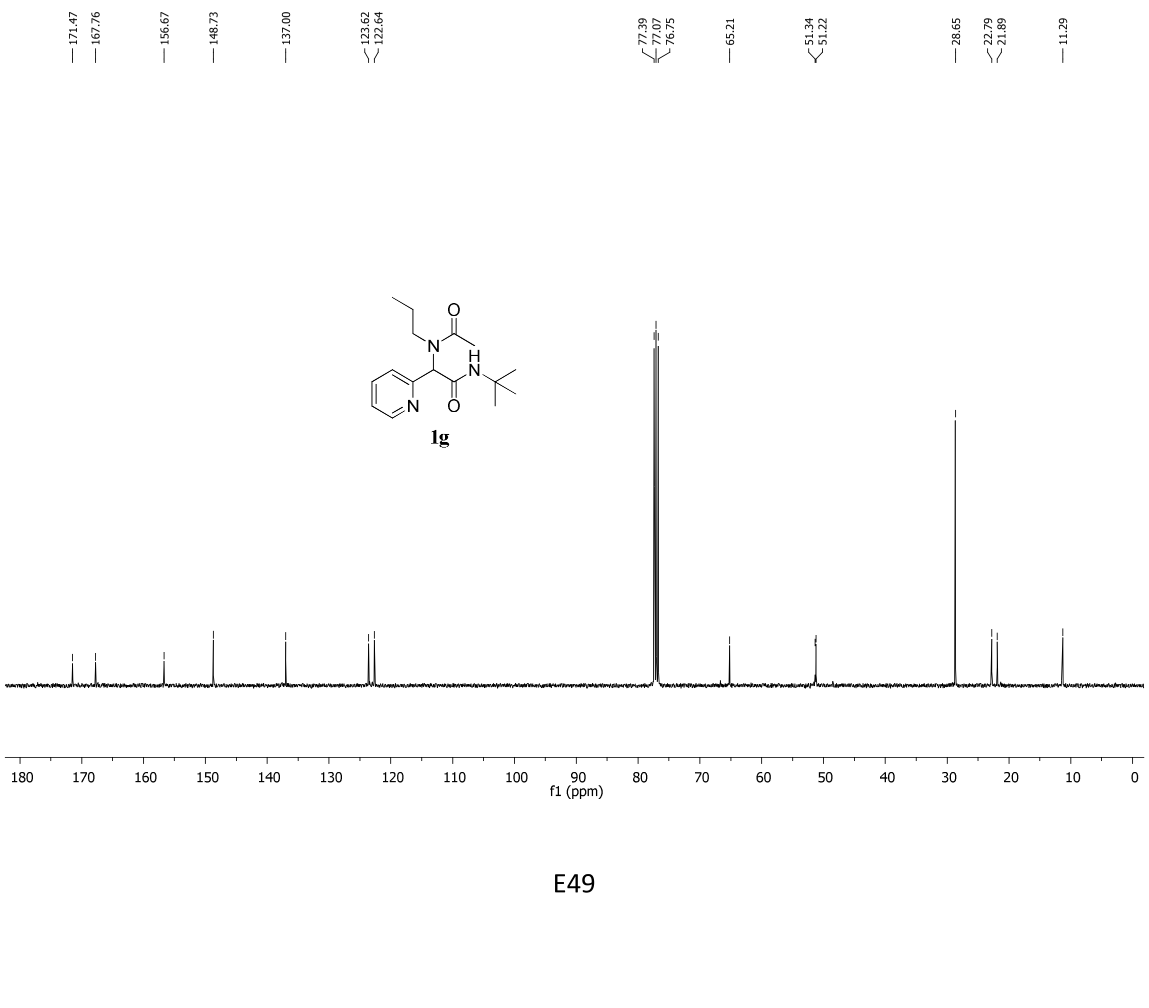
171.47
167.76
156.67
148.73
137.00
123.62
122.64
77.39
77.07
76.75
65.21
51.34
51.22
28.65
22.79
21.89
11.29
1g
180 170 160 150 140 130 120 110 100 90 80 70 60 50 40 30 20 10 0
f1 (ppm)

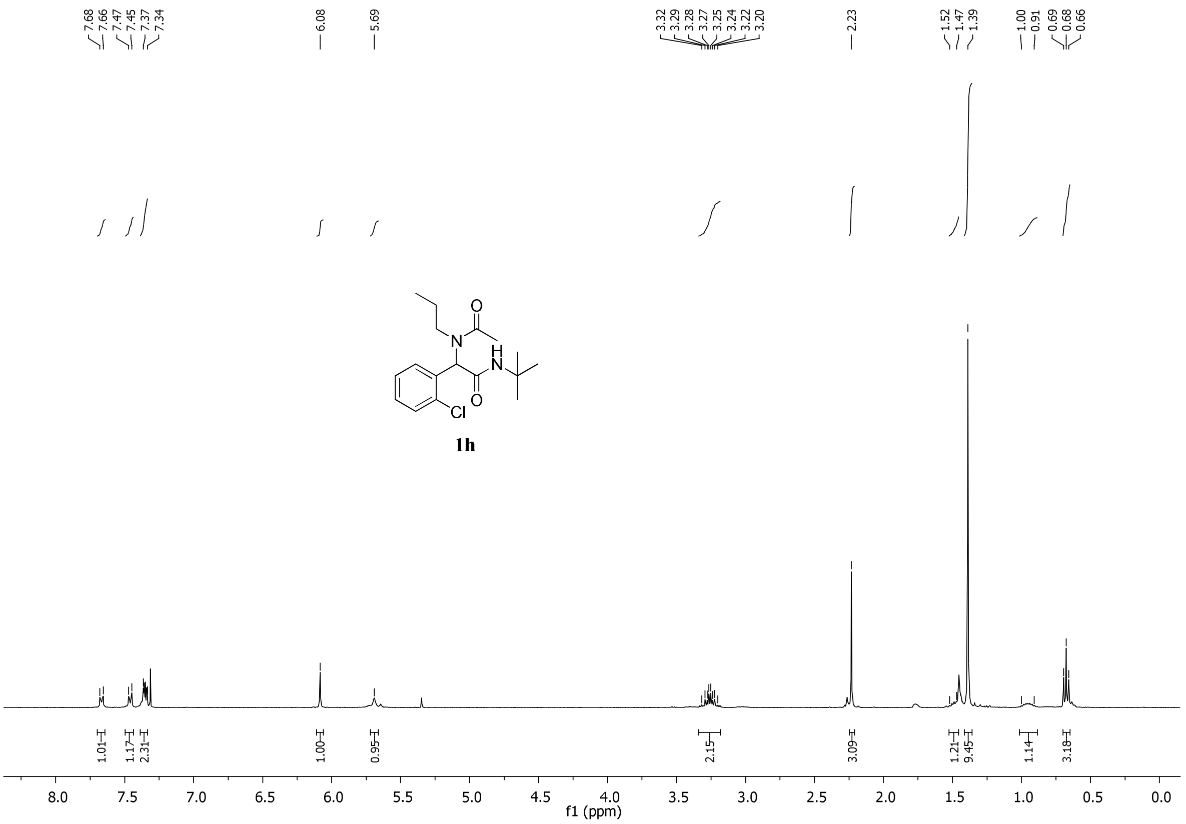
7.68
7.66
7.47
7.45
7.37
7.34
6.08
5.69
3.32
3.29
3.28
3.27
3.25
3.24
3.22
3.20
2.23
1.52
1.47
1.39
1.00
0.91
0.69
0.68
0.66
O
N
H
N
O
Cl
1h
1.01
1.17
2.31
1.00
0.95
2.15
3.09
1.21
9.45
1.14
3.18
f1 (ppm)

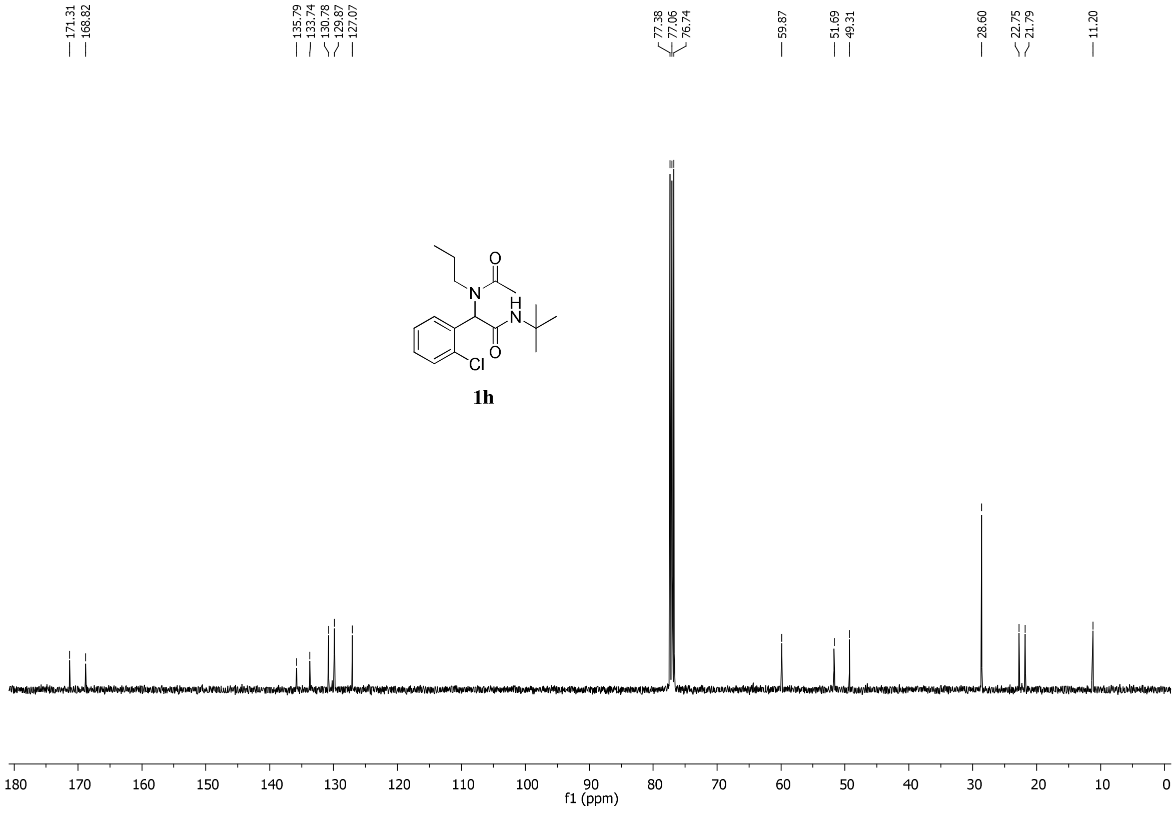
171.31
168.82
135.79
133.74
130.78
129.87
127.07
77.38
77.06
76.74
59.87
51.69
49.31
28.60
22.75
21.79
11.20
O
N
H
N
O
Cl
1h
f1 (ppm)

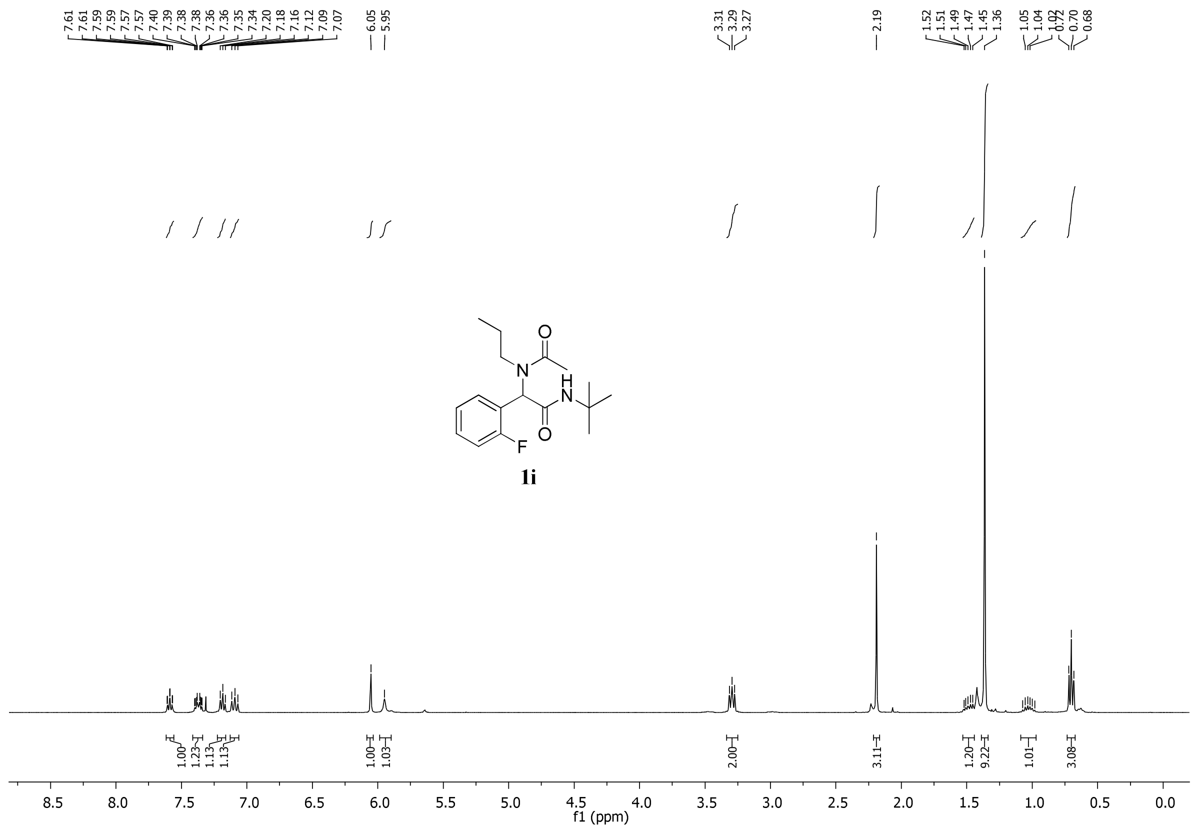
7.61 7.61 7.59 7.59 7.57 7.57 7.40 7.39 7.38 7.38 7.36 7.36 7.35 7.34 7.20 7.18 7.16 7.12 7.09 7.07
6.05 5.95
3.31 3.29 3.27
2.19
1.52 1.51 1.49 1.47 1.45 1.36
1.05 1.04 1.02 0.72 0.70 0.68
1i
1.00 1.23 1.13 1.13 1.00 1.03 2.00 3.11 1.20 9.22 1.01 3.08
f1 (ppm)

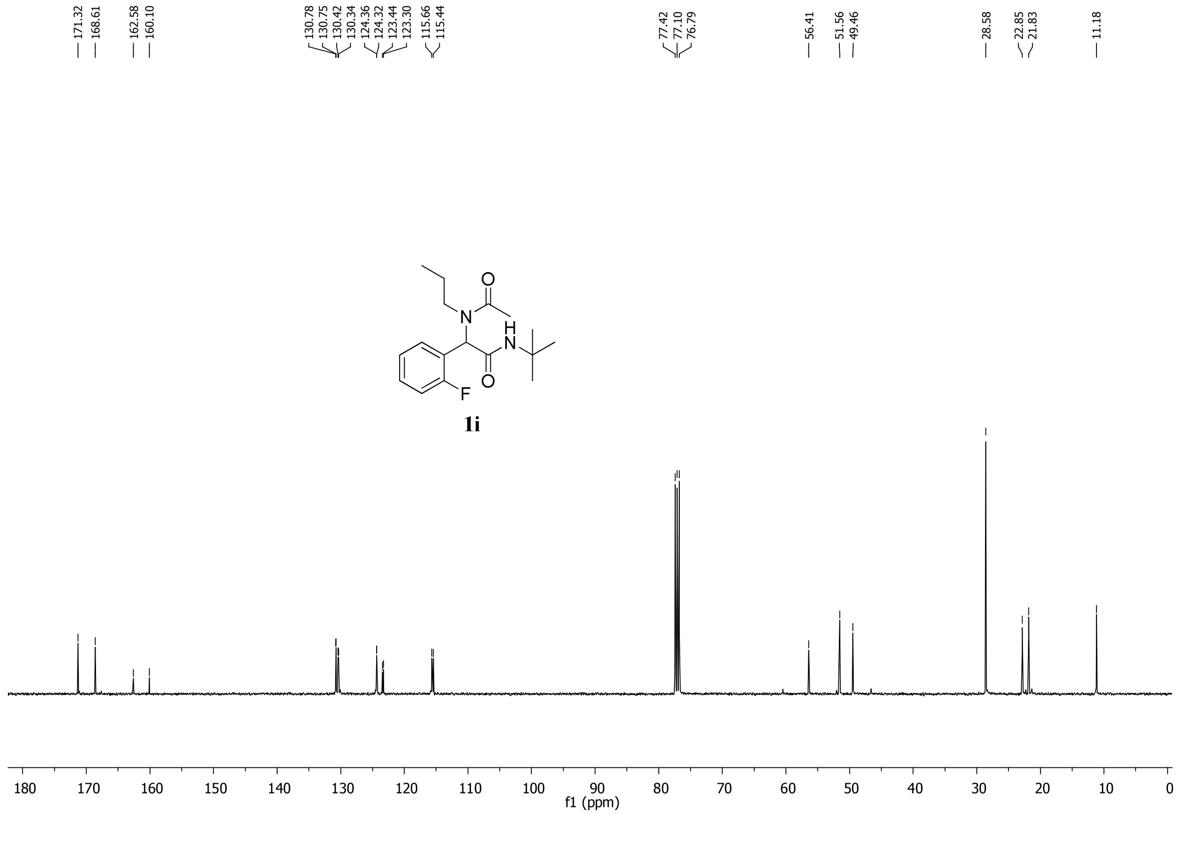
171.32 168.61 162.58 160.10
130.78 130.75 130.42 130.34 124.36 124.32 123.44 123.30 115.66 115.44
77.42 77.10 76.79
56.41 51.56 49.46
28.58 22.85 21.83 11.18
1i
f1 (ppm)

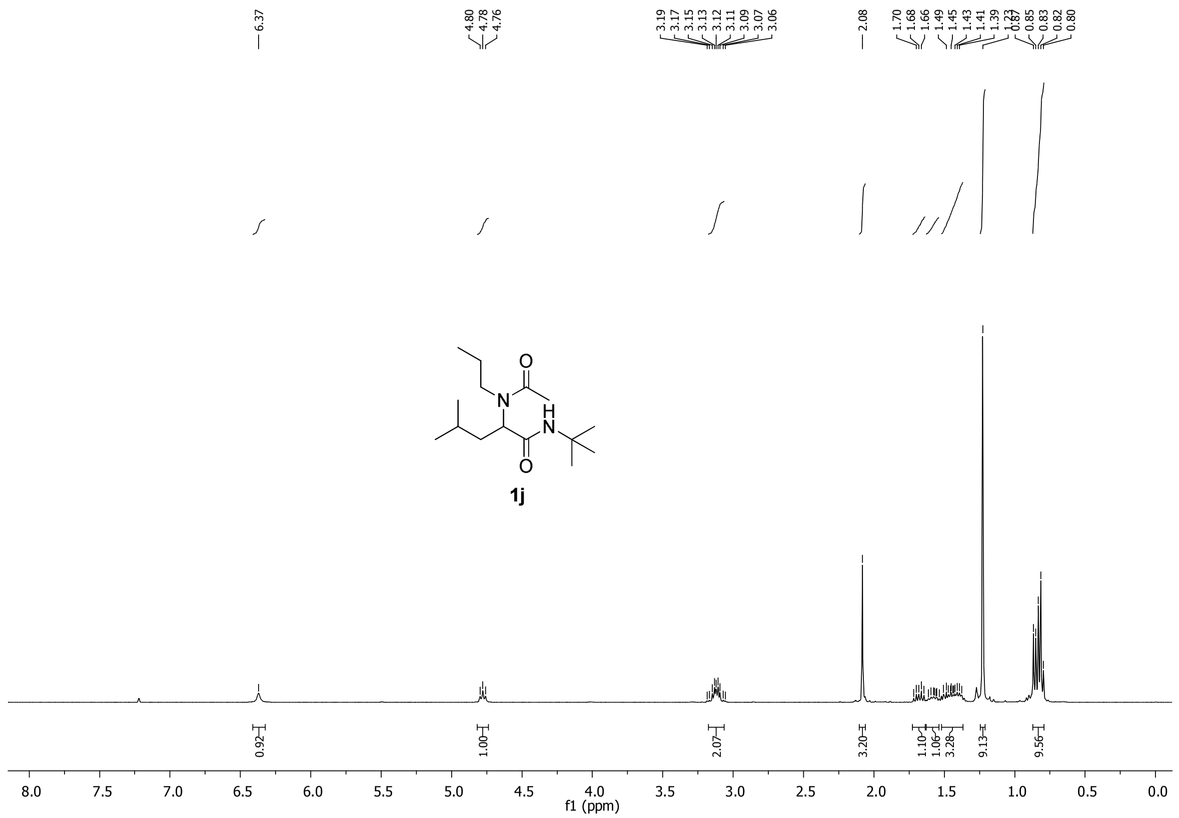

6.37
4.80
4.78
4.76
3.19
3.17
3.15
3.13
3.12
3.11
3.09
3.07
3.06
2.08
1.70
1.68
1.66
1.49
1.45
1.43
1.41
1.39
1.23
0.87
0.85
0.83
0.82
0.80
1j
0.92
1.00
2.07
3.20
1.10
1.06
3.28
9.13
9.56
8.0
7.5
7.0
6.5
6.0
5.5
5.0
4.5
4.0
3.5
3.0
2.5
2.0
1.5
1.0
0.5
0.0
f1 (ppm)

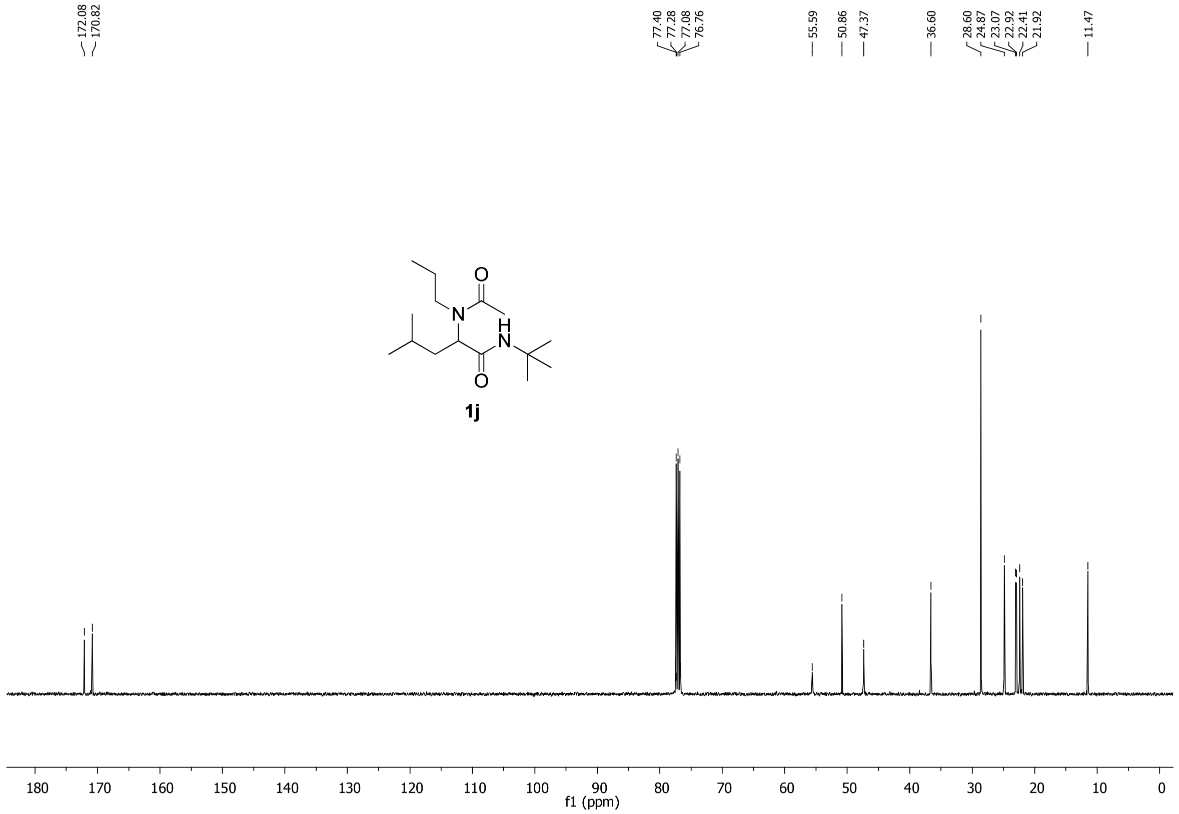

172.08
170.82
77.40
77.28
77.08
76.76
55.59
50.86
47.37
36.60
28.60
24.87
23.07
22.92
22.41
21.92
11.47
1j
180
170
160
150
140
130
120
110
100
90
80
70
60
50
40
30
20
10
0
f1 (ppm)

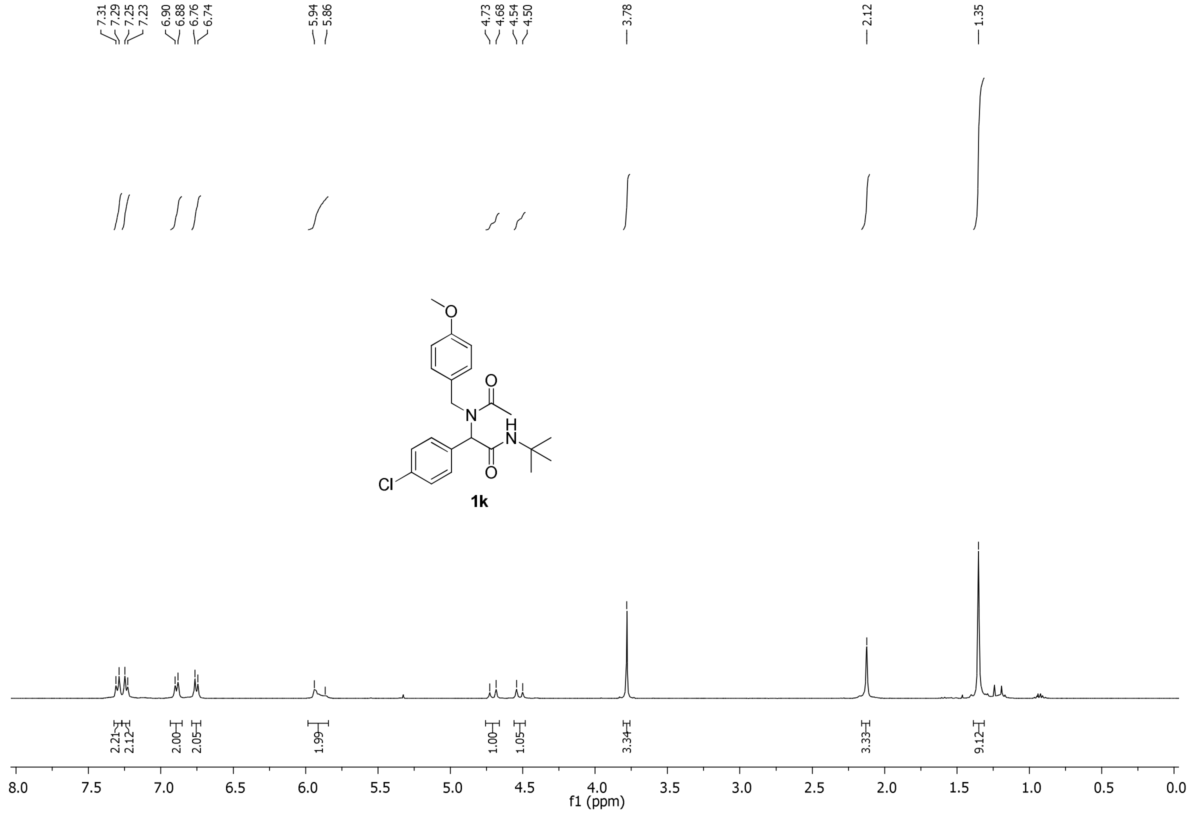
7.31
7.29
7.25
7.23
6.90
6.88
6.76
6.74
5.94
5.86
4.73
4.68
4.54
4.50
3.78
2.12
1.35
Cl
N
O
H
N
O
O
1k
2.21
2.12
2.00
2.05
1.99
1.00
1.05
3.34
3.33
9.12
f1 (ppm)

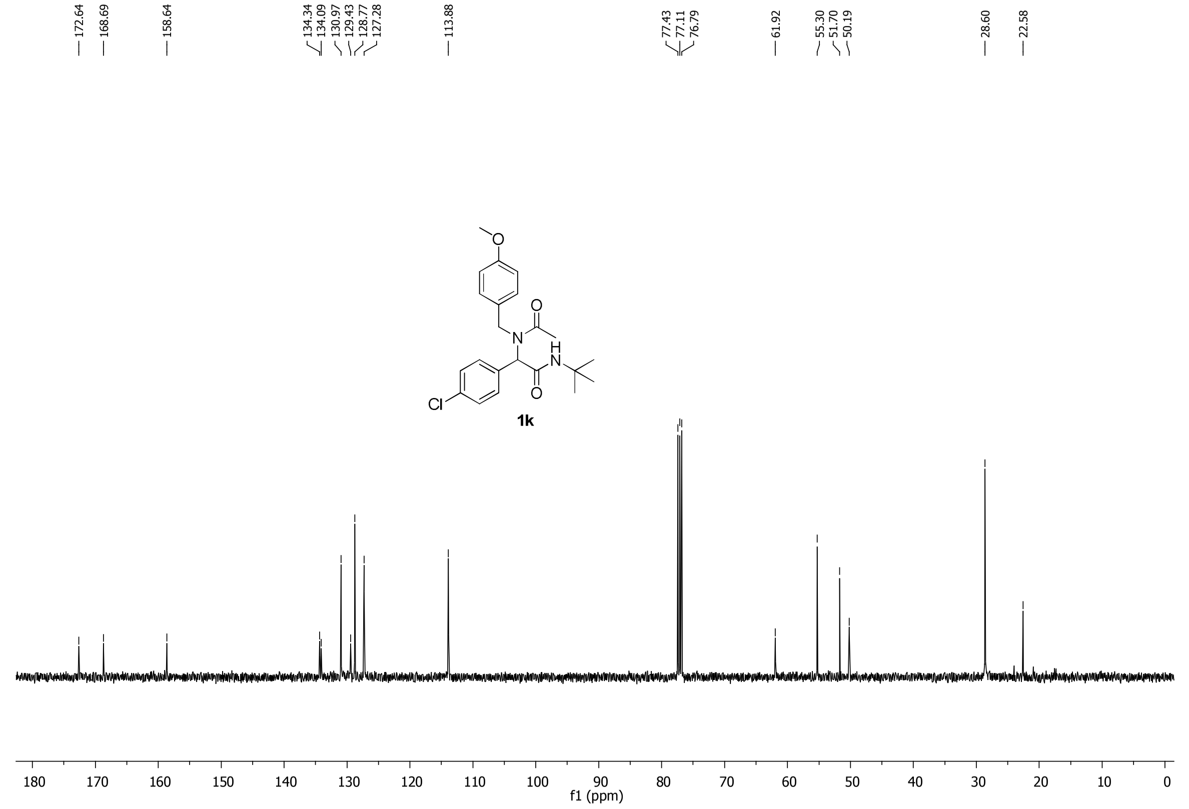
172.64
168.69
158.64
134.34
134.09
130.97
129.43
128.77
127.28
113.88
77.43
77.11
76.79
61.92
55.30
51.70
50.19
28.60
22.58
Cl
N
O
H
N
O
O
1k
f1 (ppm)

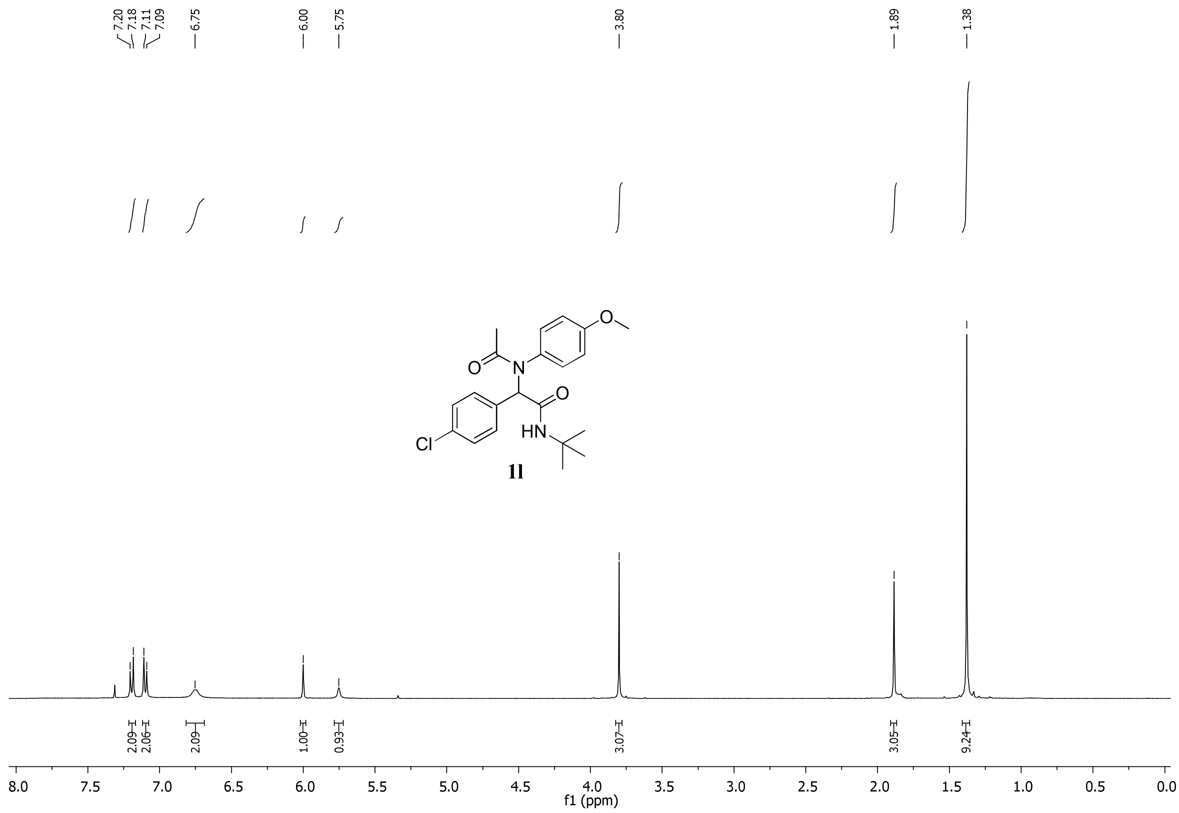

1l
f1 (ppm)

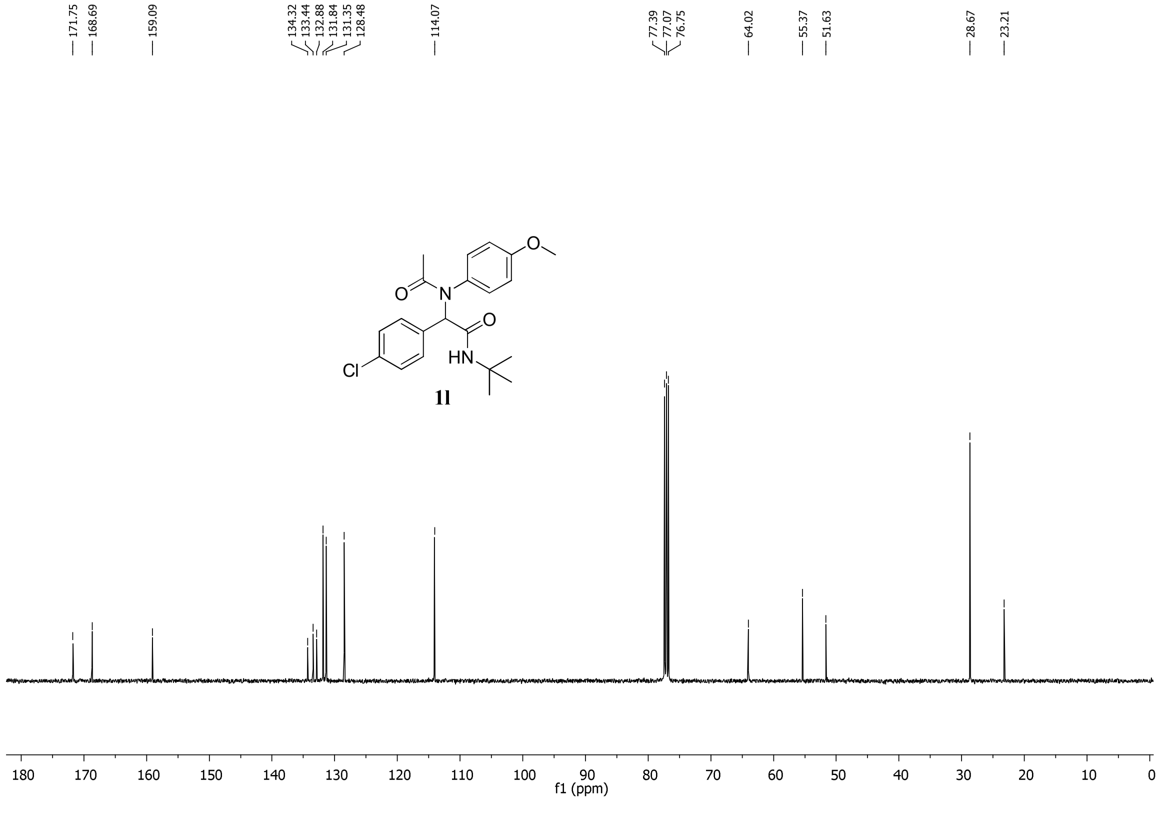

1l
f1 (ppm)

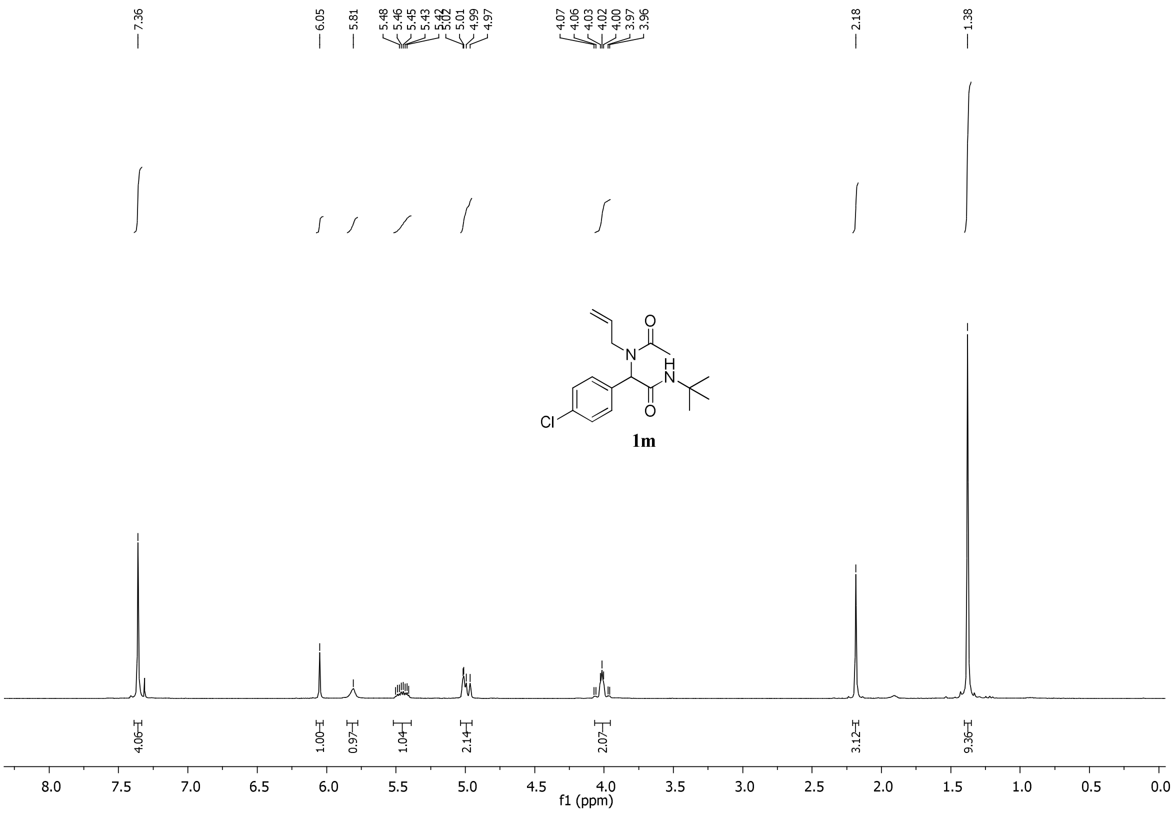
7.36
6.05
5.81
5.48
5.46
5.45
5.43
5.42
5.02
5.01
4.99
4.97
4.07
4.06
4.03
4.02
4.00
3.97
3.96
2.18
1.38
Cl
N
O
H
1m
4.06
1.00
0.97
1.04
2.14
2.07
3.12
9.36
f1 (ppm)

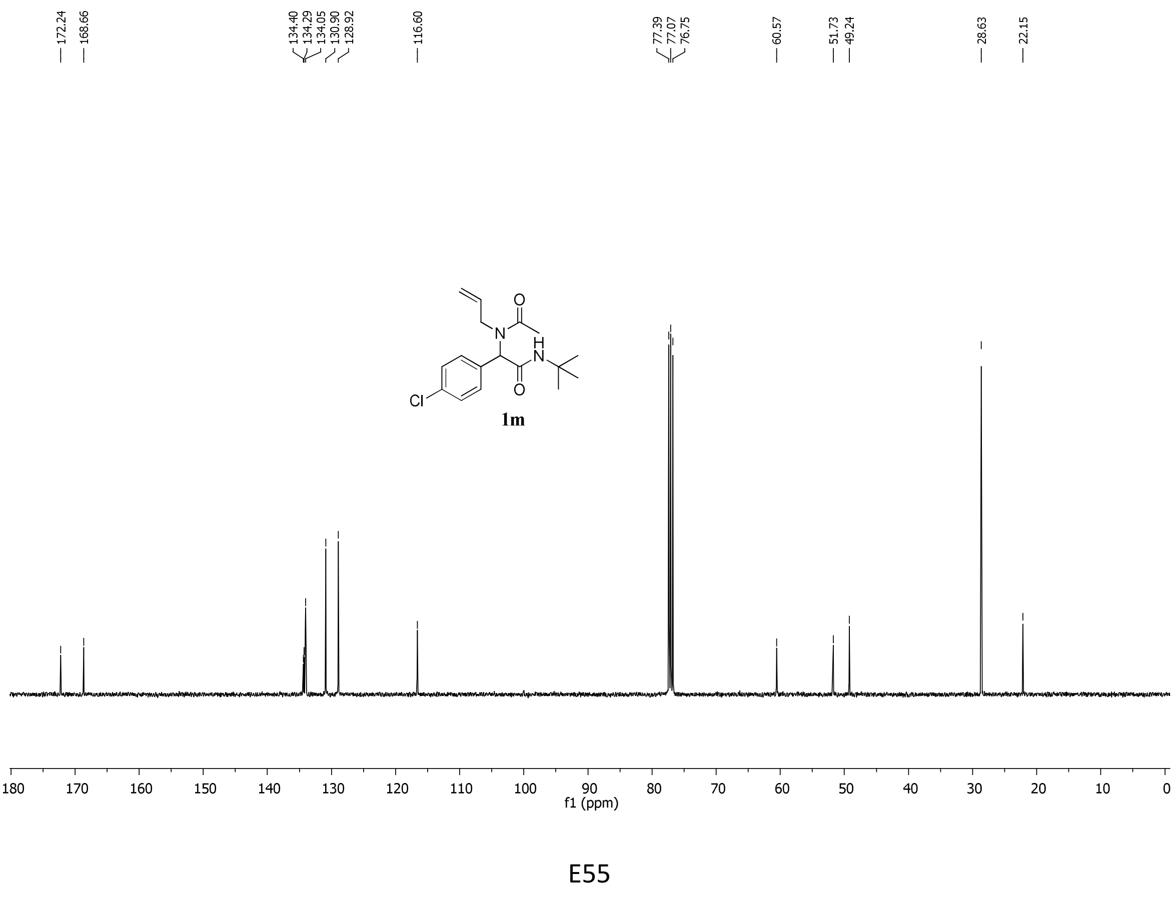
172.24
168.66
134.40
134.29
134.05
130.90
128.92
116.60
77.39
77.07
76.75
60.57
51.73
49.24
28.63
22.15
Cl
N
O
H
1m
f1 (ppm)

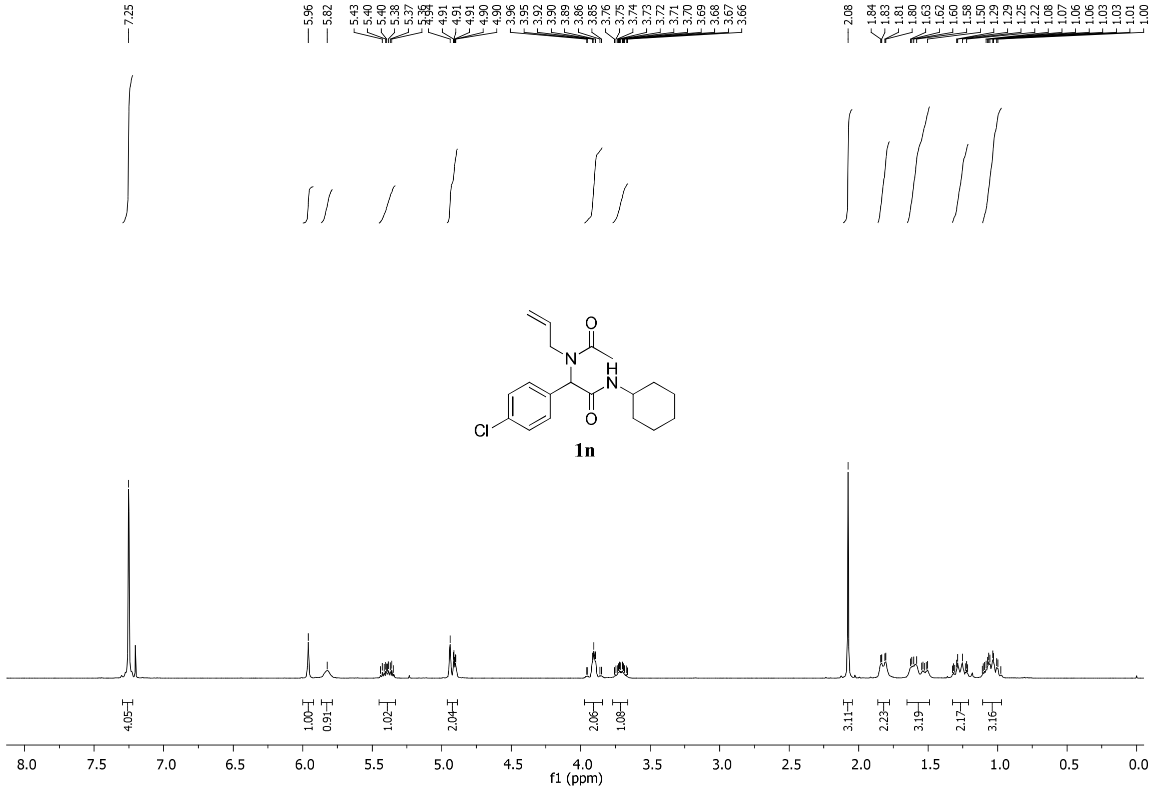
7.25
5.96
5.82
5.43
5.40
5.40
5.38
5.37
5.36
4.94
4.91
4.91
4.91
4.90
4.90
3.96
3.95
3.92
3.90
3.89
3.86
3.85
3.76
3.75
3.74
3.73
3.72
3.71
3.70
3.69
3.68
3.67
3.66
2.08
1.84
1.83
1.81
1.80
1.63
1.62
1.60
1.58
1.50
1.29
1.25
1.22
1.08
1.07
1.06
1.06
1.03
1.03
1.01
1.00
O
N
H
N
O
Cl
1n
4.05
1.00
0.91
1.02
2.04
2.06
1.08
3.11
2.23
3.19
2.17
3.16
8.0
7.5
7.0
6.5
6.0
5.5
5.0
4.5
4.0
3.5
3.0
2.5
2.0
1.5
1.0
0.5
0.0
f1 (ppm)

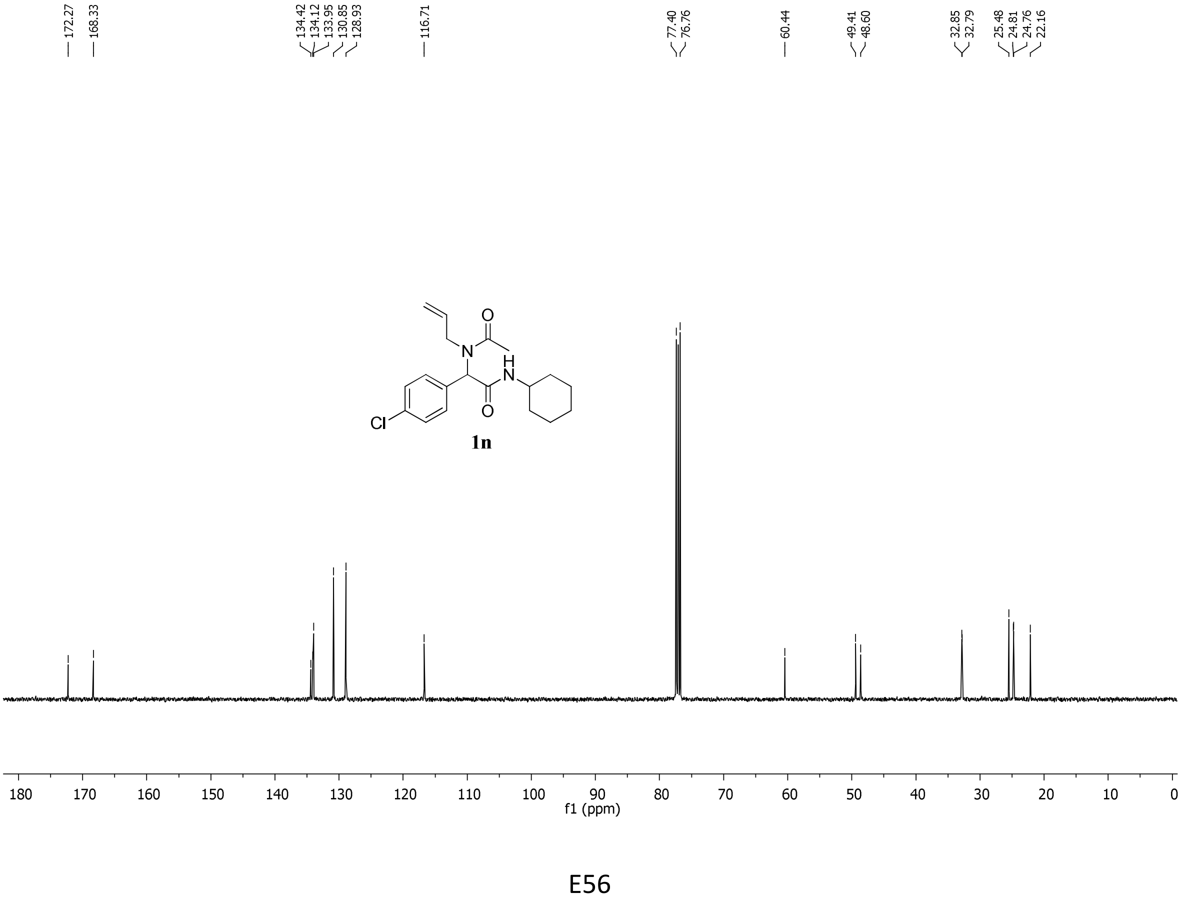
172.27
168.33
134.42
134.12
133.95
130.85
128.93
116.71
77.40
76.76
60.44
49.41
48.60
32.85
32.79
25.48
24.81
24.76
22.16
O
N
H
N
O
Cl
1n
180
170
160
150
140
130
120
110
100
90
80
70
60
50
40
30
20
10
0
f1 (ppm)

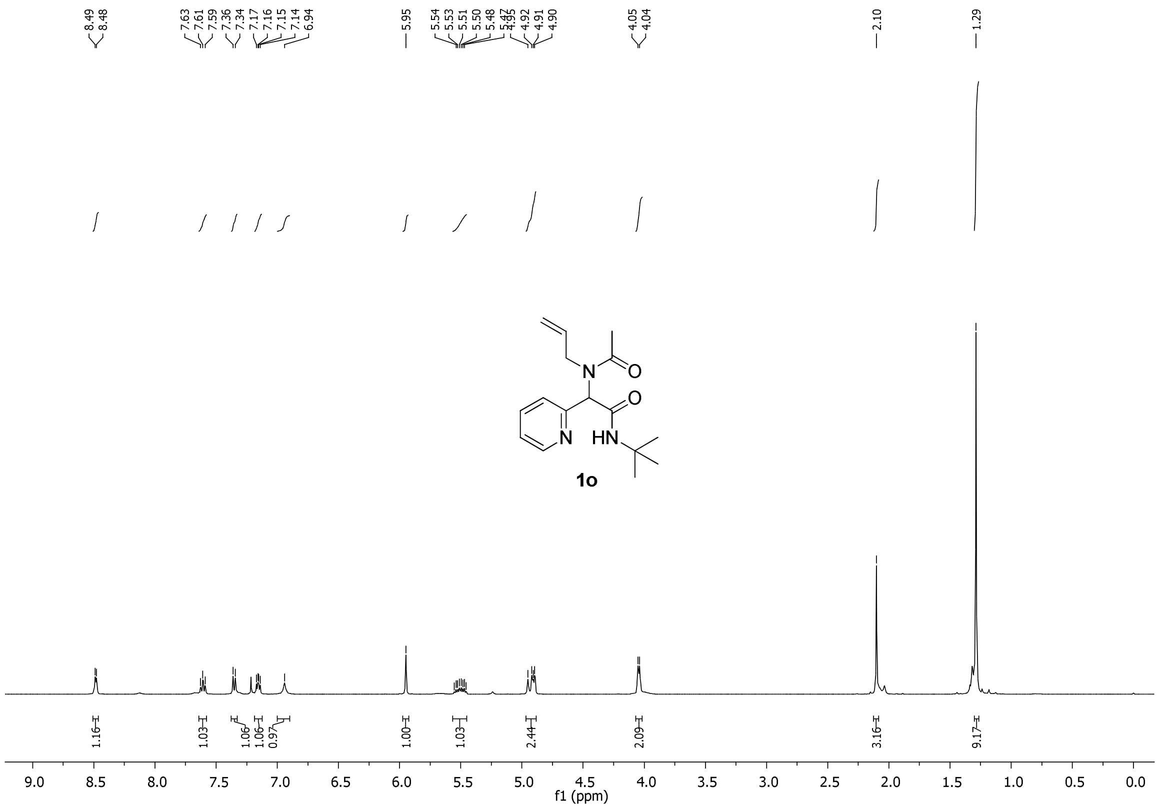
8.49
8.48
7.63
7.61
7.59
7.36
7.34
7.17
7.16
7.15
7.14
6.94
5.95
5.54
5.53
5.51
5.50
5.48
5.47
4.95
4.92
4.91
4.90
4.05
4.04
2.10
1.29
N
HN
N
O
O
1o
1.16
1.03
1.06
1.06
0.97
1.00
1.03
2.44
2.09
3.16
9.17
f1 (ppm)

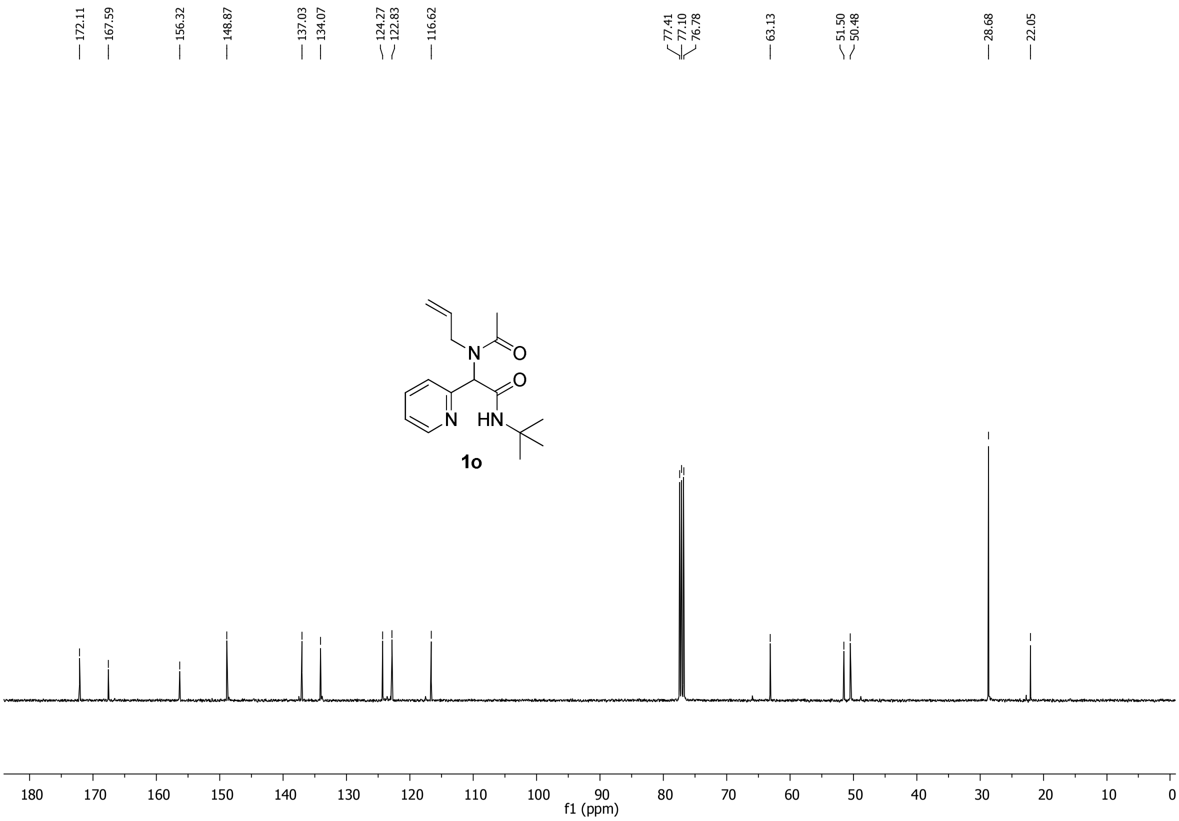
172.11
167.59
156.32
148.87
137.03
134.07
124.27
122.83
116.62
77.41
77.10
76.78
63.13
51.50
50.48
28.68
22.05
N
HN
N
O
O
1o
f1 (ppm)

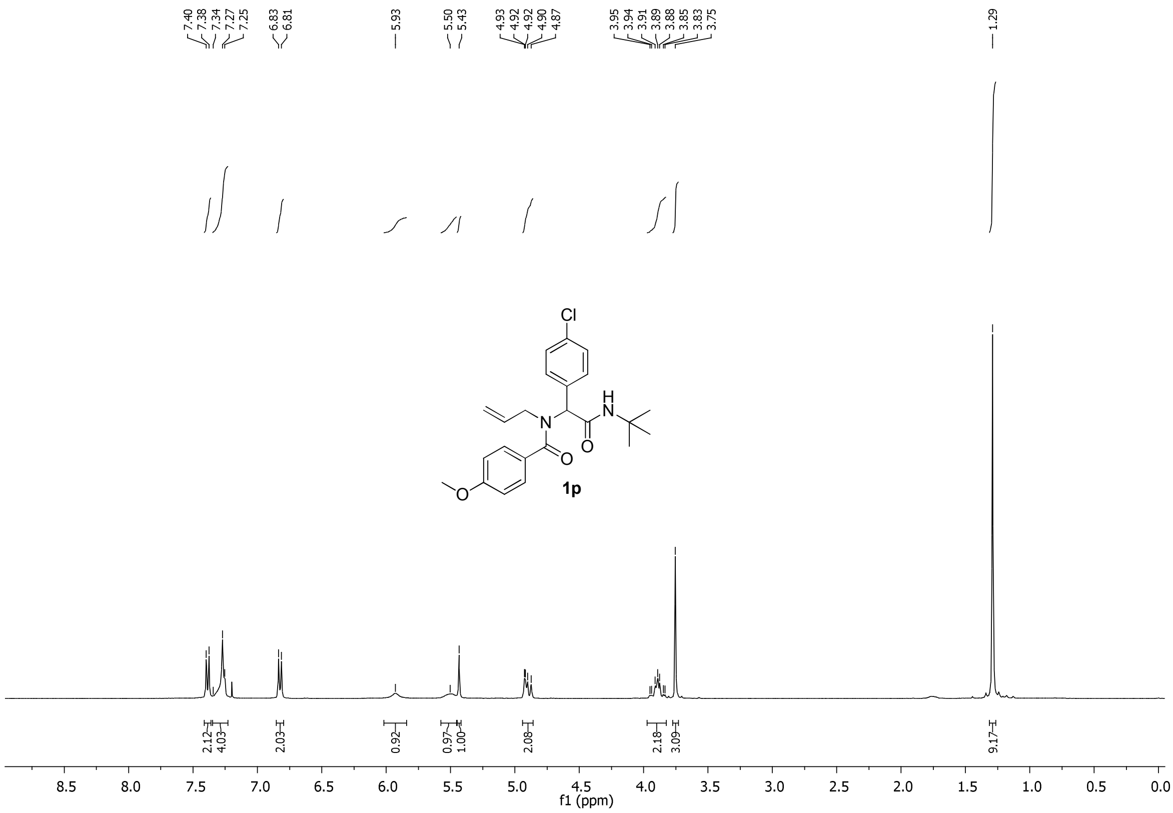
7.40
7.38
7.34
7.27
7.25
6.83
6.81
5.93
5.50
5.43
4.93
4.92
4.92
4.90
4.87
3.95
3.94
3.91
3.89
3.88
3.85
3.83
3.75
1.29
Cl
H
N
N
O
O
O
1p
2.12
4.03
2.03
0.92
0.97
1.00
2.08
2.18
3.09
9.17
f1 (ppm)

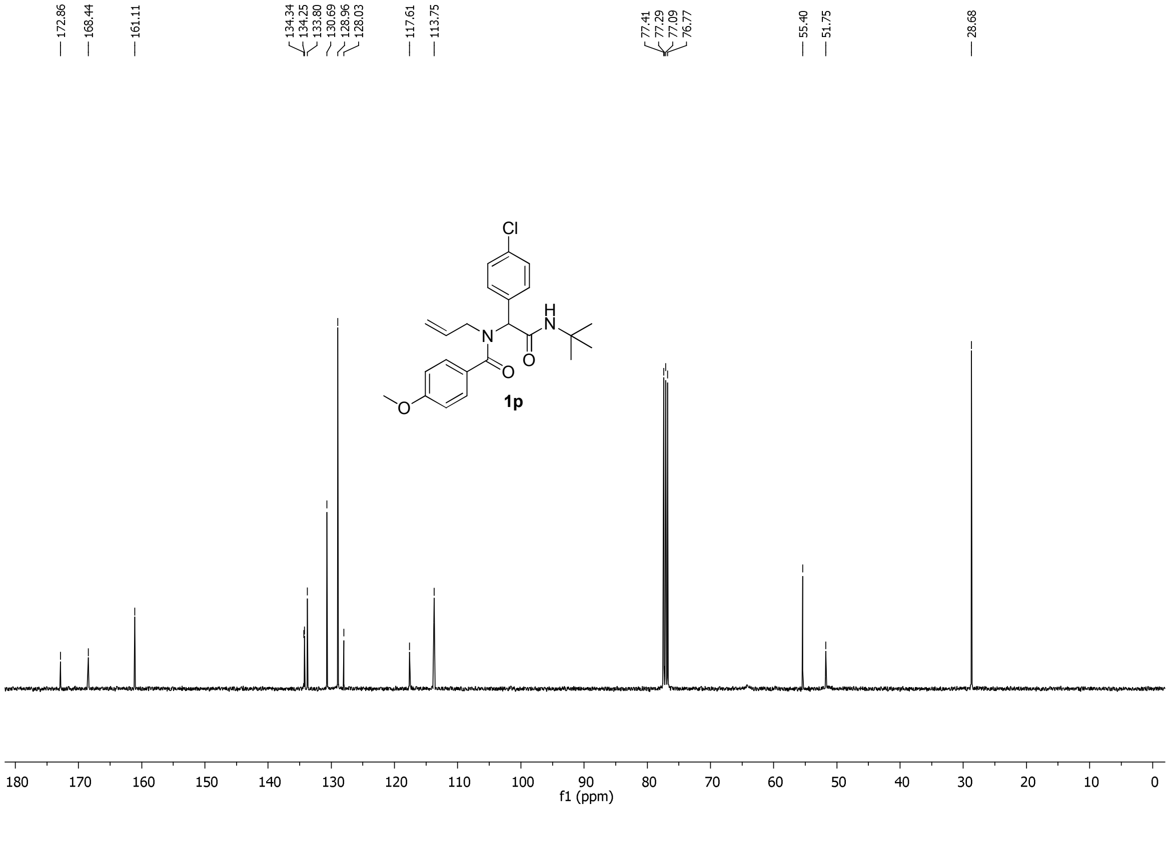
172.86
168.44
161.11
134.34
134.25
133.80
130.69
128.96
128.03
117.61
113.75
77.41
77.29
77.09
76.77
55.40
51.75
28.68
Cl
H
N
N
O
O
O
1p
f1 (ppm)

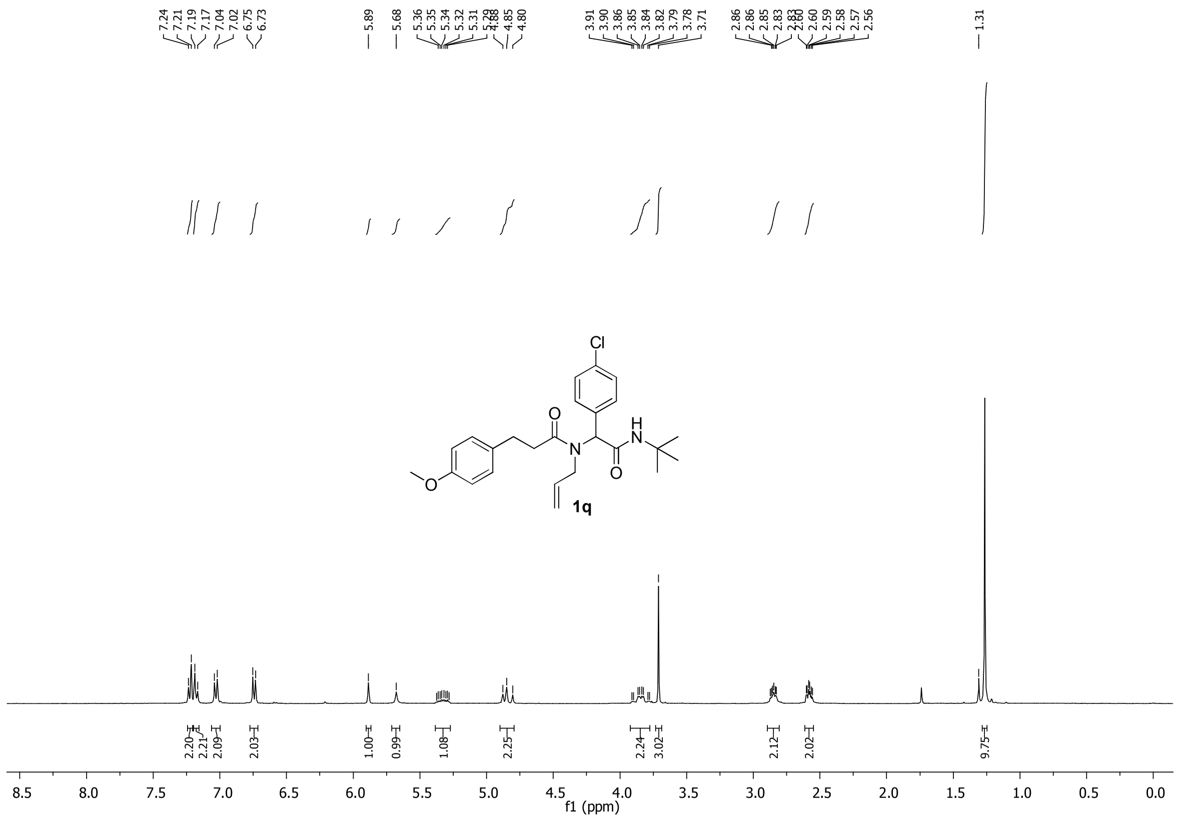

7.24
7.21
7.19
7.17
7.04
7.02
6.75
6.73
5.89
5.68
5.36
5.35
5.34
5.32
5.31
5.29
4.88
4.85
4.80
3.91
3.90
3.86
3.85
3.84
3.82
3.79
3.78
3.71
2.86
2.86
2.85
2.83
2.83
2.60
2.60
2.59
2.58
2.57
2.56
1.31
Cl
O
N
H
N
O
O
1q
2.20
2.21
2.09
2.03
1.00
0.99
1.08
2.25
2.24
3.02
2.12
2.02
9.75
8.5
8.0
7.5
7.0
6.5
6.0
5.5
5.0
4.5
4.0
3.5
3.0
2.5
2.0
1.5
1.0
0.5
0.0
f1 (ppm)

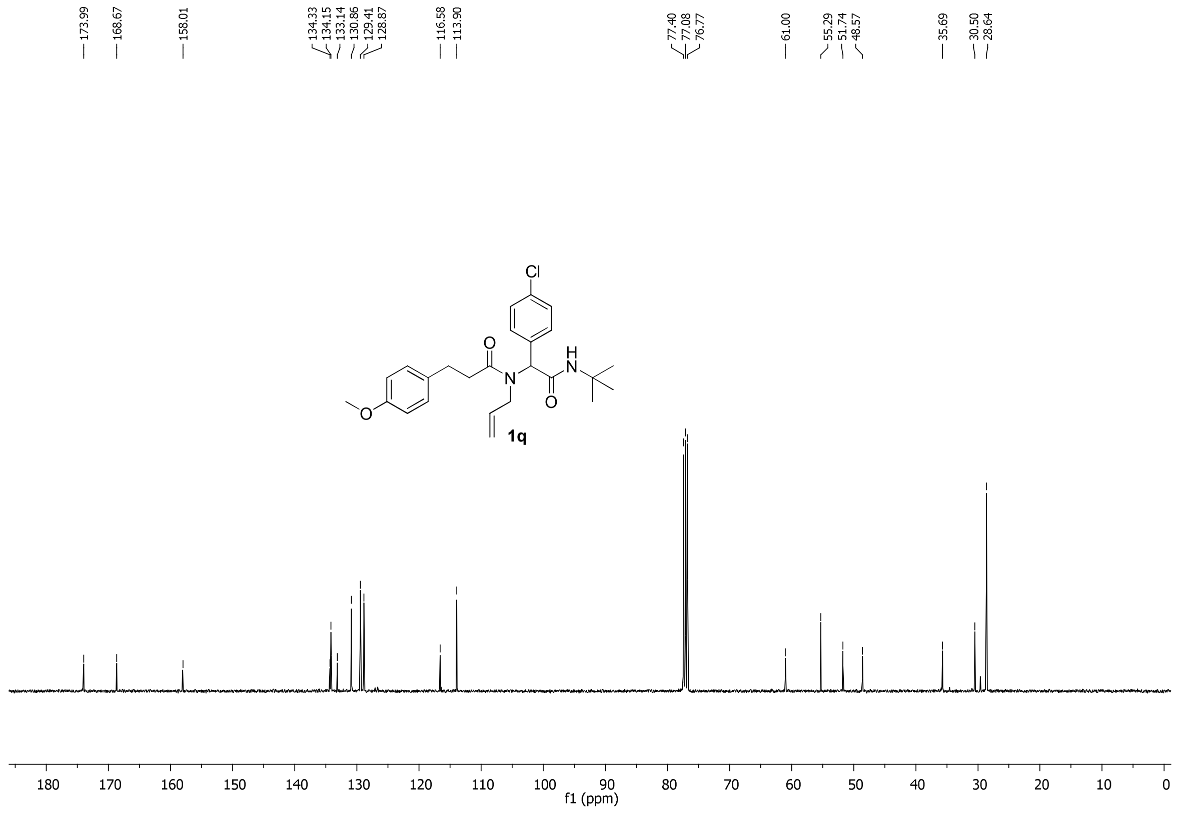

173.99
168.67
158.01
134.33
134.15
133.14
130.86
129.41
128.87
116.58
113.90
77.40
77.08
76.77
61.00
55.29
51.74
48.57
35.69
30.50
28.64
Cl
O
N
H
N
O
O
1q
180
170
160
150
140
130
120
110
100
90
80
70
60
50
40
30
20
10
0
f1 (ppm)

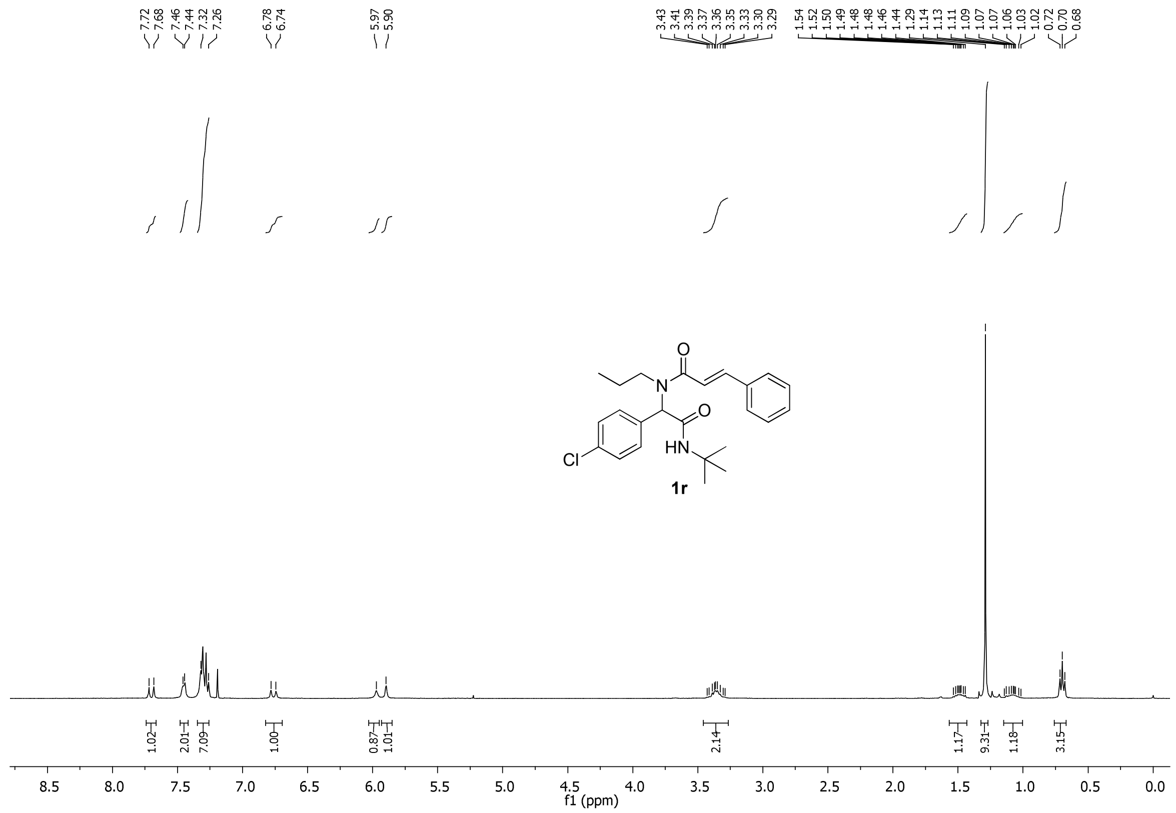
7.72
7.68
7.46
7.44
7.32
7.26
6.78
6.74
5.97
5.90
3.43
3.41
3.39
3.37
3.36
3.35
3.33
3.30
3.29
1.54
1.52
1.50
1.49
1.48
1.48
1.48
1.46
1.44
1.29
1.14
1.13
1.11
1.09
1.07
1.07
1.06
1.03
1.02
0.72
0.70
0.68
Cl
N
O
O
HN
1r
1.02
2.01
7.09
1.00
0.87
1.01
2.14
1.17
9.31
1.18
3.15
f1 (ppm)

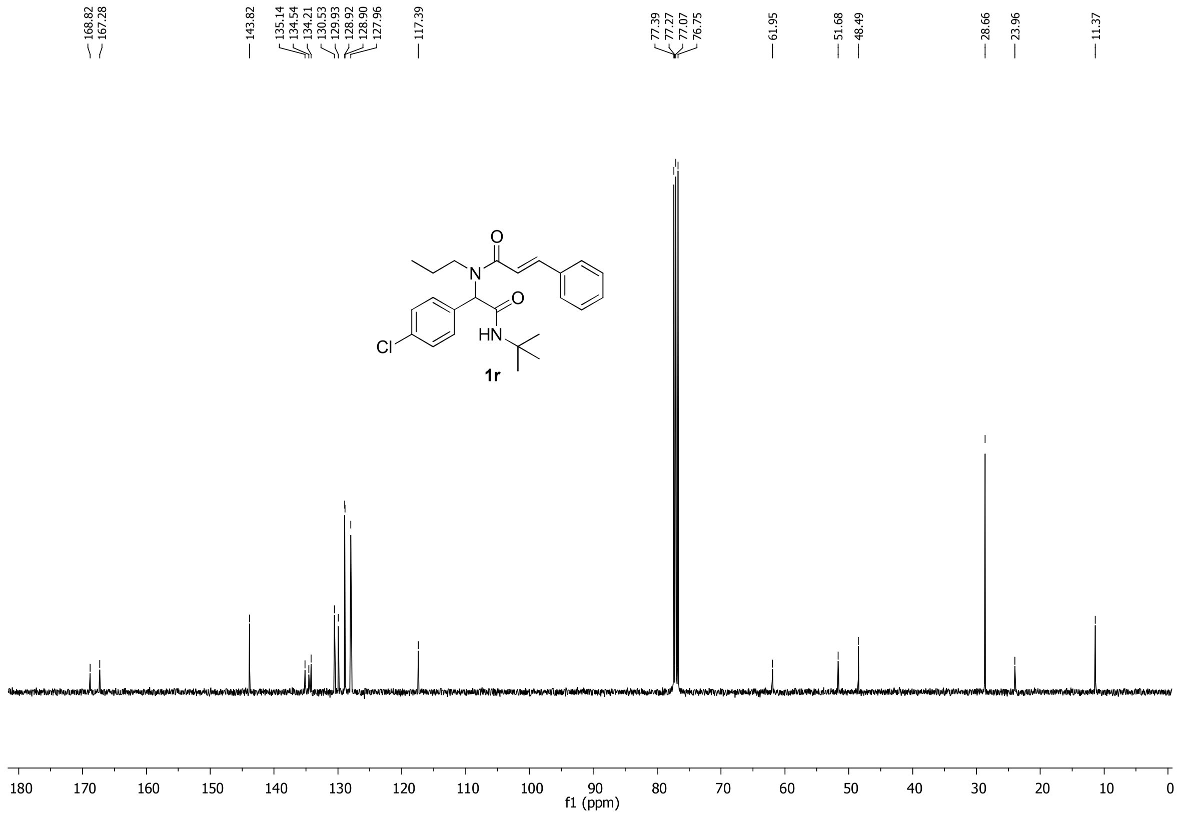
168.82
167.28
143.82
135.14
134.54
134.21
130.53
129.93
128.92
128.90
127.96
117.39
77.39
77.27
77.07
76.75
61.95
51.68
48.49
28.66
23.96
11.37
Cl
N
O
O
HN
1r
f1 (ppm)

E60

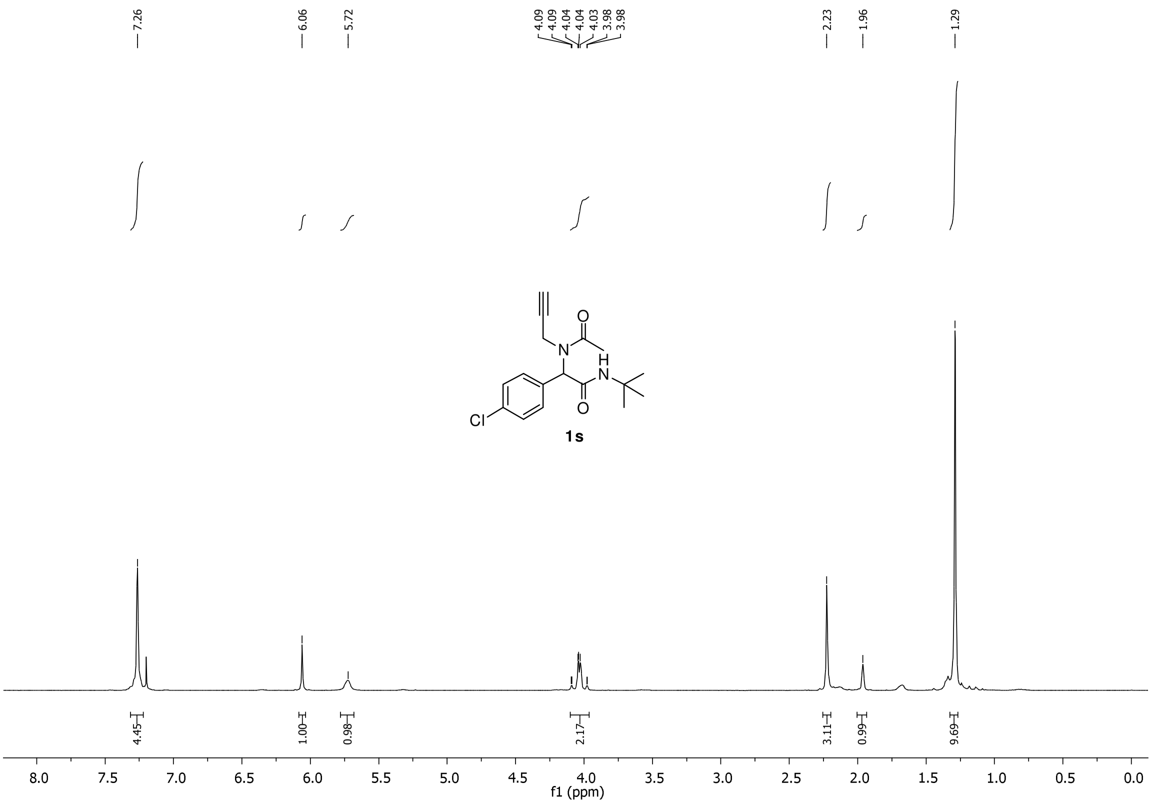

7.26
6.06
5.72
4.09
4.09
4.04
4.04
4.03
3.98
3.98
2.23
1.96
1.29
Cl
N
O
H
N
O
1s
4.45
1.00
0.98
2.17
3.11
0.99
9.69
8.0
7.5
7.0
6.5
6.0
5.5
5.0
4.5
4.0
3.5
3.0
2.5
2.0
1.5
1.0
0.5
0.0
f1 (ppm)

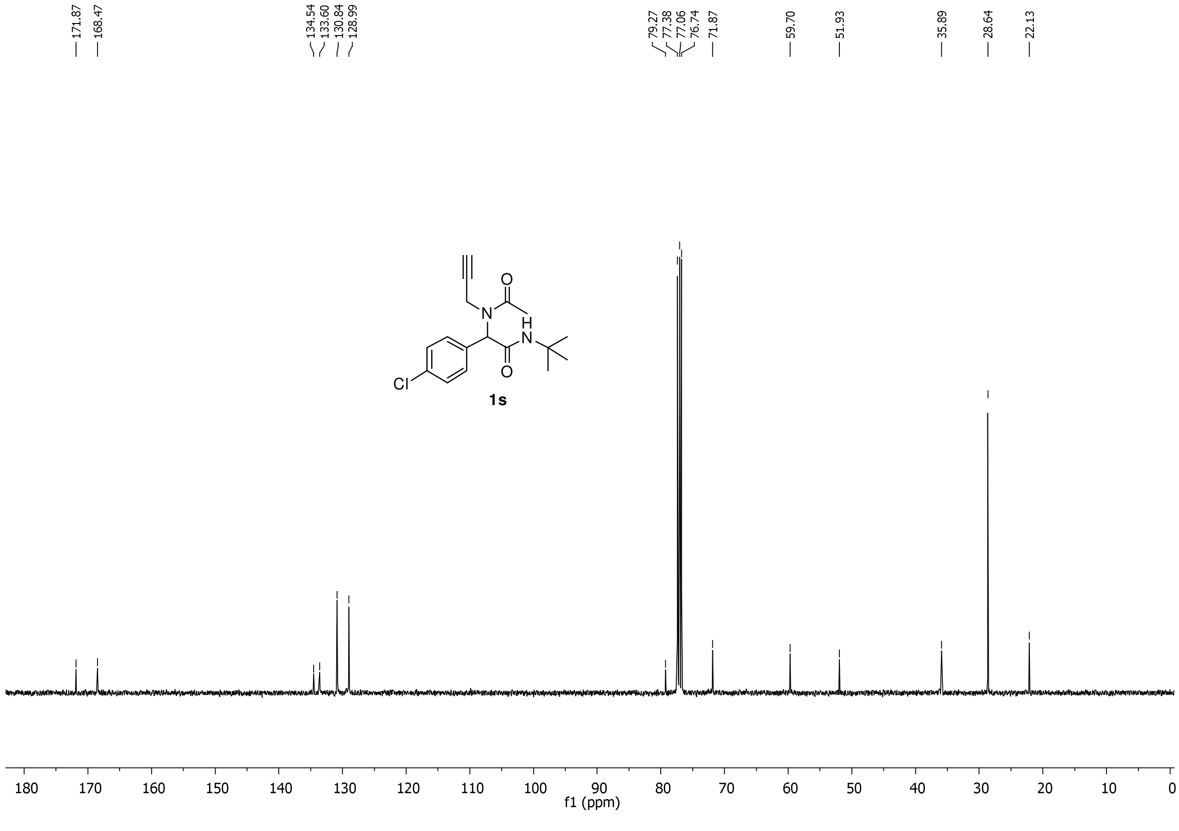

171.87
168.47
134.54
133.60
130.84
128.99
79.27
77.38
77.06
76.74
71.87
59.70
51.93
35.89
28.64
22.13
Cl
N
O
H
N
O
1s
180
170
160
150
140
130
120
110
100
90
80
70
60
50
40
30
20
10
0
f1 (ppm)

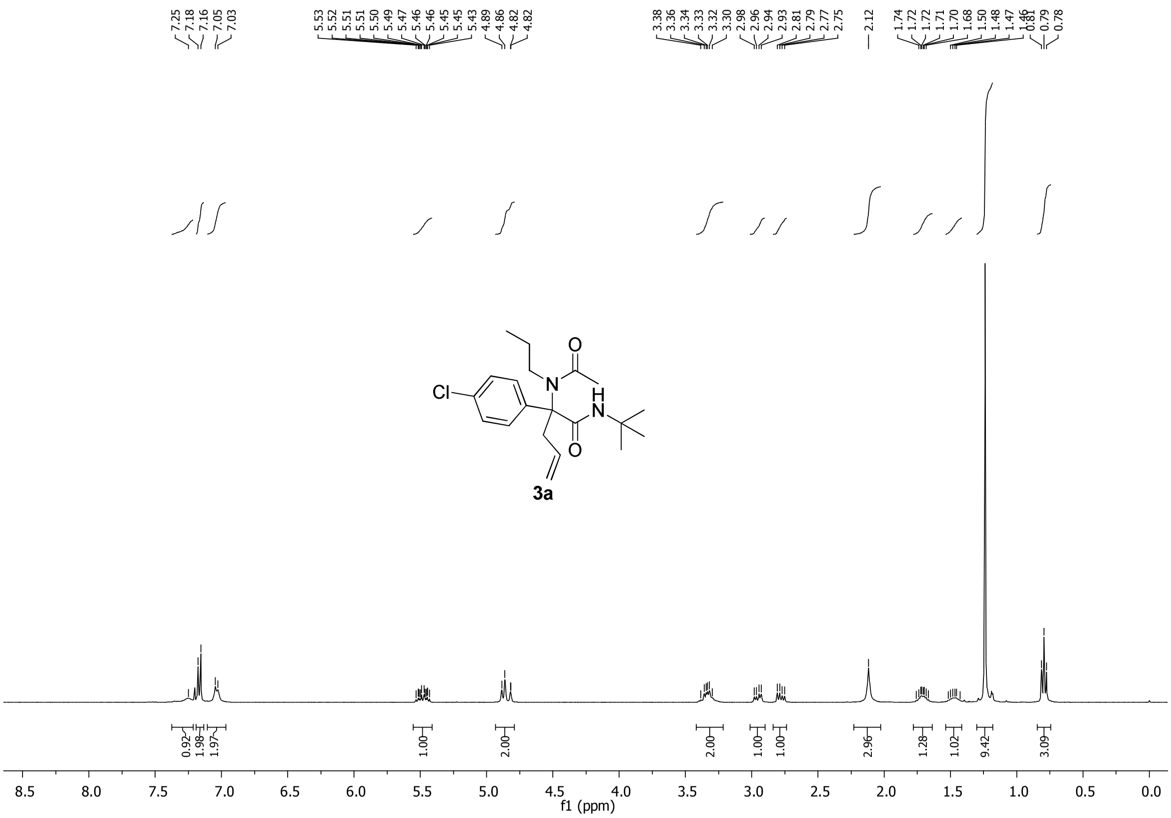
7.25
7.18
7.16
7.05
7.03
5.53
5.52
5.51
5.51
5.50
5.49
5.47
5.46
5.46
5.45
5.45
5.43
4.89
4.86
4.82
4.82
3.38
3.36
3.34
3.33
3.32
3.30
2.98
2.96
2.94
2.93
2.81
2.79
2.77
2.75
2.12
1.74
1.72
1.72
1.71
1.70
1.68
1.50
1.48
1.47
1.46
0.81
0.79
0.78
Cl
N
H
N
O
O
3a
0.92
1.98
1.97
1.00
2.00
2.00
1.00
1.00
2.96
1.28
1.02
9.42
3.09
8.5
8.0
7.5
7.0
6.5
6.0
5.5
5.0
4.5
4.0
3.5
3.0
2.5
2.0
1.5
1.0
0.5
0.0
f1 (ppm)

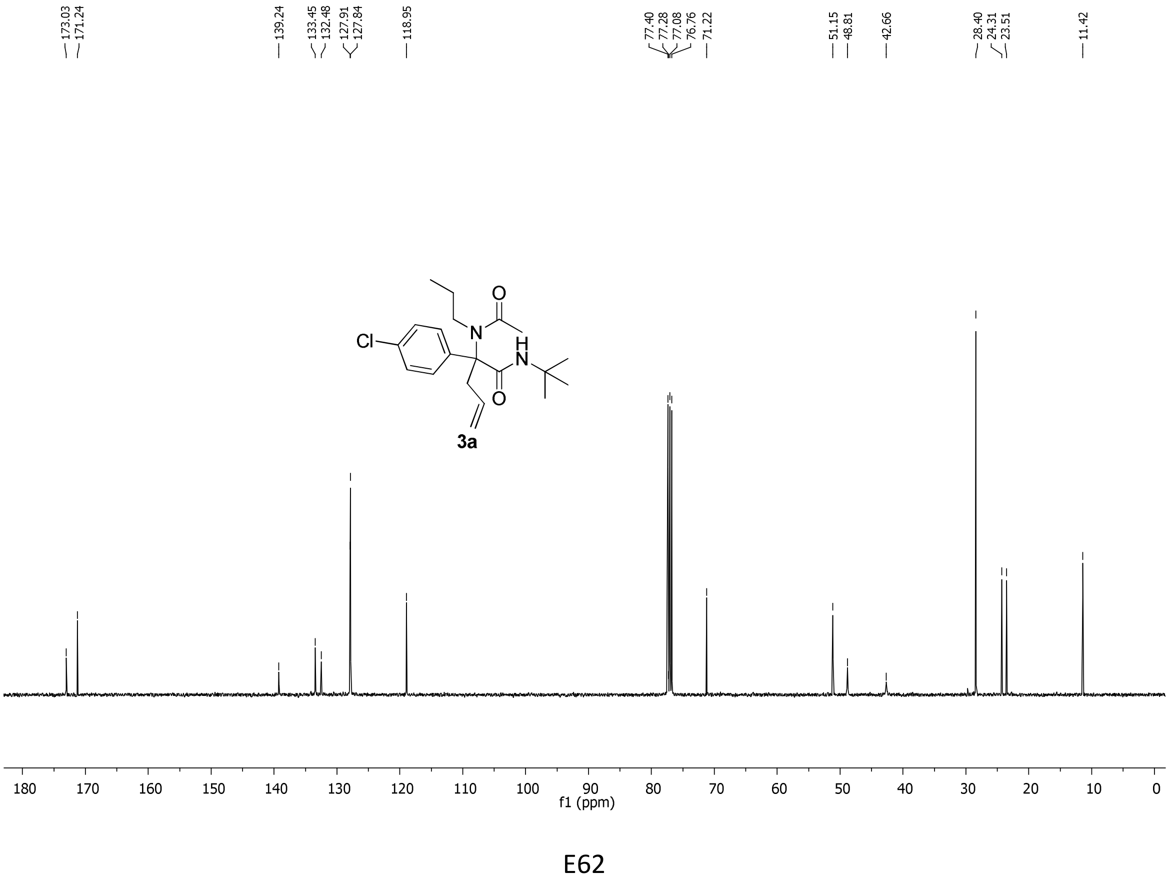
173.03
171.24
139.24
133.45
132.48
127.91
127.84
118.95
77.40
77.28
77.08
76.76
71.22
51.15
48.81
42.66
28.40
24.31
23.51
11.42
Cl
N
H
N
O
O
3a
180
170
160
150
140
130
120
110
100
90
80
70
60
50
40
30
20
10
0
f1 (ppm)

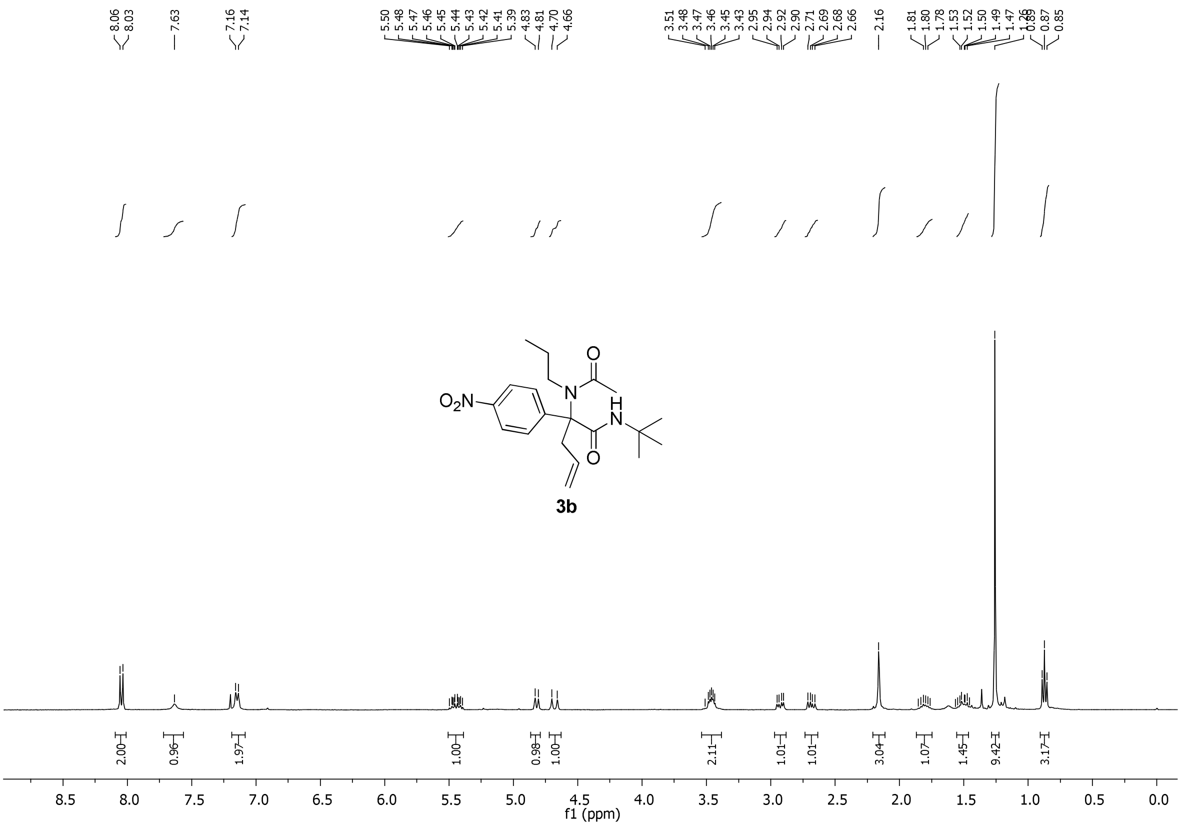
3b
f1 (ppm)

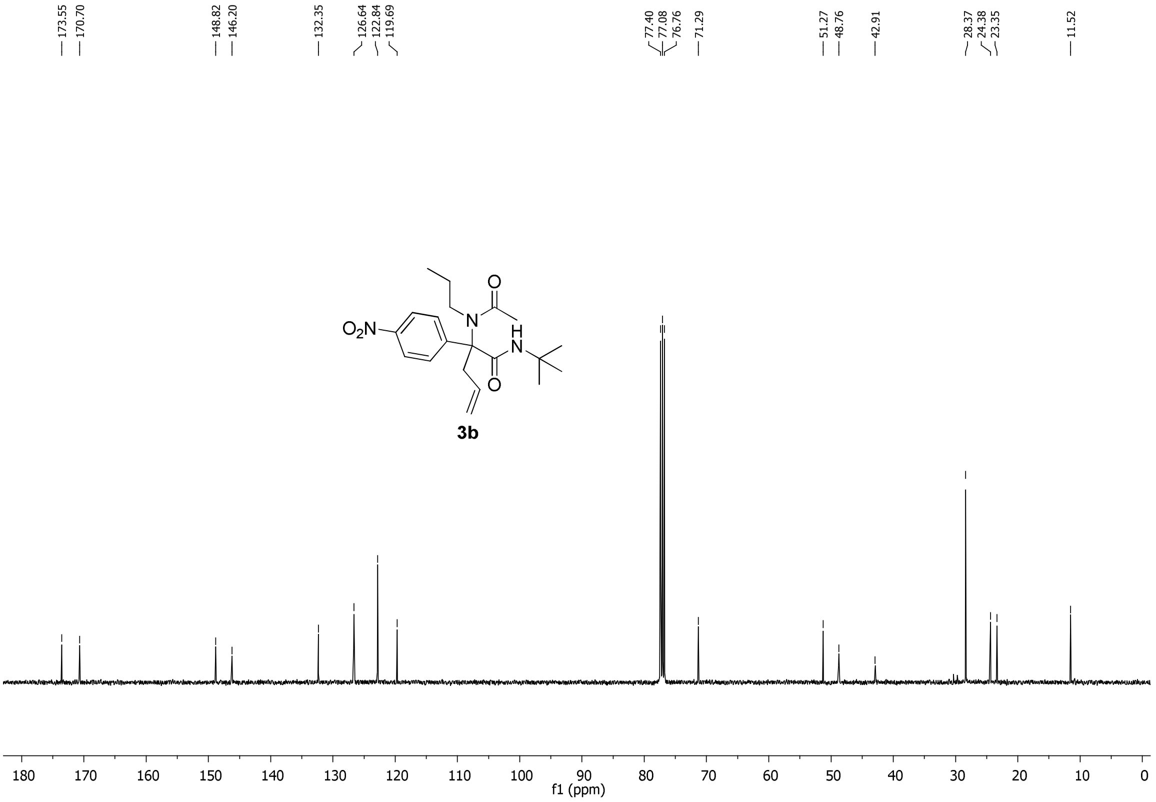
3b
f1 (ppm)

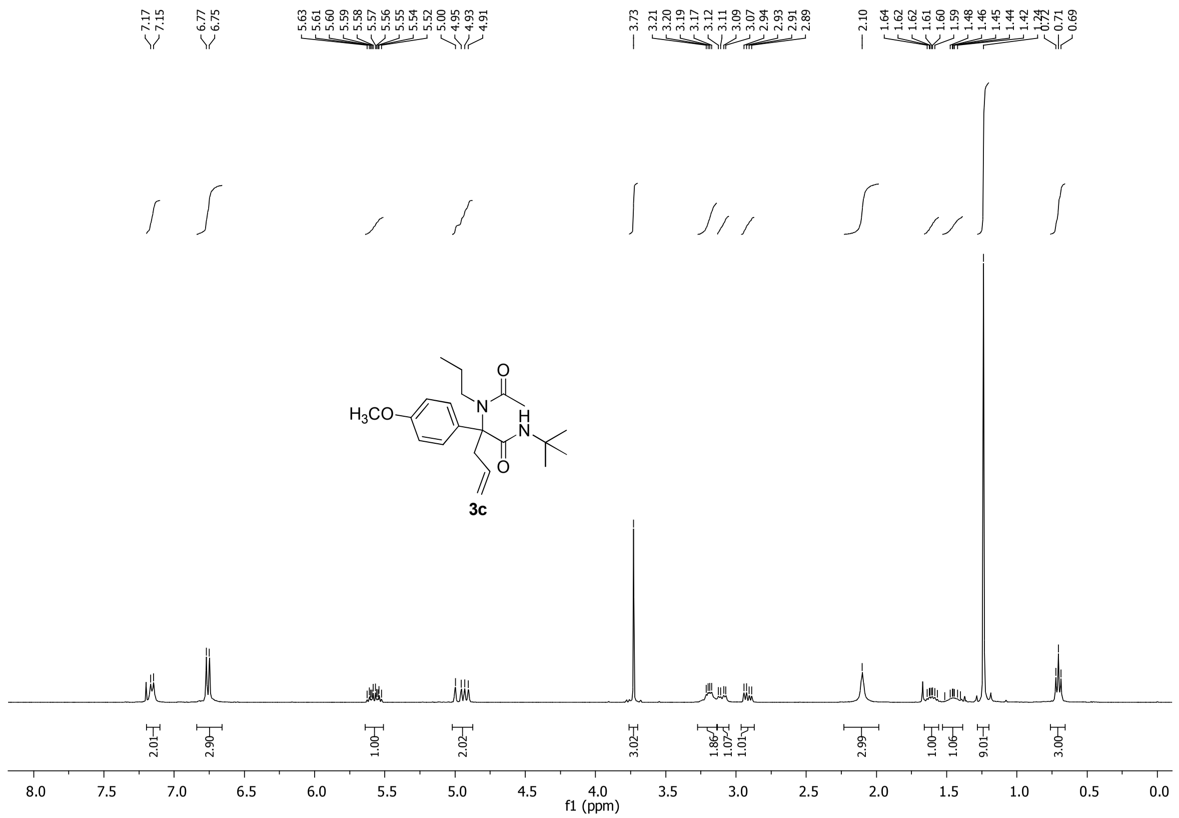
7.17
7.15
6.77
6.75
5.63
5.61
5.60
5.59
5.58
5.57
5.56
5.55
5.54
5.52
5.00
4.95
4.93
4.91
3.73
3.21
3.20
3.19
3.17
3.12
3.11
3.09
3.07
2.94
2.93
2.91
2.89
2.10
1.64
1.62
1.62
1.61
1.60
1.59
1.48
1.46
1.45
1.44
1.42
1.24
0.72
0.71
0.69
H_3CO
N
H
O
3c
2.01
2.90
1.00
2.02
3.02
1.86
1.07
1.01
2.99
1.00
1.06
9.01
3.00
8.0
7.5
7.0
6.5
6.0
5.5
5.0
4.5
4.0
3.5
3.0
2.5
2.0
1.5
1.0
0.5
0.0
f1 (ppm)

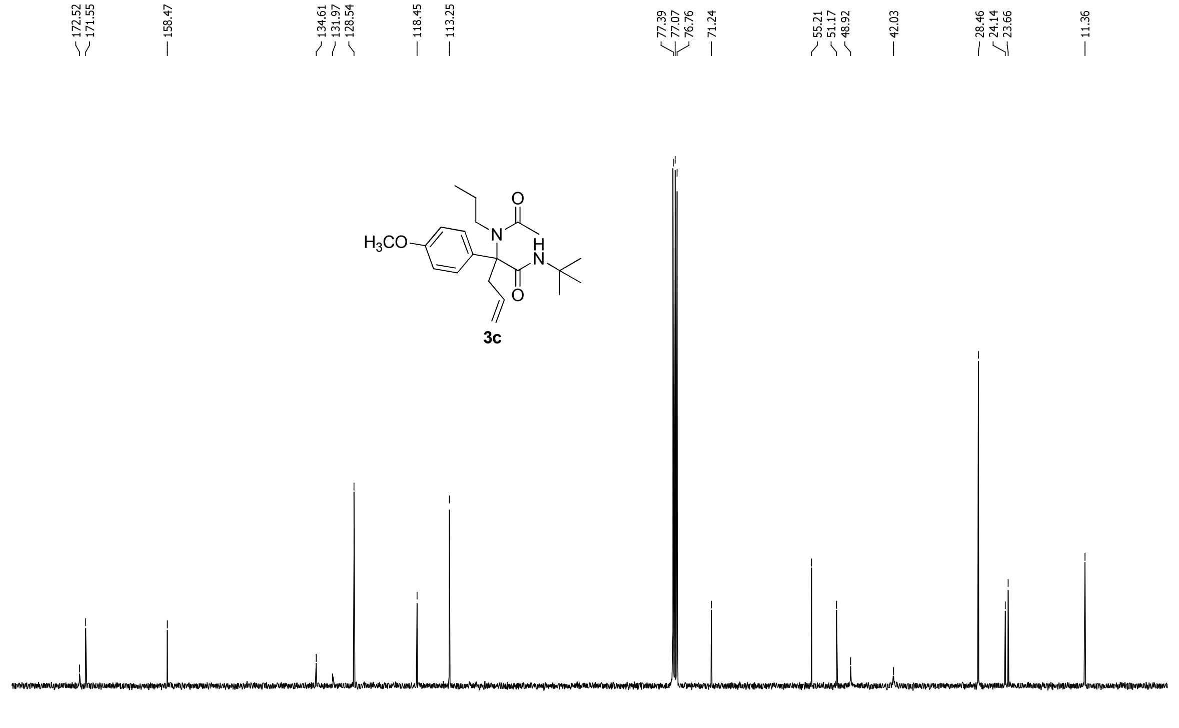
172.52
171.55
158.47
134.61
131.97
128.54
118.45
113.25
77.39
77.07
76.76
71.24
55.21
51.17
48.92
42.03
28.46
24.14
23.66
11.36
H_3CO
N
H
O
3c
180
170
160
150
140
130
120
110
100
90
80
70
60
50
40
30
20
10
0
f1 (ppm)

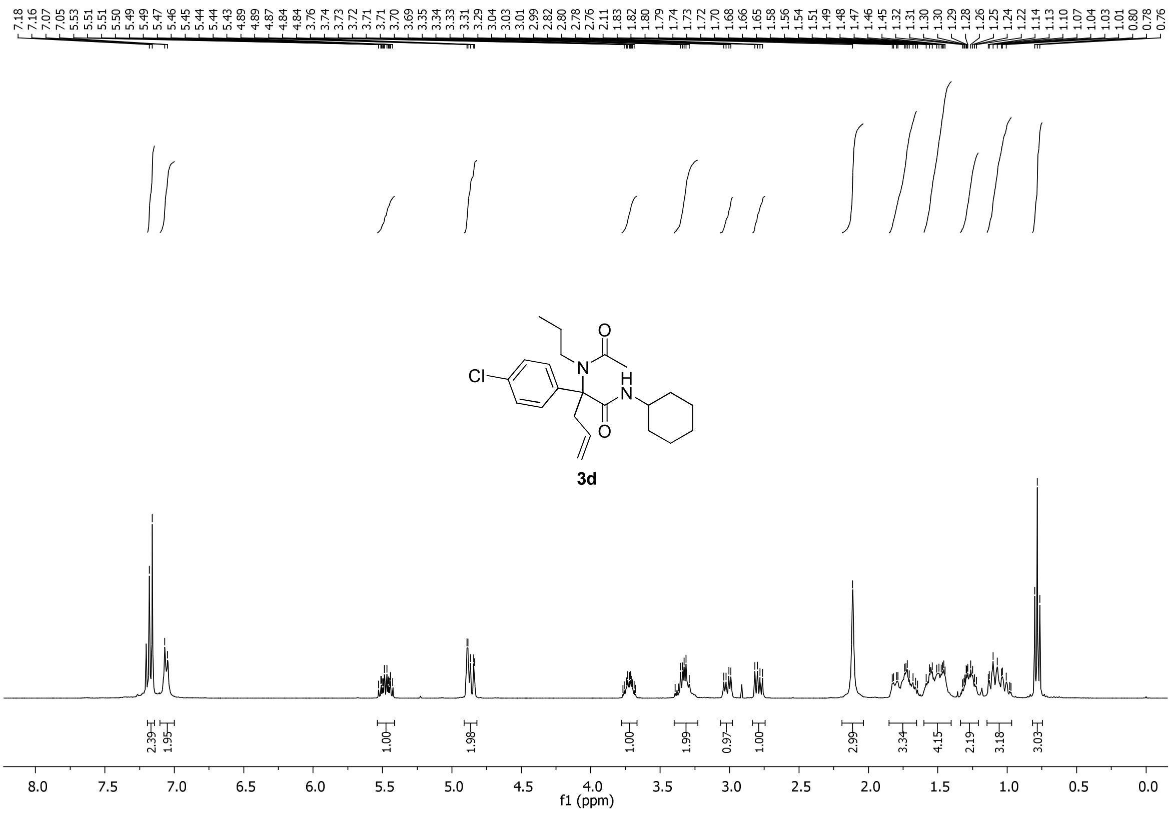
7.18
7.16
7.07
7.05
5.53
5.51
5.51
5.50
5.49
5.49
5.47
5.46
5.45
5.44
5.44
5.43
4.89
4.89
4.87
4.84
4.84
3.76
3.74
3.73
3.72
3.71
3.71
3.70
3.69
3.35
3.34
3.33
3.31
3.29
3.04
3.03
3.01
2.99
2.82
2.80
2.78
2.76
2.11
1.83
1.82
1.80
1.79
1.74
1.73
1.72
1.70
1.68
1.66
1.65
1.58
1.56
1.54
1.51
1.49
1.48
1.47
1.46
1.45
1.32
1.31
1.30
1.30
1.29
1.28
1.26
1.25
1.24
1.22
1.14
1.13
1.10
1.07
1.04
1.03
1.01
0.80
0.78
0.76
Cl
N
O
H
N
O
3d
2.39
1.95
1.00
1.98
1.00
1.99
0.97
1.00
2.99
3.34
4.15
2.19
3.18
3.03
8.0
7.5
7.0
6.5
6.0
5.5
5.0
4.5
4.0
3.5
3.0
2.5
2.0
1.5
1.0
0.5
0.0
f1 (ppm)

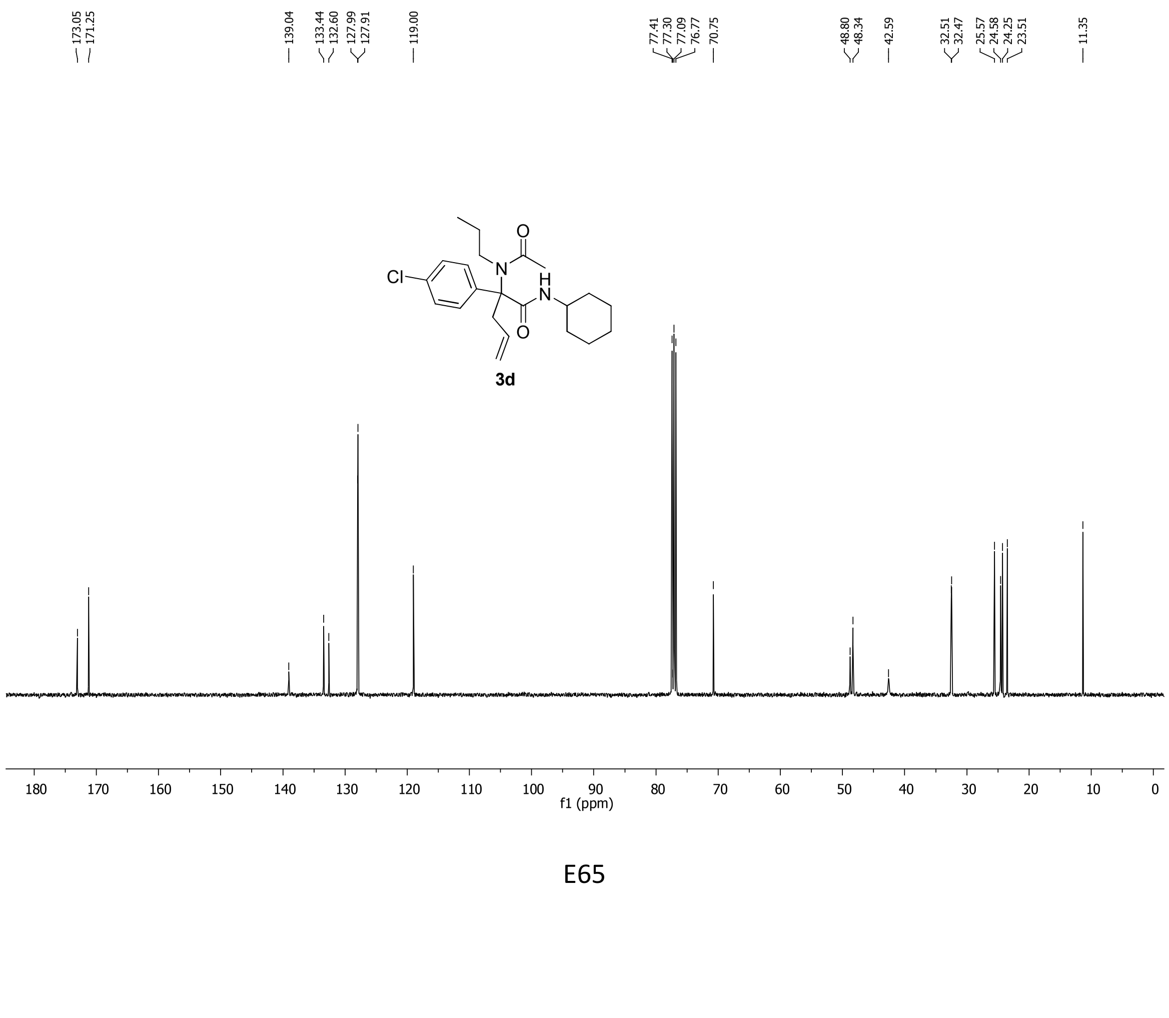
173.05
171.25
139.04
133.44
132.60
127.99
127.91
119.00
77.41
77.30
77.09
76.77
70.75
48.80
48.34
42.59
32.51
32.47
25.57
24.58
24.25
23.51
11.35
Cl
N
O
H
N
O
3d
180
170
160
150
140
130
120
110
100
90
80
70
60
50
40
30
20
10
0
f1 (ppm)

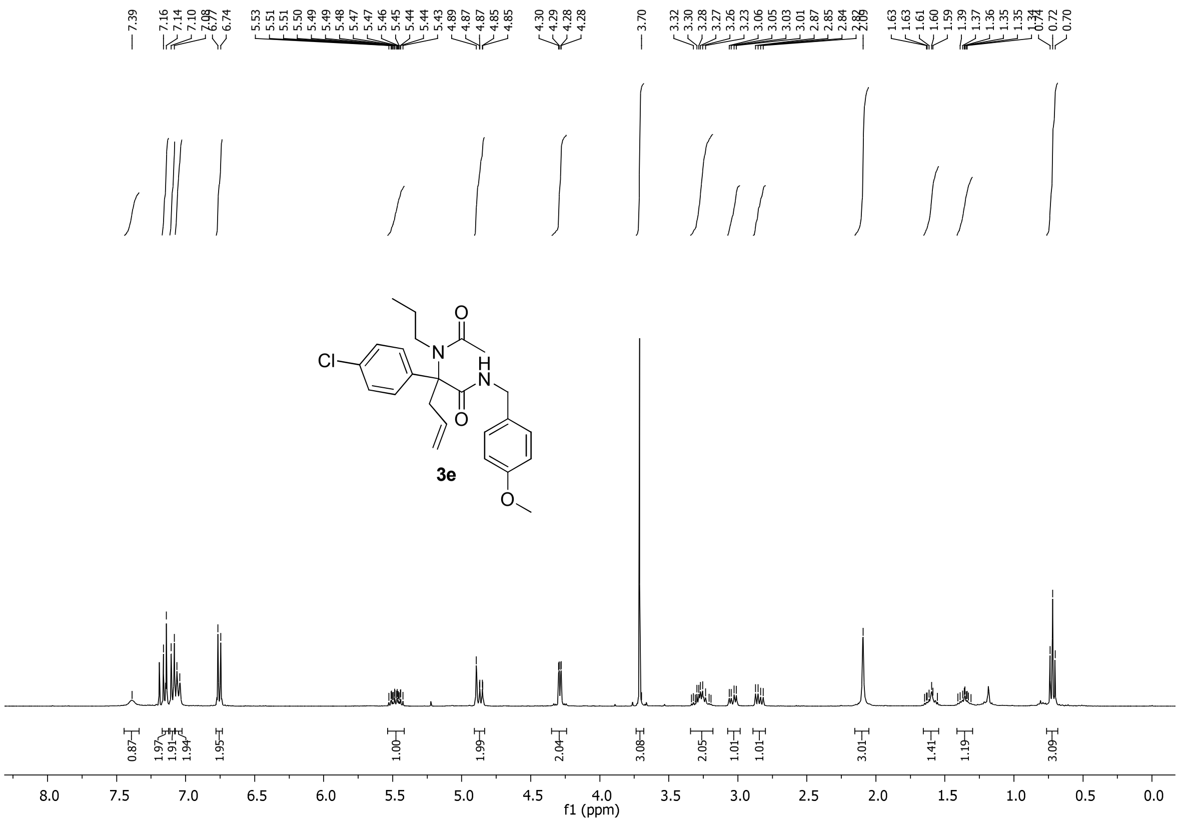
3e
Cl
N
H
O
f1 (ppm)

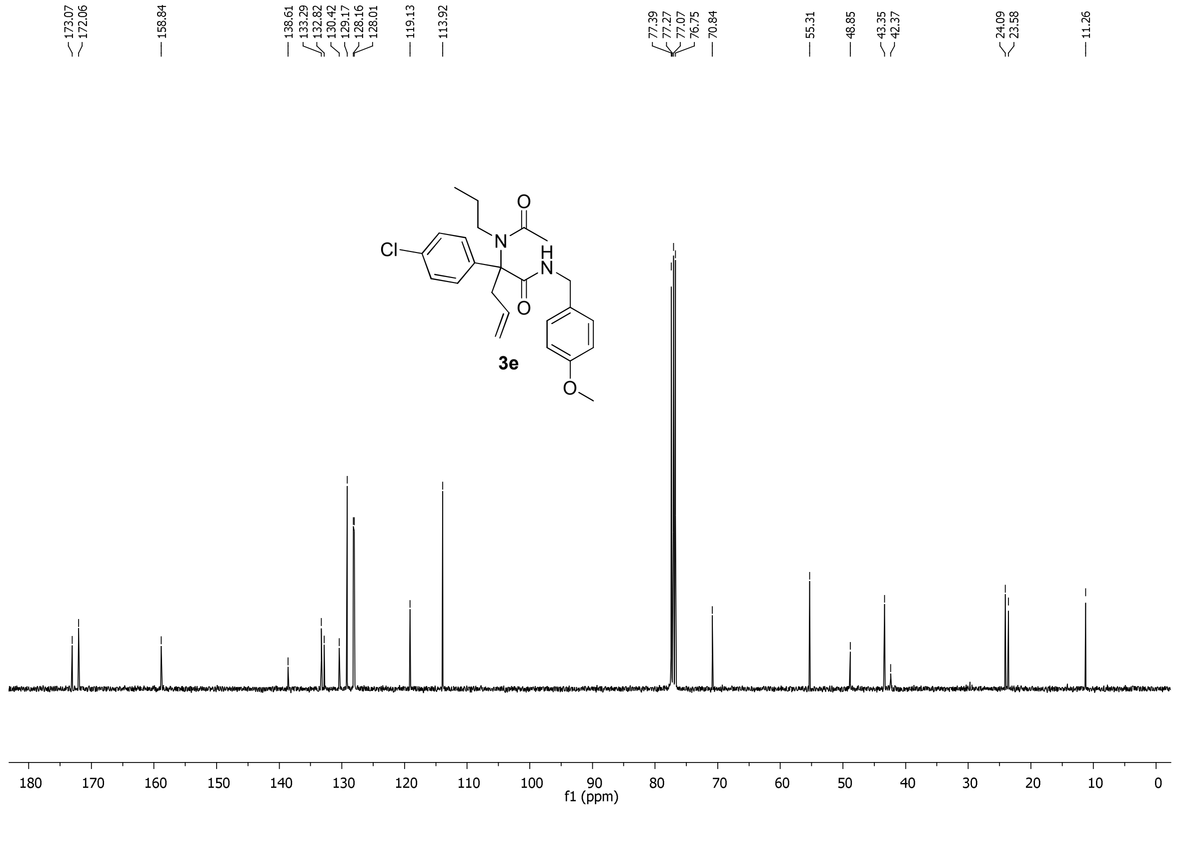
3e
Cl
N
H
O
f1 (ppm)

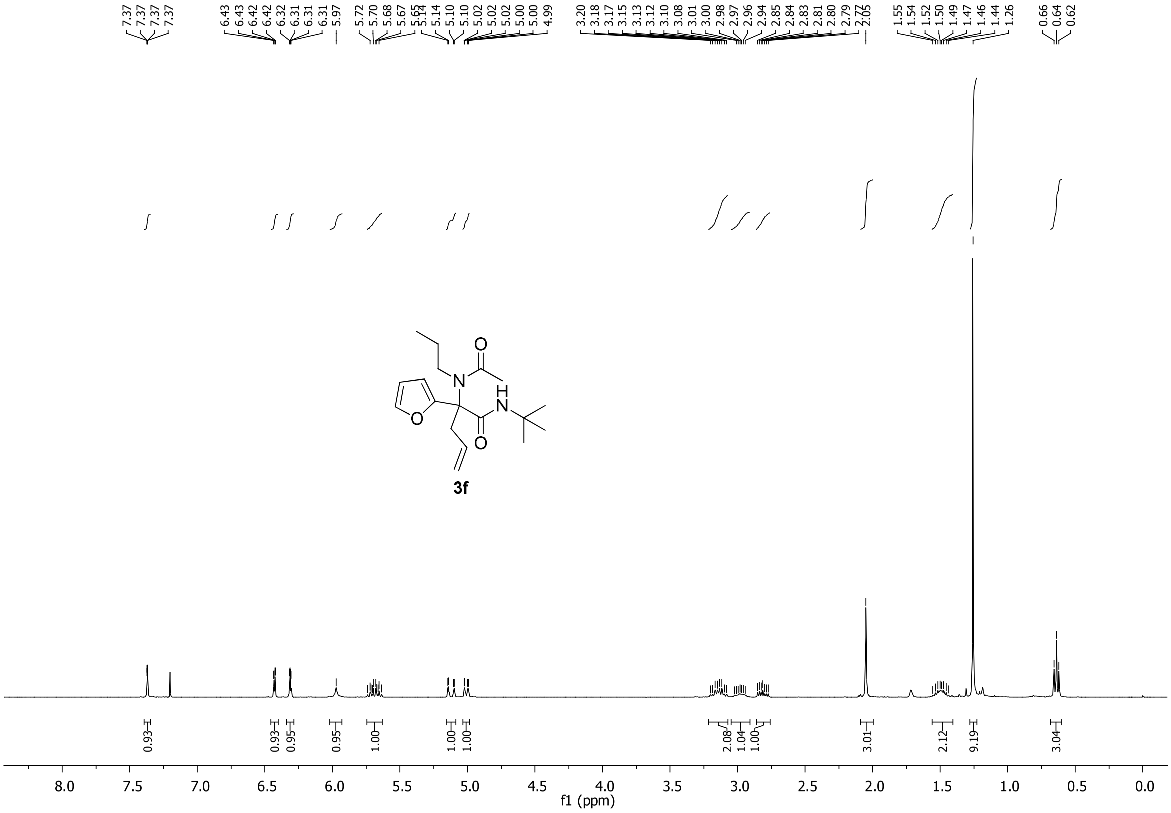
7.37
7.37
7.37
7.37
6.43
6.43
6.42
6.42
6.32
6.31
6.31
6.31
5.97
5.72
5.70
5.68
5.67
5.65
5.14
5.14
5.10
5.10
5.02
5.02
5.02
5.00
5.00
4.99
3.20
3.18
3.17
3.15
3.13
3.12
3.10
3.08
3.01
3.00
2.98
2.97
2.96
2.94
2.85
2.84
2.83
2.81
2.80
2.79
2.77
2.05
1.55
1.54
1.52
1.50
1.49
1.47
1.46
1.44
1.26
0.66
0.64
0.62
3f
0.93
0.93
0.95
0.95
1.00
1.00
1.00
2.08
1.04
1.00
3.01
2.12
9.19
3.04
8.0
7.5
7.0
6.5
6.0
5.5
5.0
4.5
4.0
3.5
3.0
2.5
2.0
1.5
1.0
0.5
0.0
f1 (ppm)

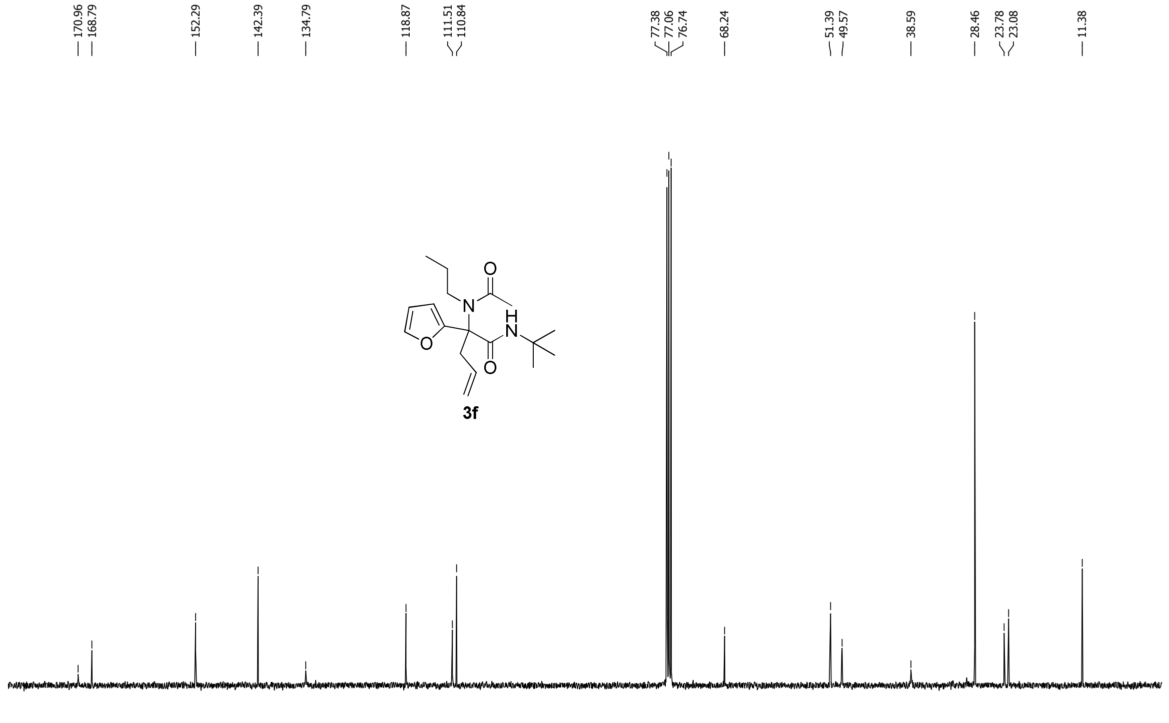
170.96
168.79
152.29
142.39
134.79
118.87
111.51
110.84
77.38
77.06
76.74
68.24
51.39
49.57
38.59
28.46
23.78
23.08
11.38
3f
180
170
160
150
140
130
120
110
100
90
80
70
60
50
40
30
20
10
0
f1 (ppm)

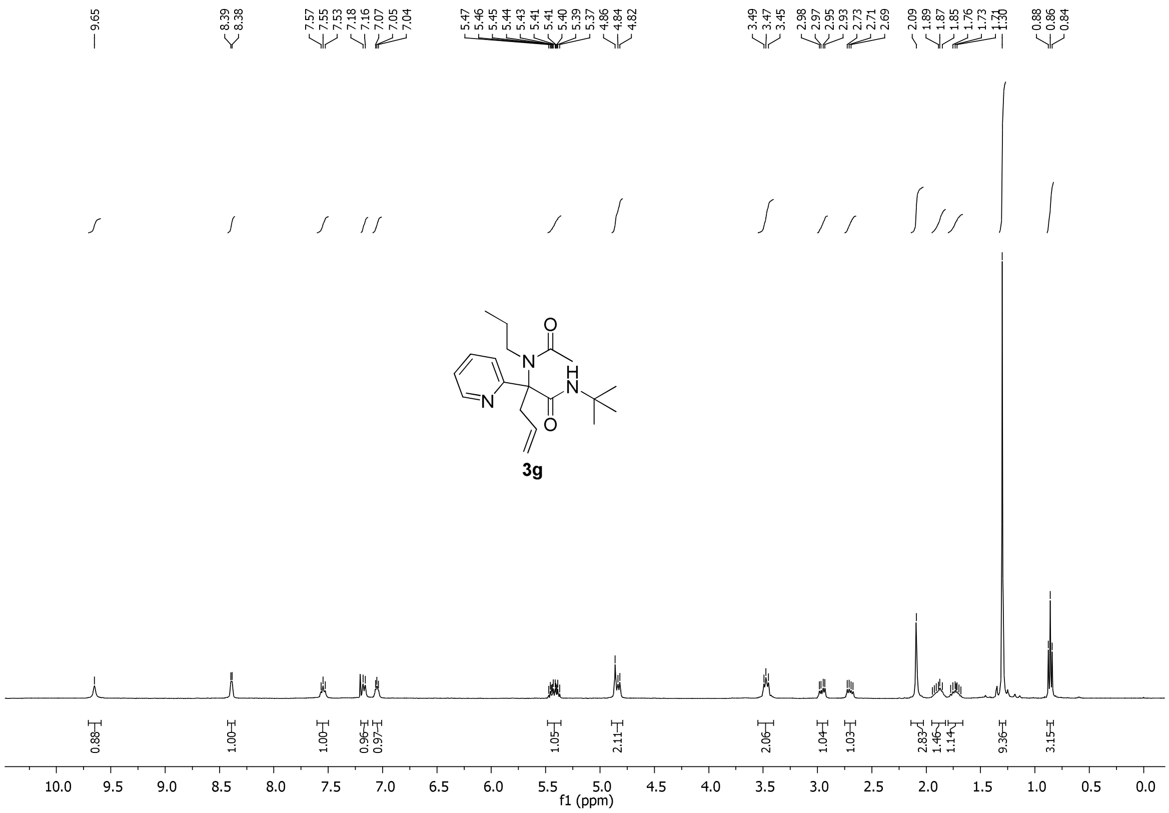
3g
f1 (ppm)

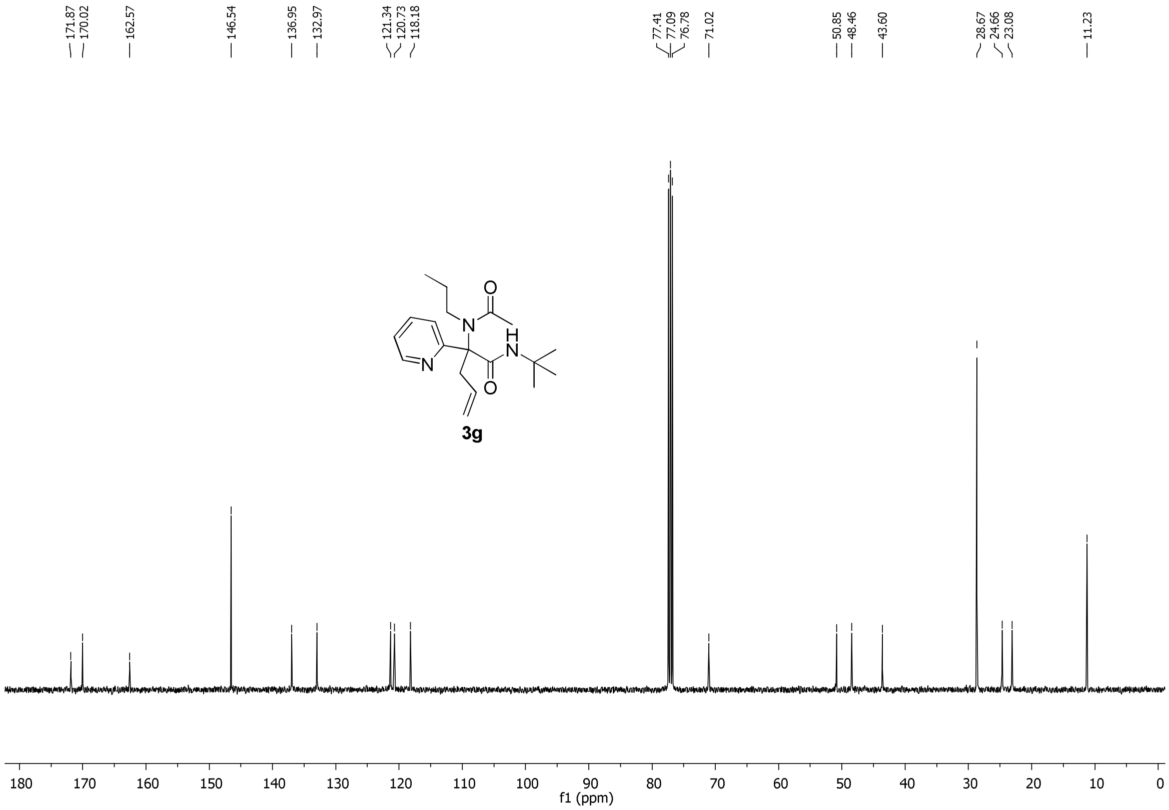
3g
f1 (ppm)

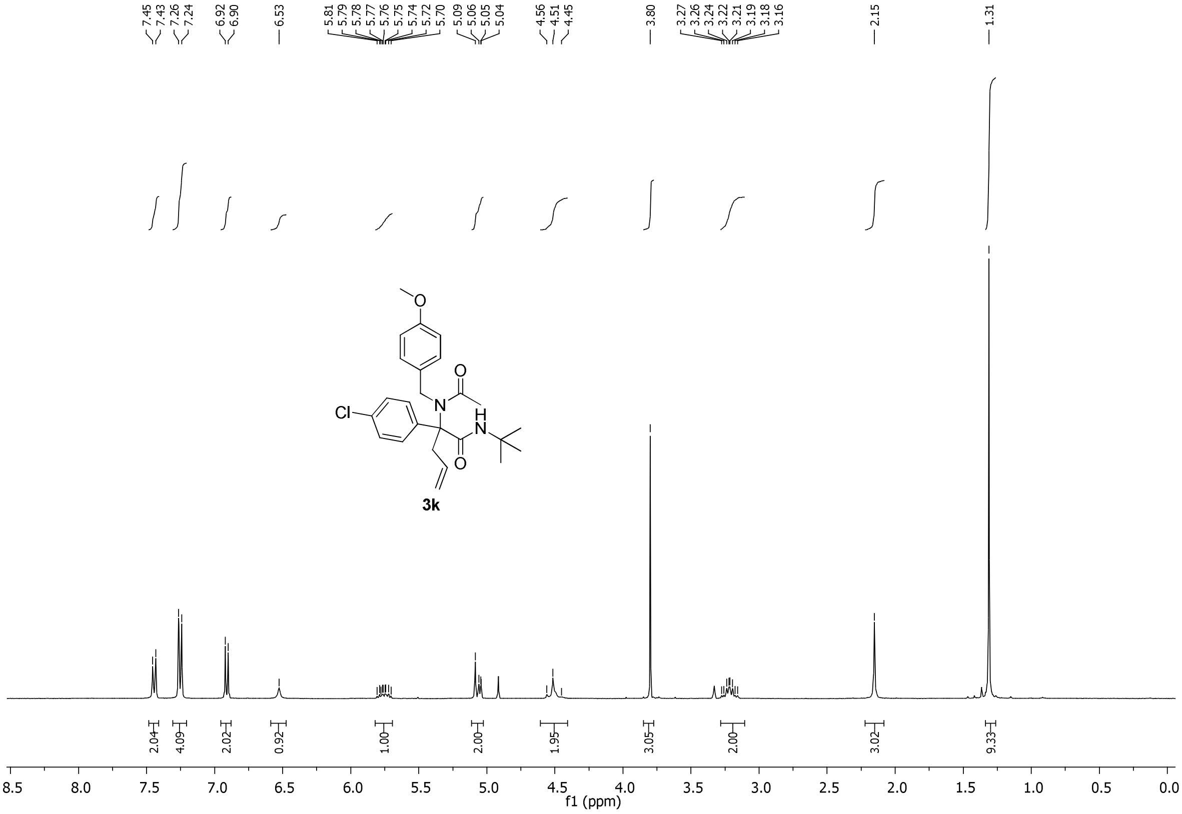
7.45
7.43
7.26
7.24
6.92
6.90
6.53
5.81
5.79
5.78
5.77
5.76
5.75
5.74
5.72
5.70
5.09
5.06
5.05
5.04
4.56
4.51
4.45
3.80
3.27
3.26
3.24
3.22
3.21
3.19
3.18
3.16
2.15
1.31
Cl
N
H
N
O
O
3k
2.04
4.09
2.02
0.92
1.00
2.00
1.95
3.05
2.00
3.02
9.33
8.5
8.0
7.5
7.0
6.5
6.0
5.5
5.0
4.5
4.0
3.5
3.0
2.5
2.0
1.5
1.0
0.5
0.0
f1 (ppm)

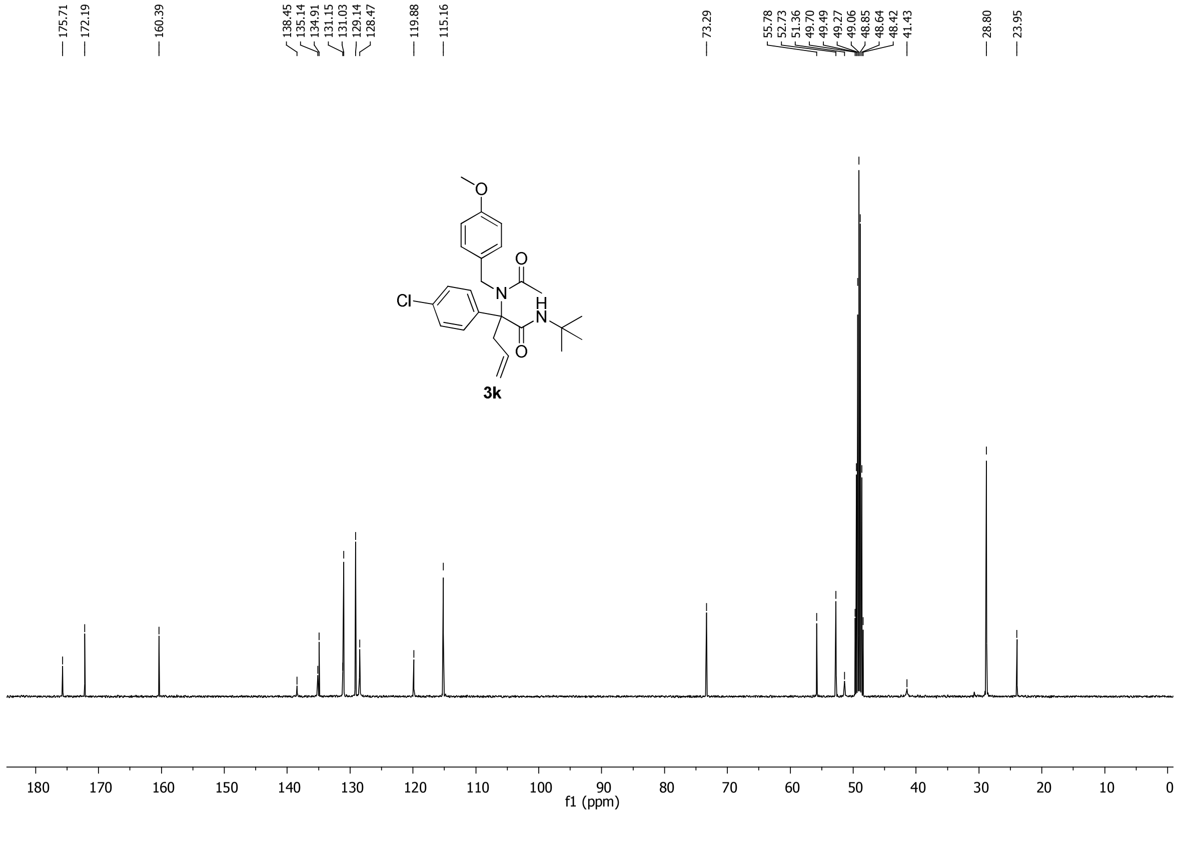
175.71
172.19
160.39
138.45
135.14
134.91
131.15
131.03
129.14
128.47
119.88
115.16
73.29
55.78
52.73
51.36
49.70
49.49
49.27
49.06
48.85
48.64
48.42
41.43
28.80
23.95
Cl
N
H
N
O
O
3k
180
170
160
150
140
130
120
110
100
90
80
70
60
50
40
30
20
10
0
f1 (ppm)

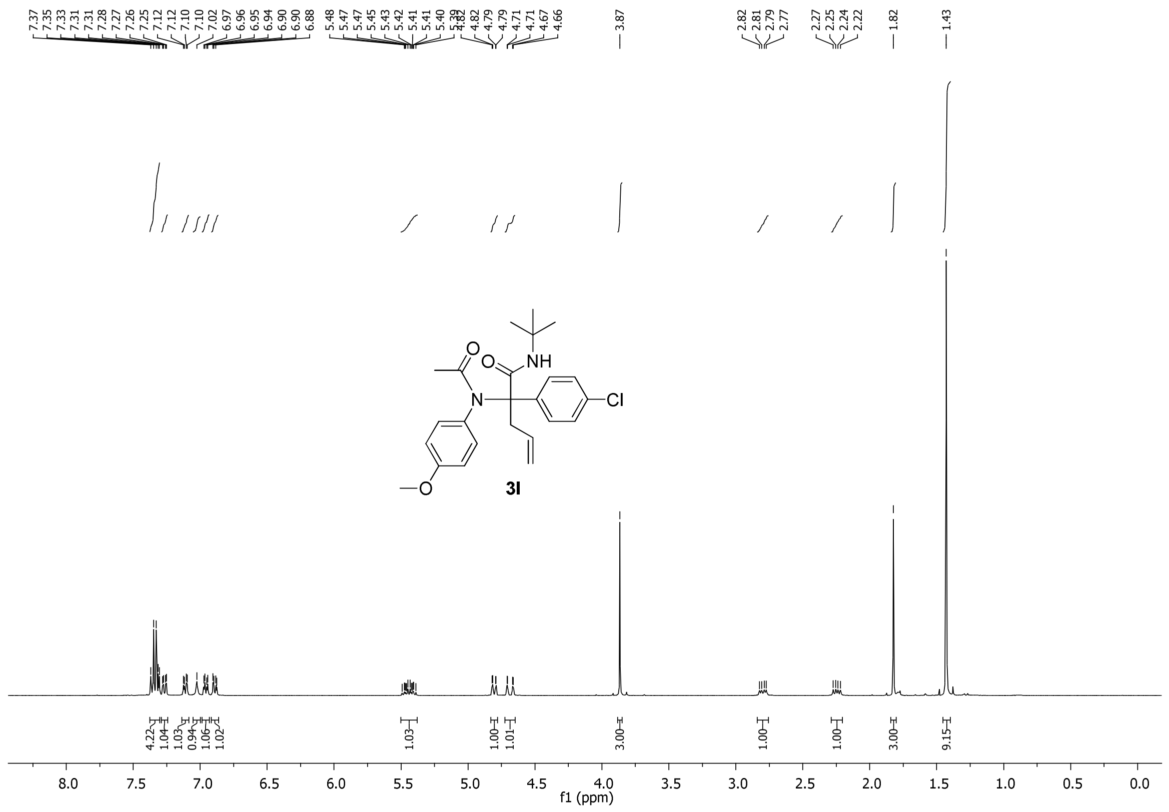

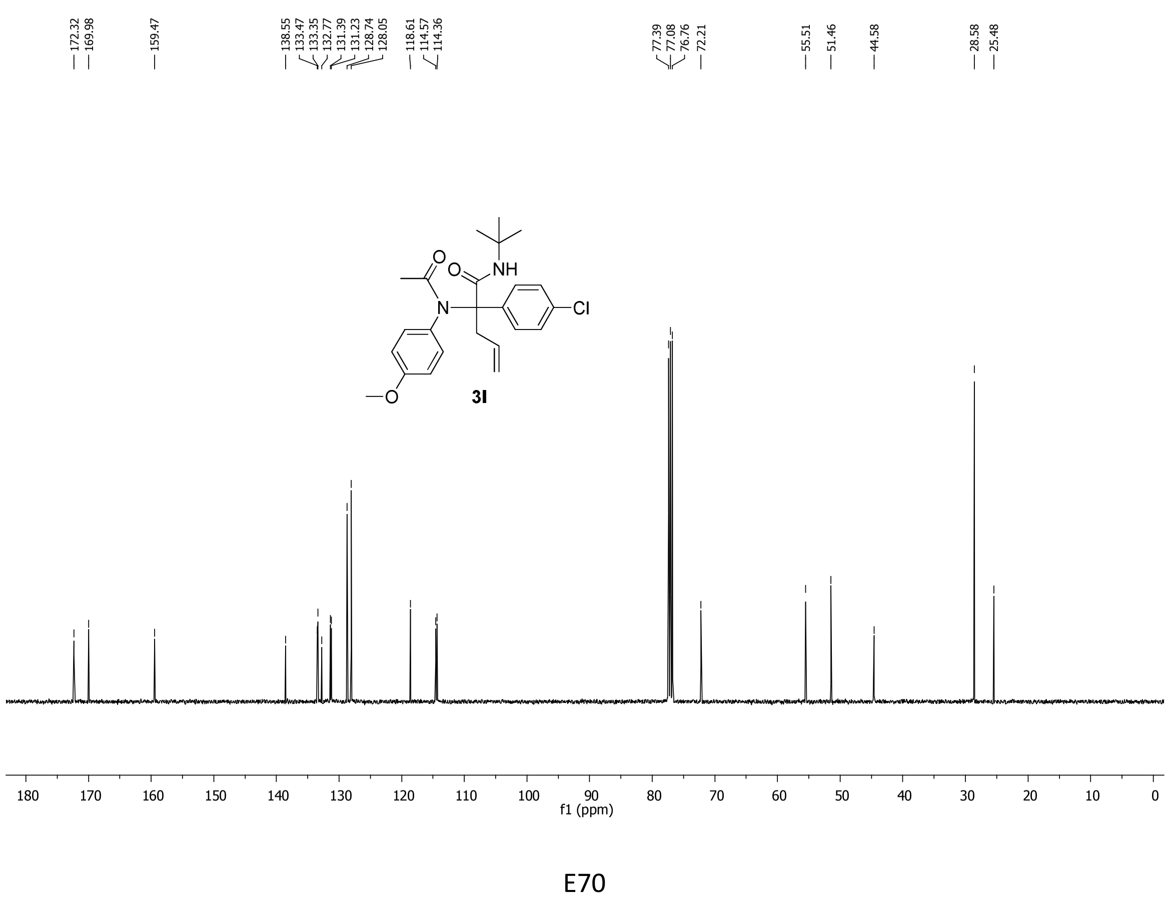

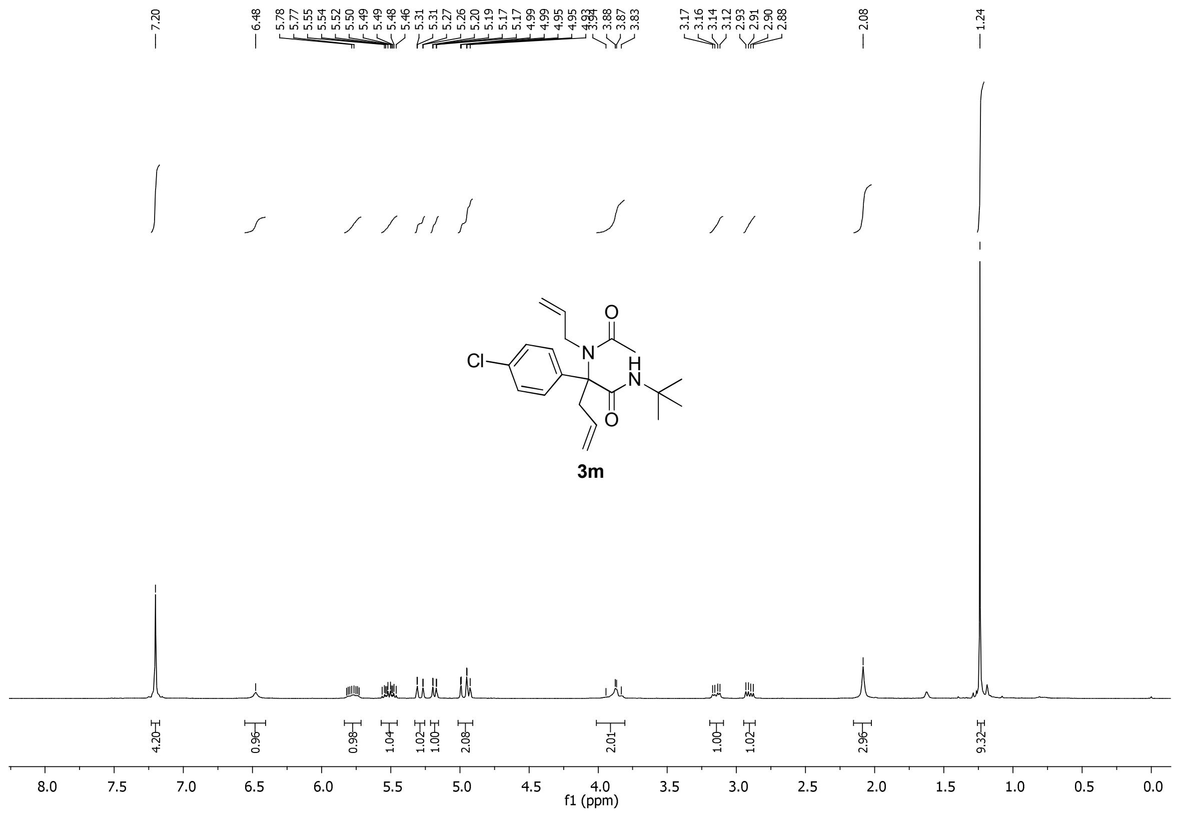

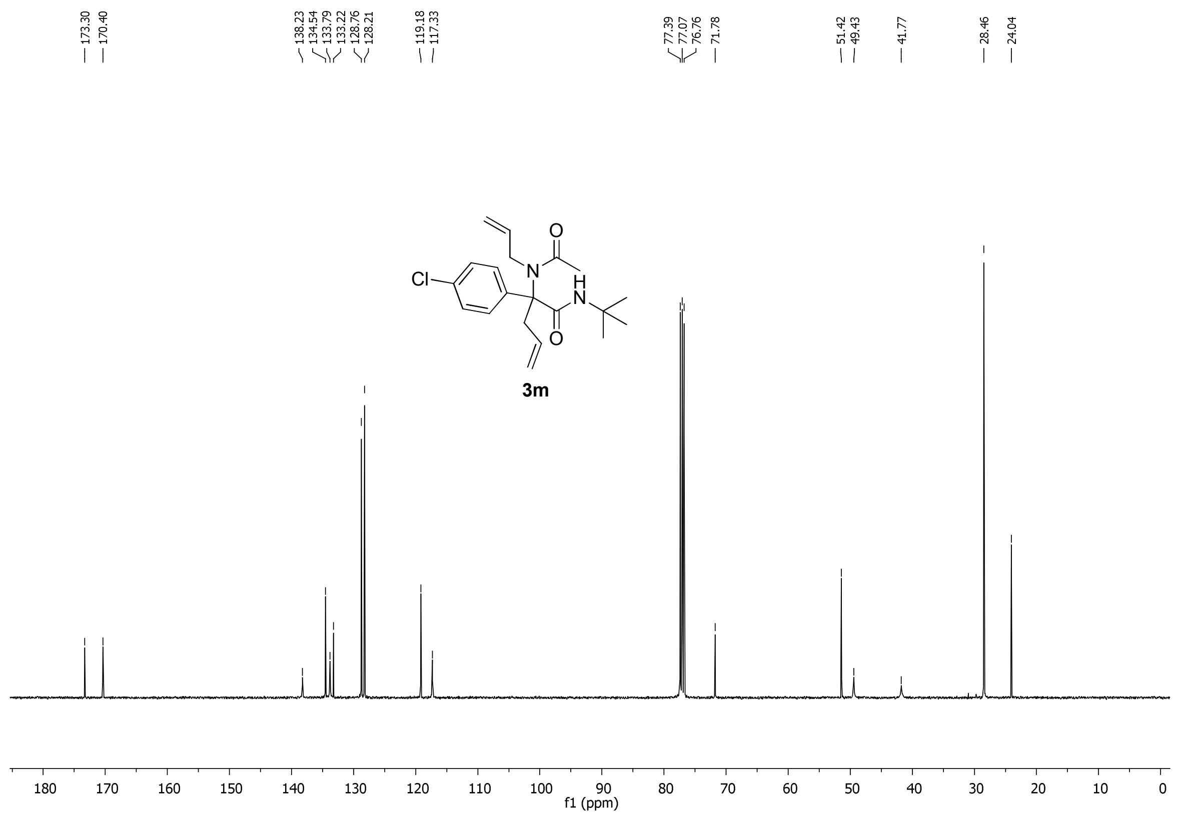

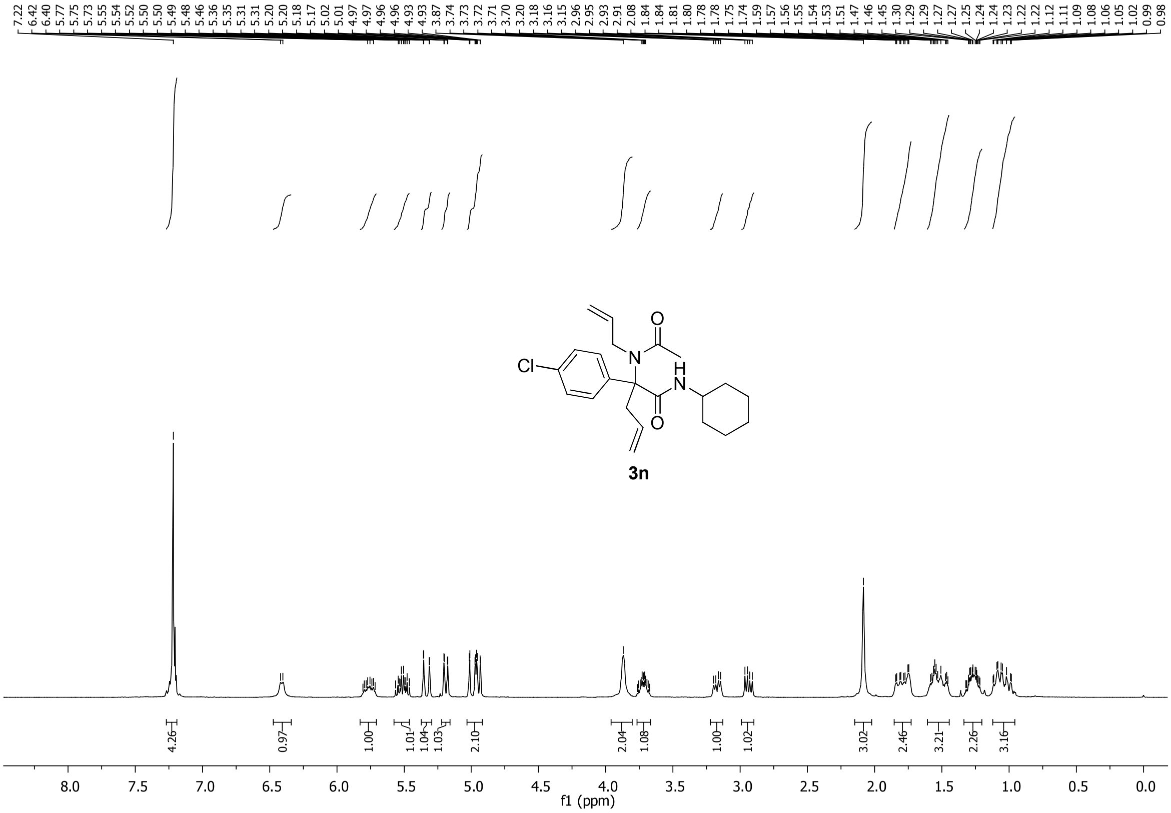
7.22 6.42 6.40 5.77 5.75 5.73 5.55 5.54 5.52 5.50 5.50 5.49 5.48 5.46 5.36 5.35 5.31 5.31 5.20 5.20 5.18 5.17 5.02 5.01 4.97 4.97 4.96 4.96 4.93 4.93 3.87 3.74 3.73 3.72 3.71 3.70 3.20 3.18 3.16 3.15 2.96 2.95 2.93 2.91 2.08 1.84 1.84 1.81 1.80 1.78 1.78 1.75 1.74 1.59 1.57 1.56 1.55 1.54 1.53 1.51 1.47 1.46 1.45 1.30 1.29 1.29 1.27 1.27 1.25 1.24 1.24 1.23 1.22 1.22 1.12 1.11 1.09 1.08 1.06 1.05 1.02 0.99 0.98
Cl
N
O
H
N
O
3n
4.26
0.97
1.00
1.01
1.04
1.03
2.10
2.04
1.08
1.00
1.02
3.02
2.46
3.21
2.26
3.16
8.0 7.5 7.0 6.5 6.0 5.5 5.0 4.5 4.0 3.5 3.0 2.5 2.0 1.5 1.0 0.5 0.0
f1 (ppm)

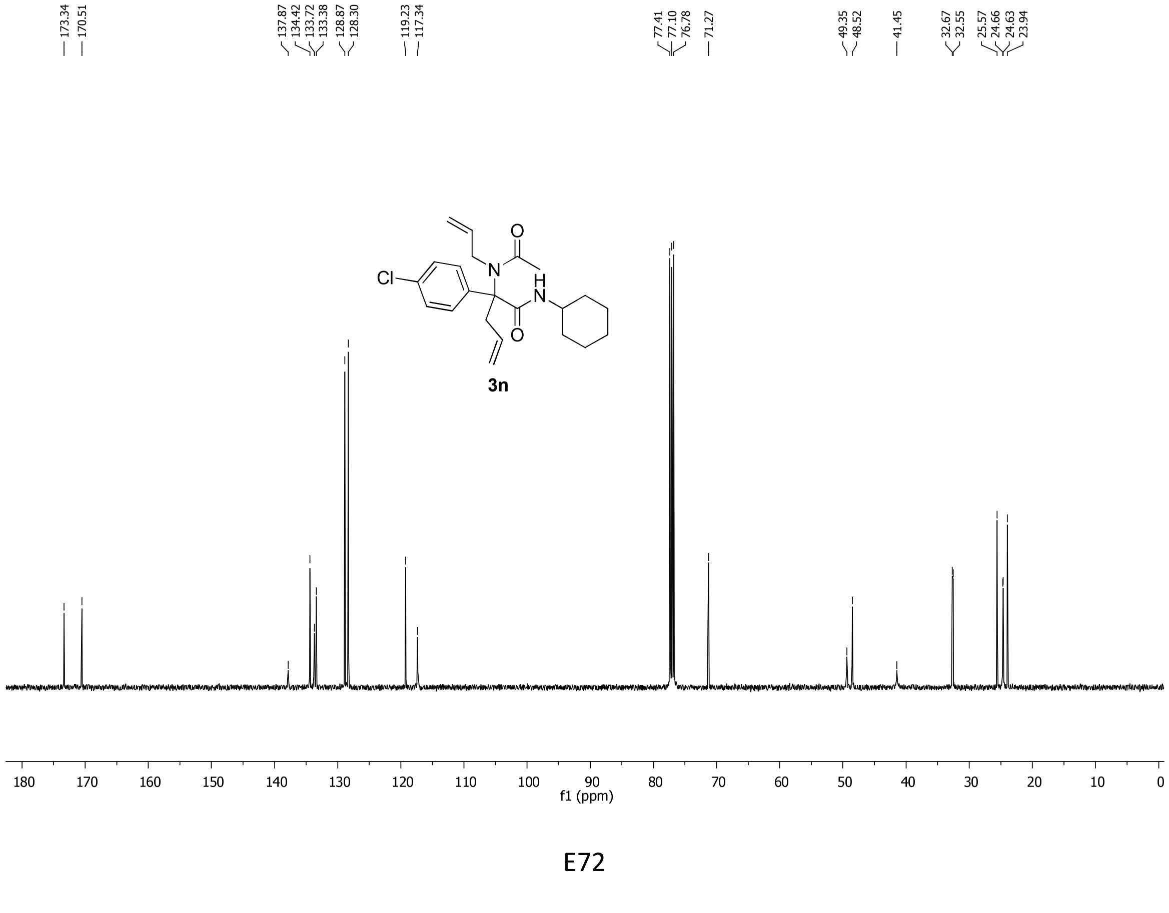
173.34 170.51 137.87 134.42 133.72 133.38 128.87 128.30 119.23 117.34 77.41 77.10 76.78 71.27 49.35 48.52 41.45 32.67 32.55 25.57 24.66 24.63 23.94
Cl
N
O
H
N
O
3n
180 170 160 150 140 130 120 110 100 90 80 70 60 50 40 30 20 10 0
f1 (ppm)

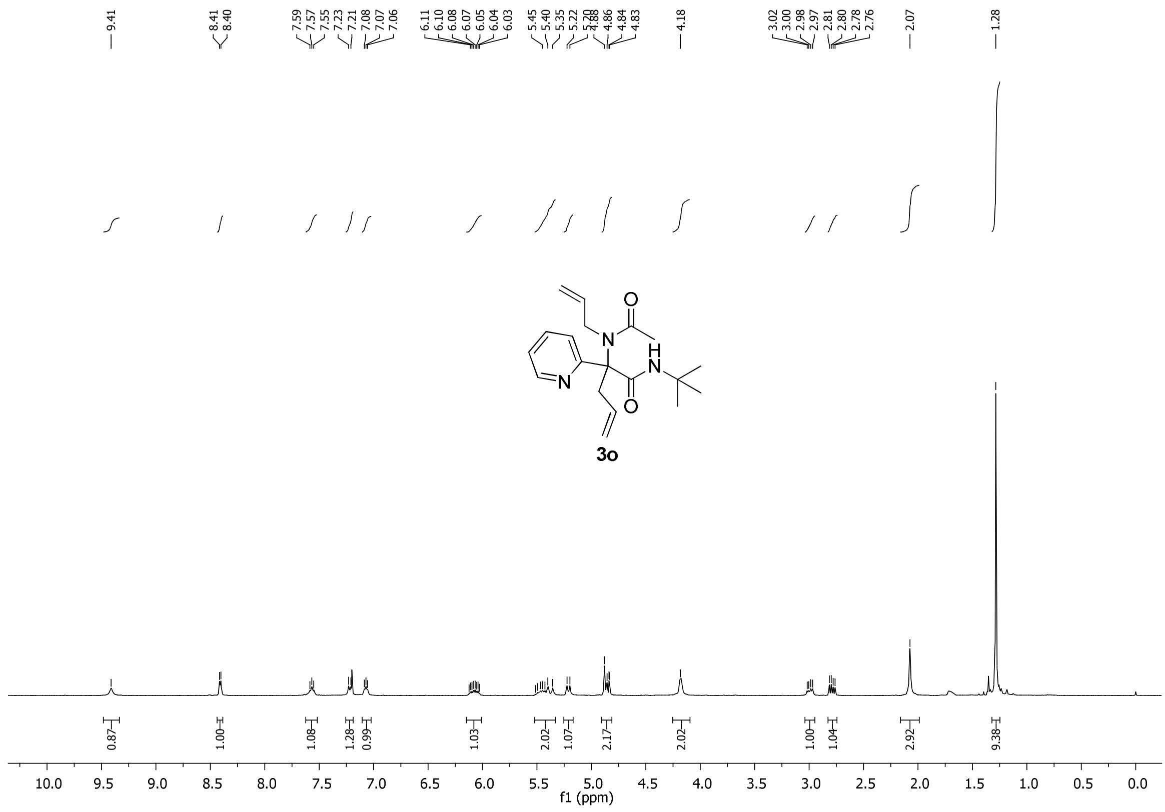
9.41
8.41
8.40
7.59
7.57
7.55
7.23
7.21
7.08
7.07
7.06
6.11
6.10
6.08
6.07
6.05
6.04
6.03
5.45
5.40
5.35
5.22
5.20
4.88
4.86
4.84
4.83
4.18
3.02
3.00
2.98
2.97
2.81
2.80
2.78
2.76
2.07
1.28
3o
0.87
1.00
1.08
1.28
0.99
1.03
2.02
1.07
2.17
2.02
1.00
1.04
2.92
9.38
f1 (ppm)

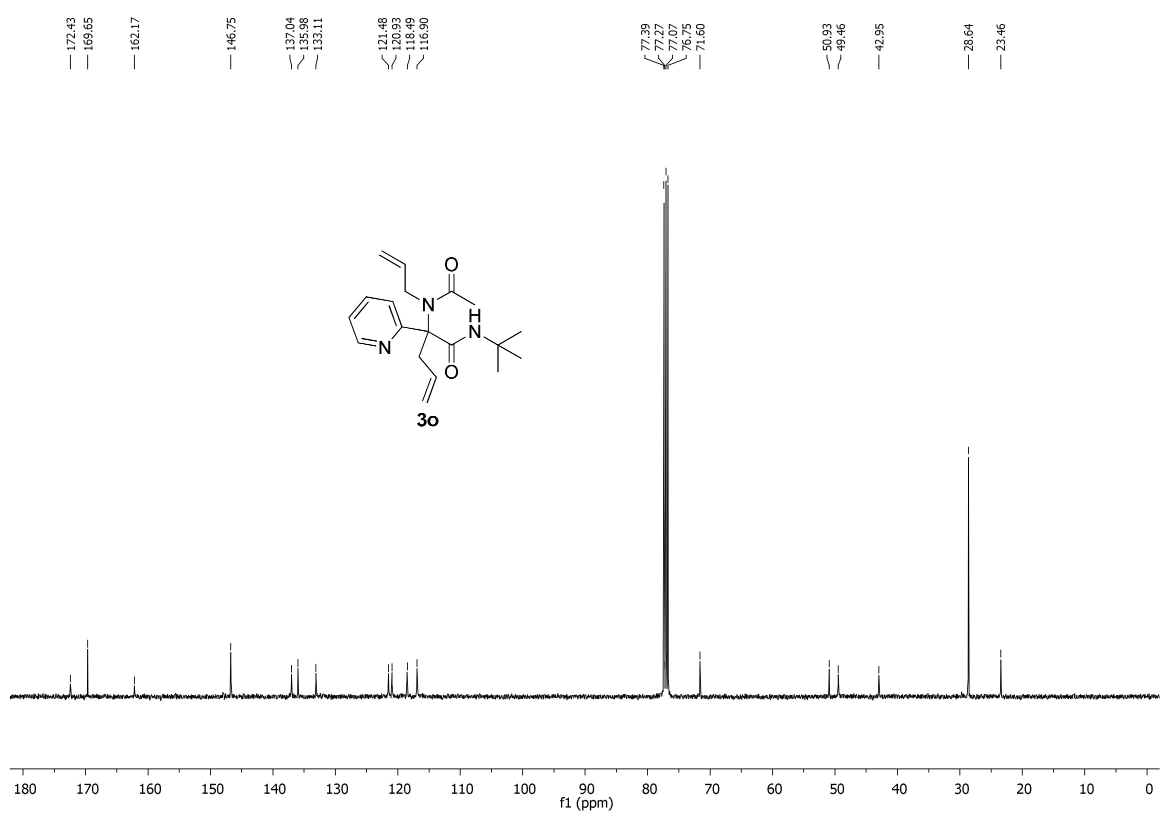
172.43
169.65
162.17
146.75
137.04
135.98
133.11
121.48
120.93
118.49
116.90
77.39
77.27
77.07
76.75
71.60
50.93
49.46
42.95
28.64
23.46
3o
f1 (ppm)

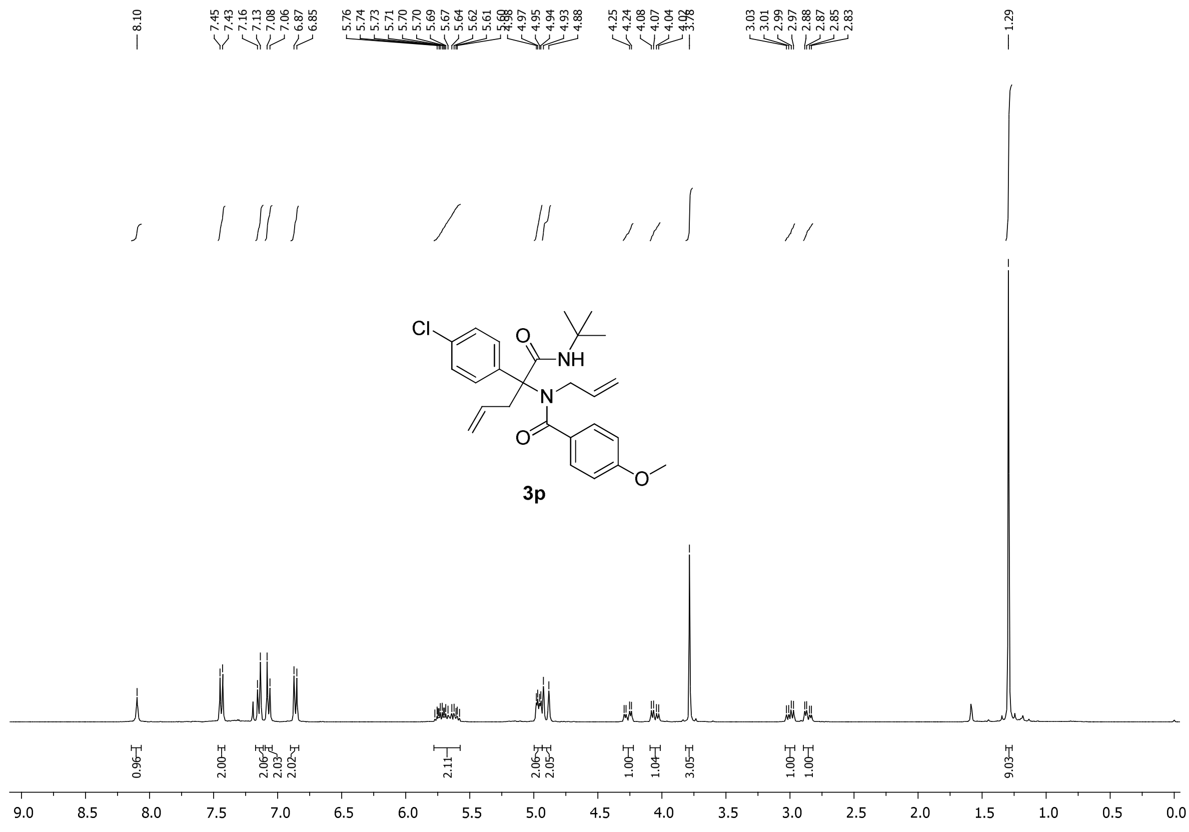
3p

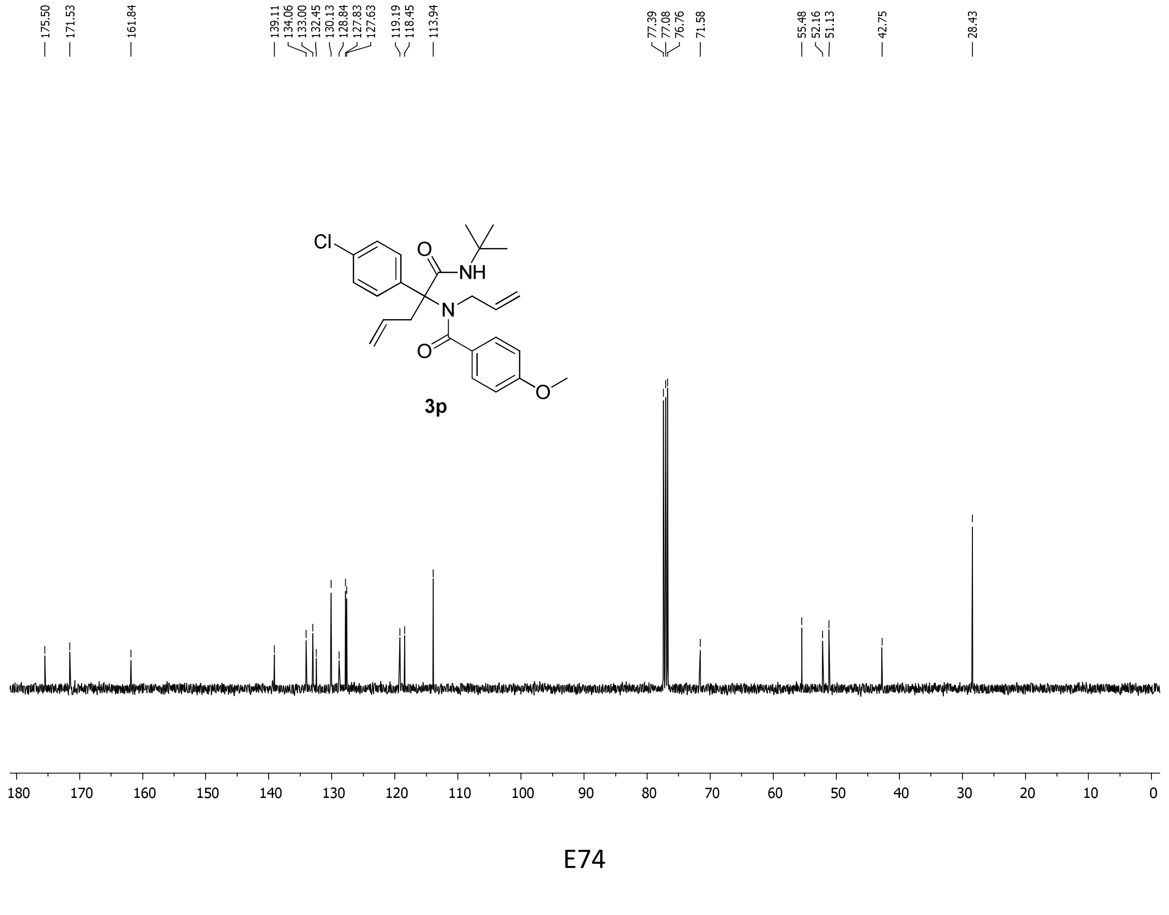
3p

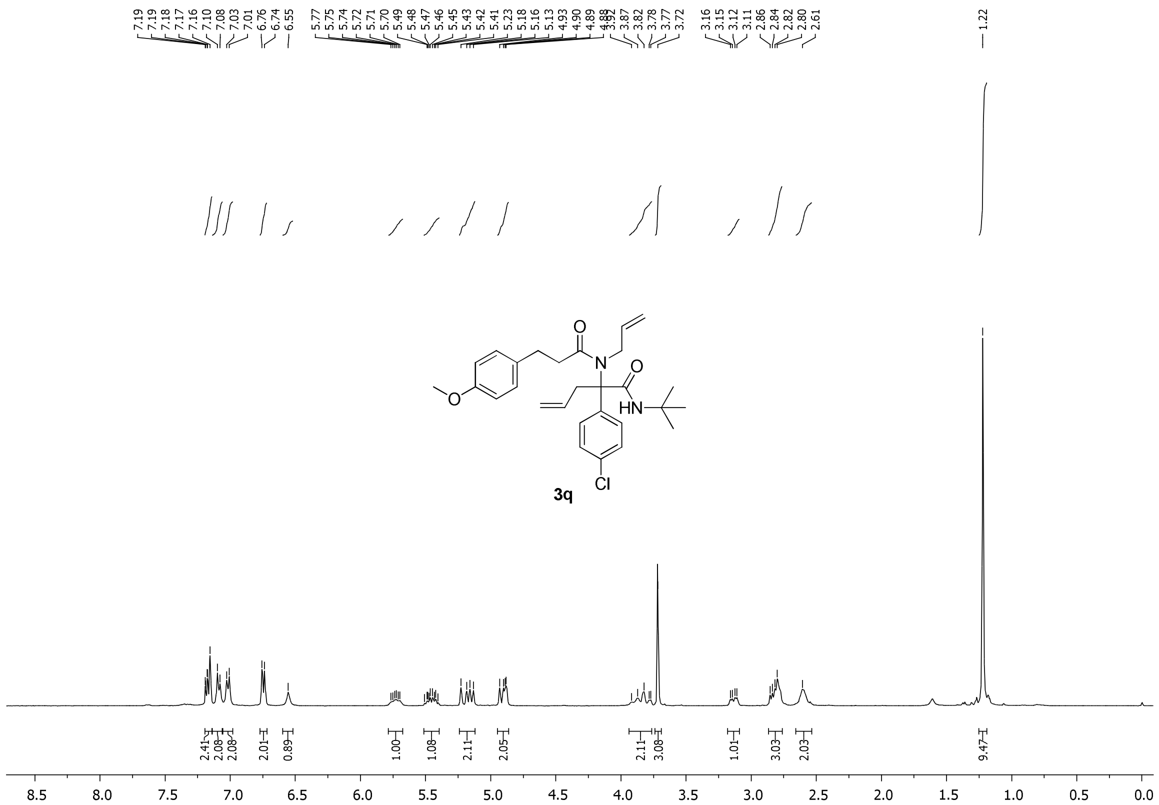
7.19 7.19 7.18 7.17 7.16 7.10 7.08 7.03 7.01 6.76 6.74 6.55 5.77 5.75 5.74 5.72 5.71 5.70 5.49 5.48 5.47 5.46 5.45 5.43 5.42 5.41 5.23 5.18 5.16 5.13 4.93 4.90 4.89 4.88 3.92 3.87 3.82 3.78 3.77 3.72 3.16 3.15 3.12 3.11 2.86 2.84 2.82 2.80 2.61 1.22
2.41 2.08 2.08 2.01 0.89 1.00 1.08 2.11 2.05 2.11 3.08 1.01 3.03 2.03 9.47
3q
O
N
HN
Cl
8.5 8.0 7.5 7.0 6.5 6.0 5.5 5.0 4.5 4.0 3.5 3.0 2.5 2.0 1.5 1.0 0.5 0.0

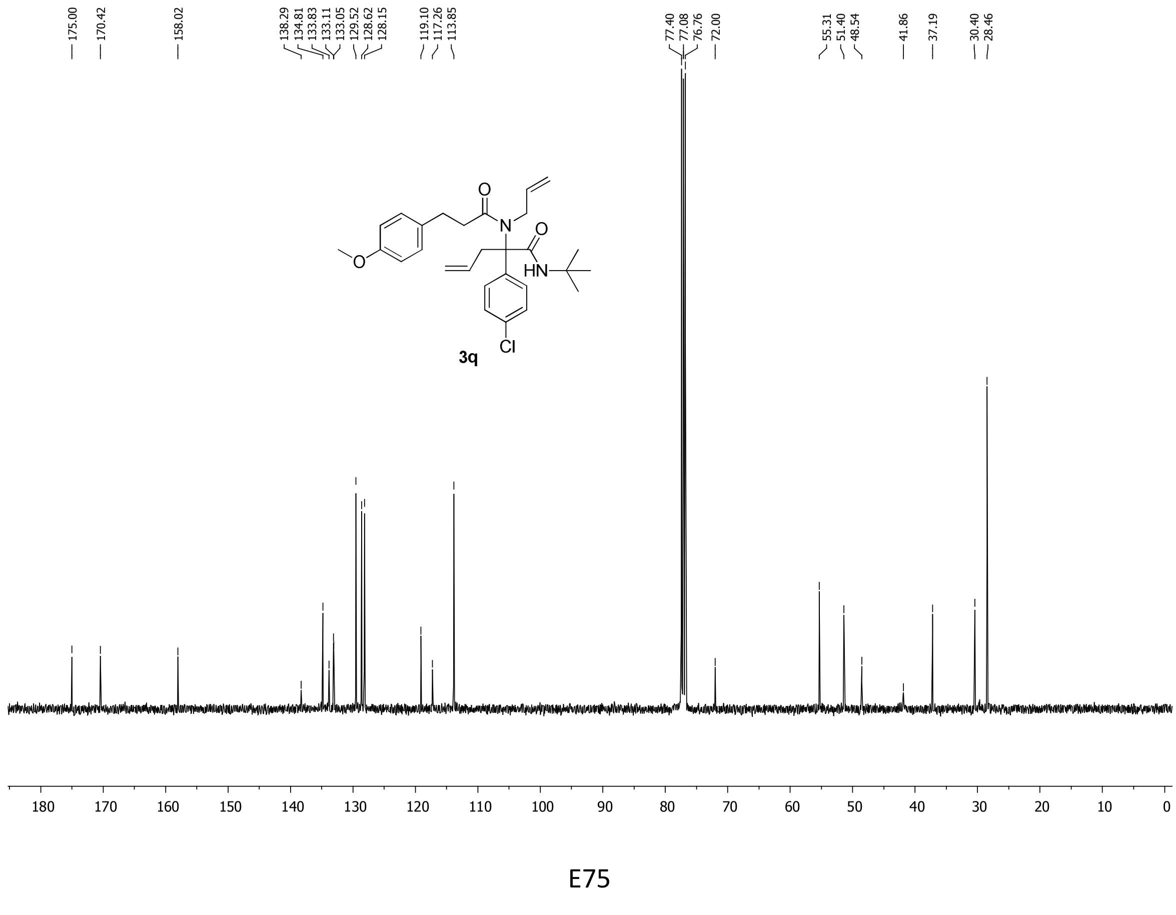
175.00 170.42 158.02 138.29 134.81 133.83 133.11 133.05 129.52 128.62 128.15 119.10 117.26 113.85 77.40 77.08 76.76 72.00 55.31 51.40 48.54 41.86 37.19 30.40 28.46
3q
O
N
HN
Cl
180 170 160 150 140 130 120 110 100 90 80 70 60 50 40 30 20 10 0

E75

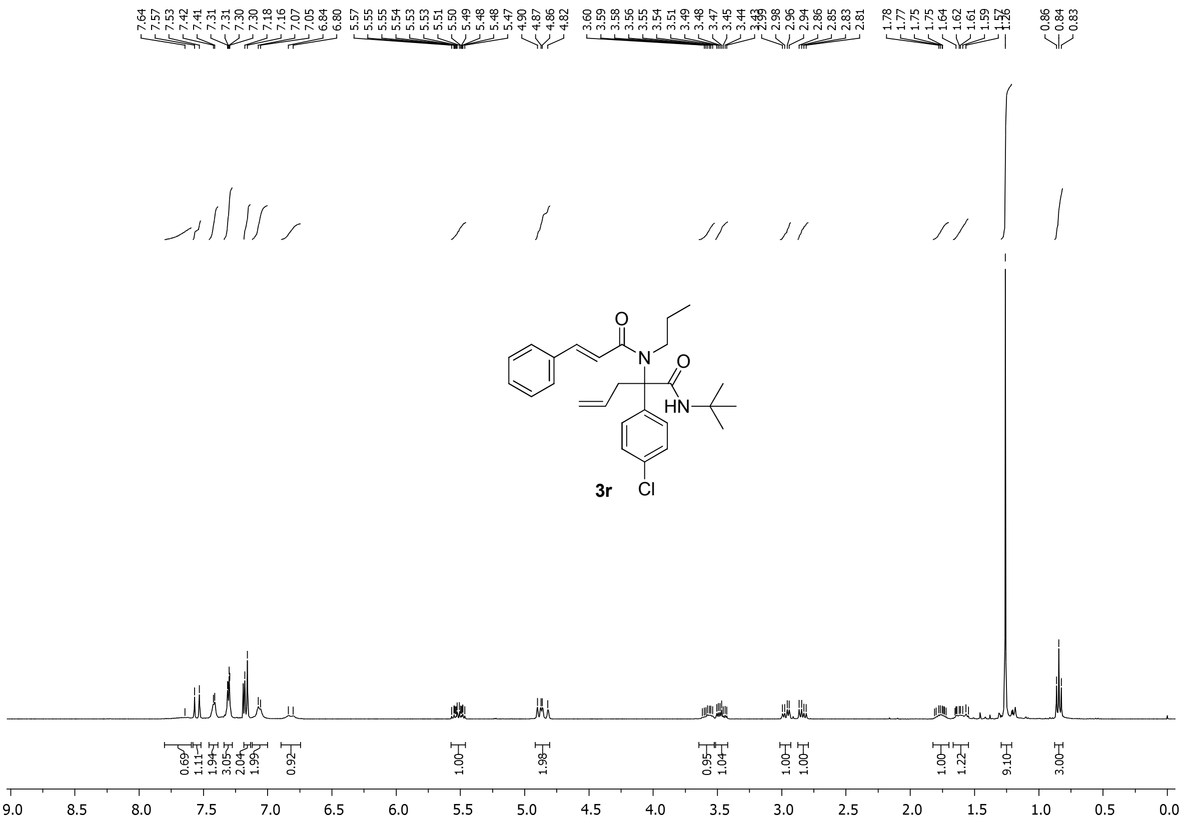
3r

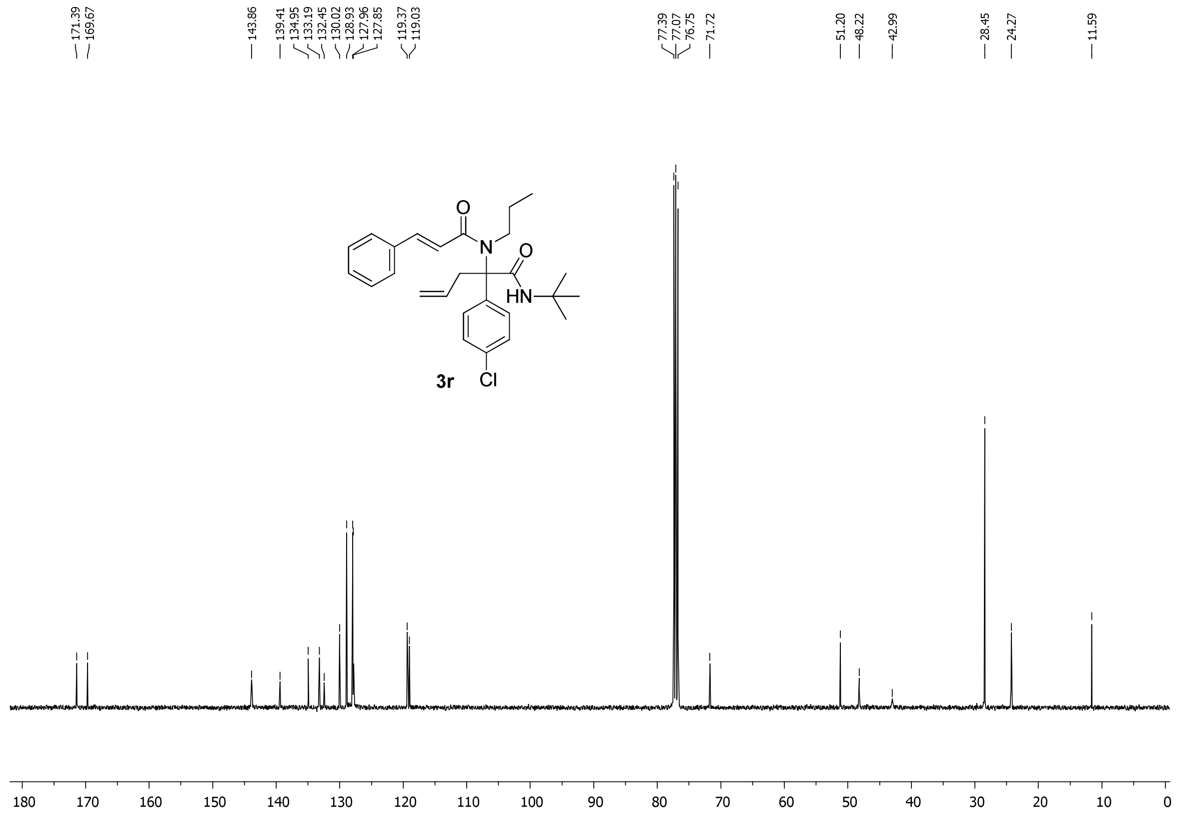
3r

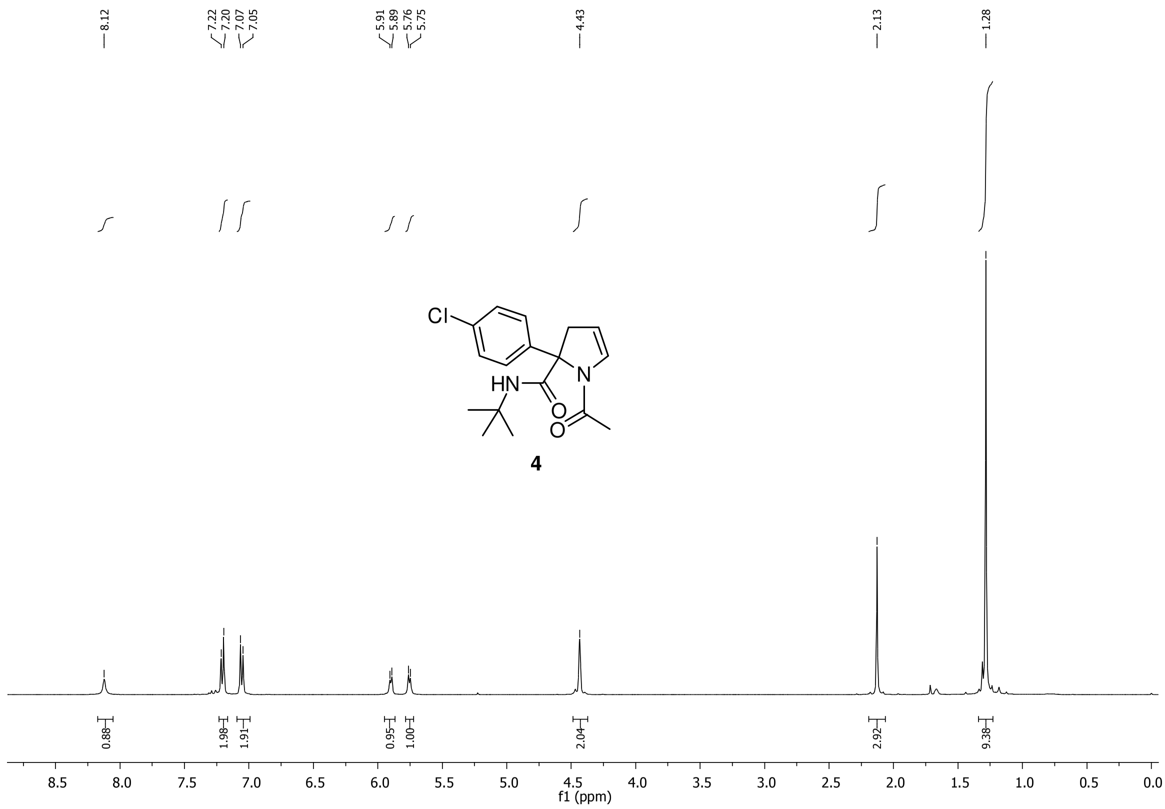
8.12
7.22
7.20
7.07
7.05
5.91
5.89
5.76
5.75
4.43
2.13
1.28
Cl
HN
N
O
O
4
0.88
1.98
1.91
0.95
1.00
2.04
2.92
9.38
f1 (ppm)

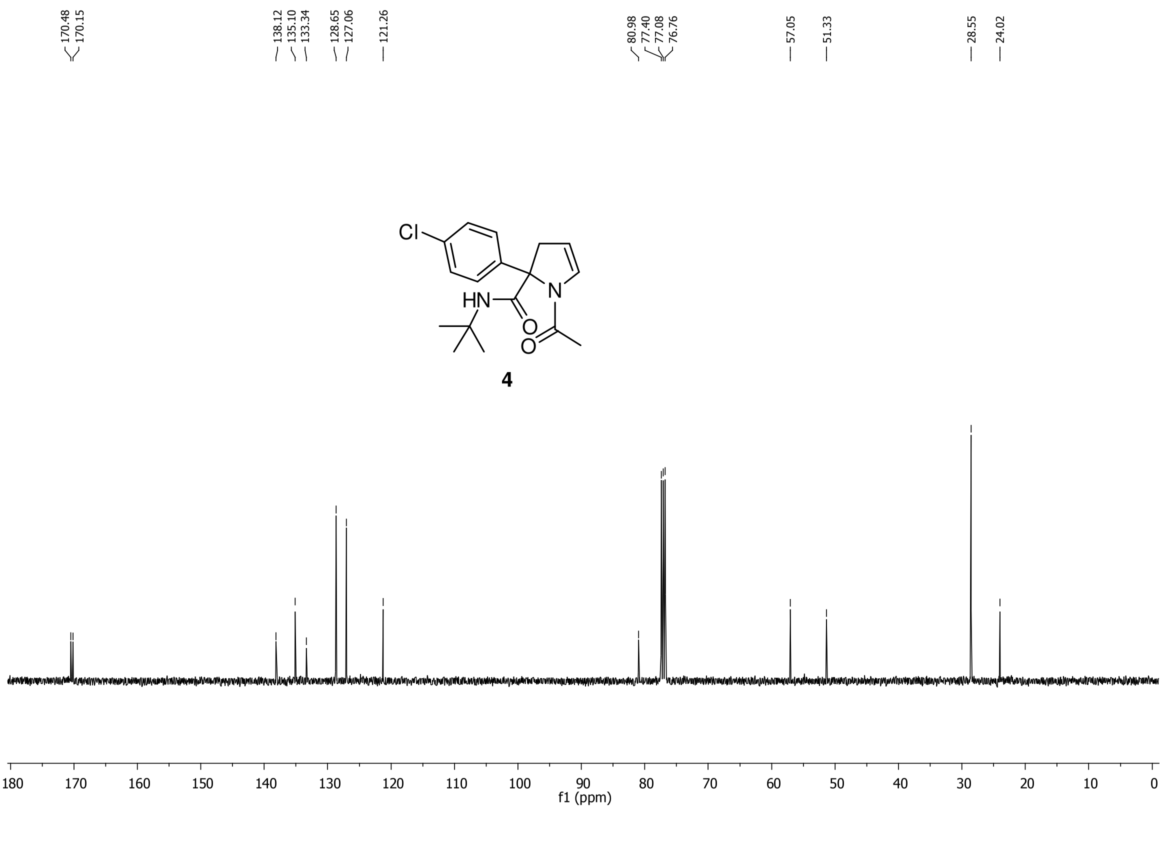
170.48
170.15
138.12
135.10
133.34
128.65
127.06
121.26
80.98
77.40
77.08
76.76
57.05
51.33
28.55
24.02
Cl
HN
N
O
O
4
f1 (ppm)

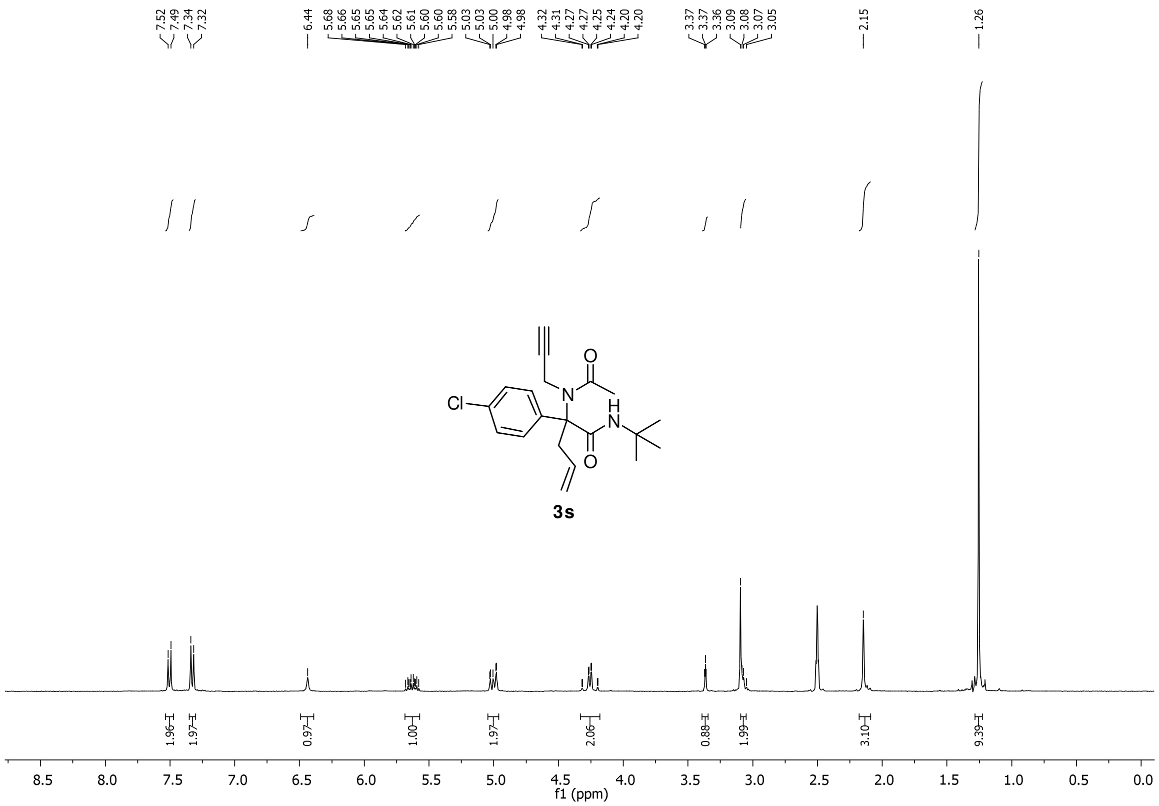
7.52
7.49
7.34
7.32
6.44
5.68
5.66
5.65
5.65
5.64
5.62
5.61
5.60
5.60
5.58
5.03
5.03
5.00
4.98
4.98
4.32
4.31
4.27
4.27
4.25
4.24
4.20
4.20
3.37
3.37
3.36
3.09
3.08
3.07
3.05
2.15
1.26
Cl
N
O
H
N
O
3s
1.96
1.97
0.97
1.00
1.97
2.06
0.88
1.99
3.10
9.39
f1 (ppm)

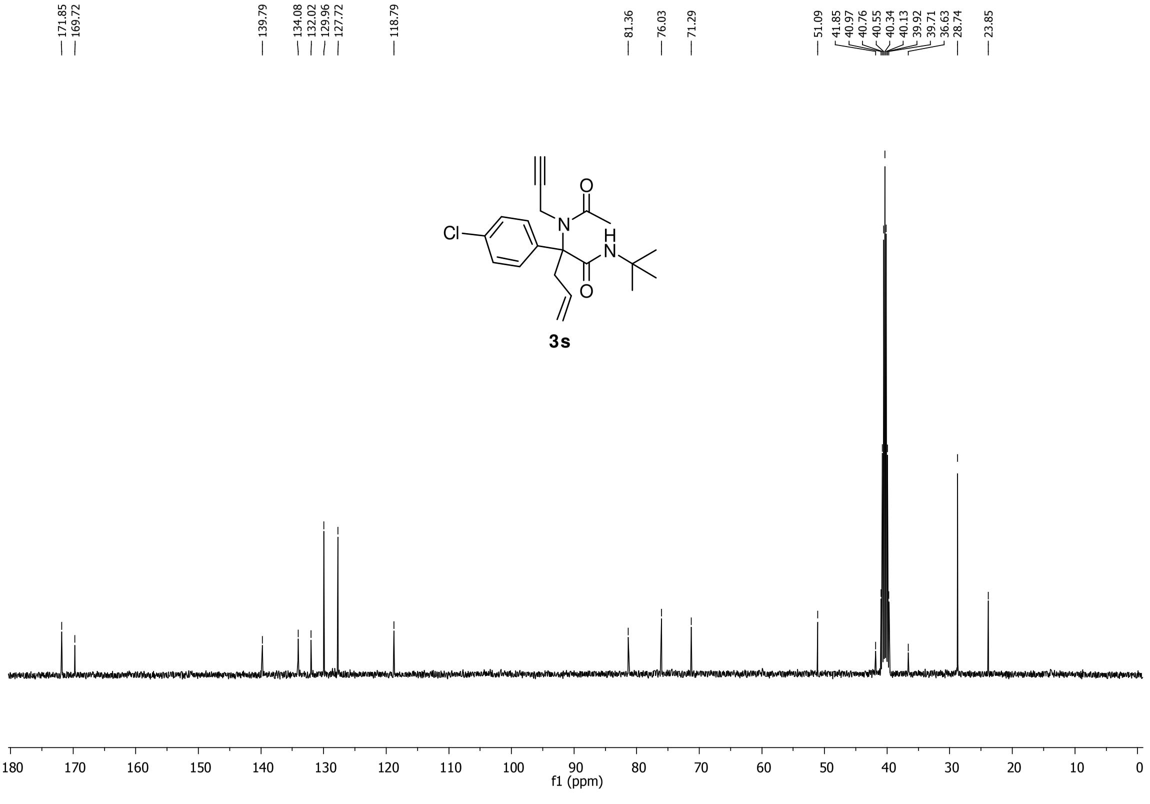
171.85
169.72
139.79
134.08
132.02
129.96
127.72
118.79
81.36
76.03
71.29
51.09
41.85
40.97
40.76
40.55
40.34
40.13
39.92
39.71
36.63
28.74
23.85
Cl
N
O
H
N
O
3s
f1 (ppm)

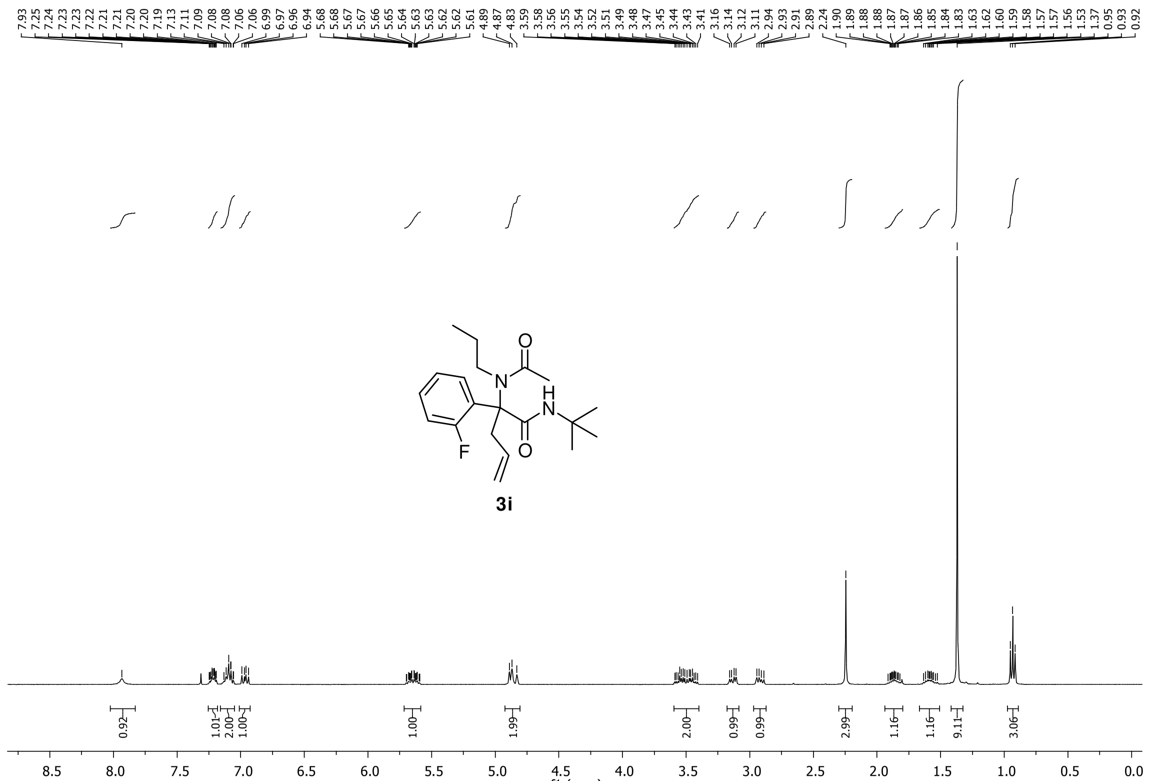

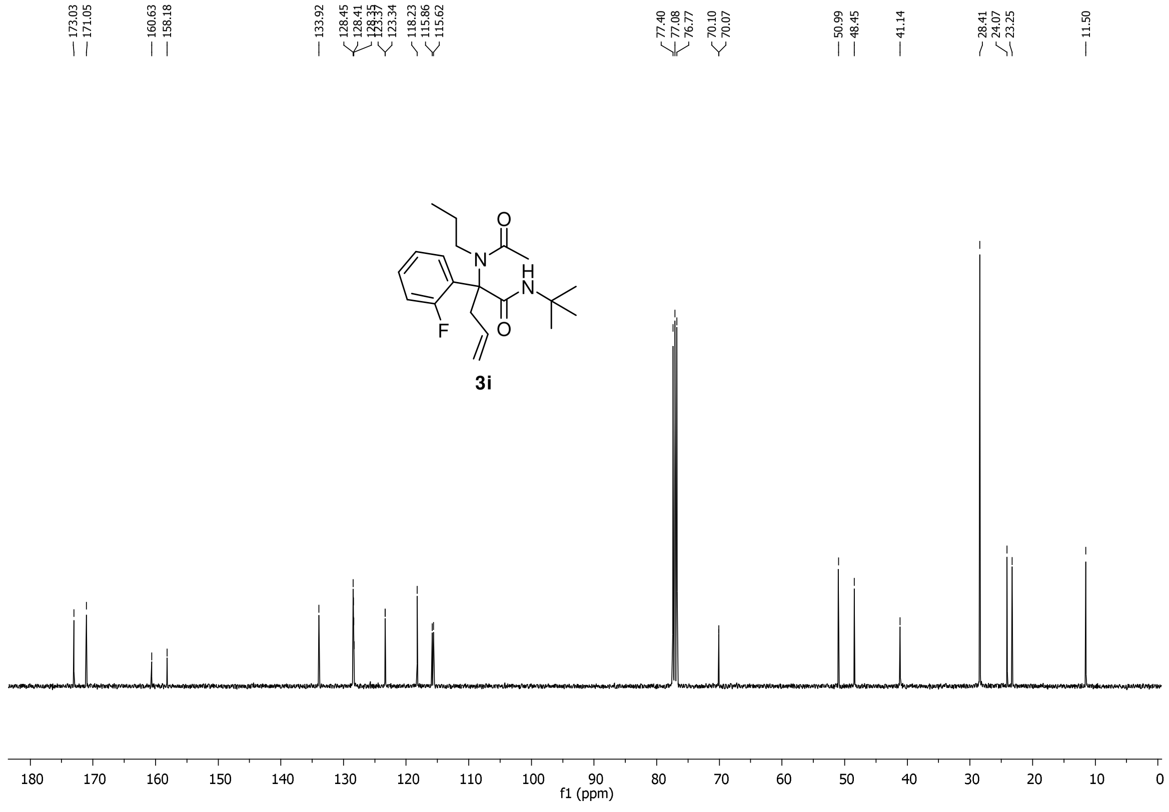

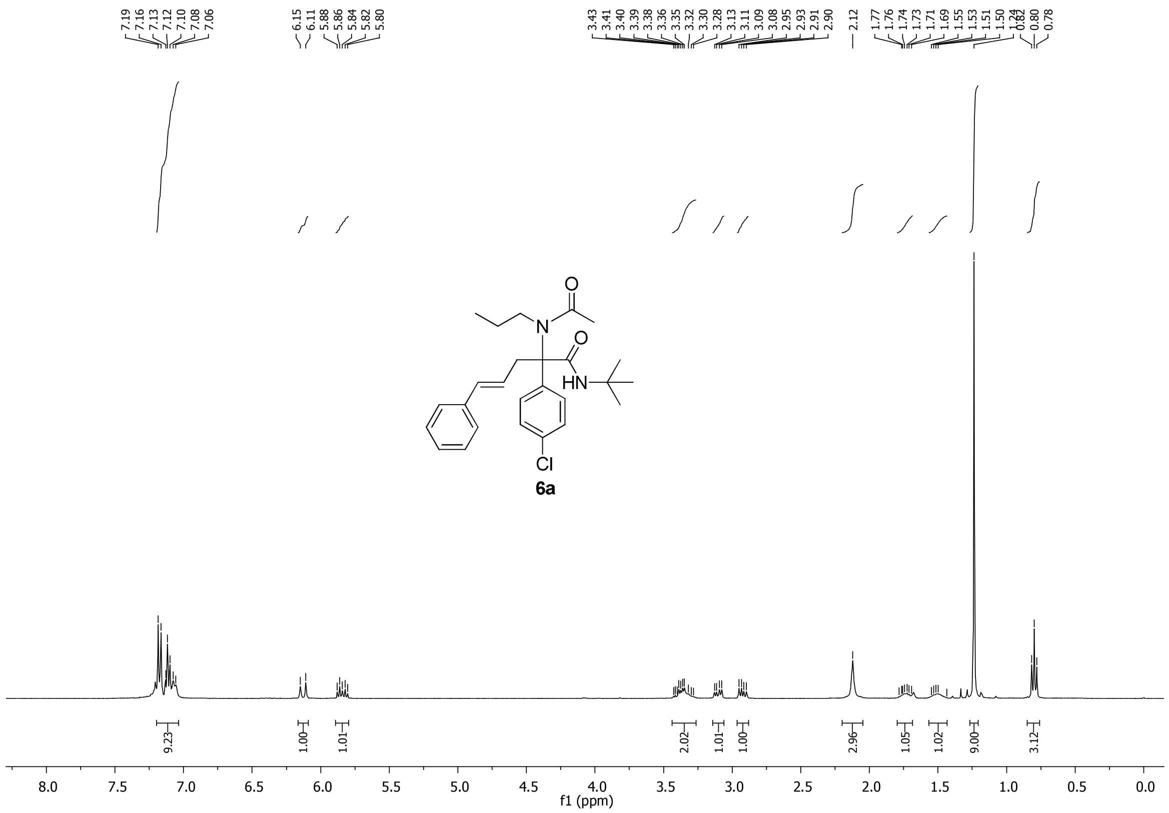
7.19
7.16
7.13
7.12
7.10
7.08
7.06
6.15
6.11
5.88
5.86
5.84
5.82
5.80
3.43
3.41
3.40
3.39
3.38
3.36
3.35
3.32
3.30
3.28
3.13
3.11
3.09
3.08
2.95
2.93
2.91
2.90
2.12
1.77
1.76
1.74
1.73
1.71
1.69
1.55
1.53
1.51
1.50
1.24
0.82
0.80
0.78
Cl
HN
N
O
O
6a
9.23
1.00
1.01
2.02
1.01
1.00
2.96
1.05
1.02
9.00
3.12
8.0
7.5
7.0
6.5
6.0
5.5
5.0
4.5
4.0
3.5
3.0
2.5
2.0
1.5
1.0
0.5
0.0
f1 (ppm)

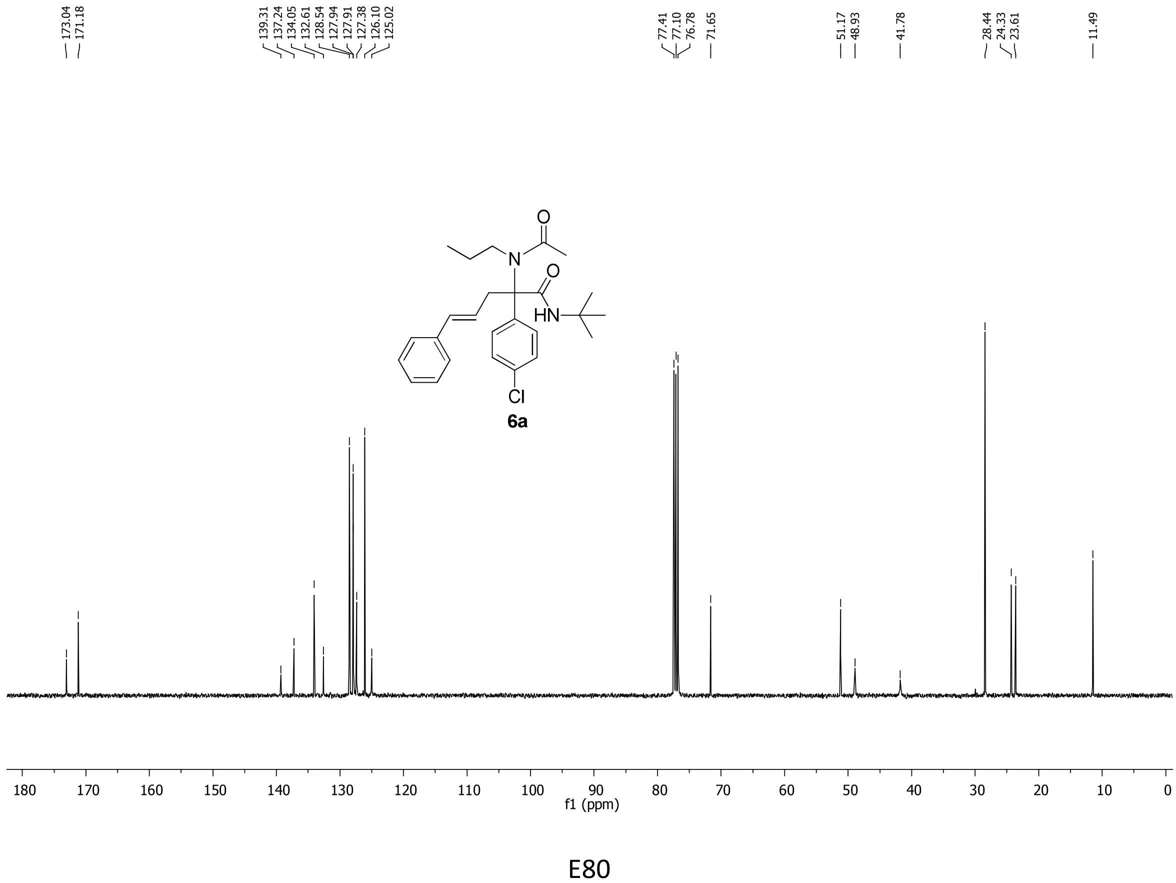
173.04
171.18
139.31
137.24
134.05
132.61
128.54
127.94
127.91
127.38
126.10
125.02
77.41
77.10
76.78
71.65
51.17
48.93
41.78
28.44
24.33
23.61
11.49
Cl
HN
N
O
O
6a
180
170
160
150
140
130
120
110
100
90
80
70
60
50
40
30
20
10
0
f1 (ppm)

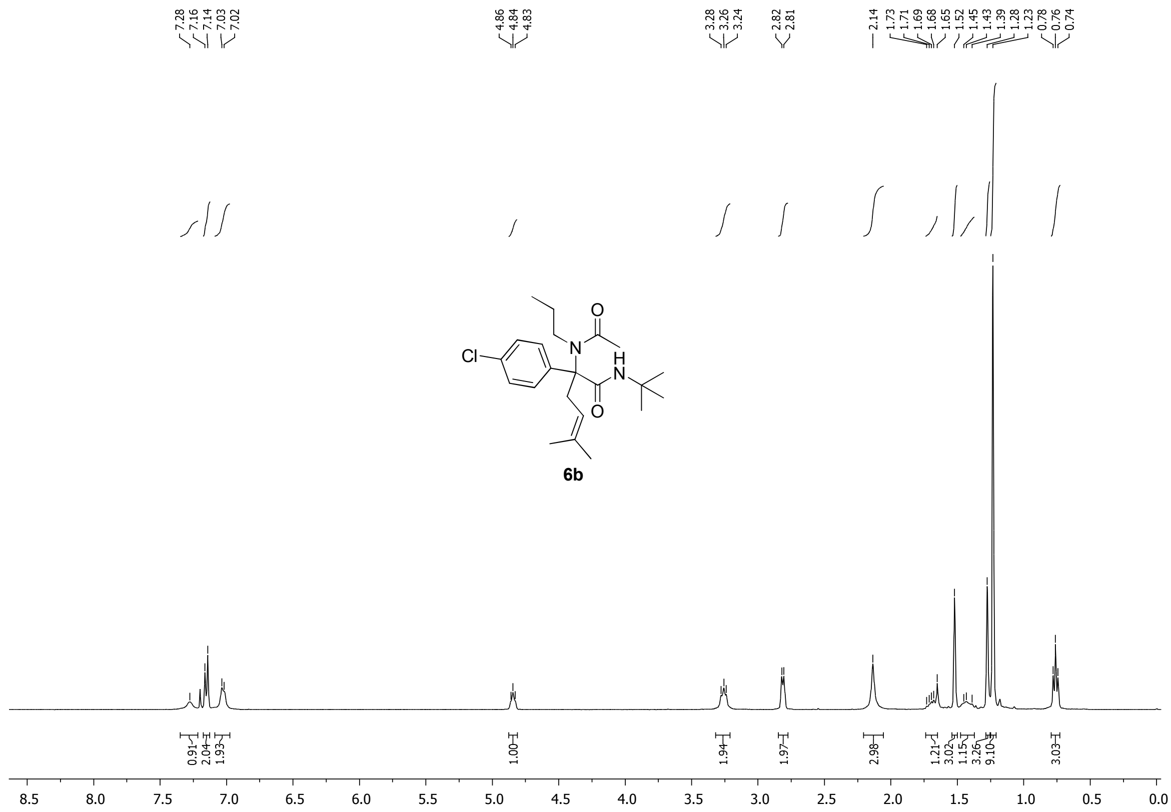
6b

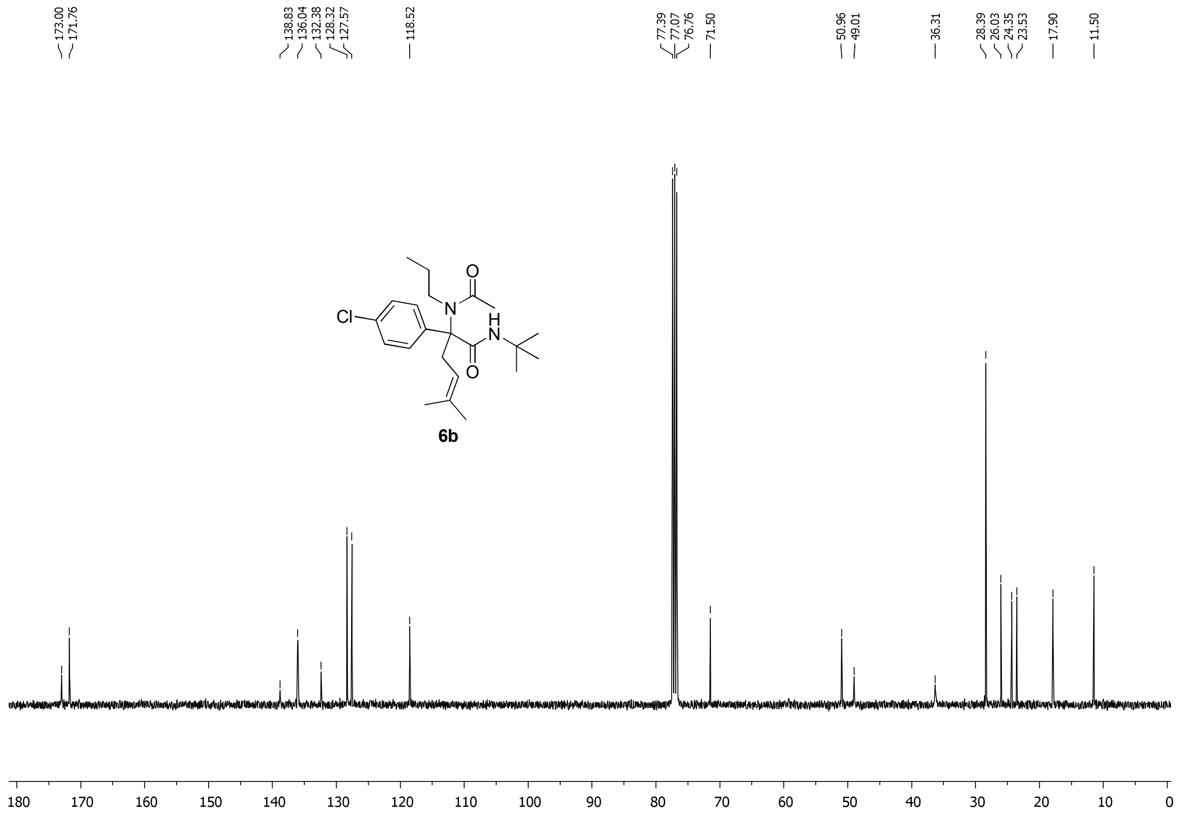
6b

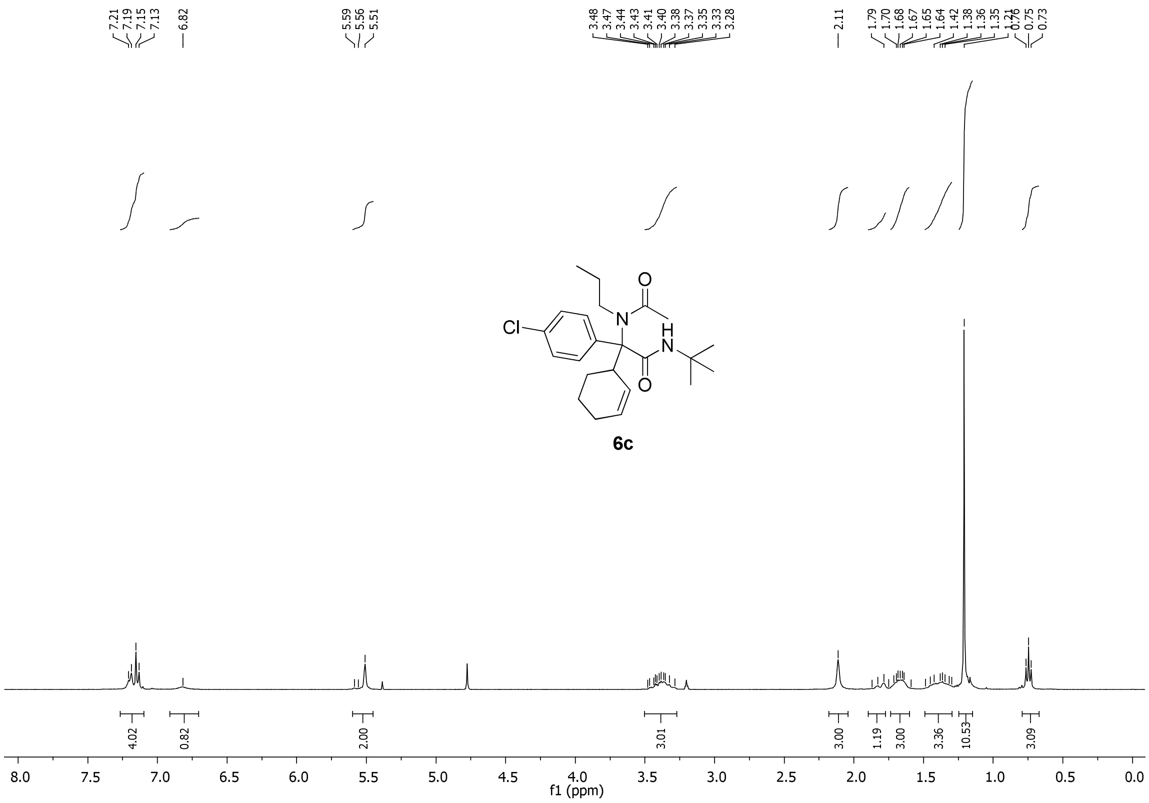
6c
f1 (ppm)
7.21 7.19 7.15 7.13 6.82 5.59 5.56 5.51 3.48 3.47 3.44 3.43 3.41 3.40 3.38 3.37 3.35 3.33 3.28 2.11 1.79 1.70 1.68 1.67 1.65 1.64 1.42 1.38 1.36 1.35 1.21 0.76 0.75 0.73
4.02 0.82 2.00 3.01 3.00 1.19 3.00 3.36 10.53 3.09

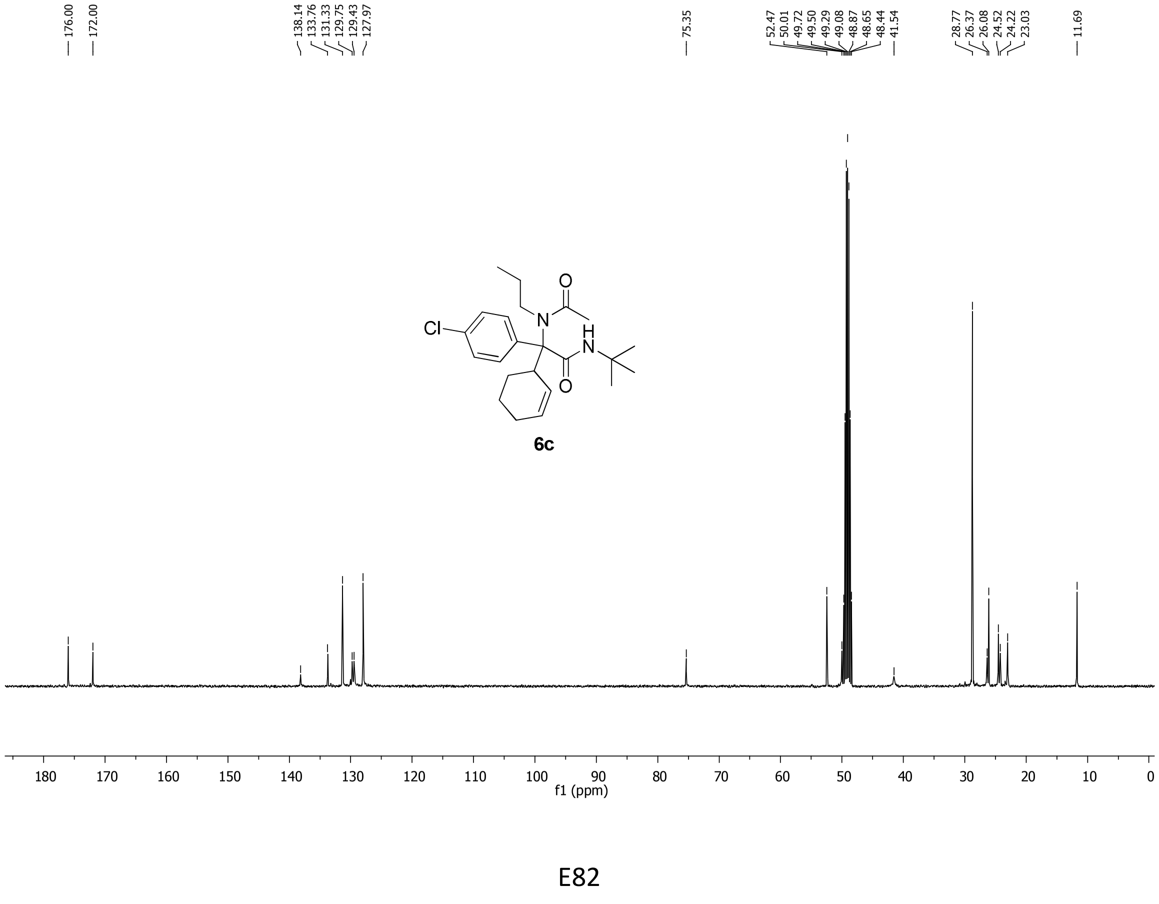
6c
f1 (ppm)
176.00 172.00 138.14 133.76 131.33 129.75 129.43 127.97 75.35 52.47 50.01 49.72 49.50 49.29 49.08 48.87 48.65 48.44 41.54 28.77 26.37 26.08 24.52 24.22 23.03 11.69

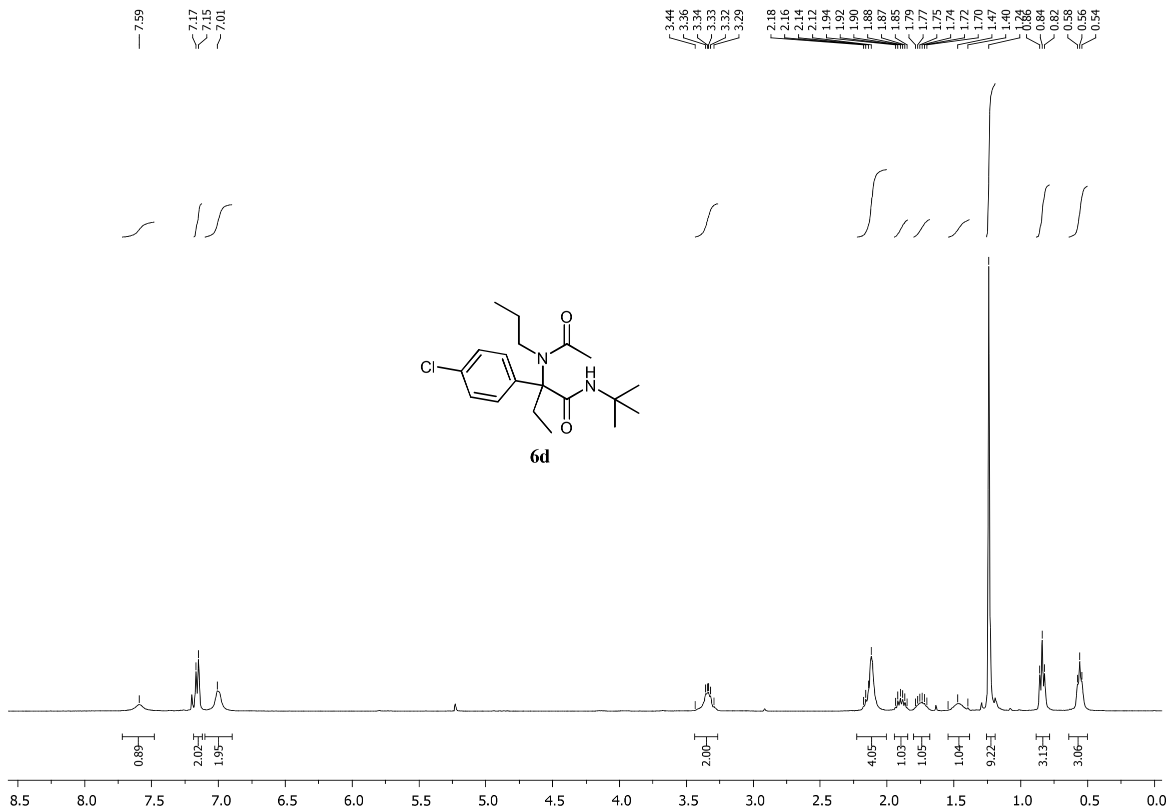
7.59
7.17
7.15
7.01
3.44
3.36
3.34
3.33
3.32
3.29
2.18
2.16
2.14
2.12
1.94
1.92
1.90
1.88
1.87
1.85
1.79
1.77
1.75
1.74
1.72
1.70
1.47
1.40
1.24
0.86
0.84
0.82
0.58
0.56
0.54
Cl
N
O
H
6d
0.89
2.02
1.95
2.00
4.05
1.03
1.05
1.04
9.22
3.13
3.06
8.5 8.0 7.5 7.0 6.5 6.0 5.5 5.0 4.5 4.0 3.5 3.0 2.5 2.0 1.5 1.0 0.5 0.0

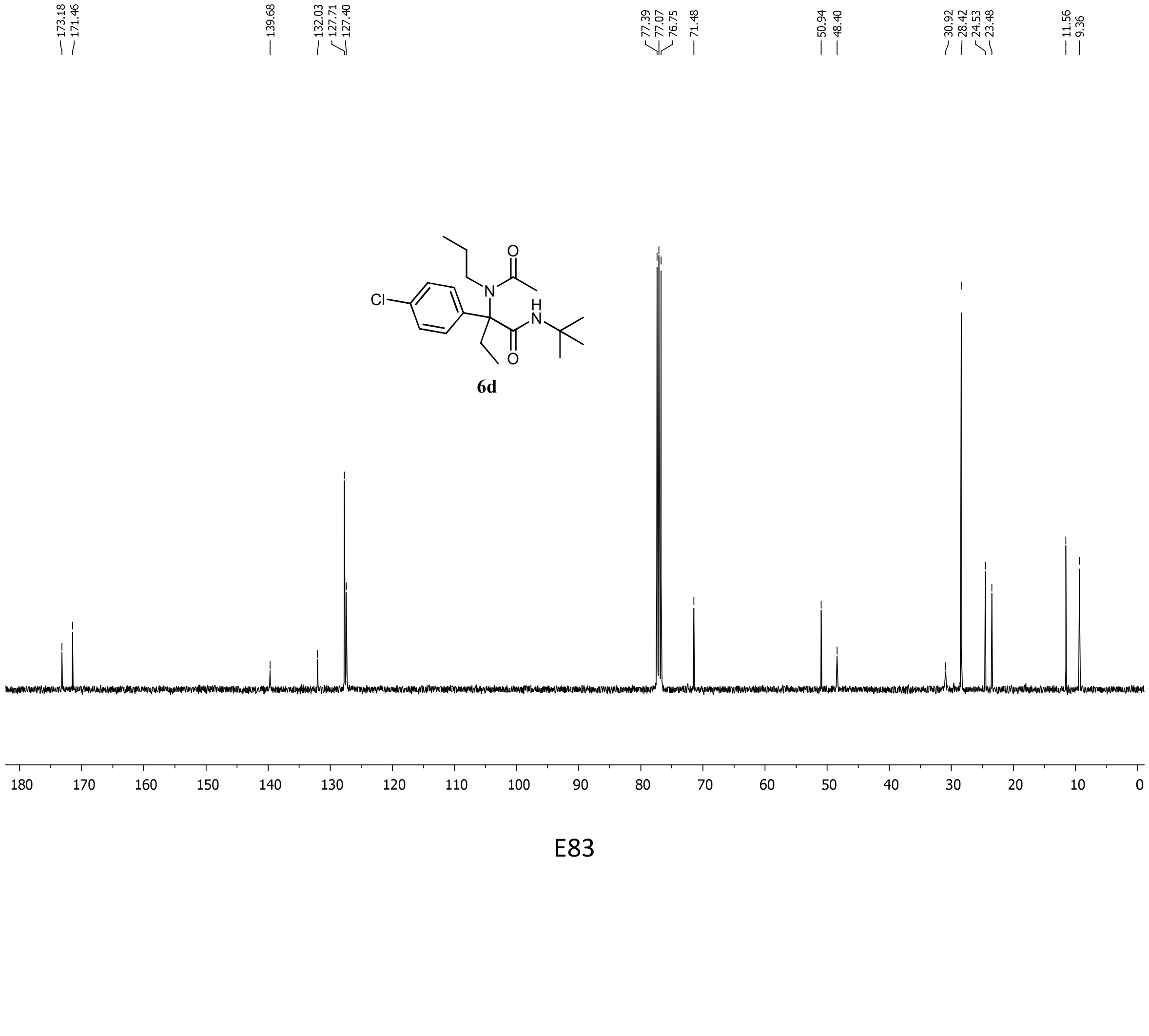
173.18
171.46
139.68
132.03
127.71
127.40
77.39
77.07
76.75
71.48
50.94
48.40
30.92
28.42
24.53
23.48
11.56
9.36
Cl
N
O
H
6d
180 170 160 150 140 130 120 110 100 90 80 70 60 50 40 30 20 10 0

E83

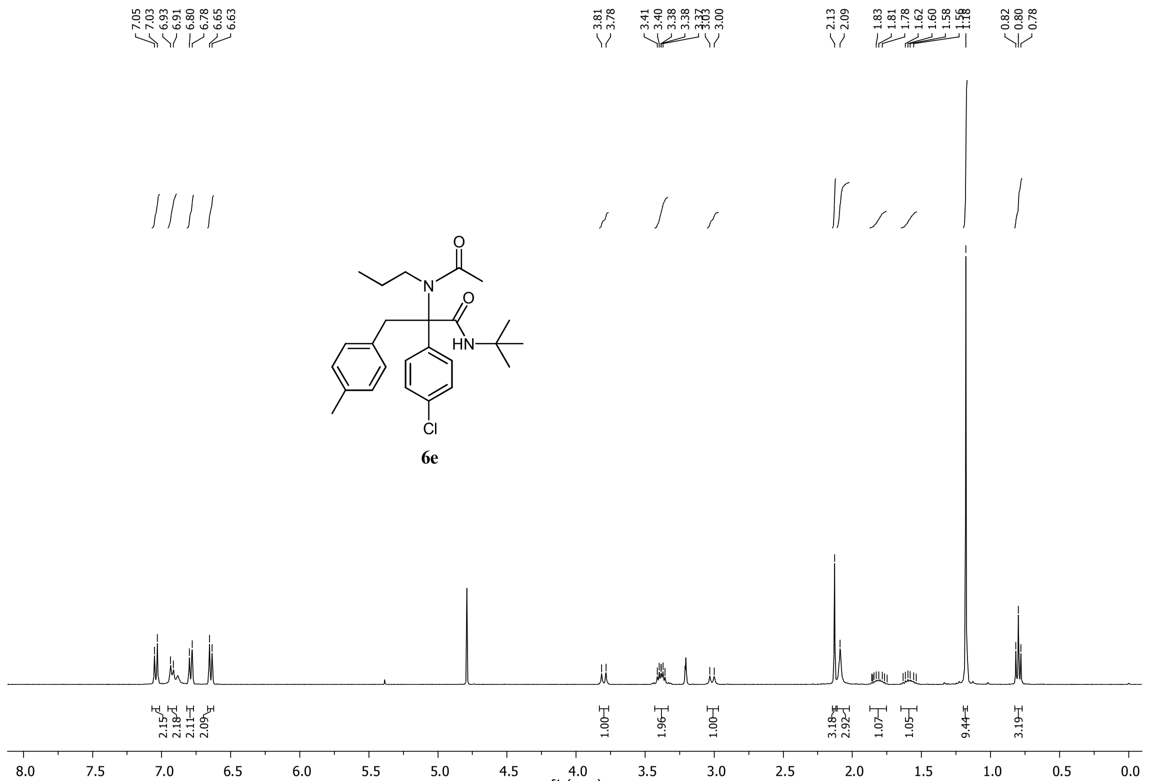
7.05
7.03
6.93
6.91
6.80
6.78
6.65
6.63
3.81
3.78
3.41
3.40
3.38
3.38
3.37
3.03
3.00
2.13
2.09
1.83
1.81
1.78
1.62
1.60
1.58
1.56
1.18
0.82
0.80
0.78
6e
2.15
2.18
2.11
2.09
1.00
1.96
1.00
3.18
2.92
1.07
1.05
9.44
3.19
f1 (ppm)

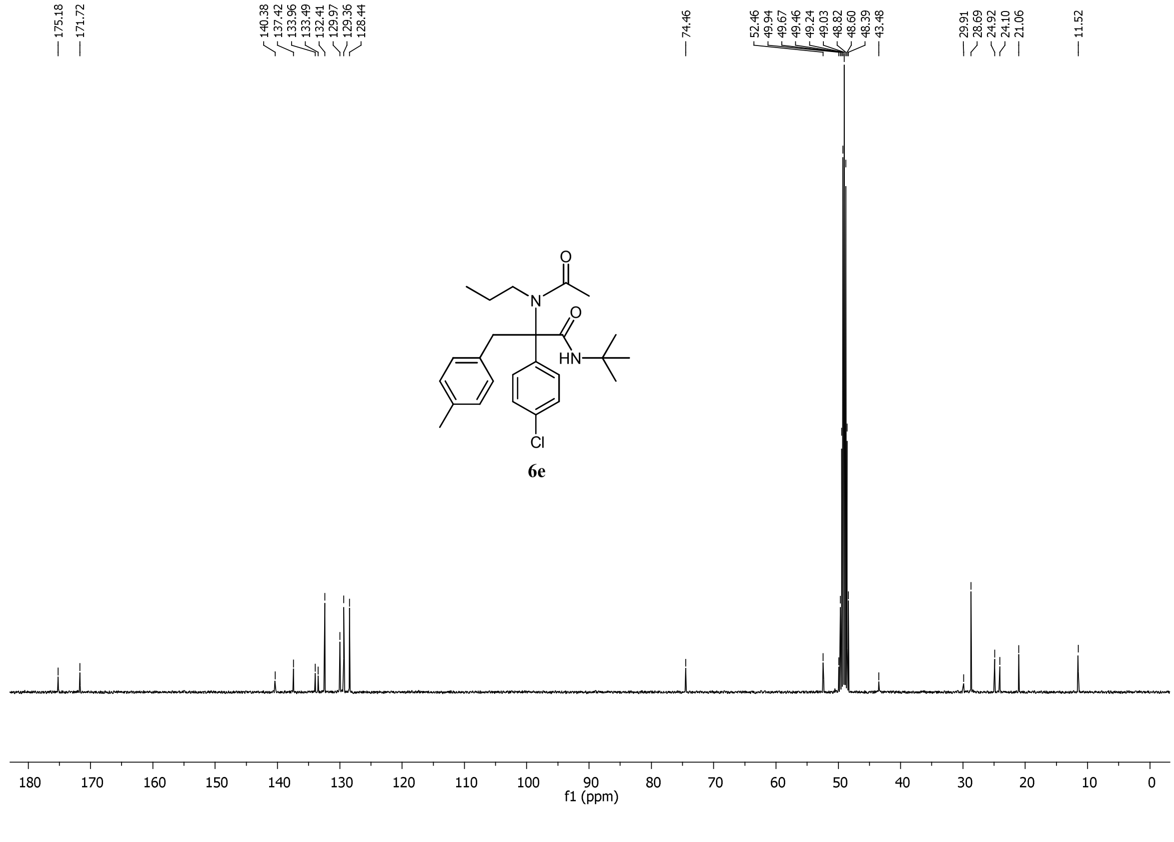
175.18
171.72
140.38
137.42
133.96
133.49
132.41
129.97
129.36
128.44
74.46
52.46
49.94
49.67
49.46
49.24
49.03
48.82
48.60
48.39
43.48
29.91
28.69
24.92
24.10
21.06
11.52
6e
f1 (ppm)

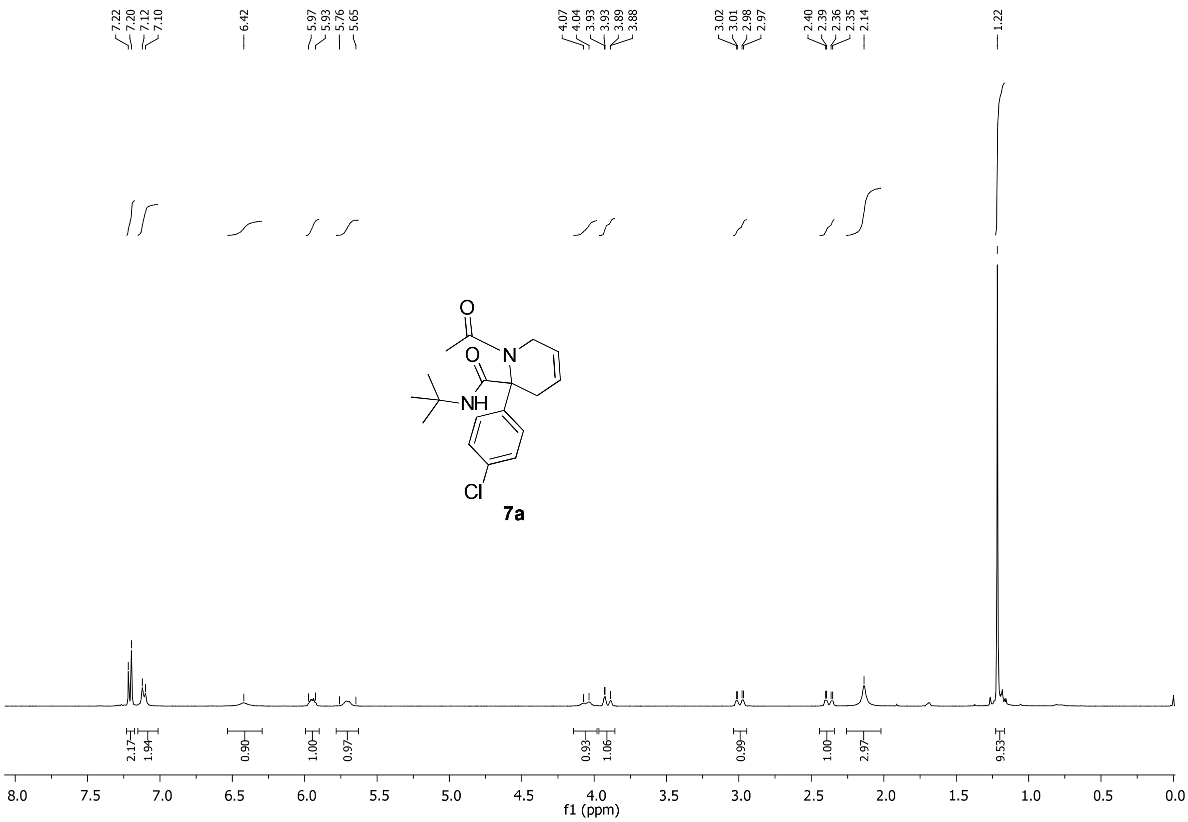
7.22
7.20
7.12
7.10
6.42
5.97
5.93
5.76
5.65
4.07
4.04
3.93
3.93
3.89
3.88
3.02
3.01
2.98
2.97
2.40
2.39
2.36
2.35
2.14
1.22
O
N
O
NH
Cl
7a
2.17
1.94
0.90
1.00
0.97
0.93
1.06
0.99
1.00
2.97
9.53
f1 (ppm)

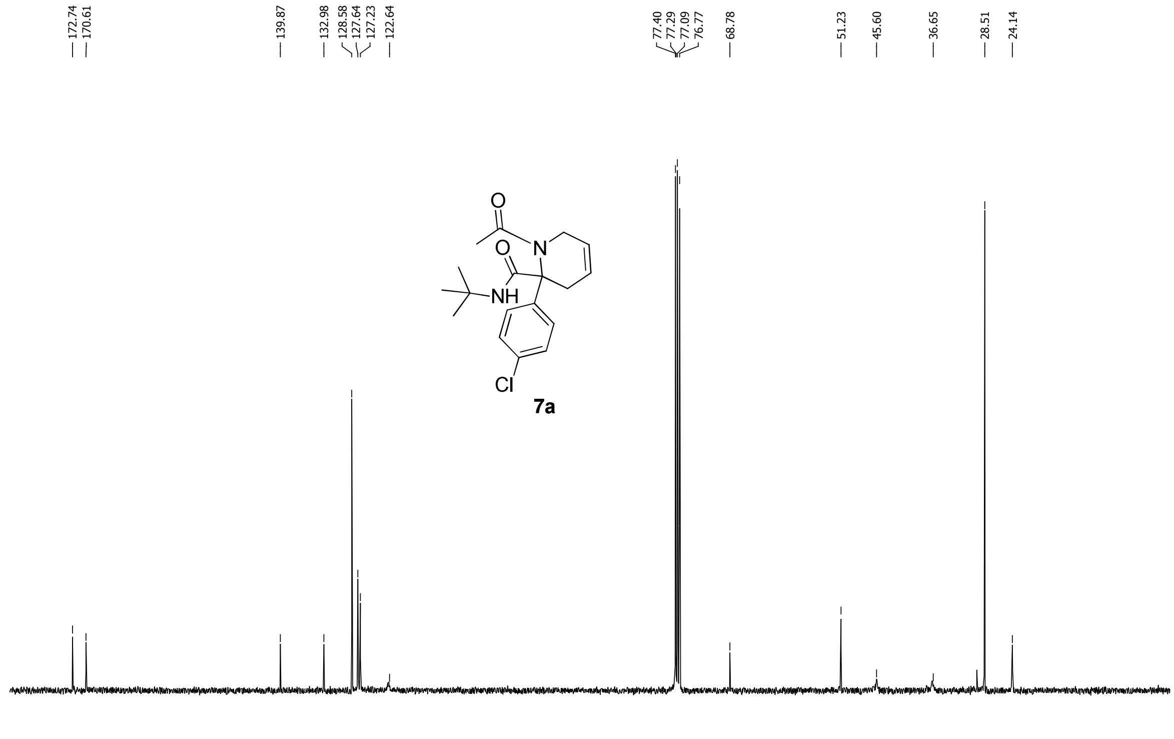
172.74
170.61
139.87
132.98
128.58
127.64
127.23
122.64
77.40
77.29
77.09
76.77
68.78
51.23
45.60
36.65
28.51
24.14
O
N
O
NH
Cl
7a
f1 (ppm)

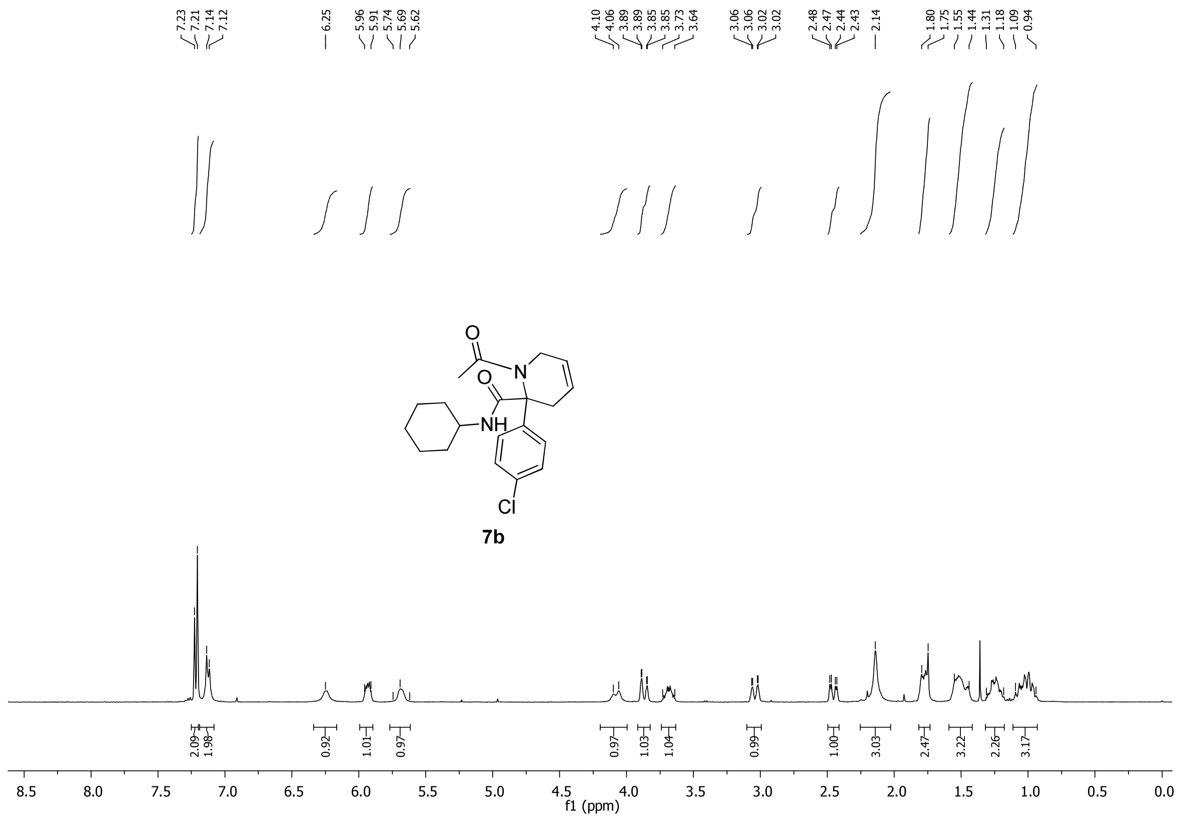
7.23
7.21
7.14
7.12
6.25
5.96
5.91
5.74
5.69
5.62
4.10
4.06
3.89
3.89
3.85
3.85
3.73
3.64
3.06
3.06
3.02
3.02
2.48
2.47
2.44
2.43
2.14
1.80
1.75
1.55
1.44
1.31
1.18
1.09
0.94
O
N
O
NH
Cl
7b
2.09
1.98
0.92
1.01
0.97
0.97
1.03
1.04
0.99
1.00
3.03
2.47
3.22
2.26
3.17
8.5
8.0
7.5
7.0
6.5
6.0
5.5
5.0
4.5
4.0
3.5
3.0
2.5
2.0
1.5
1.0
0.5
0.0
f1 (ppm)

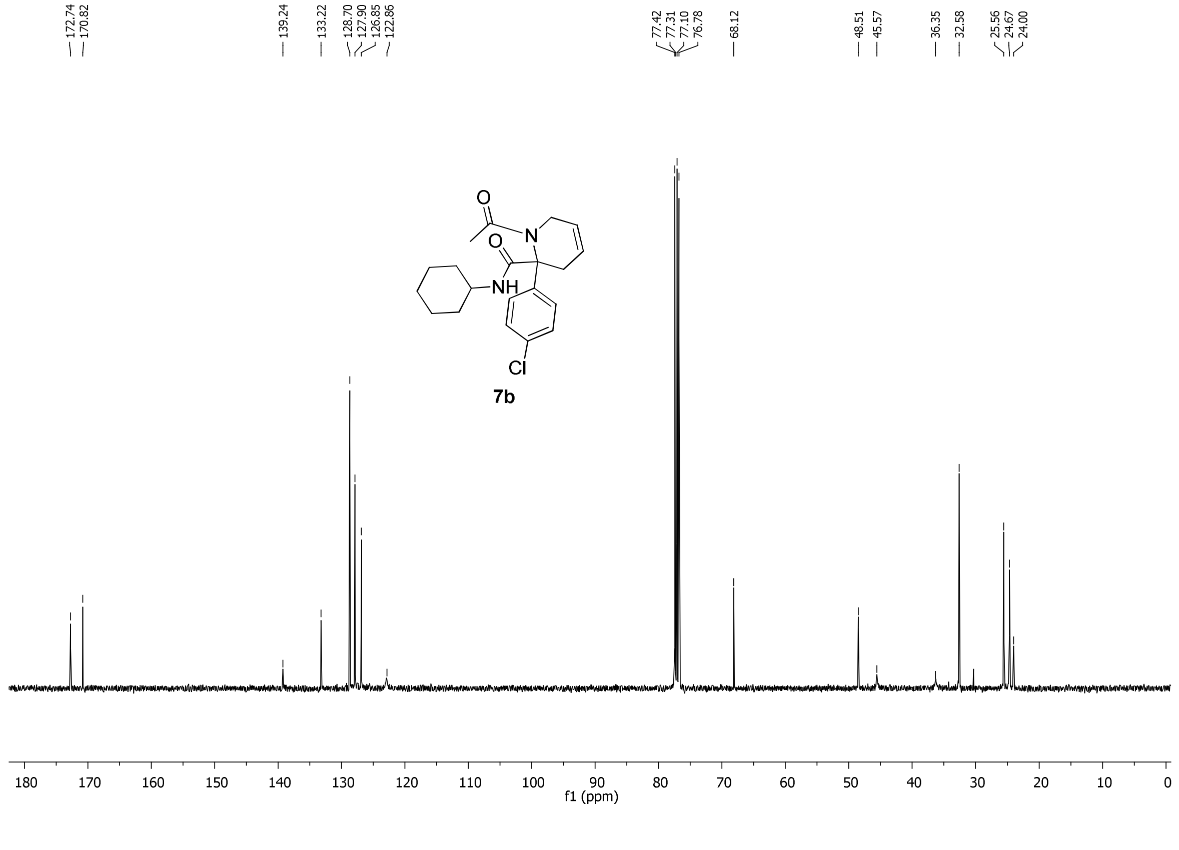
172.74
170.82
139.24
133.22
128.70
127.90
126.85
122.86
77.42
77.31
77.10
76.78
68.12
48.51
45.57
36.35
32.58
25.56
24.67
24.00
O
N
O
NH
Cl
7b
180
170
160
150
140
130
120
110
100
90
80
70
60
50
40
30
20
10
0
f1 (ppm)

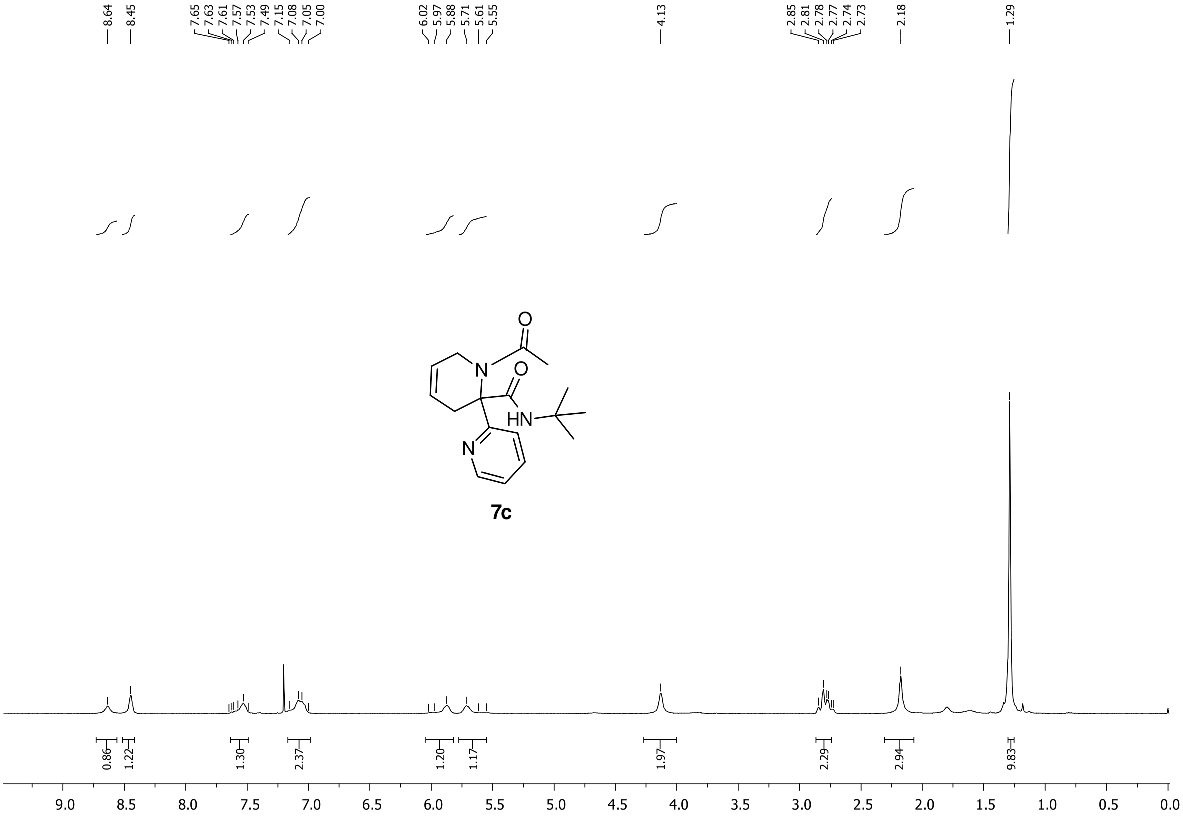
8.64
8.45
7.65
7.63
7.61
7.57
7.53
7.49
7.15
7.08
7.05
7.00
6.02
5.97
5.88
5.71
5.61
5.55
4.13
2.85
2.81
2.78
2.77
2.74
2.73
2.18
1.29
0.86
1.22
1.30
2.37
1.20
1.17
1.97
2.29
2.94
9.83
7c
9.0 8.5 8.0 7.5 7.0 6.5 6.0 5.5 5.0 4.5 4.0 3.5 3.0 2.5 2.0 1.5 1.0 0.5 0.0

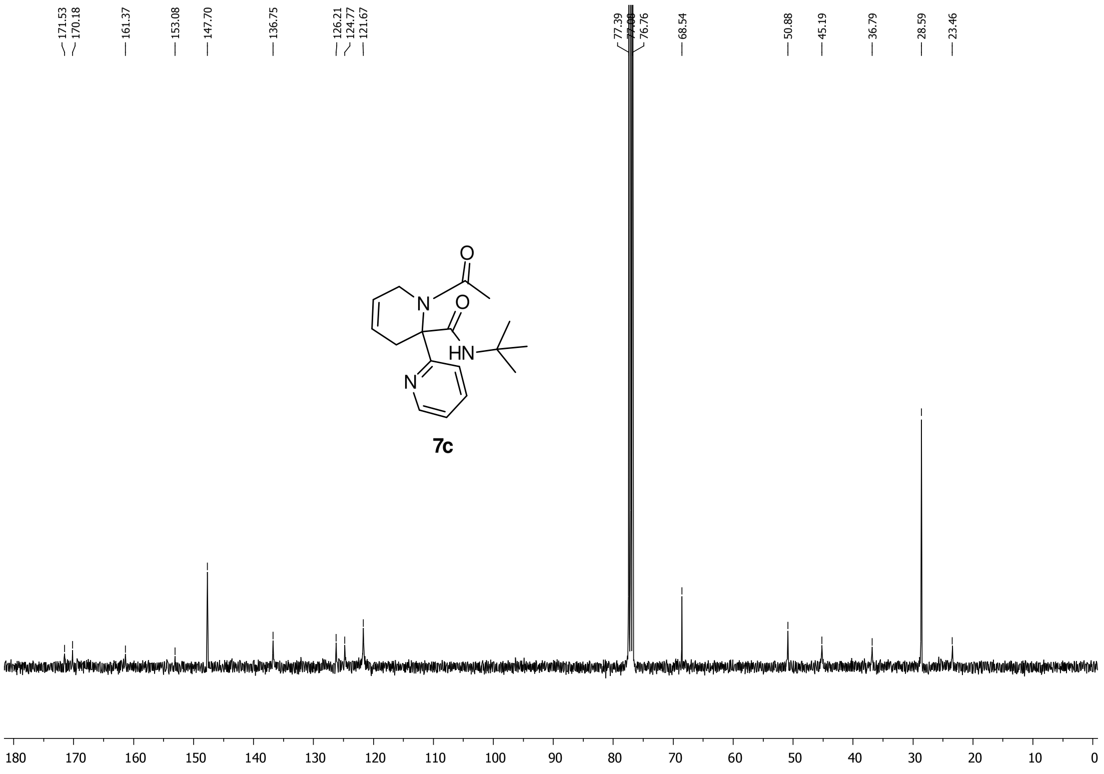
171.53
170.18
161.37
153.08
147.70
136.75
126.21
124.77
121.67
77.39
76.76
68.54
50.88
45.19
36.79
28.59
23.46
7c
180 170 160 150 140 130 120 110 100 90 80 70 60 50 40 30 20 10 0

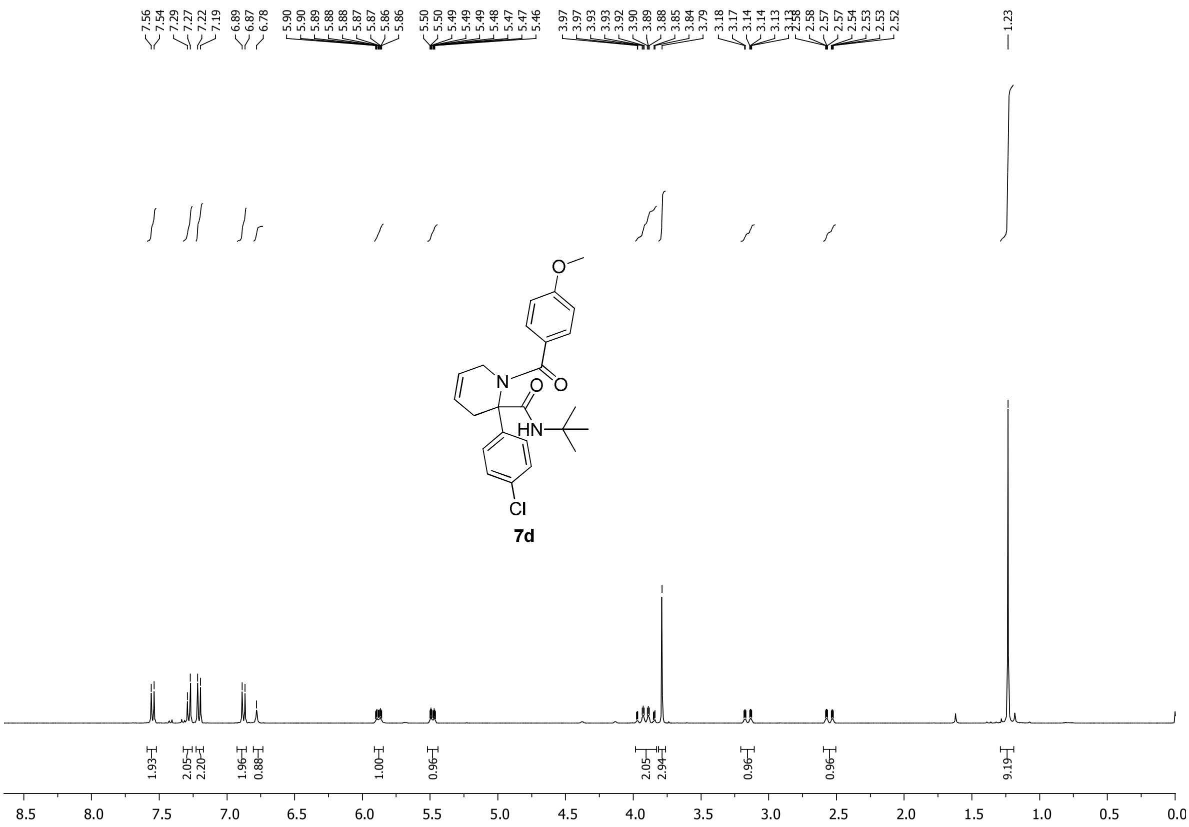
7.56
7.54
7.29
7.27
7.22
7.19
6.89
6.87
6.78
5.90
5.90
5.89
5.88
5.88
5.87
5.87
5.86
5.86
5.50
5.50
5.49
5.49
5.49
5.48
5.47
5.47
5.46
3.97
3.97
3.93
3.93
3.92
3.90
3.89
3.88
3.85
3.84
3.79
3.18
3.17
3.14
3.14
3.13
3.13
2.58
2.58
2.57
2.57
2.54
2.53
2.53
2.52
1.23
N
O
O
O
HN
Cl
1.93
2.05
2.20
1.96
0.88
1.00
0.96
2.05
2.94
0.96
0.96
9.19
8.5
8.0
7.5
7.0
6.5
6.0
5.5
5.0
4.5
4.0
3.5
3.0
2.5
2.0
1.5
1.0
0.5
0.0

7d

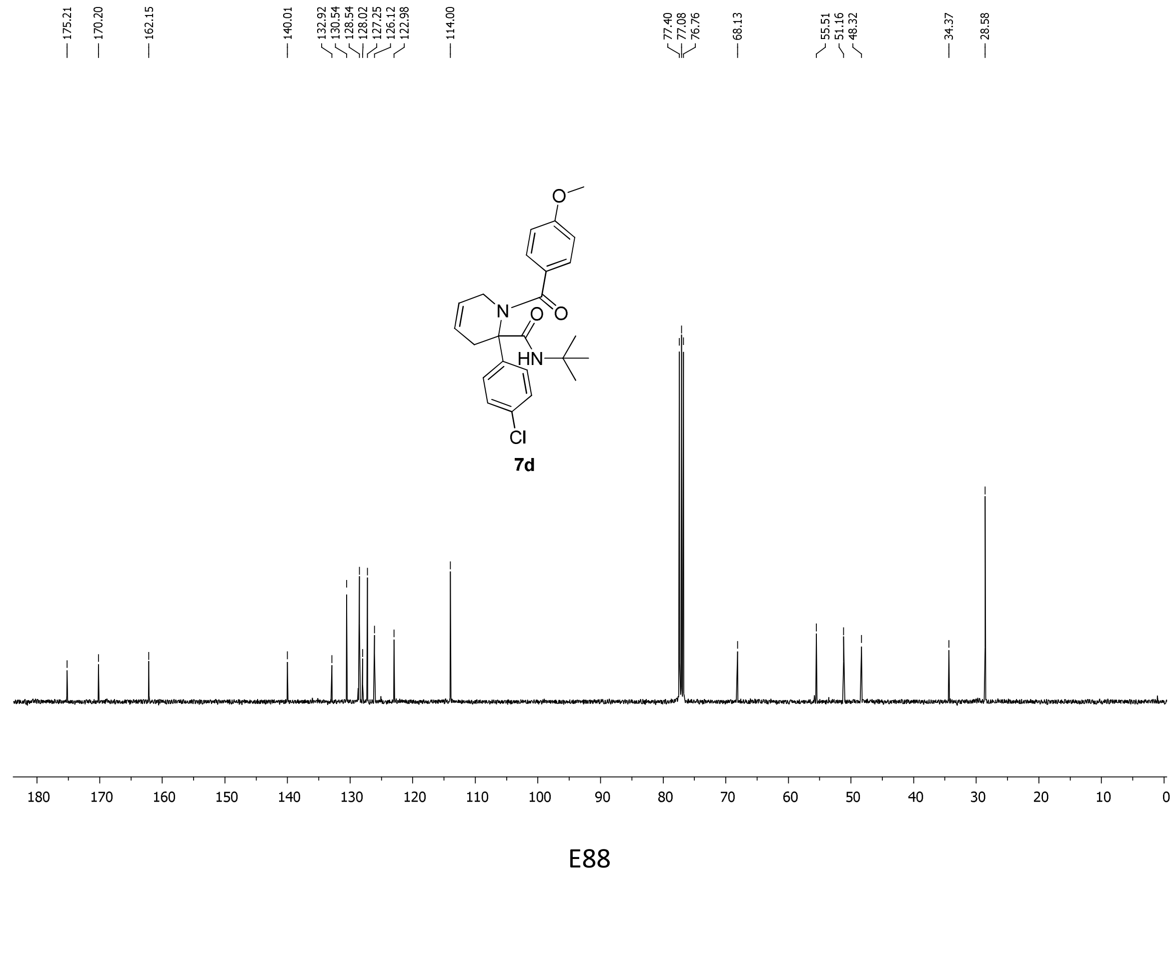
175.21
170.20
162.15
140.01
132.92
130.54
128.54
128.02
127.25
126.12
122.98
114.00
77.40
77.08
76.76
68.13
55.51
51.16
48.32
34.37
28.58
N
O
O
O
HN
Cl
180
170
160
150
140
130
120
110
100
90
80
70
60
50
40
30
20
10
0

7d

E88

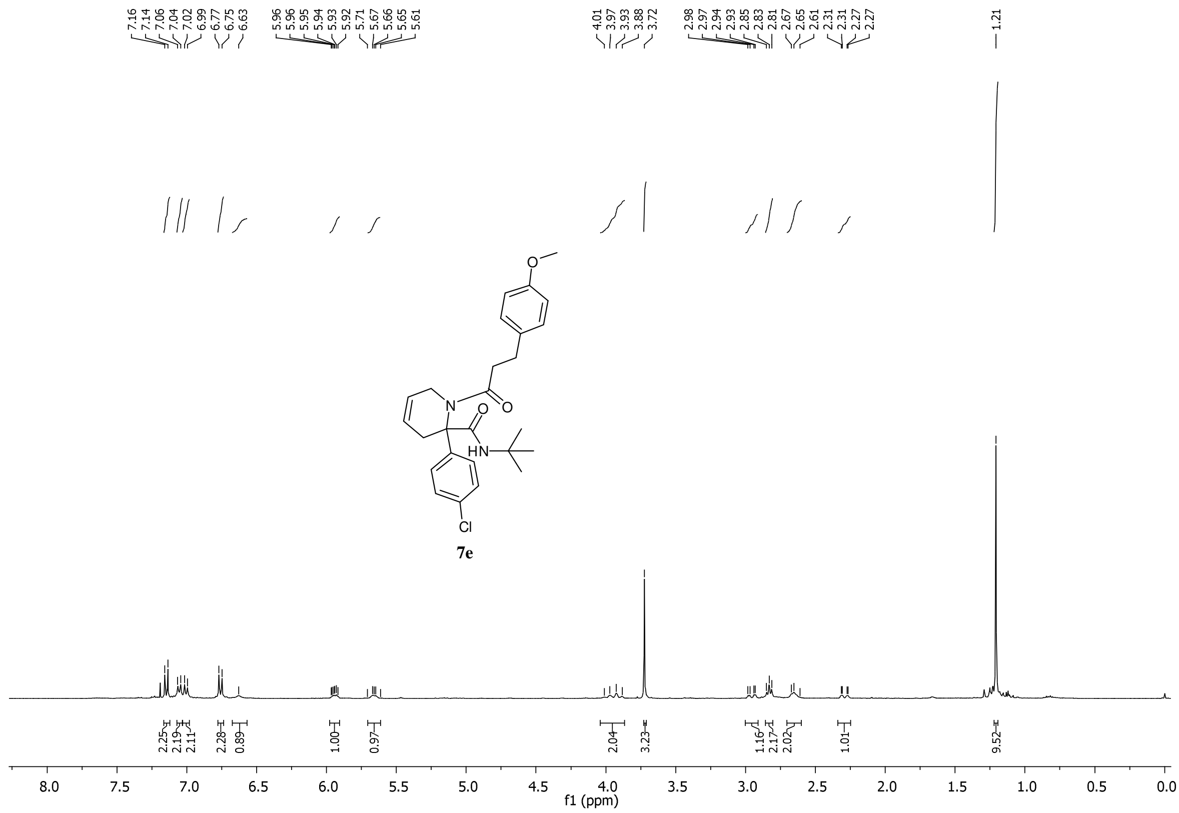

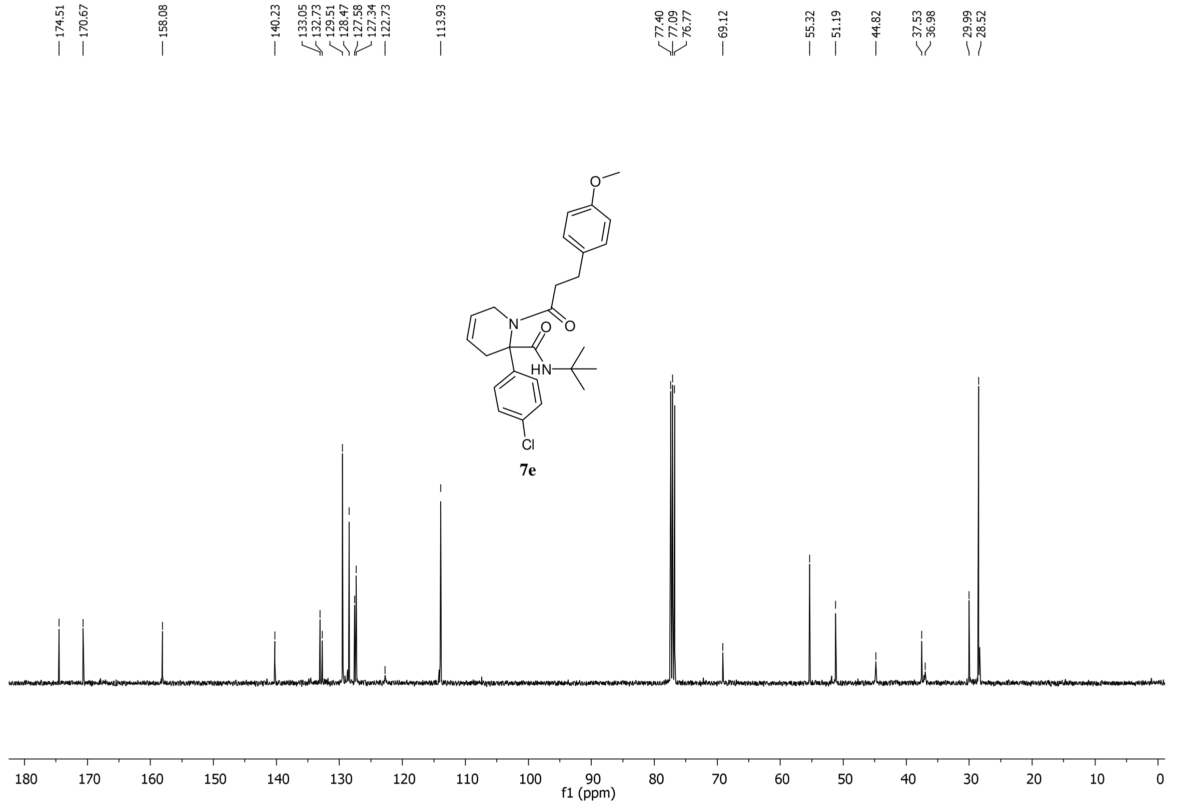

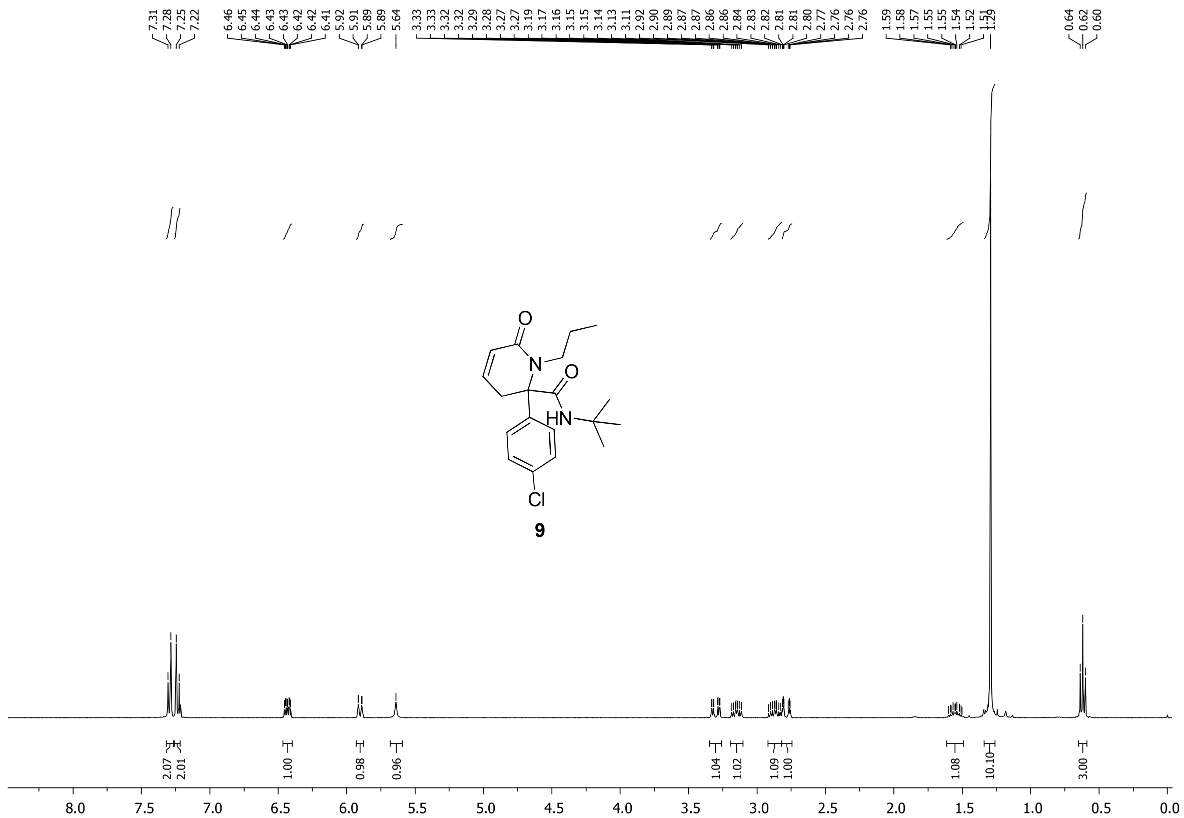
7.31
7.28
7.25
7.22
6.46
6.45
6.44
6.43
6.43
6.42
6.42
6.41
5.92
5.91
5.89
5.89
5.64
3.33
3.33
3.32
3.32
3.29
3.28
3.27
3.27
3.19
3.17
3.16
3.15
3.15
3.14
3.13
3.11
2.92
2.90
2.89
2.87
2.87
2.86
2.86
2.84
2.83
2.82
2.81
2.81
2.80
2.77
2.76
2.76
2.76
1.59
1.58
1.57
1.55
1.55
1.54
1.52
1.51
1.29
0.64
0.62
0.60
O
N
O
HN
Cl
9
2.07
2.01
1.00
0.98
0.96
1.04
1.02
1.09
1.00
1.08
10.10
3.00
8.0
7.5
7.0
6.5
6.0
5.5
5.0
4.5
4.0
3.5
3.0
2.5
2.0
1.5
1.0
0.5
0.0

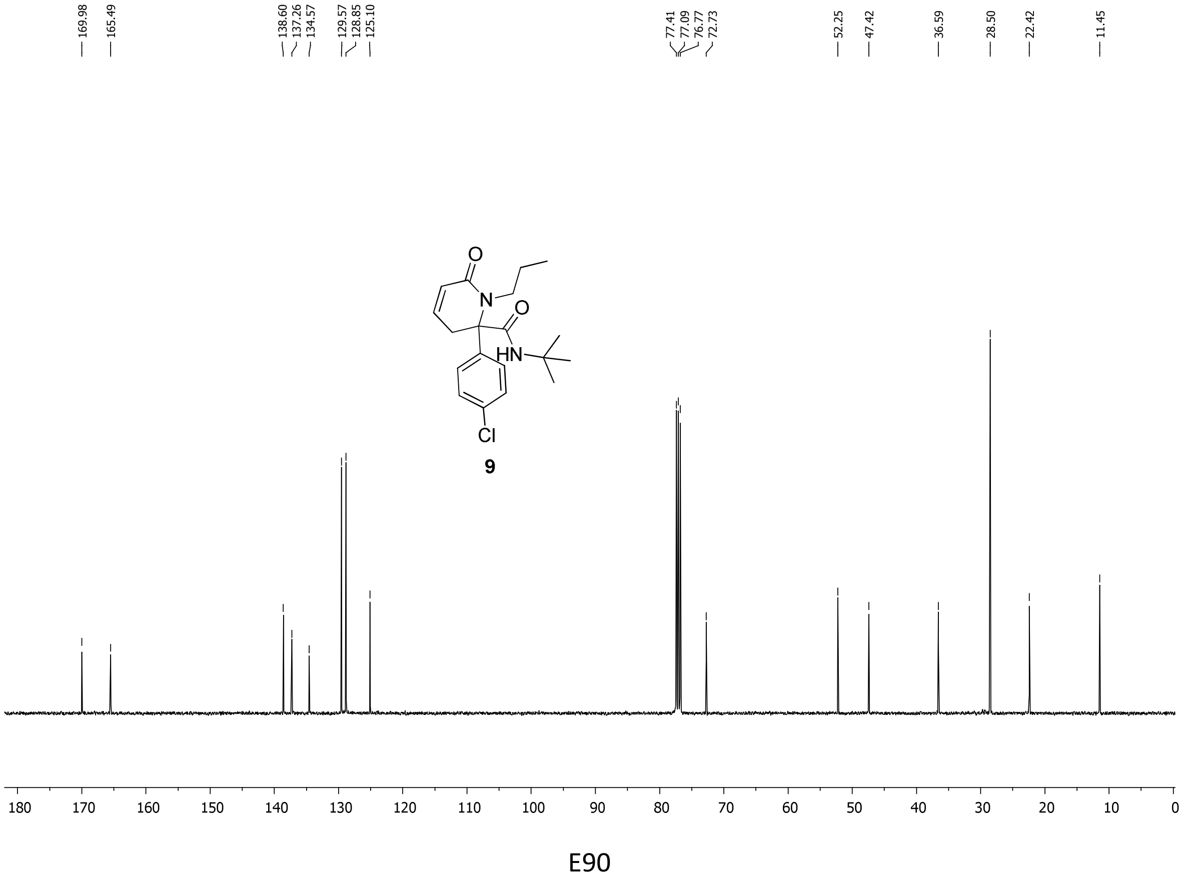
169.98
165.49
138.60
137.26
134.57
129.57
128.85
125.10
77.41
77.09
76.77
72.73
52.25
47.42
36.59
28.50
22.42
11.45
O
N
O
HN
Cl
9
180
170
160
150
140
130
120
110
100
90
80
70
60
50
40
30
20
10
0

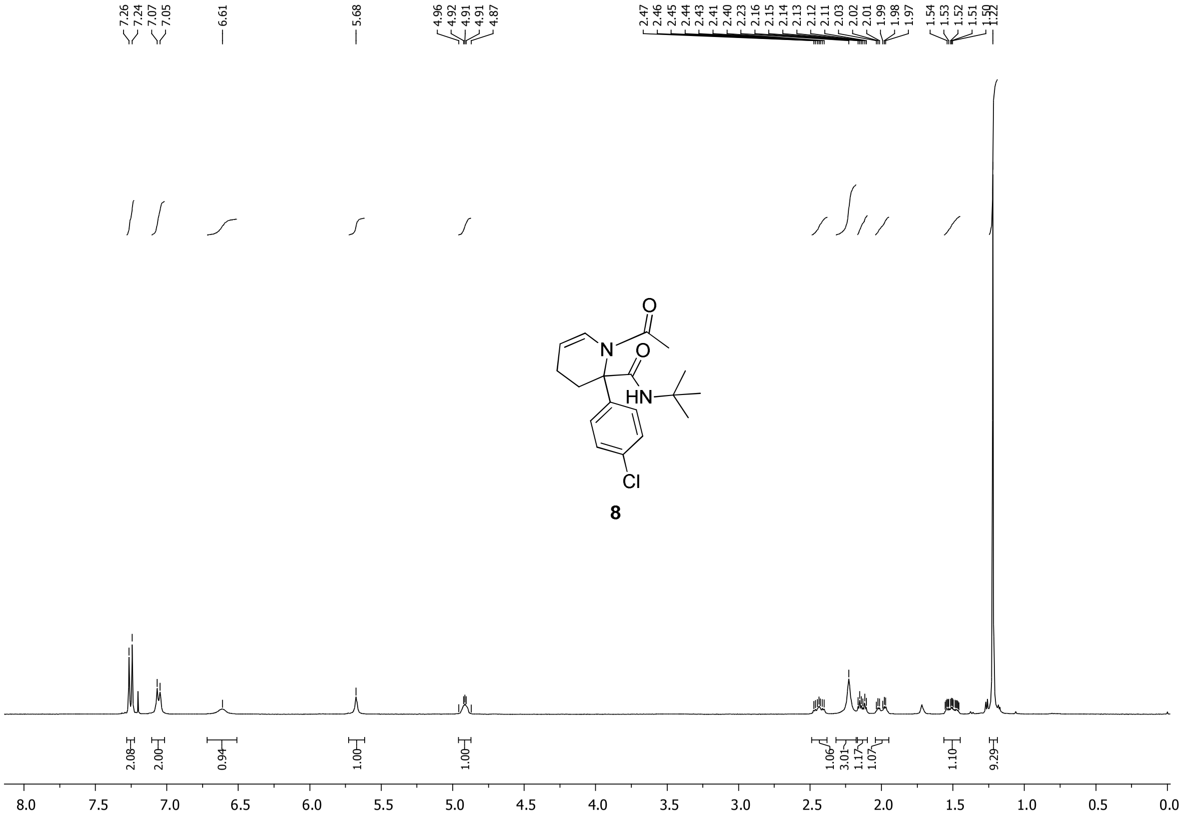

7.26
7.24
7.07
7.05
6.61
5.68
4.96
4.92
4.91
4.91
4.87
2.47
2.46
2.45
2.44
2.43
2.41
2.40
2.23
2.16
2.15
2.14
2.13
2.12
2.11
2.03
2.02
2.01
1.99
1.98
1.97
1.54
1.53
1.52
1.51
1.50
1.22
N
O
HN
O
Cl
8
2.08
2.00
0.94
1.00
1.00
1.06
3.01
1.17
1.07
1.10
9.29
8.0 7.5 7.0 6.5 6.0 5.5 5.0 4.5 4.0 3.5 3.0 2.5 2.0 1.5 1.0 0.5 0.0

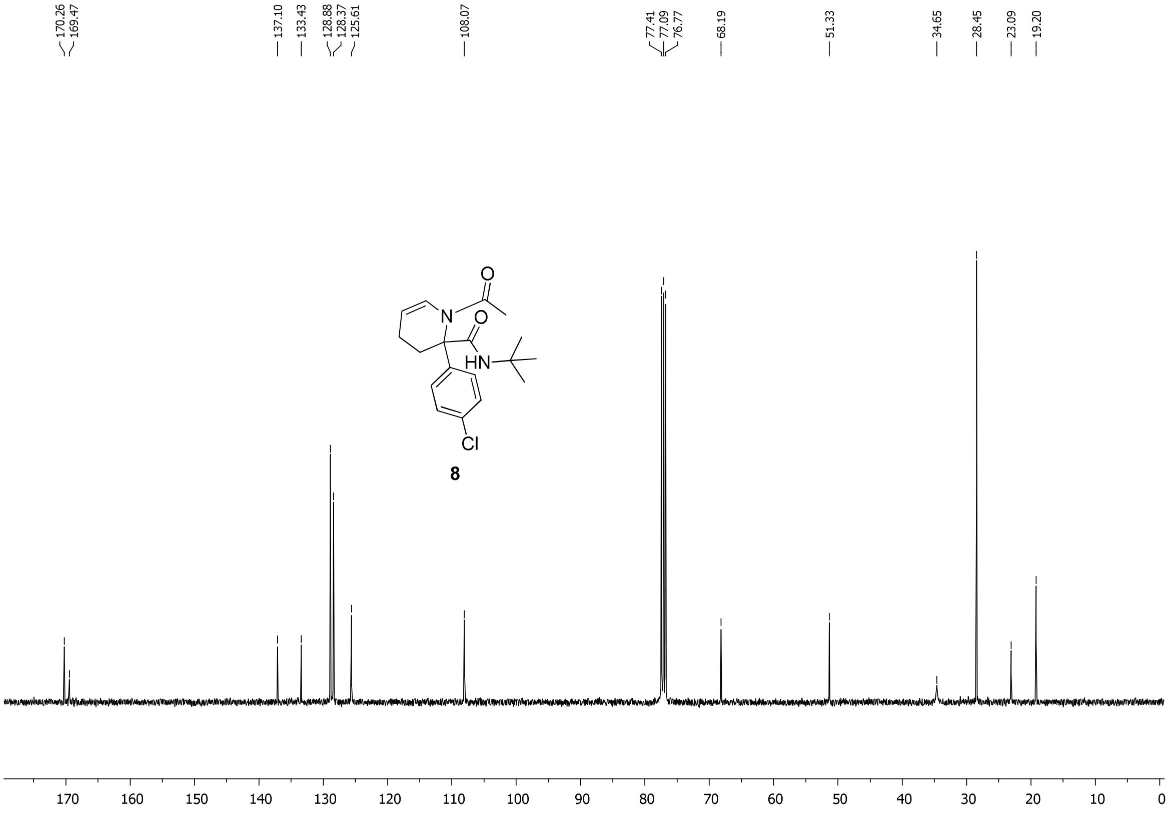

170.26
169.47
137.10
133.43
128.88
128.37
125.61
108.07
77.41
77.09
76.77
68.19
51.33
34.65
28.45
23.09
19.20
N
O
HN
O
Cl
8
170 160 150 140 130 120 110 100 90 80 70 60 50 40 30 20 10 0

Laboratoire de Synthèse Organique

09/06/2016 09:11:35

File: ZA86
Sample:

Date Run: 09-06-2016 (Time Run: 08:59:38)
Instrument: JEOL JMSGCmateII
Inlet: Direct Probe

Run By: Vincent Jactel
Ionization mode: EI+

324,1603 : 3 % **Scan: 333** **TIC: 18843230**

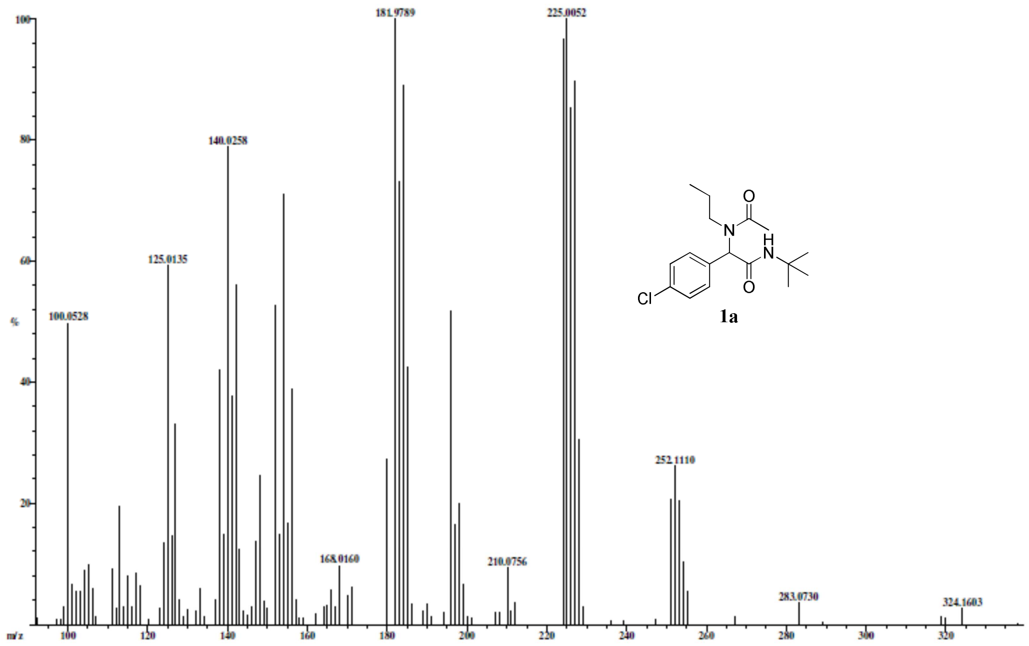

Laboratoire de Synthèse Organique, Palaiseau, France

08/08/2016 10:32:31

File: ZA82
Sample:

Date Run: 08-08-2016 (Time Run: 10:21:03)
Instrument: JEOL JMSGCmateII
Inlet: Direct Probe

Run By: Vincent Jactel
Ionization mode: EI+

C17H25N3O4 335,1837 : 1,5 % **Scan: 70** **TIC: 13367404**

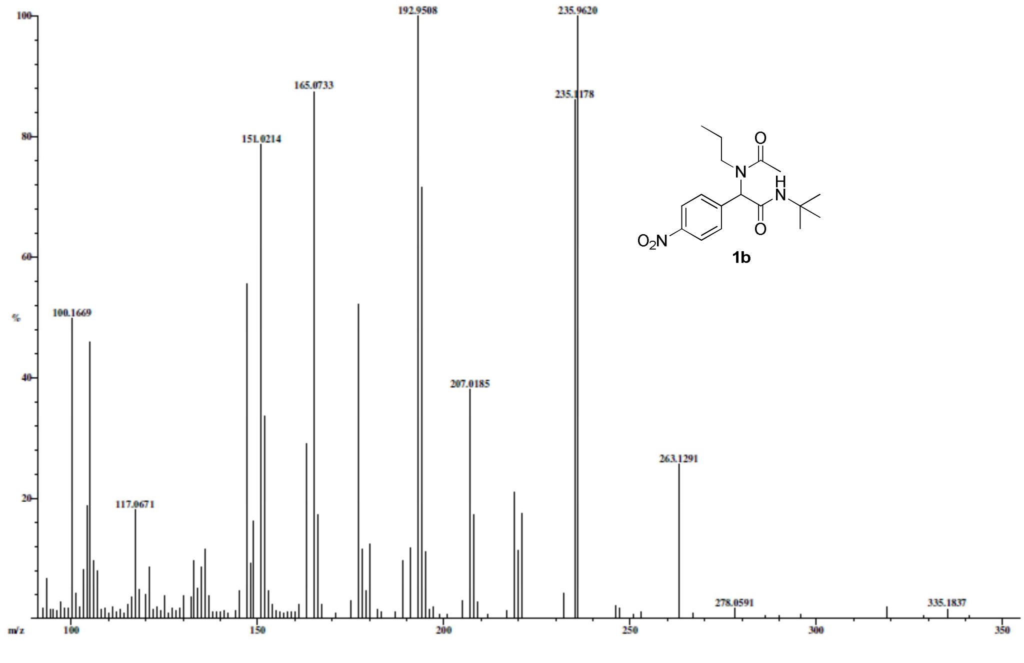

Laboratoire de Synthèse Organique

20/05/2016 17:19:52

File: ZA129 Date Run: 05-20-2016 (Time Run: 17:12:26) Run By: Vincent Jactel
Sample: Instrument: JEOL JMSGCmateII Inlet: Direct Probe Ionization mode: EI+

Scan: 116 **TIC: 15666316**

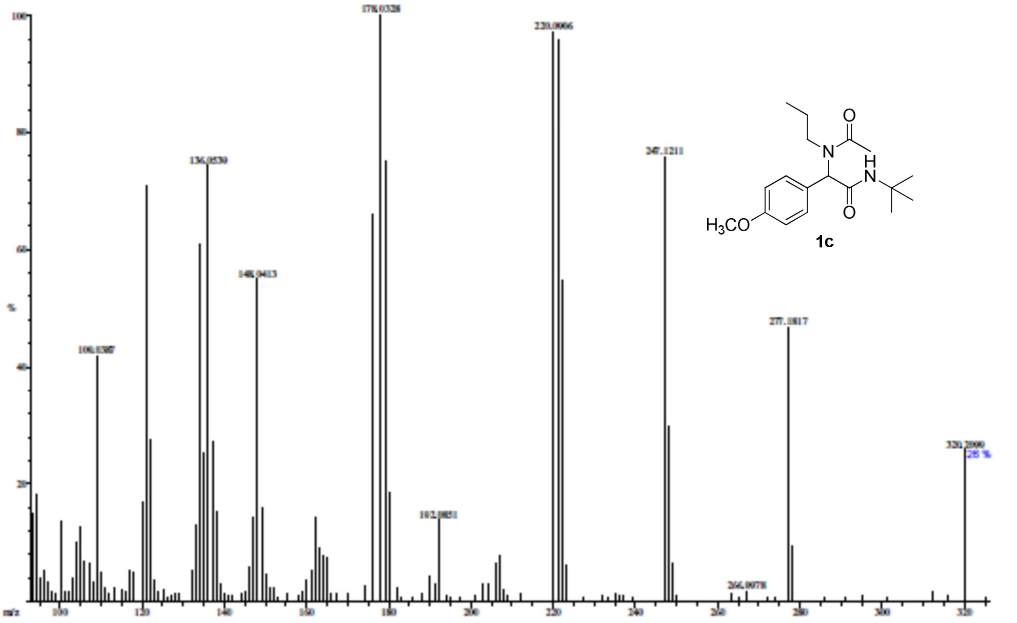

Laboratoire de Synthèse Organique

24/05/2016 09:58:39

File: ZA110 Date Run: 05-24-2016 (Time Run: 09:45:29) Run By: Vincent Jactel
Sample: Instrument: JEOL JMSGCmateII Inlet: Direct Probe Ionization mode: EI+

Scan: 294 **TIC: 13252728**

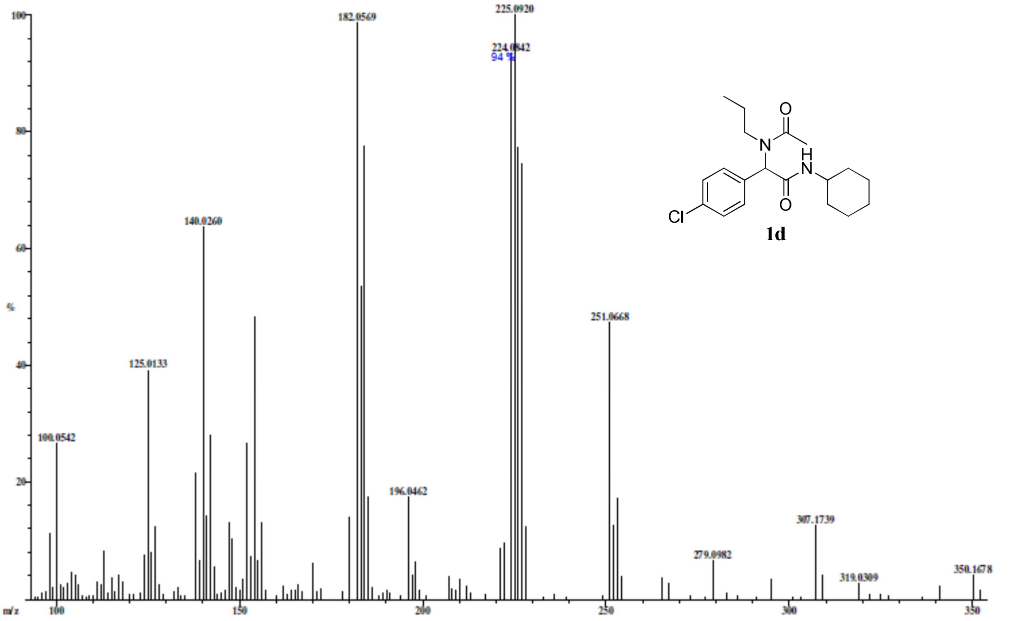

Laboratoire de Synthèse Organique

08/06/2016 17:48:08

File: ZA111
Sample:

Date Run: 08-06-2016 (Time Run: 17:31:05)
Instrument: JEOL JMSGCmateII
Inlet: Direct Probe

Run By: Vincent Jactel
Ionization mode: EI+

388,1569 : 3 % **Scan: 300** **TIC: 2972732**

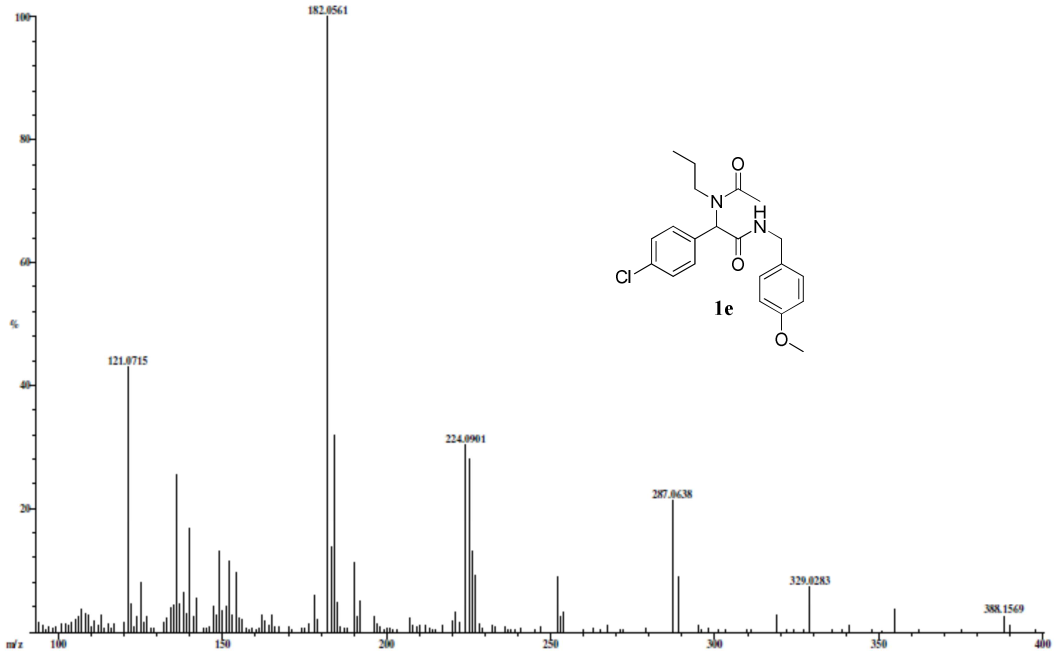

Laboratoire de Synthèse Organique

23/05/2016 11:22:42

File: ZA134
Sample:

Date Run: 05-20-2016 (Time Run: 16:54:25)
Instrument: JEOL JMSGCmateII
Inlet: Direct Probe

Run By: Vincent Jactel
Ionization mode: EI+

Scan: 72 **TIC: 13918908**

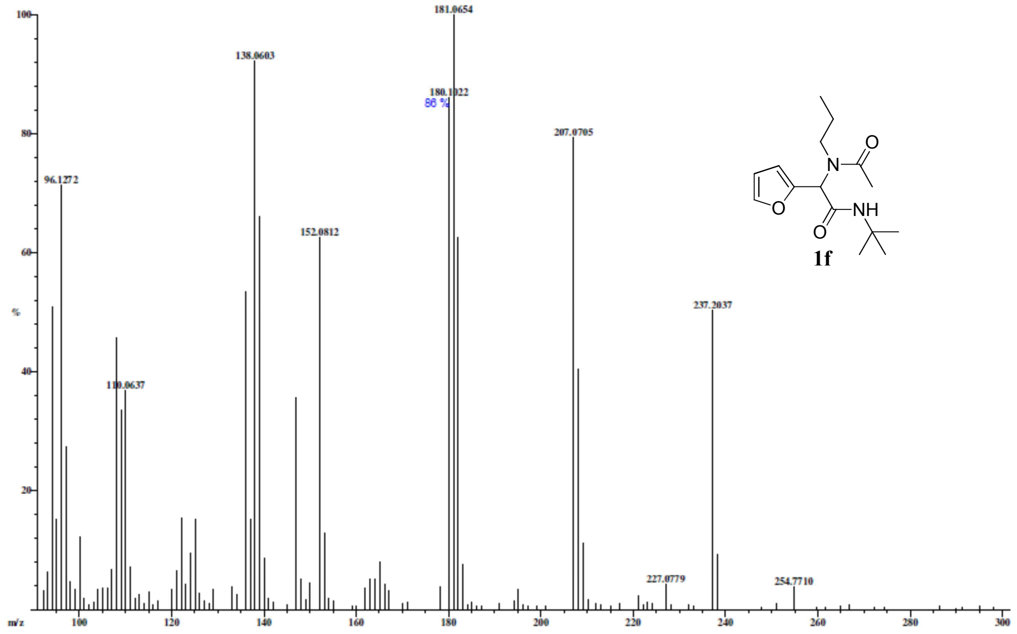

Laboratoire de Synthèse Organique

08/06/2016 16:58:49

File: ZA139
Sample:

Date Run: 08-06-2016 (Time Run: 16:49:14)
Instrument: JEOL JMSGCmateII

Inlet: Direct Probe

Run By: Vincent Jactel
Ionization mode: EI+

291,1954 : 3 % **Scan: 181** **TIC: 15470756**

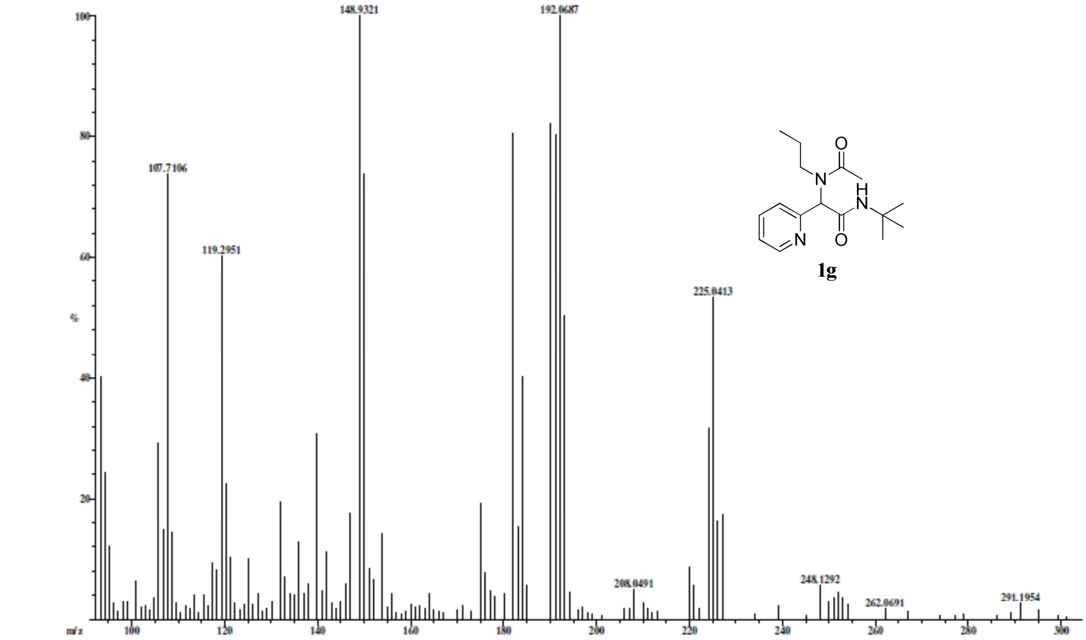

Laboratoire de Synthèse Organique

08/06/2016 16:45:29

File: ZA132 2
Sample: ZA132

Date Run: 08-06-2016 (Time Run: 16:40:25)
Instrument: JEOL JMSGCmateII

Inlet: Direct Probe

Run By: Vincent Jactel
Ionization mode: EI+

324,1614 : 2 % **Scan: 112** **TIC: 11203096**

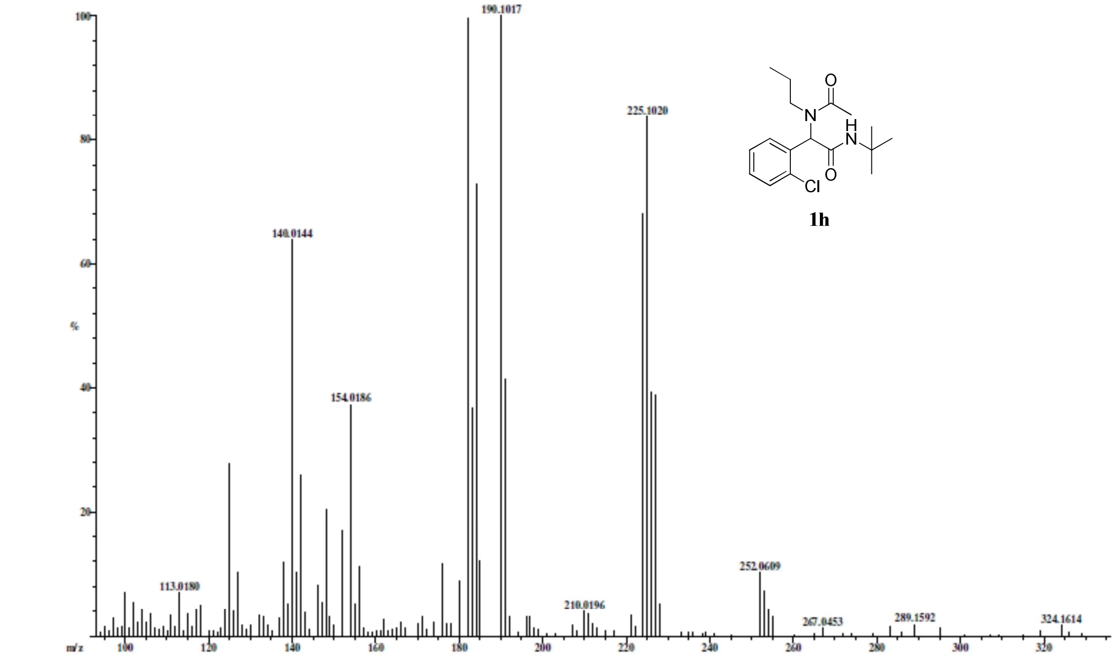

Laboratoire de Synthèse Organique, Palaiseau, France 22/12/2017 15:37:58

File: ZA652 Date Run: 12-22-2017 (Time Run: 15:22:09)
Sample: Instrument: JEOL JMSGCmateII Ionization mode: EI+ Run By: Vincent Jactel

C12H16FNO 209,1213 36% **Scan: 445** **TIC: 643104**

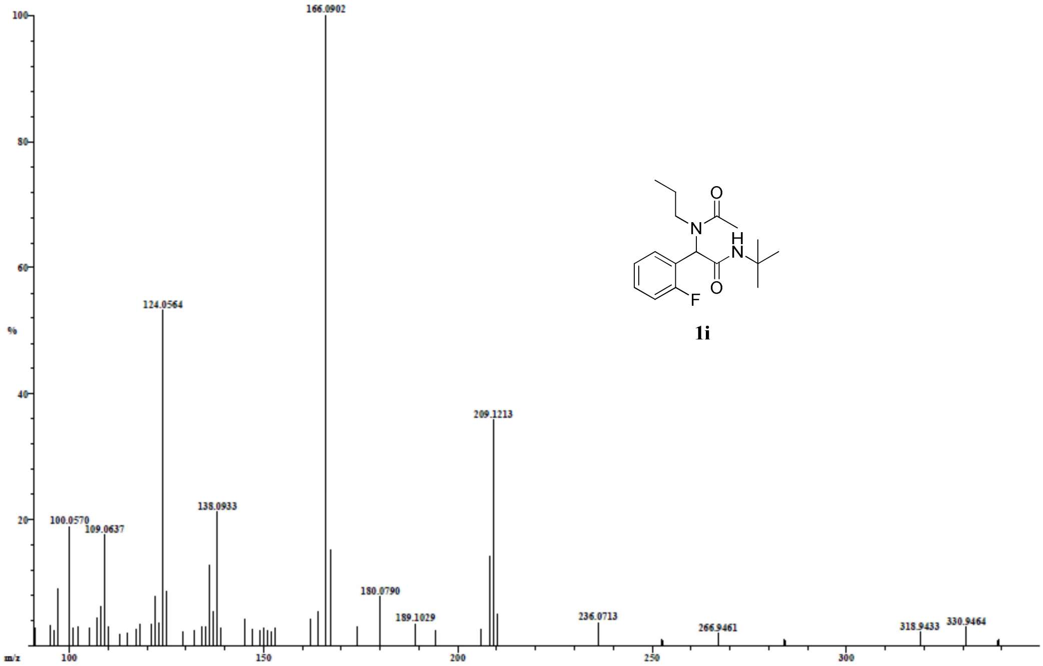

Laboratoire de Synthèse Organique, Palaiseau, France 15/12/2016 09:49:21

File: ZA320 Date Run: 01-12-2016 (Time Run: 15:17:50)
Sample: Instrument: JEOL JMSGCmateII Ionization mode: EI+ Run By: Vincent Jactel

C10H20NO 170,1550 uma 100 % **Scan: 13** **TIC: 5491232**

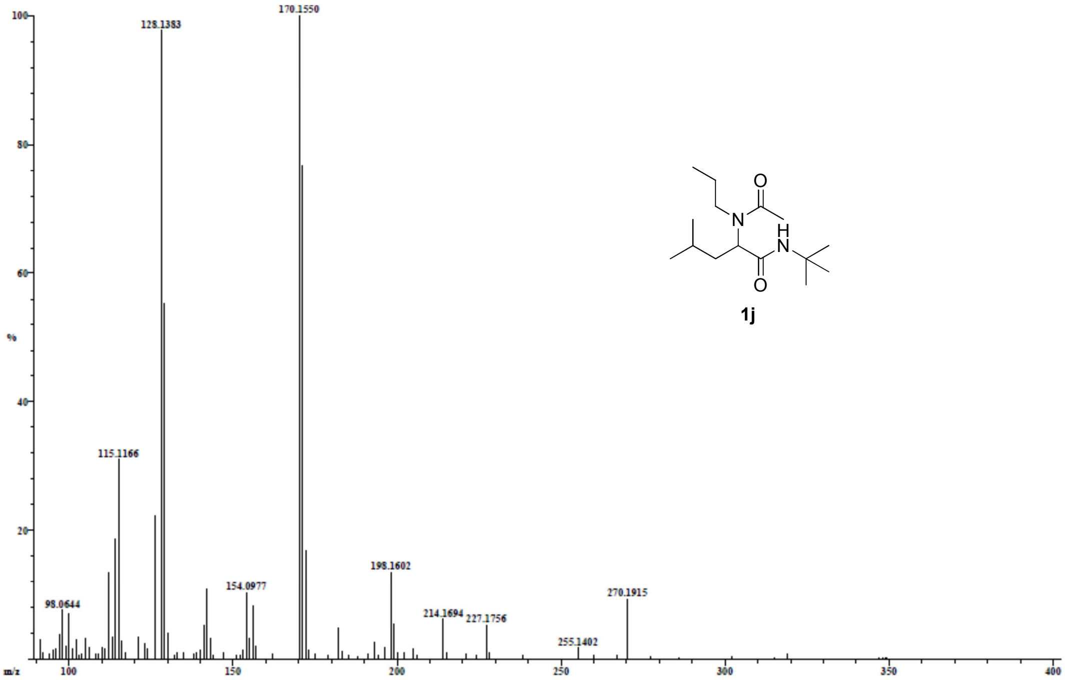

Laboratoire de Synthèse Organique

22/06/2016 09:44:13

File: ZA152
Sample:

Date Run: 06-20-2016 (Time Run: 12:50:30)
Instrument: JEOL JMSGCmateII

Inlet: Direct Probe

Run By: Vincent Jactel
Ionization mode: EI+

303,1024 : 8 % **Scan: 331** **TIC: 7102522**

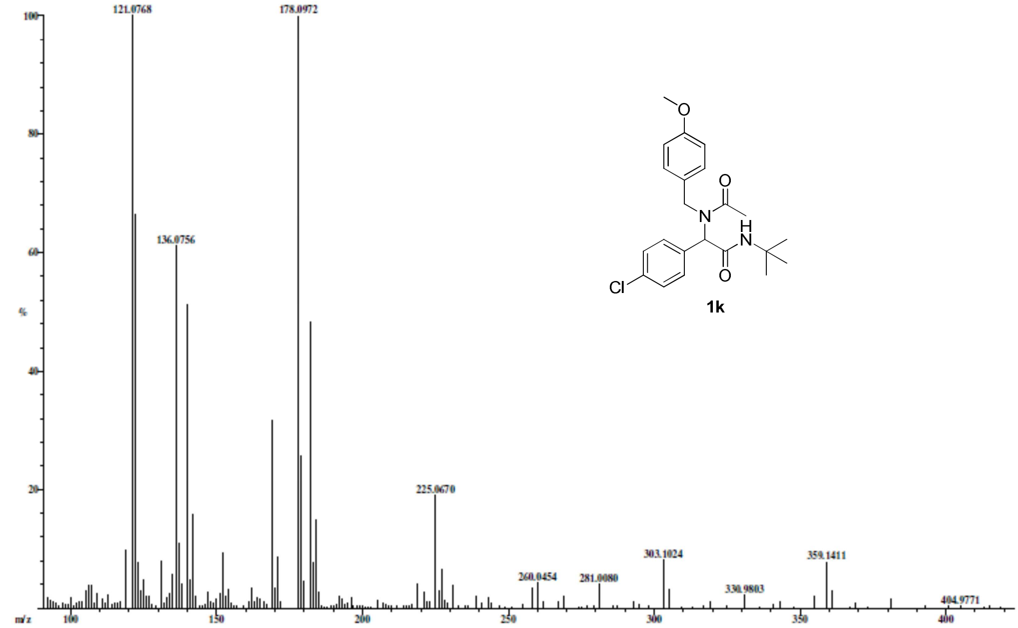

1k

Laboratoire de Synthèse Organique

21/06/2016 15:55:04

File: ZA151
Sample:

Date Run: 06-20-2016 (Time Run: 17:01:13)
Instrument: JEOL JMSGCmate II

Inlet: Direct Probe

Run By: Vincent Jactel
Ionization mode: EI+

388,1543 : 0,6 % **Scan: 373** **TIC: 3930702**

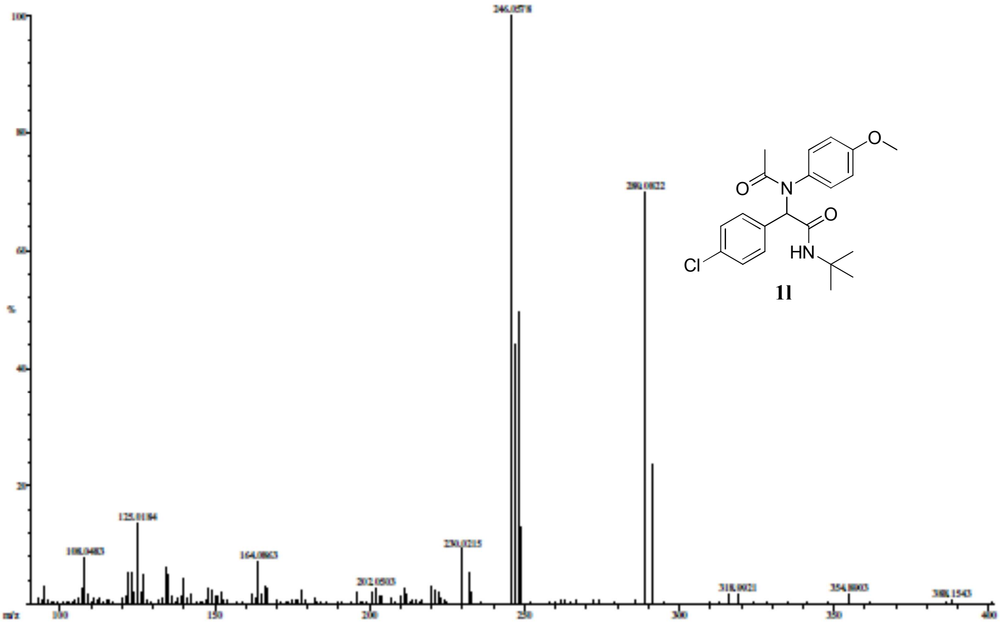

1l

Laboratoire de Synthèse Organique

08/06/2016 18:03:45

File: ZA148
Sample:

Date Run: 08-06-2016 (Time Run: 17:50:37)
Instrument: JEOL JMSGCmateII
Inlet: Direct Probe

Run By: Vincent Jactel
Ionization mode: EI+

322,1456 : 1 % **Scan: 261** **TIC: 8209978**

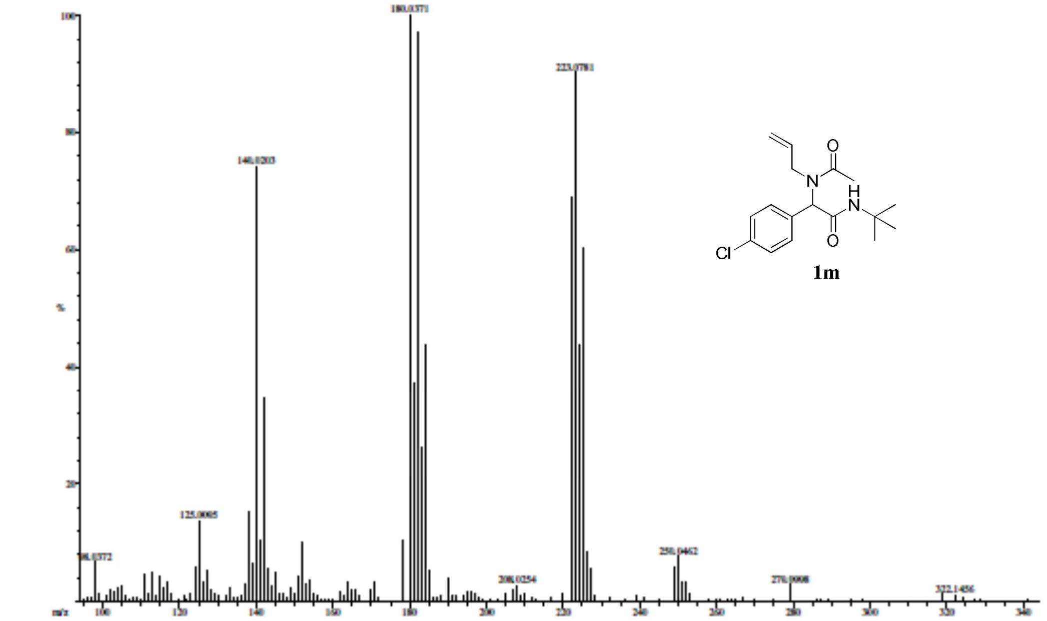

Laboratoire de Synthèse Organique, Palaiseau, France

28/09/2016 13:00:32

File: ZA190
Sample:

Date Run: 09-28-2016 (Time Run: 12:53:11)
Instrument: JEOL JMSGCmateII
Inlet: Direct Probe

Run By: Vincent Jactel
Ionization mode: EI+

C19H25ClN2O2 348,1614 7 % **Scan: 242** **TIC: 9268186**

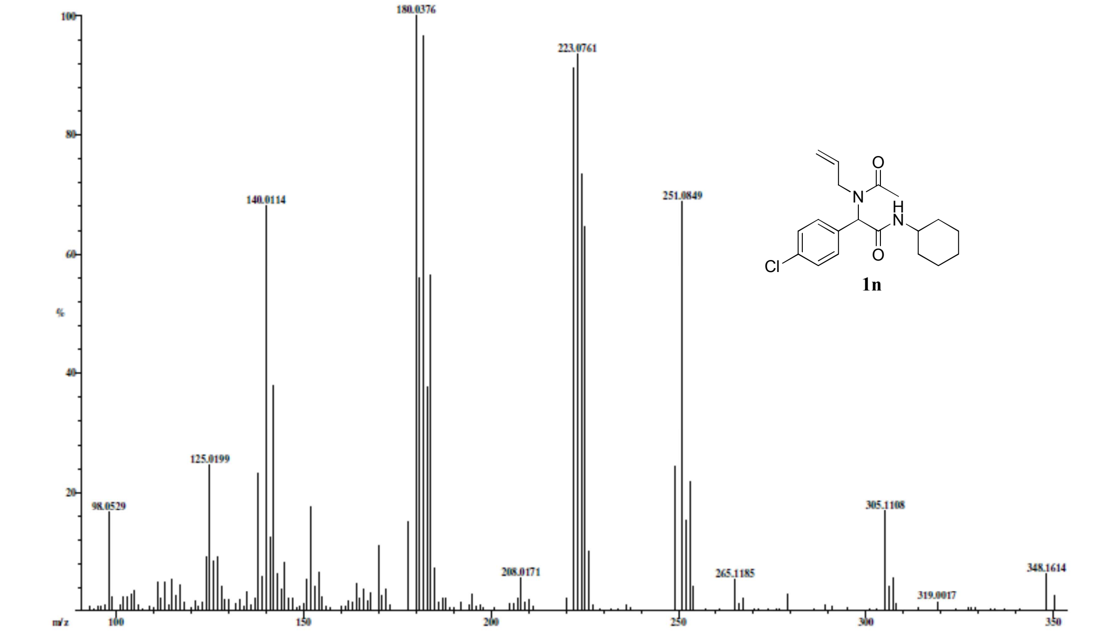

Laboratoire de Synthèse Organique, Palaiseau, France

08/08/2016 11:30:45

File: ZA204
Sample:

Date Run: 08-08-2016 (Time Run: 11:24:14)
Instrument: JEOL JMSGCmate II
Inlet: Direct Probe

Run By: Vincent Jactel
Ionization mode: EI+

C16H23N3O2 289,1783 : 3 % **Scan: 227** **TIC: 11772832**

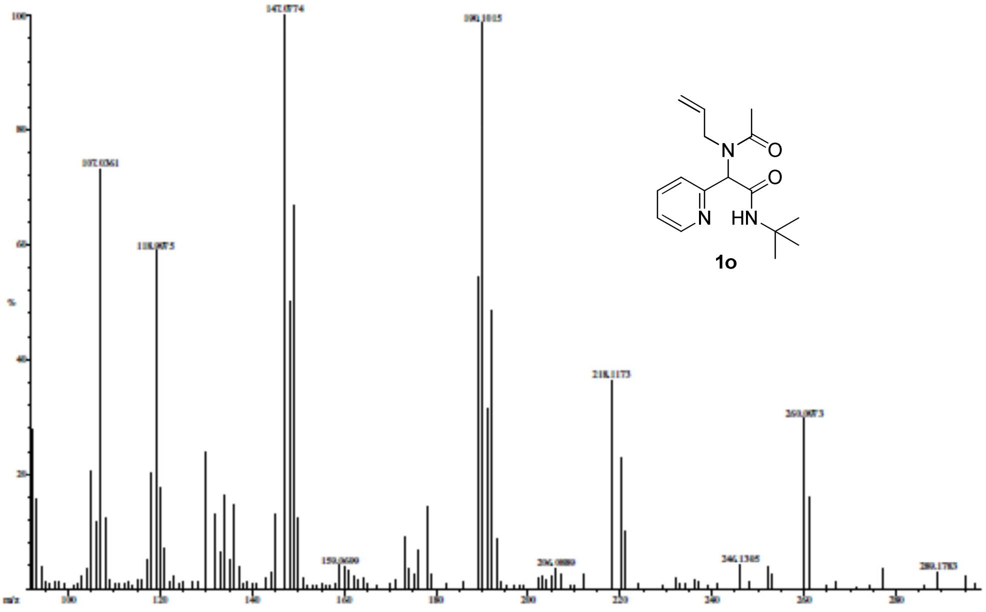

Laboratoire de Synthèse Organique, Palaiseau, France

26/09/2016 18:00:48

File: ZA174
Sample:

Date Run: 09-26-2016 (Time Run: 17:21:14)
Instrument: JEOL JMSGCmate II
Inlet: Direct Probe

Run By: Vincent Jactel
Ionization mode: EI+

C23H27ClN2O3 : 414,1701 : 0,4 % L'absence du pic du second isotope du chlore est due à un dysfonctionnement du spectromètre. **TIC: 3134536**

Scan: 246

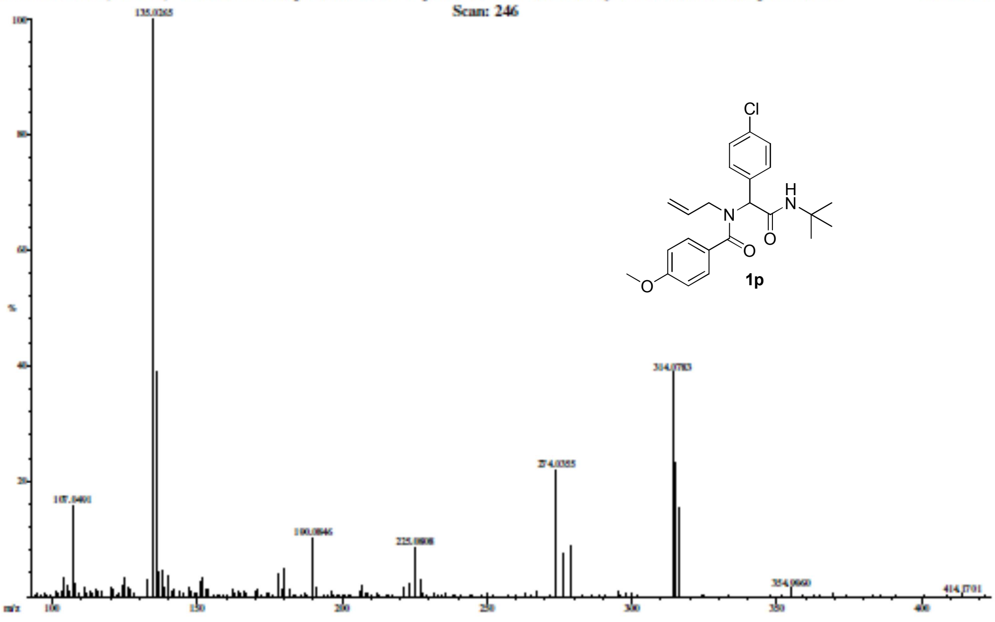

Laboratoire de Synthèse Organique, Palaiseau, France

28/09/2016 13:10:16

File: ZA203 Date Run: 09-28-2016 (Time Run: 13:03:49) Run By: Vincent Jactel
Sample: Instrument: JEOL JMSGCmateII Inlet: Direct Probe Ionization mode: EI+

C25H31ClN2O3 442,2023 9% L'absence du pic des molécules porteuses de l'isotope 37Cl est due à un dysfonctionnement du spectromètre.

Scan: 180 **TIC: 3978082**

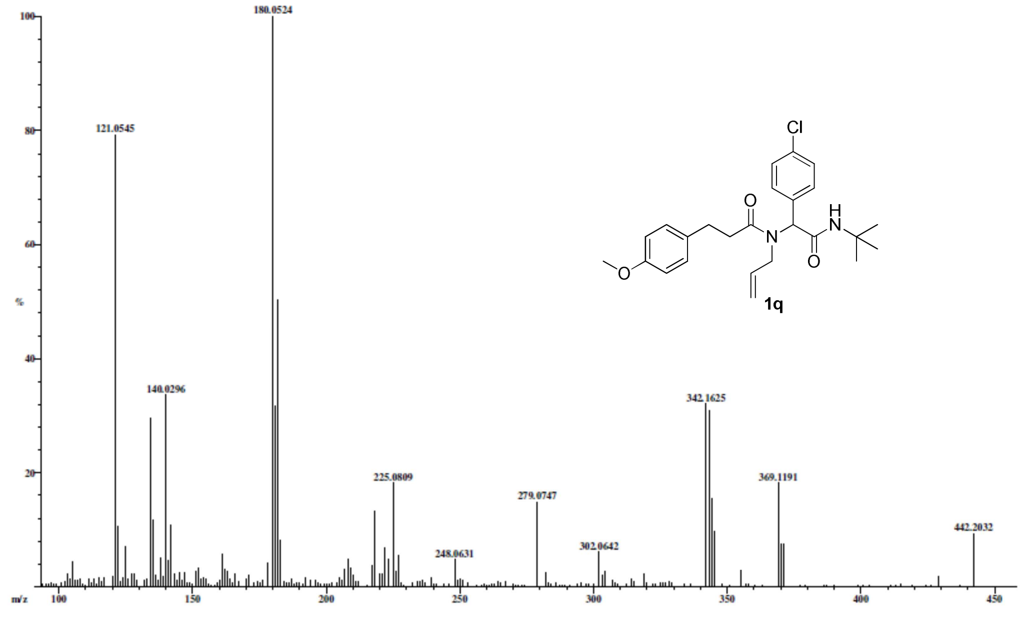

Laboratoire de Synthèse Organique, Palaiseau, France 13/12/2016 11:30:53

File: ZA321 Date Run: 12-13-2016 (Time Run: 08:37:56)
Sample: Instrument: JEOL JMSGCmateII Ionization mode: EI+ Run By: Vincent Jactel

C19H19ClNO 312.1156 uma 25% L'absence du pic des molécules avec l'isotope 37Cl est due à un dysfonctionnement du spectromètre. TIC: 2183984

Scan: 178

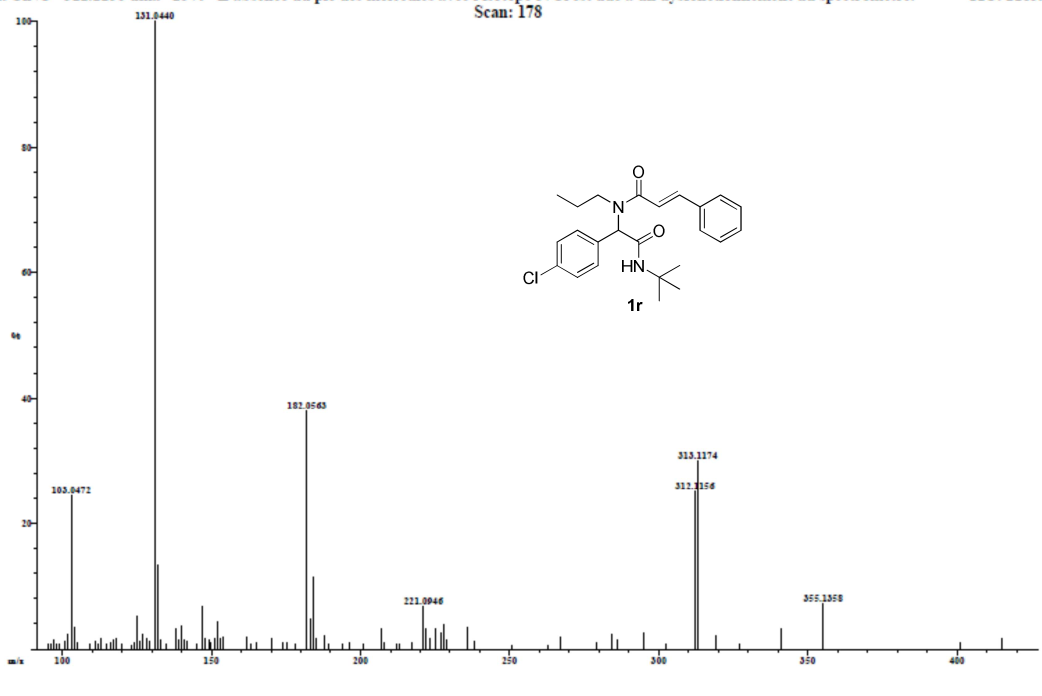

Laboratoire de Synthèse Organique

08/06/2016 14:46:02

File: ZA149
Sample:

Date Run: 08-06-2016 (Time Run: 14:39:54)
Instrument: JEOL JMSGCmateII
Inlet: Direct Probe

Run By: Vincent Jactel
Ionization mode: EI+

320,1279 : 3 % **Scan: 213** **TIC: 14622804**

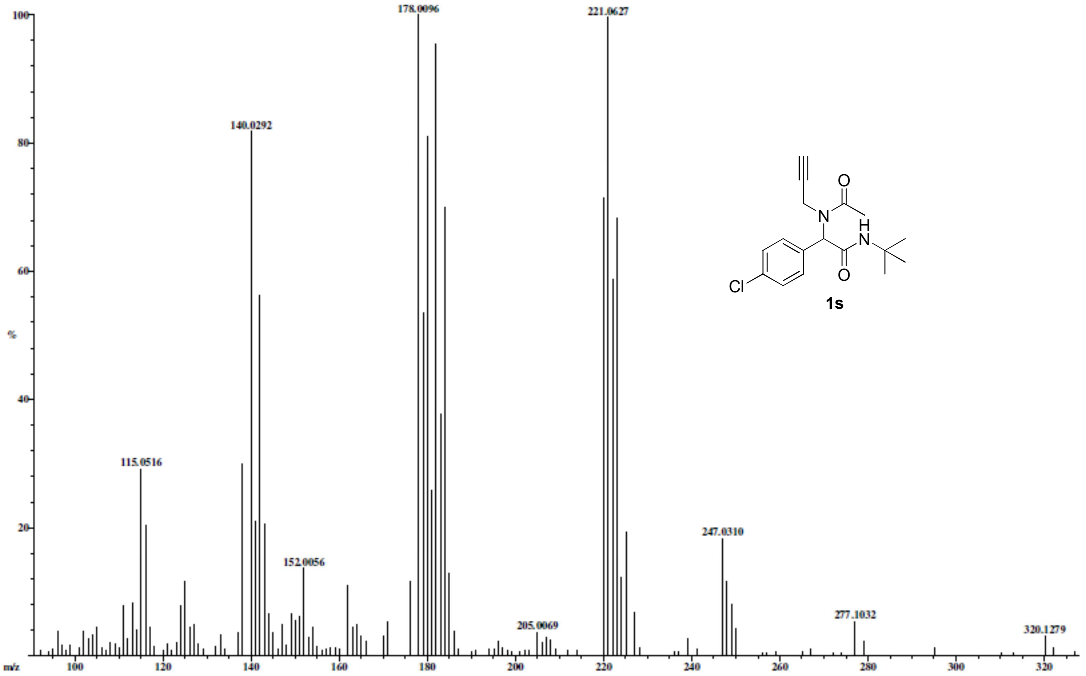

Laboratoire de Synthèse Organique, Palaiseau, France

04/11/2016 17:20:33

File: ZA114
Sample:

Date Run: 04-11-2016 (Time Run: 17:10:50)
Instrument: JEOL JMSGCmateII

Run By: Vincent Jactel
Ionization mode: EI+

C20H29ClN2O2 364,1911 amu 1 % **Scan: 266** **TIC: 5779872**

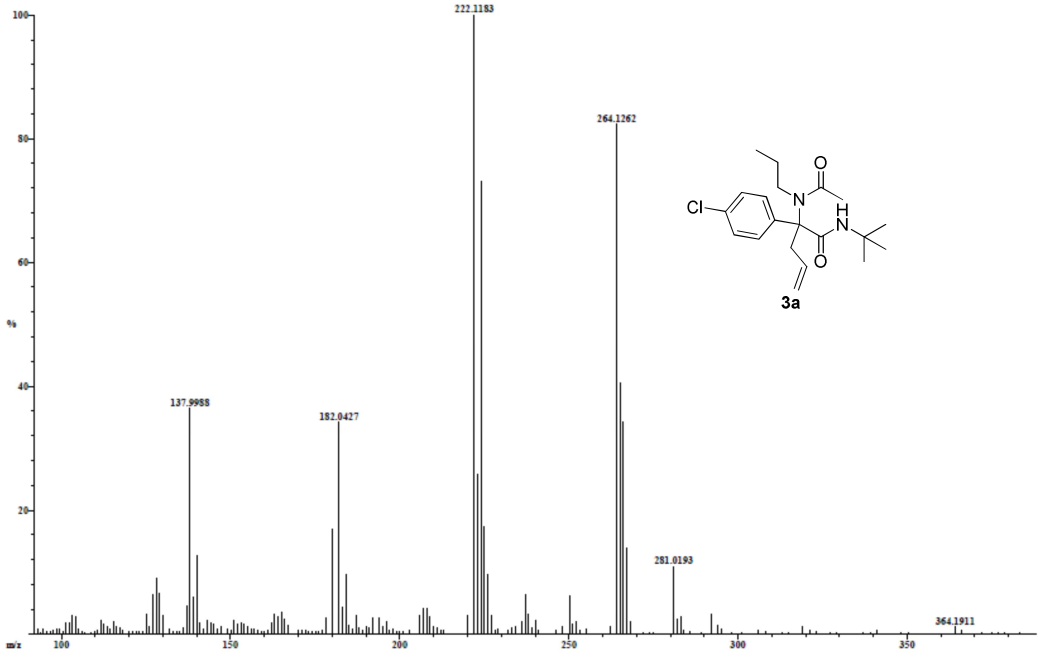

Laboratoire de Synthèse Organique, Palaiseau, France 04/11/2016 17:26:56

File: ZA205 Date Run: 04-11-2016 (Time Run: 17:19:18) Run By: Vincent Jactel
Sample: Instrument: JEOL JMSGCmateII Ionization mode: EI+

C20H29N3O4 375,2173 amu 4% **Scan: 162** **TIC: 11192314**

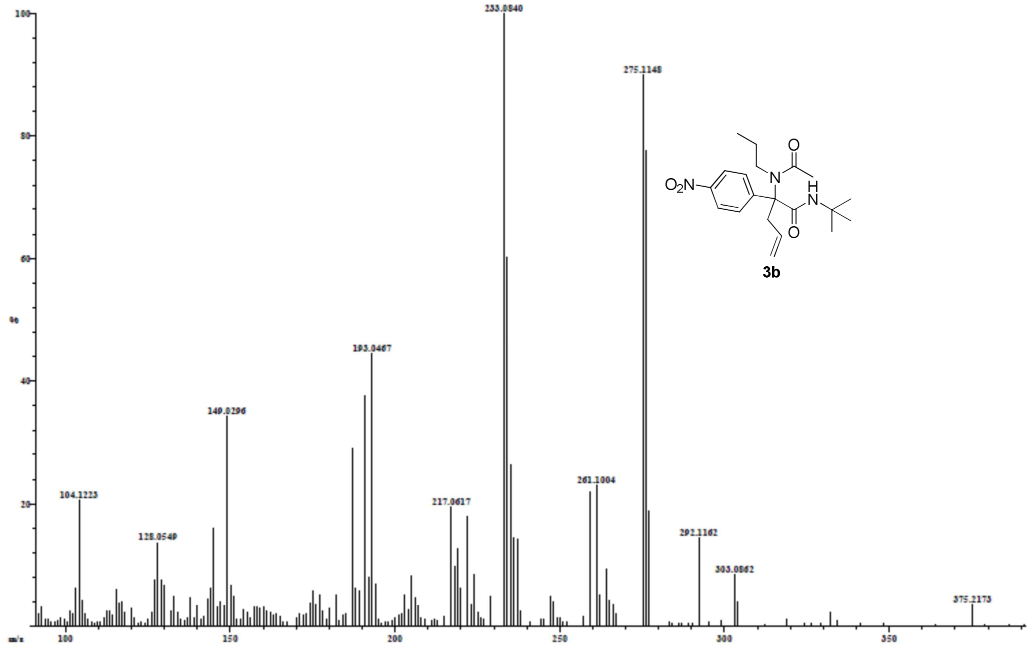

Laboratoire de Synthèse Organique, Palaiseau, France

08/08/2016 11:21:34

File: ZA133 Date Run: 08-08-2016 (Time Run: 11:16:24) Run By: Vincent Jactel
Sample: Instrument: JEOL JMSGCmateII Inlet: Direct Probe Ionization mode: EI+

C21H32N2O3 : 360,2420 : 4 % **Scan: 138** **TIC: 12909458**

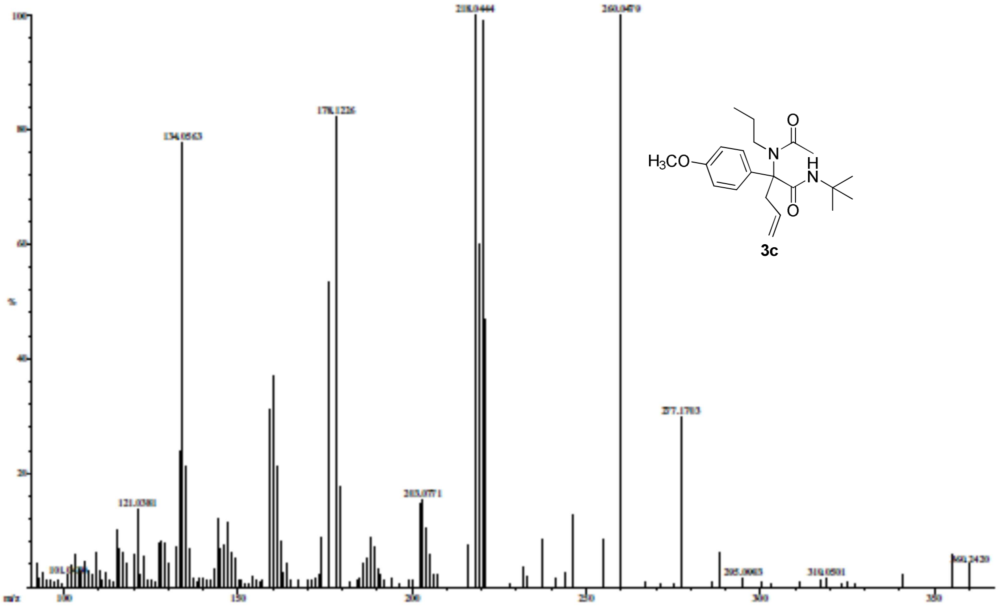

Laboratoire de Synthèse Organique, Palaiseau, France

27/09/2016 08:40:57

File: ZA117 Date Run: 09-27-2016 (Time Run: 08:29:05) Run By: Vincent Jactel
Sample: Instrument: JEOL JMSGCmate II Inlet: Direct Probe Ionization mode: EI+

C15H19ClNO 390,2088 : 1% L'absence du pic moléculaire de l'isotope 37 du chlore est due à un dysfonctionnement du spectromètre. TIC: 3096598

Scan: 116

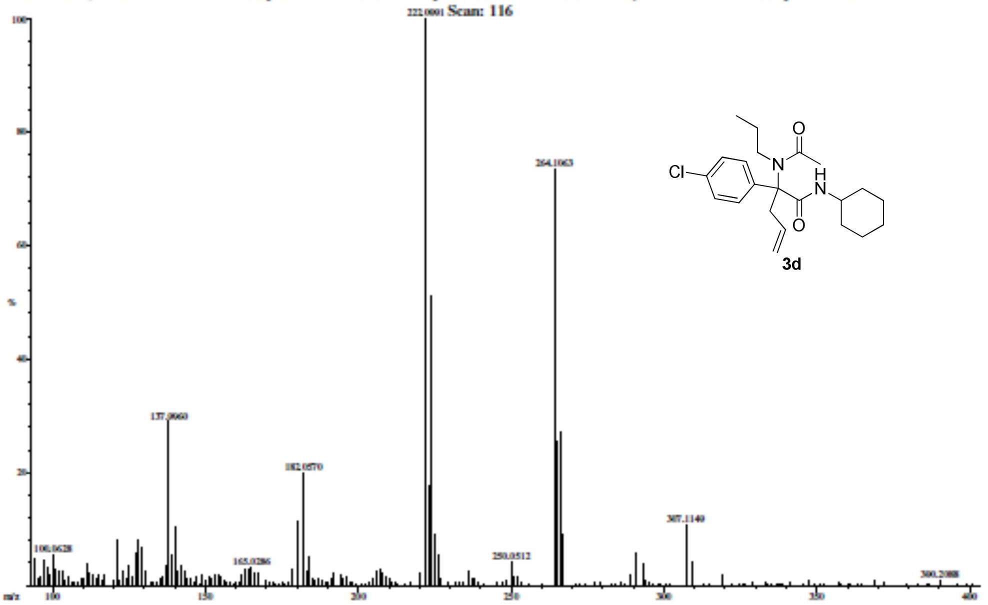

Laboratoire de Synthèse Organique

24/05/2016 10:42:47

File: ZA118 Date Run: 05-24-2016 (Time Run: 09:57:05) Run By: Vincent Jactel
Sample: Instrument: JEOL JMSGCmate II Inlet: Direct Probe Ionization mode: EI+

Scan: 335 TIC: 12972312

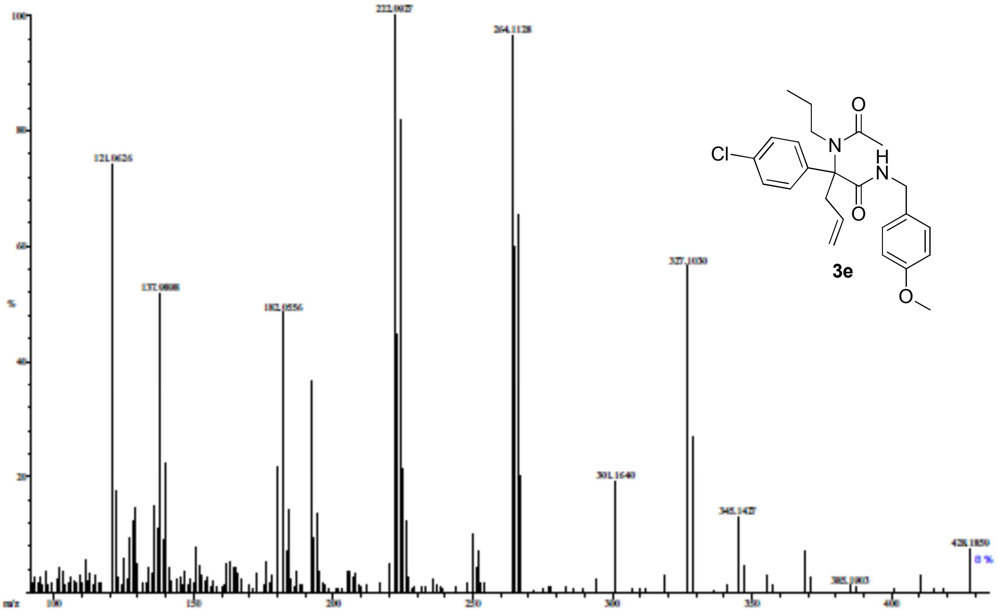

Laboratoire de Synthèse Organique

20/05/2016 17:52:54

File: ZA137 Date Run: 05-20-2016 (Time Run: 17:46:48) Run By: Vincent Jactel
Sample: Instrument: JEOL JMSGCmateII Inlet: Direct Probe Ionization mode: EI+

Scan: 124 **TIC: 11318458**

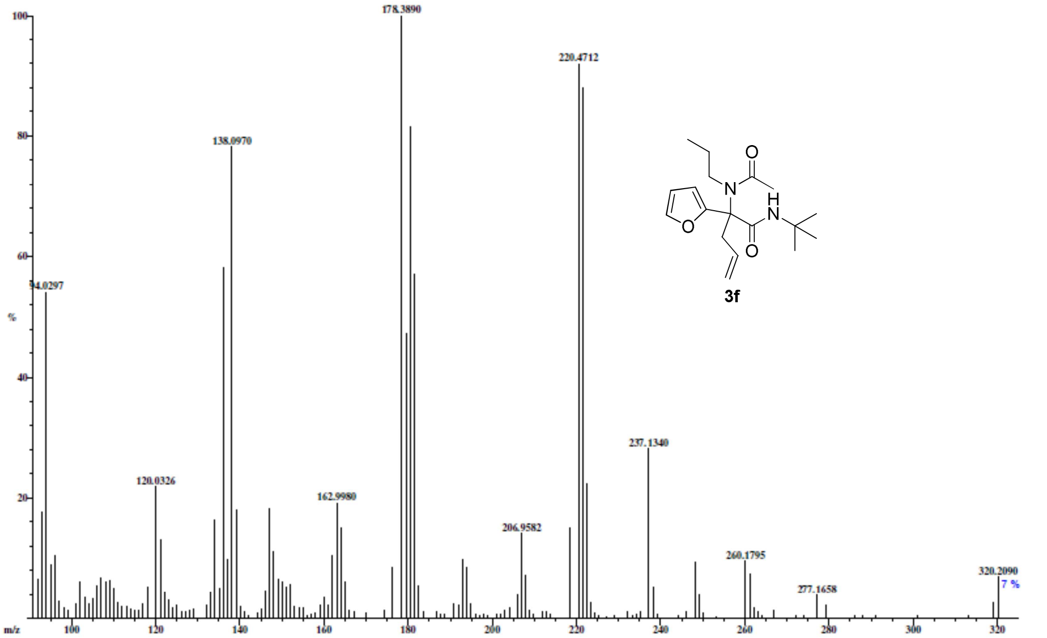

Laboratoire de Synthèse Organique, Palaiseau, France

28/09/2016 16:11:22

File: ZA199 Date Run: 08-08-2016 (Time Run: 09:25:04) Run By: Vincent Jactel
Sample: Instrument: JEOL JMSGCmateII Inlet: Direct Probe Ionization mode: EI+

C14H19N2O 231,1494 85 % **Scan: 149** **TIC: 8652792**

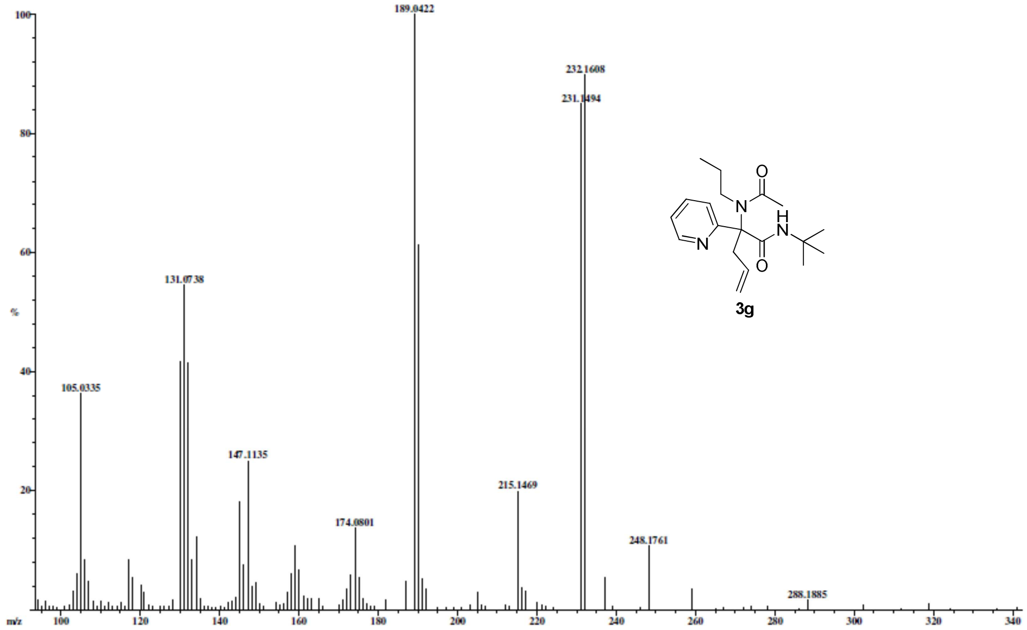

Laboratoire de Synthèse Organique, Palaiseau, France

26/09/2016 18:53:43

File: ZA166 | Date Run: 09-26-2016 (Time Run: 18:43:47) | Run By: Vincent Jactel
Sample: | Instrument: JEOL JMSGCmateII | Inlet: Direct Probe | Ionization mode: EI+

C20H21ClNO2 : 342,1254 15% L'absence du pic du second isotope du chlore est due à un dysfonctionnement du spectromètre. **TIC: 5960590**

Scan: 258

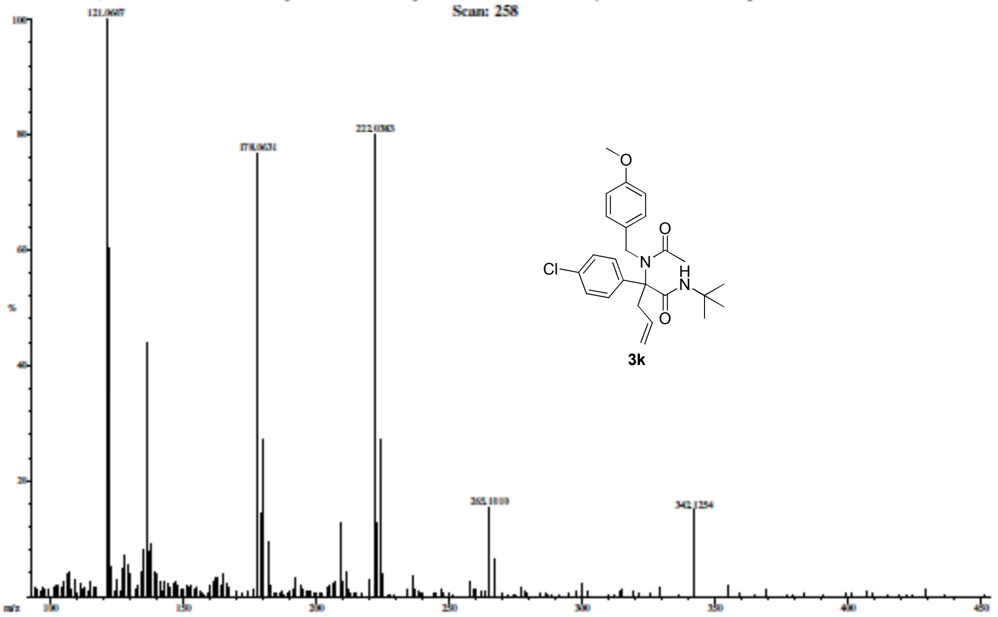

Laboratoire de Synthèse Organique

21/06/2016 15:00:45

File: ZA158 2 | Date Run: 06-21-2016 (Time Run: 14:53:36) | Run By: Vincent Jactel
Sample: ZA158 | Instrument: JEOL JMSGCmateII | Inlet: Direct Probe | Ionization mode: EI+

428,1885 : 3 % **Scan: 178** **TIC: 6477590**

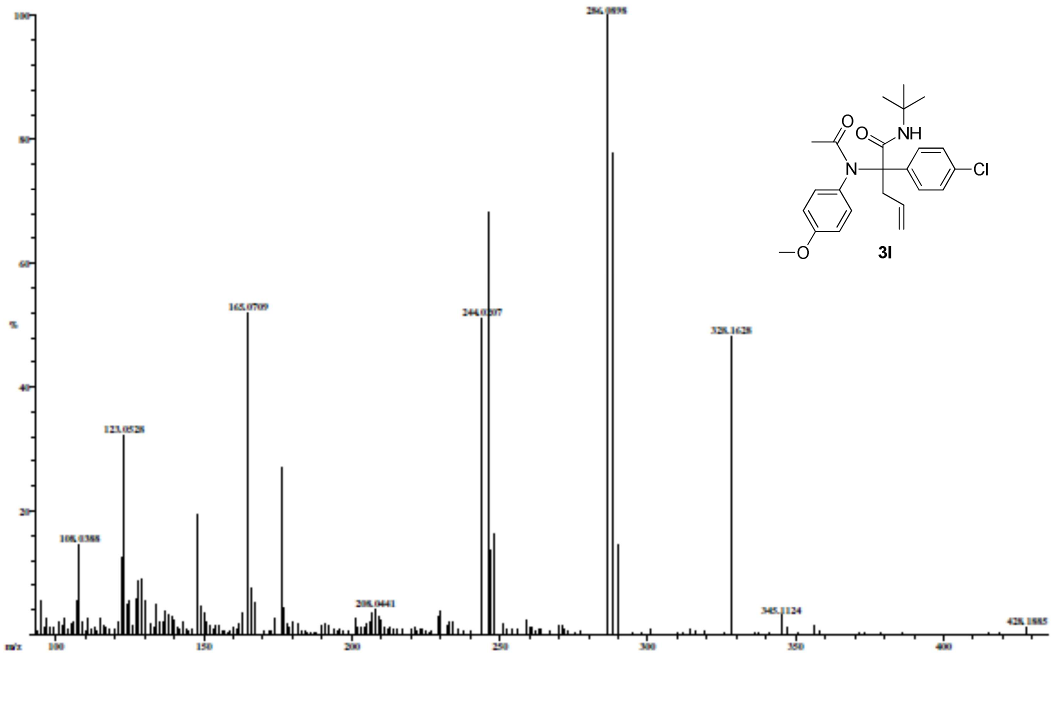

Laboratoire de Synthèse Organique

21/06/2016 15:32:23

File: ZA150
Sample:

Date Run: 06-20-2016 (Time Run: 15:12:10)
Instrument: JEOL JMSGCmateII
Inlet: Direct Probe

Run By: Vincent Jactel
Ionization mode: EI+

362,1749 : 3 % **Scan: 262** **TIC: 15960044**

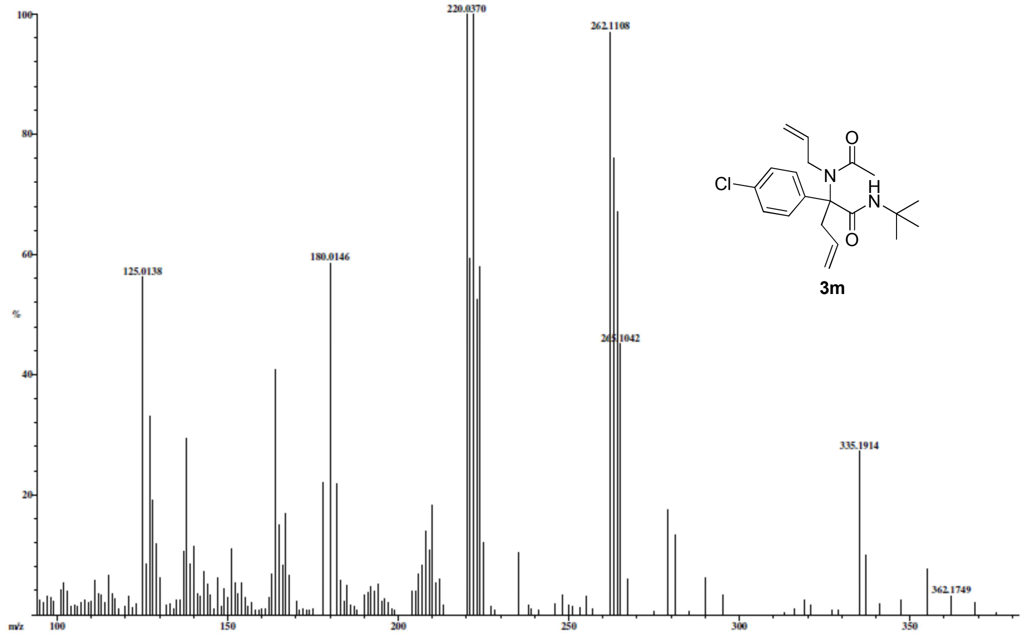

Laboratoire de Synthèse Organique, Palaiseau, France

29/09/2016 09:05:43

File: ZA198 bis
Sample: ZA198

Date Run: 09-29-2016 (Time Run: 08:55:45)
Instrument: JEOL JMSGCmateII
Inlet: Direct Probe

Run By: Vincent Jactel
Ionization mode: EI+

C22H29ClN2O2 388,1913 4 % **Scan: 364** **TIC: 5252558**

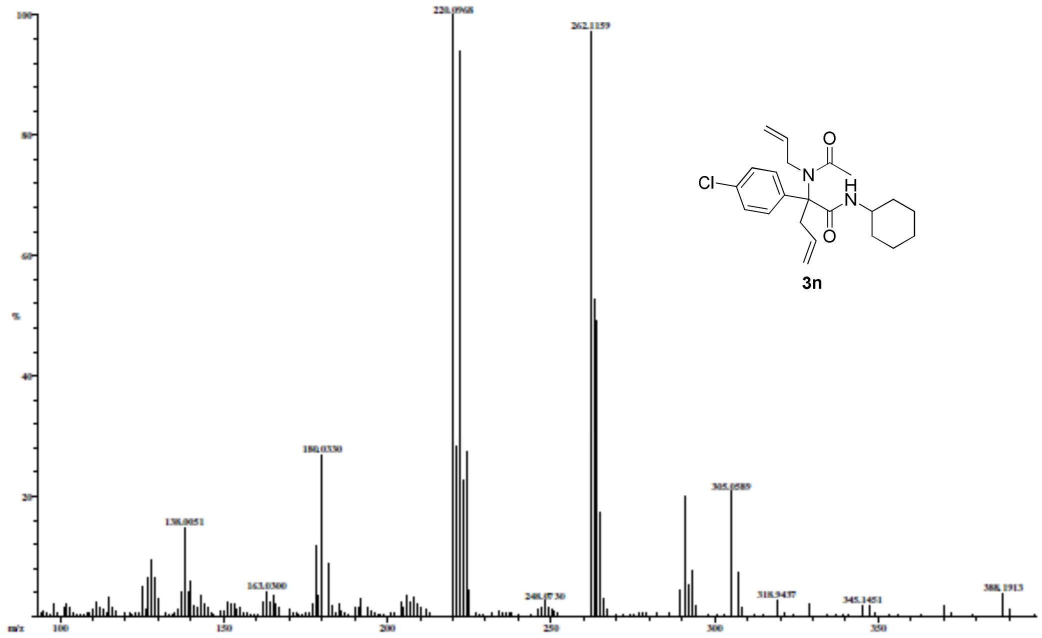

Laboratoire de Synthèse Organique, Palaiseau, France 04/11/2016 14:50:01

File: ZA210 Date Run: 04-11-2016 (Time Run: 14:46:59) Run By: Vincent Jactel
Sample: Instrument: JEOL JMSGCmateII Ionization mode: EI+

C19H27N3O2 329,2098 amu 7 % **Scan: 82** **TIC: 28506572**

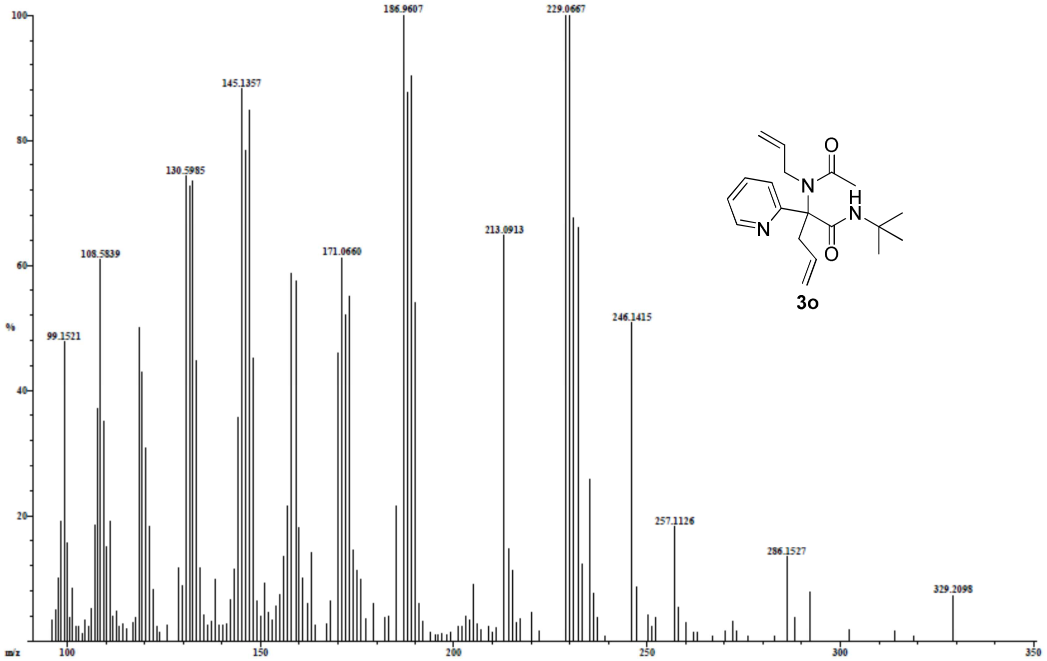

3o

Laboratoire de Synthèse Organique, Palaiseau, France

26/09/2016 17:51:31

File: ZA176 Date Run: 09-26-2016 (Time Run: 17:34:56) Run By: Vincent Jactel
Sample: Instrument: JEOL JMSGCmateII Inlet: Direct Probe Ionization mode: EI+

C21H21ClNO2 : 354,1262 29 % L'abscence du pic du second isotope du chlore est due à un dysfonctionnement du spectromètre. **TIC: 2165892**
Scan: 277

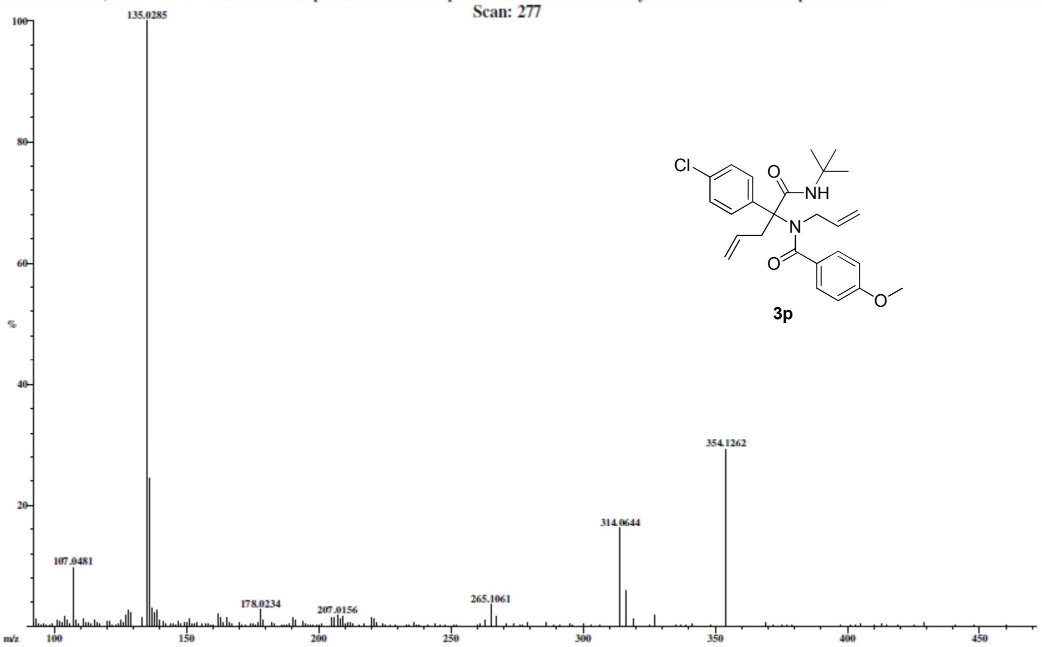

3p

Laboratoire de Synthèse Organique, Palaiseau, France 04/11/2016 14:44:07

File: ZA209 Date Run: 04-11-2016 (Time Run: 14:23:15) Run By: Vincent Jactel
Sample: Instrument: JEOL JMSGCmateII Ionization mode: EI+

C23H25NO2Cl 382,1579 amu 13 % **Scan: 570** **TIC: 858726**

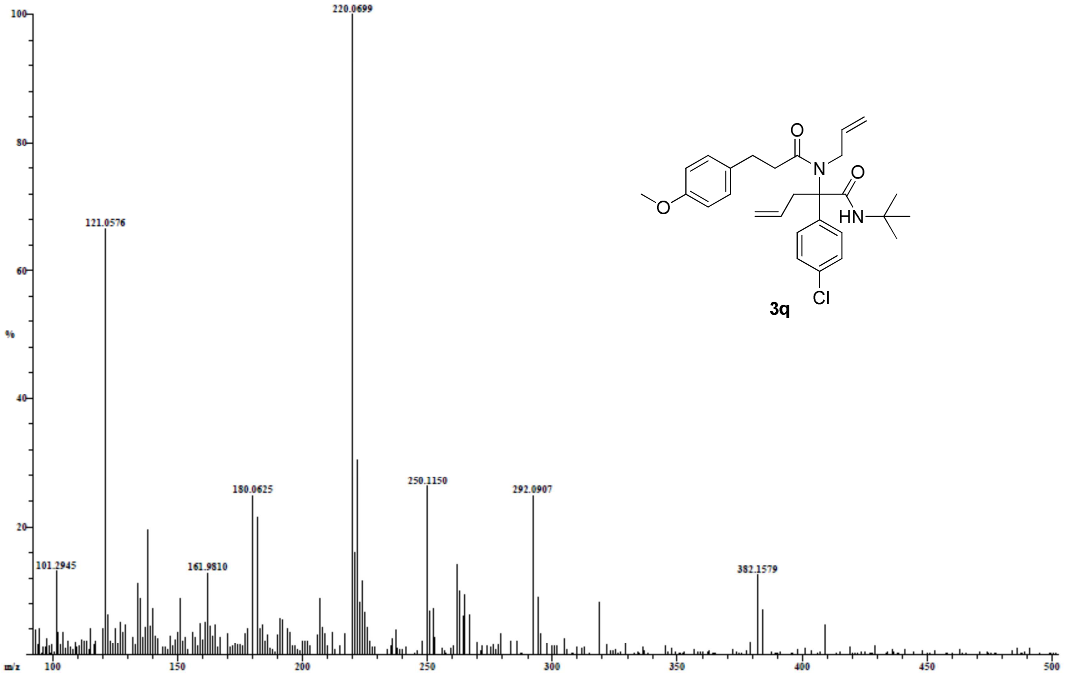

Laboratoire de Synthèse Organique, Palaiseau, France 02/12/2016 12:19:14

File: ZA328 Date Run: 02-12-2016 (Time Run: 09:37:13)
Sample: Instrument: JEOL JMSGCmateII Ionization mode: EI+ Run By: Vincent Jactel

C27H33ClN2O2 452.2228 amu 1% L'absence du pic des molécules avec l'isotope 37Cl est due à un dysfonctionnement du spectromètre. **TIC: 5154592**
Scan: 232

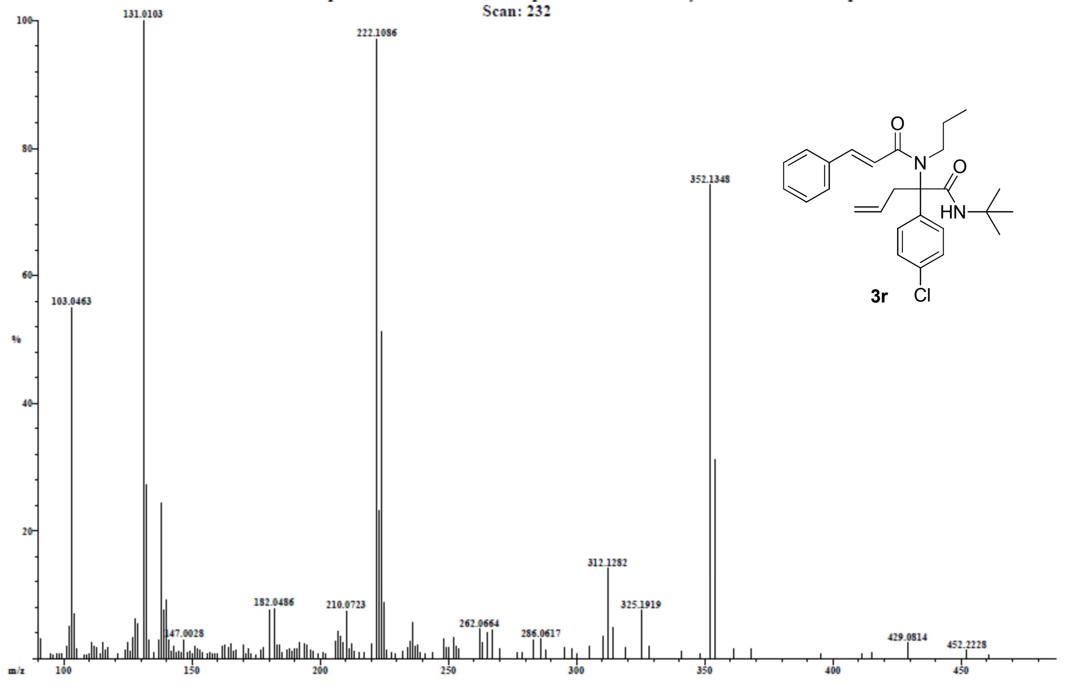

Laboratoire de Synthèse Organique

21/06/2016 16:32:14

File: ZA157 2
Sample: ZA157

Date Run: 06-21-2016 (Time Run: 16:19:25)
Instrument: JEOL JMSGCmateII
Inlet: Direct Probe
Run By: Vincent Jactel
Ionization mode: EI+

Scan: 197 TIC: 5099216

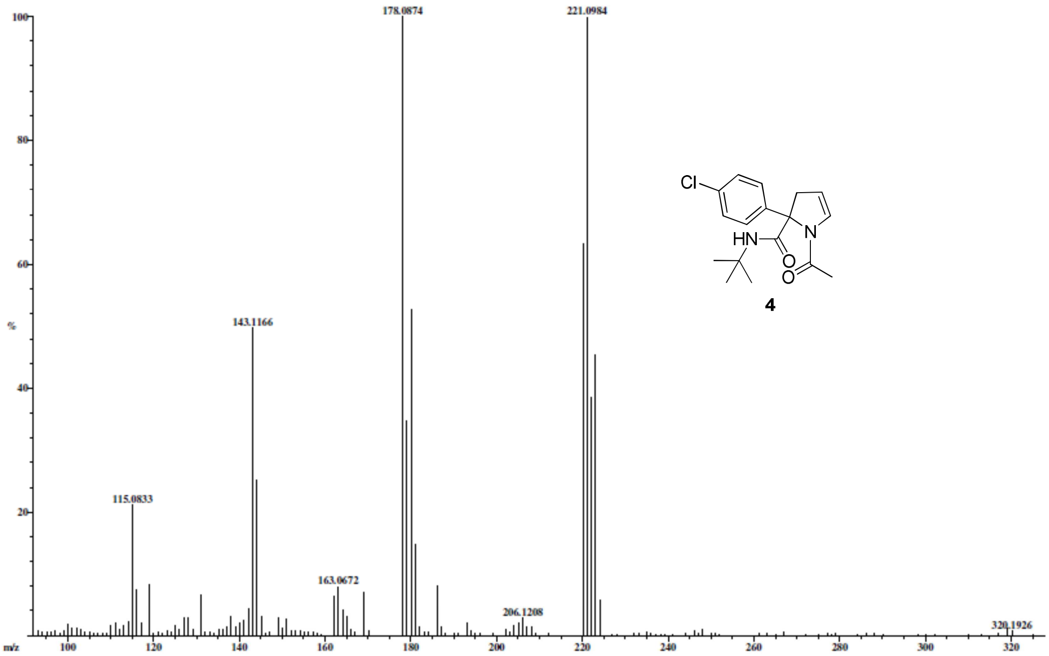

Laboratoire de Synthèse Organique, Palaiseau, France

20/02/2018 08:52:30

File: ZA681
Sample:

Date Run: 02-20-2018 (Time Run: 08:40:17)
Instrument: JEOL JMSGCmateII
Ionization mode: EI+
Run By: Vincent Jactel

C15H15ClNO 260.0844 15% Scan: 384 TIC: 2095024

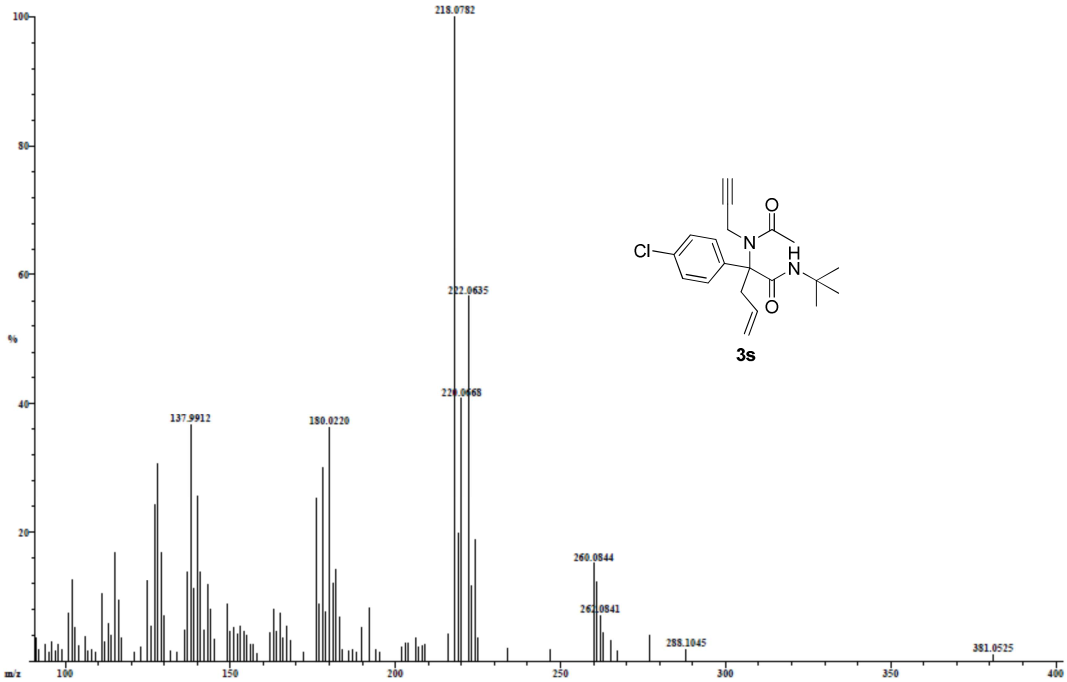

Laboratoire de Synthèse Organique, Palaiseau, France 22/12/2017 08:39:41

File: ZA660 Date Run: 12-22-2017 (Time Run: 08:22:12)
Sample: Instrument: JEOL JMSGCmateII Ionization mode: EI+ Run By: Vincent Jactel

C15H99FNO 248,1507 33% **Scan: 312** **TIC: 381248**

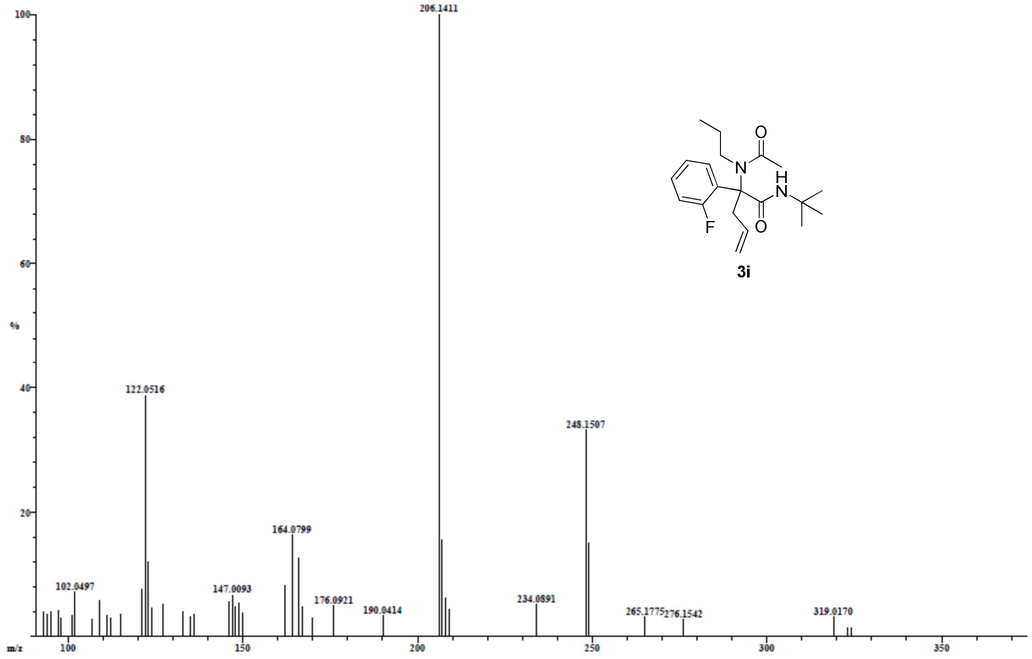

Laboratoire de Synthèse Organique, Palaiseau, France

29/09/2016 08:41:36

File: ZA182 Date Run: 09-29-2016 (Time Run: 08:34:31) Run By: Vincent Jactel
Sample: Instrument: JEOL JMSGCmateII Inlet: Direct Probe Ionization mode: EI+

C26H33ClN2O2 440,2229 5% **Scan: 253** **TIC: 20280400**

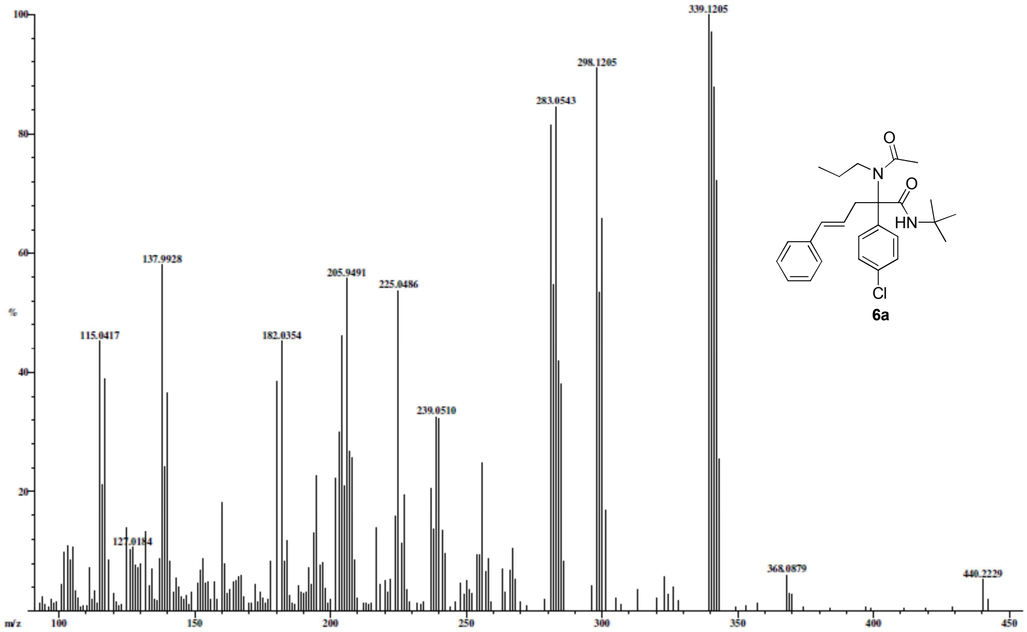

Laboratoire de Synthèse Organique, Palaiseau, France 04/11/2016 14:05:16

File: ZA216 Date Run: 04-11-2016 (Time Run: 13:59:49) Run By: Vincent Jactel
Sample: Instrument: JEOL JMSGCmateII Ionization mode: EI+

C17H23ClNO 292,1475 amu 100 % **Scan: 75** **TIC: 16743530**

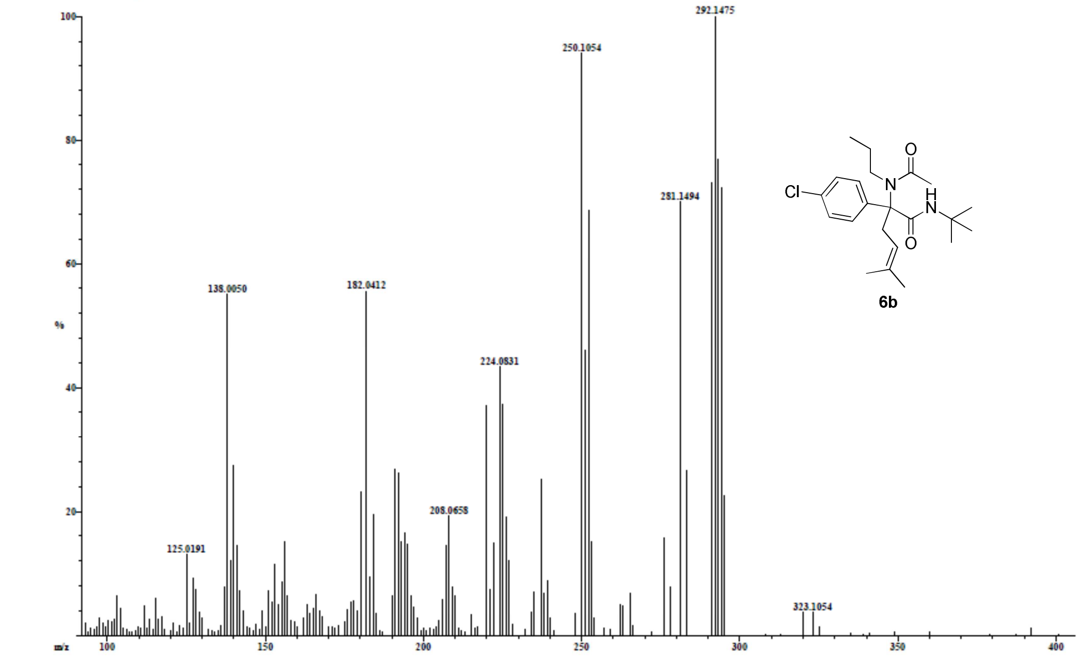

Laboratoire de Synthèse Organique, Palaiseau, France 04/11/2016 16:36:47

File: ZA217 Date Run: 04-11-2016 (Time Run: 16:24:06) Run By: Vincent Jactel
Sample: Instrument: JEOL JMSGCmateII Ionization mode: EI+

C18H23ClNO 304,1472 amu 100 % **Scan: 153** **TIC: 2011596**

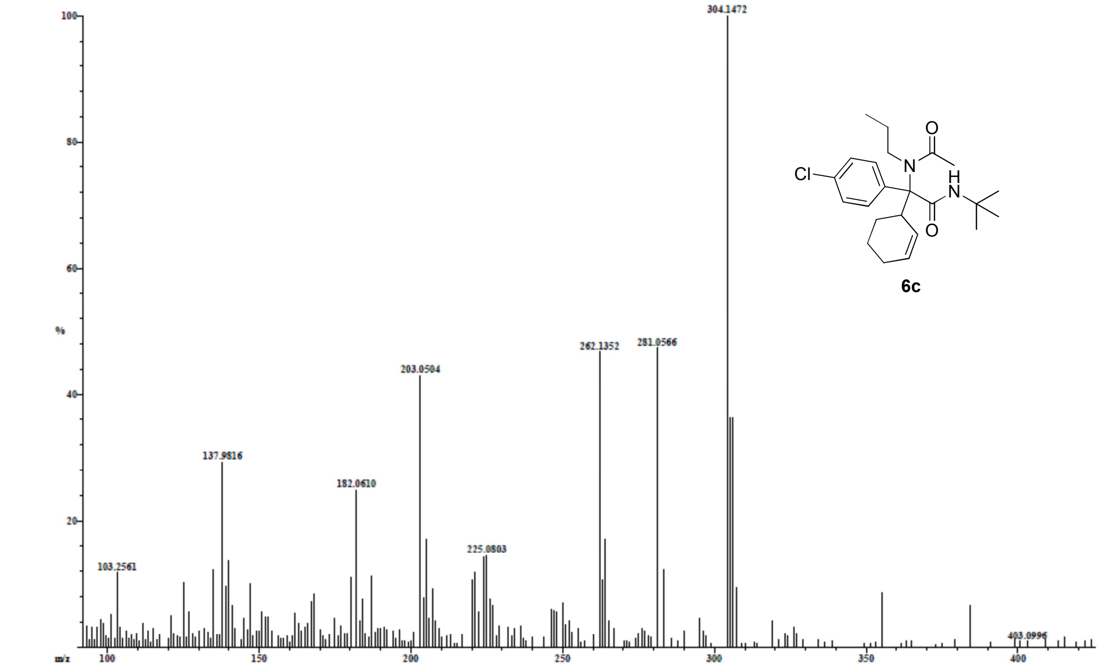

Laboratoire de Synthèse Organique, Palaiseau, France 02/12/2016 12:17:23

File: ZA326 Date Run: 02-12-2016 (Time Run: 09:13:22)

Sample: Instrument: JEOL JMSGCmateII Ionization mode: EI+ Run By: Vincent Jactel

C14H19ClNO 252.1153 amu 95% **Scan: 89** **TIC: 14148224**

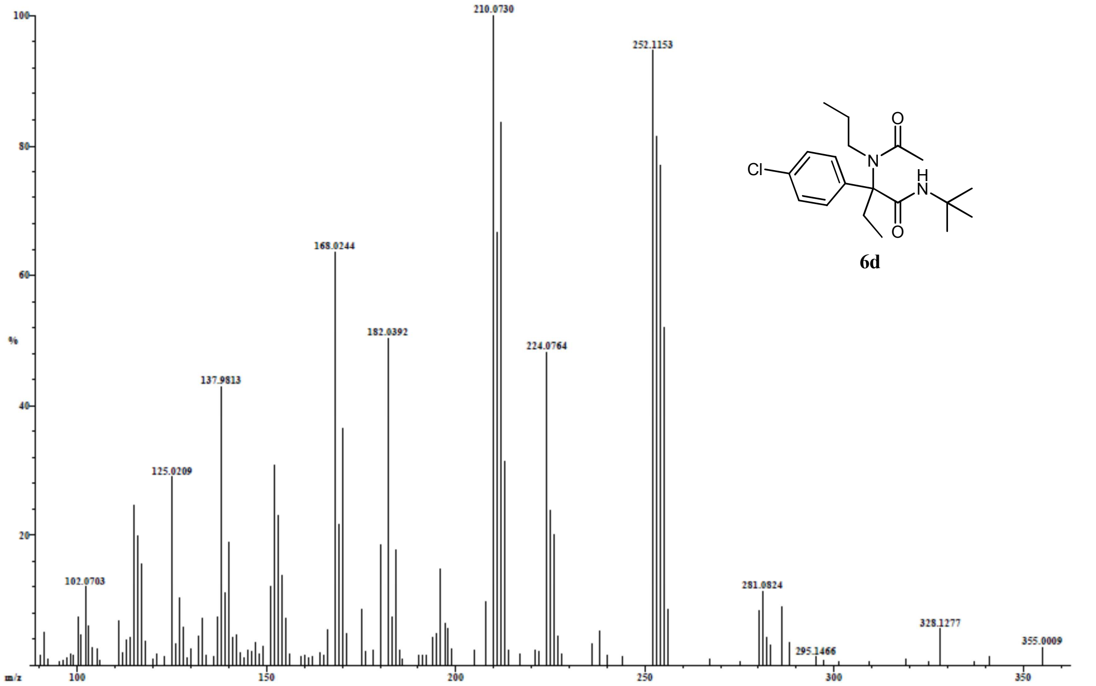

6d

Laboratoire de Synthèse Organique, Palaiseau, France 02/12/2016 12:16:00

File: za325 Date Run: 02-12-2016 (Time Run: 09:05:42)

Sample: Instrument: JEOL JMSGCmateII Ionization mode: EI+ Run By: Vincent Jactel

C20H23ClNO 328,1480 uma 67% **Scan: 117** **TIC: 3101296**

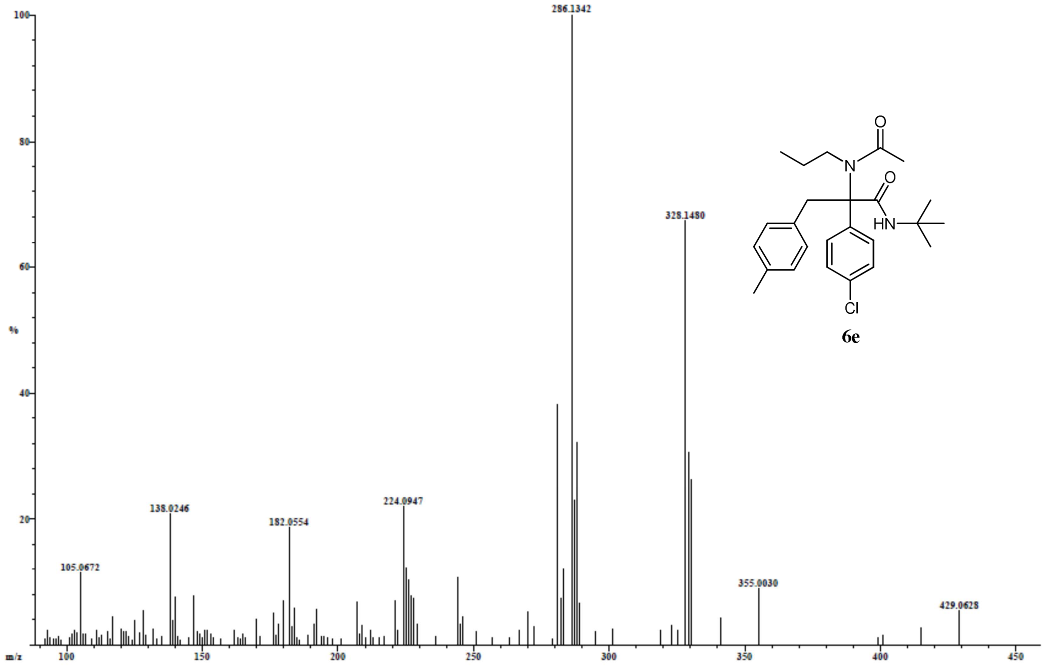

6e

Laboratoire de Synthèse Organique 17/11/2016 17:16:13

File: ZA164H 2 Date Run: 11-17-2016 (Time Run: 14:13:09)

Sample: ZA164H Instrument: JEOL JMSGCmateII Ionization mode: EI+ Run By: Vincent Jactel

C13H13ClNO 234,0887 uma 19 % L'absence du pic des molécules avec l'isotope 37Cl est due à un dysfonctionnement du spectromètre. TIC: 1157120

Scan: 53

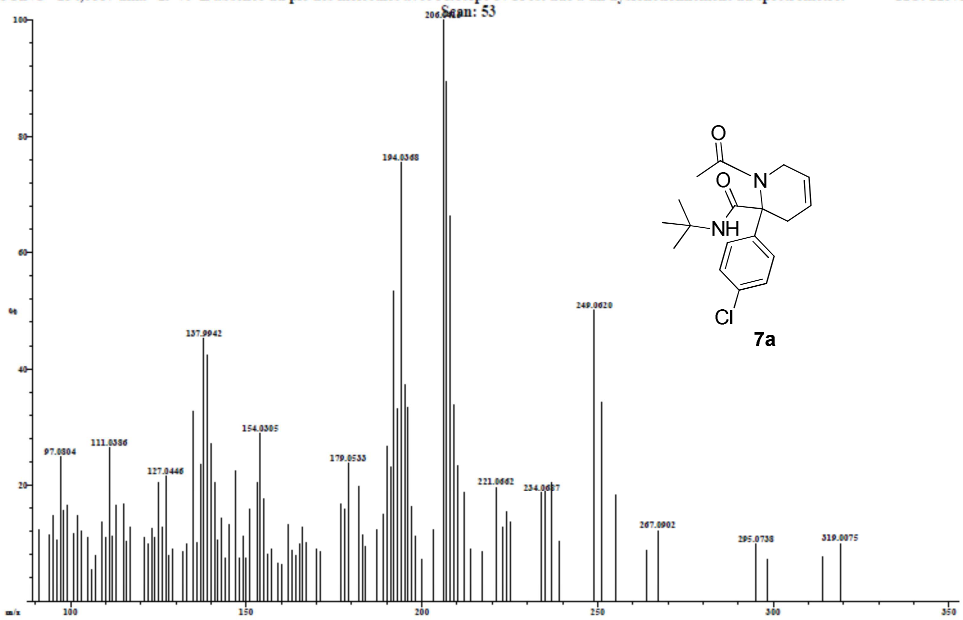

Laboratoire de Synthèse Organique, Palaiseau, France 04/11/2016 16:46:17

File: ZA207 Date Run: 04-11-2016 (Time Run: 16:38:43) Run By: Vincent Jactel

Sample: Instrument: JEOL JMSGCmateII Ionization mode: EI+

C20H25ClN2O2 360,1613 amu 1% Scan: 229 TIC: 3479104

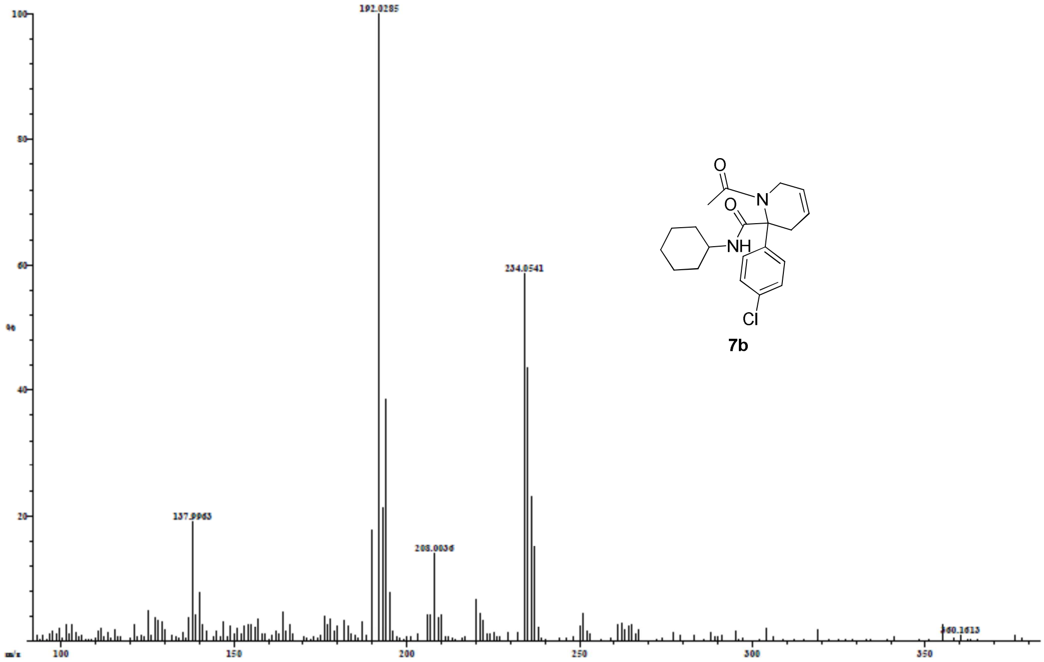

Laboratoire de Synthèse Organique, Palaiseau, France 04/11/2016 13:33:10

File: ZA213 Date Run: 04-11-2016 (Time Run: 13:22:23) Run By: Vincent Jactel
Sample: Instrument: JEOL JMSGCmateII Ionization mode: EI+

C17H23N3O2 301,1801 amu 1 % **Scan: 298** **TIC: 9884204**

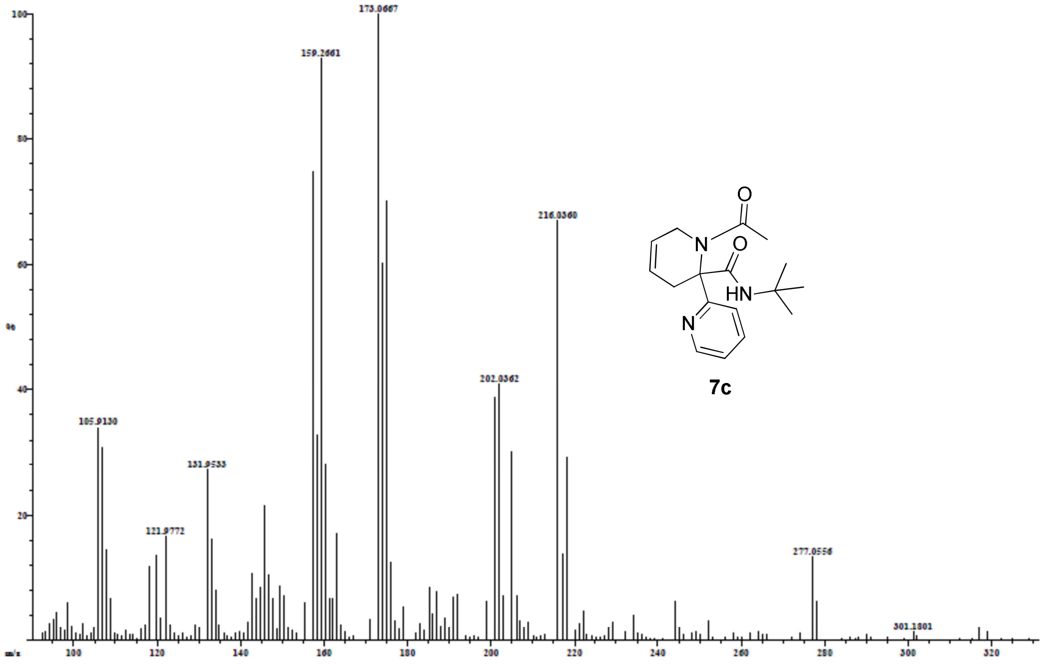

Laboratoire de Synthèse Organique, Palaiseau, France 04/11/2016 16:22:42

File: ZA214 Date Run: 04-11-2016 (Time Run: 16:05:03) Run By: Vincent Jactel
Sample: Instrument: JEOL JMSGCmateII Ionization mode: EI+

C24H27ClN2O3 426,1729 amu 1 % **Scan: 362** **TIC: 5358100**

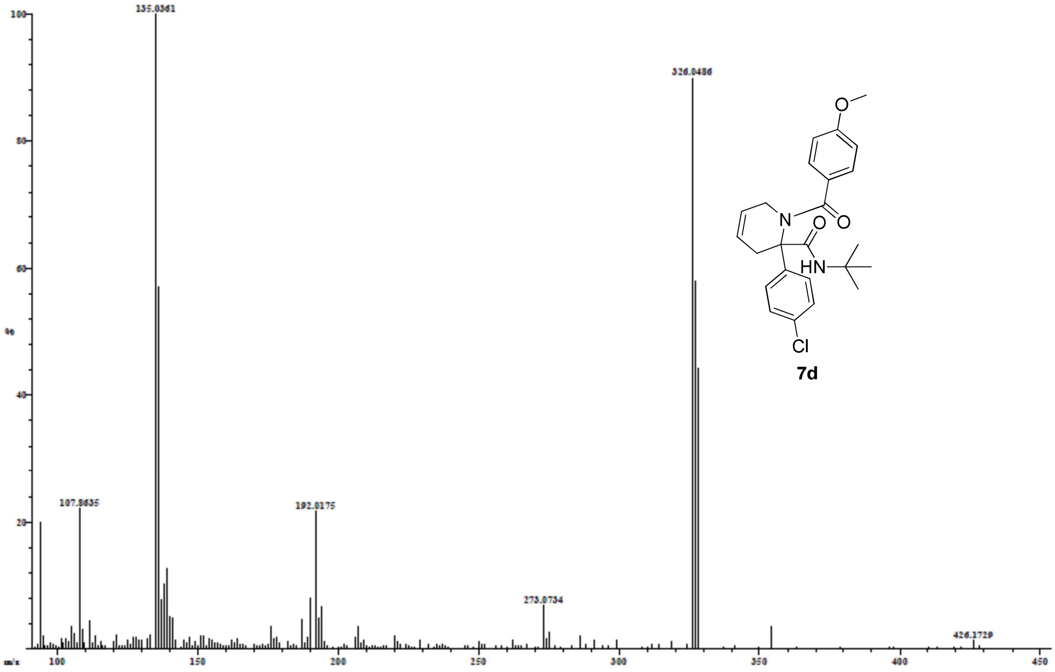

Laboratoire de Synthèse Organique 17/11/2016 16:29:10

File: ZA277 Date Run: 11-17-2016 (Time Run: 16:08:47)
Sample: Instrument: JEOL JMSGCmateII Ionization mode: EI+ Run By: Vincent Jactel

C26H31ClN2O3 454,2012 amu 2% L'absence du pic des molécules avec l'isotope 37Cl est due à un dysfonctionnement du spectromètre. TIC: 6104624

Scan: 132

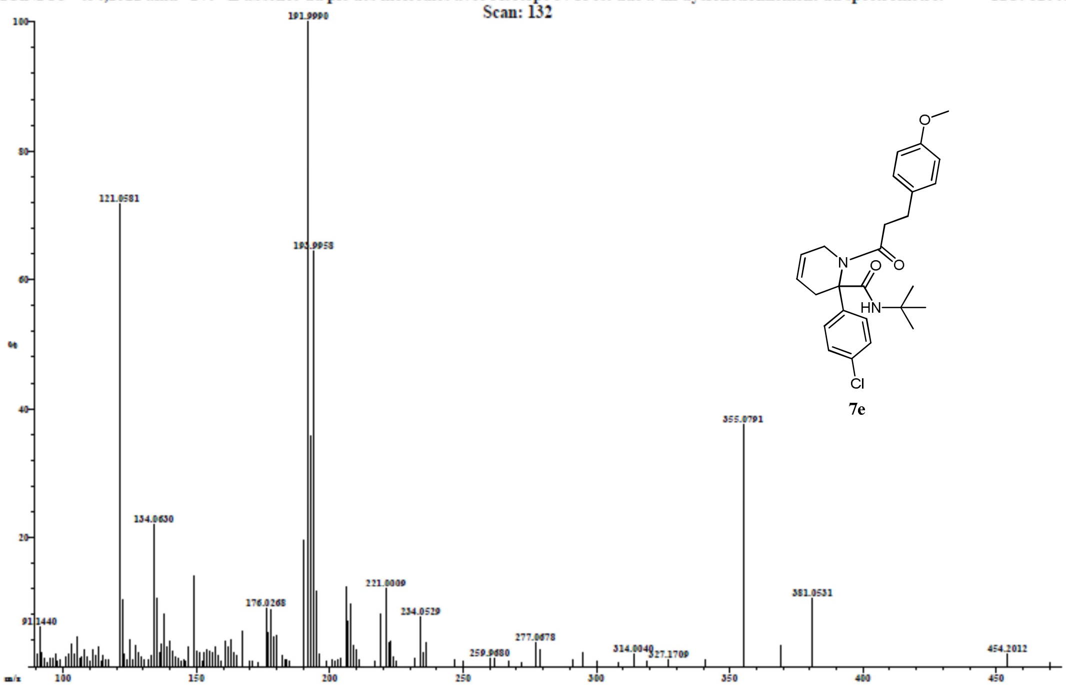

Laboratoire de Synthèse Organique, Palaiseau, France 22/12/2016 15:21:07

File: ZA331 Date Run: 12-22-2016 (Time Run: 15:14:18)
Sample: Instrument: JEOL JMSGCmateII Ionization mode: EI+ Run By: Vincent Jactel

C14H15ClNO 248.0831 amu 100% Scan: 94 TIC: 2635008

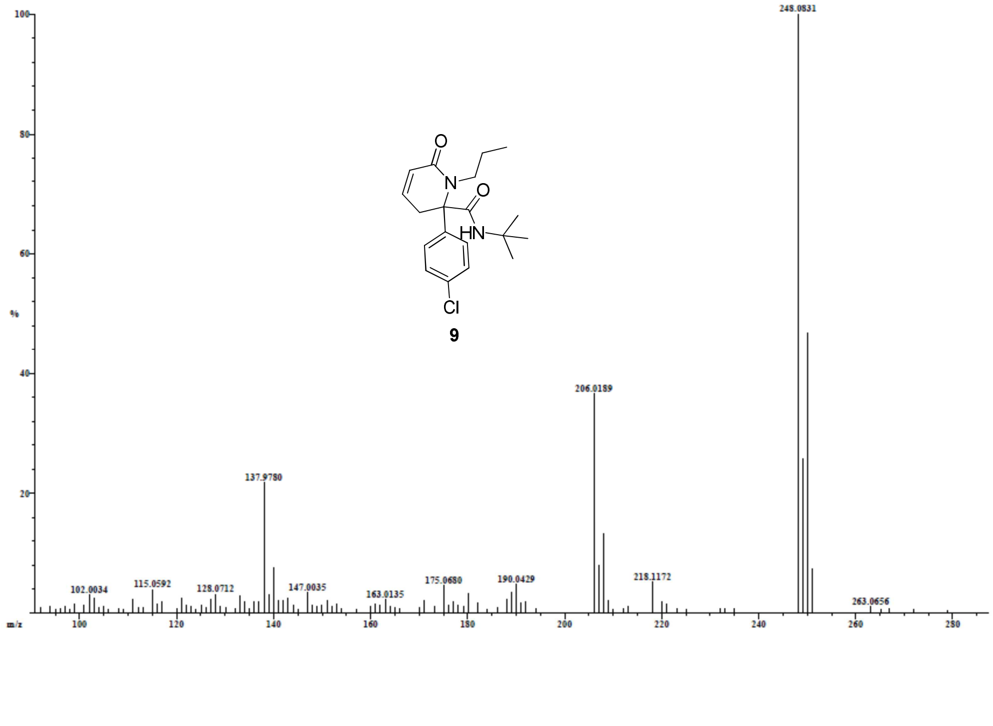

Laboratoire de Synthèse Organique, Palaiseau, France

27/09/2016 18:02:37

File: ZA165
Sample:

Date Run: 09-27-2016 (Time Run: 17:54:54)
Instrument: JEOL JMSGCmateII

Inlet: Direct Probe

Run By: Vincent Jactel
Ionization mode: EI+

C18H23ClN2O2 334,1444 27 %

Scan: 76

TIC: 3364638

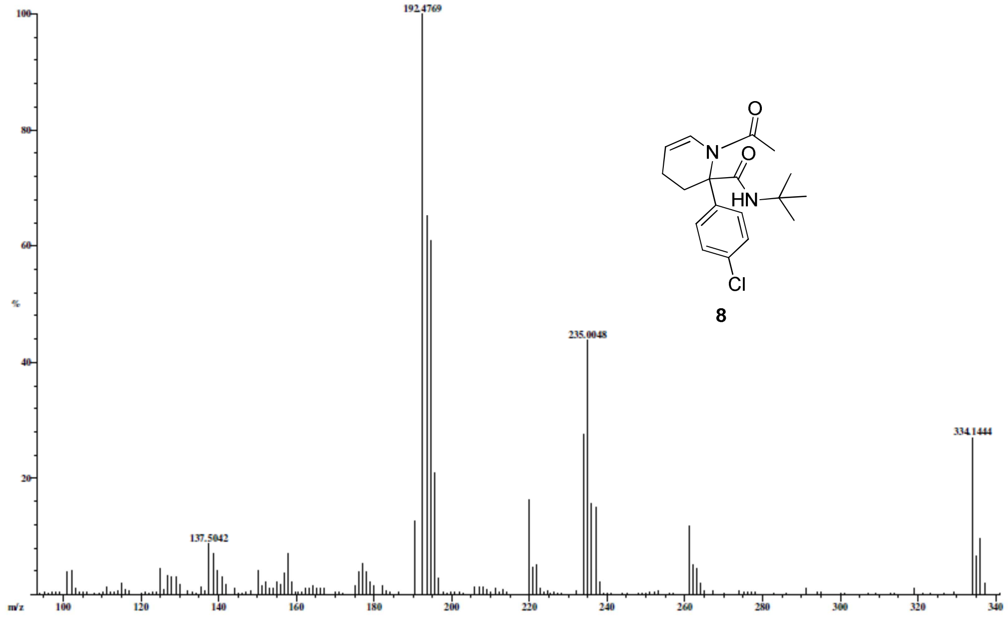

Supplement: Supplementary file 1 [file Data_Sheet_1.PDF]
